# Supplementary material for: A Bayesian Approach for Analysis of Whole-Genome Bisulfite Sequencing Data Identifies Disease-Associated Changes in DNA Methylation
Source: Genetics. 2017 Feb 16;205(4):1443–58. doi: 10.1534/genetics.116.195008 (PMC5378105; doi:10.1534/genetics.116.195008)

Supplementary Figure 1. Correlation of methylation levels (number of methylated reads over the total number of reads per CpG) for varying CpGs-distances. Spearman's rank correlation (*y*-axis) of methylation levels in eight rats on chromosome 20 (see Methods for details on WGBS data generation in rat macrophages) is calculated between CpG sites that are up to 8,000 base pairs apart or 3 CpG shores (*x*-axis). Blue solid line depicts the General Additive Model (GAM) smoothed line.

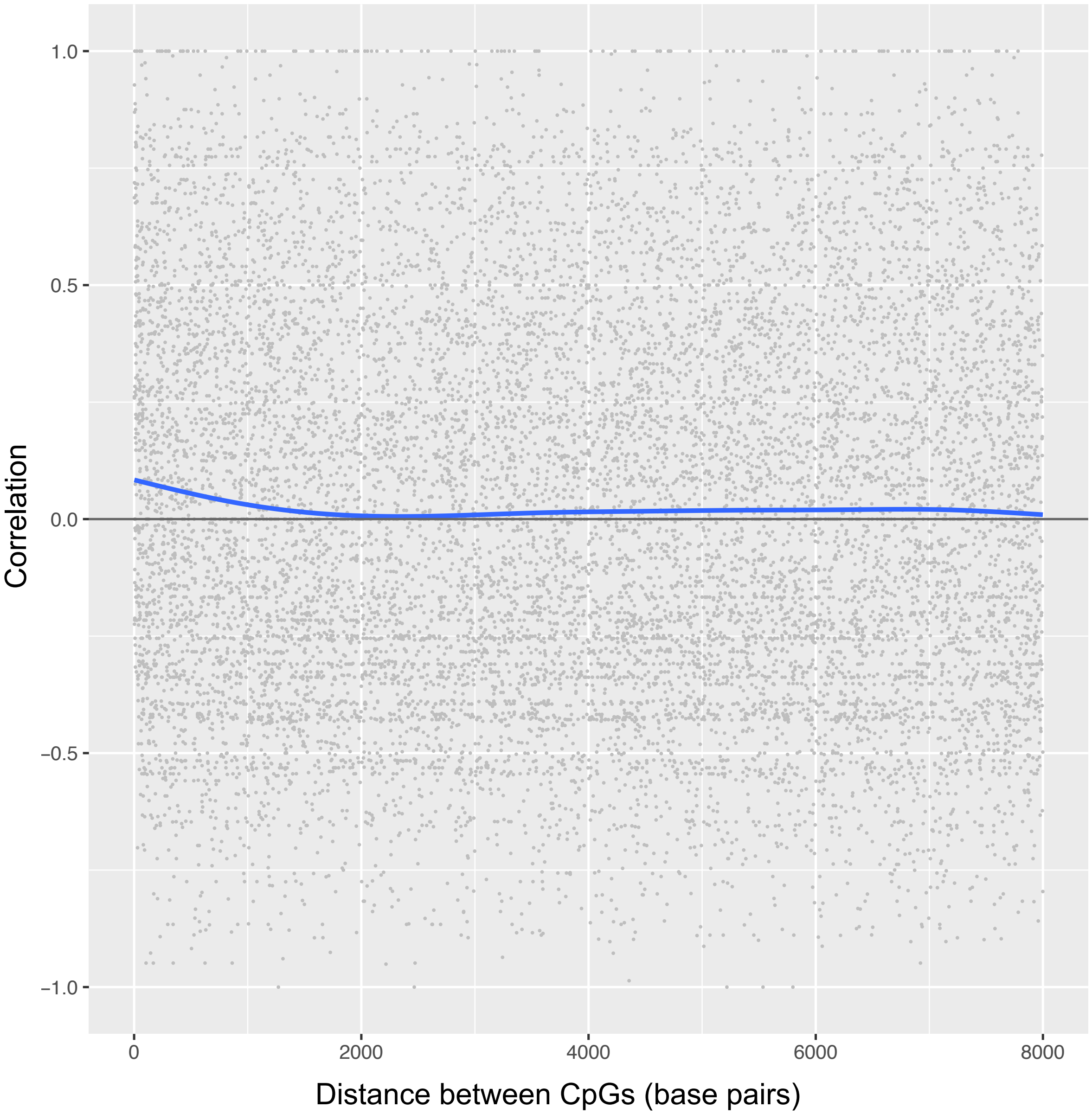

Supplement: Supplementary file 1 [file 1443FigureS1.pdf]
