# Supplementary material for: A Bayesian Approach for Analysis of Whole-Genome Bisulfite Sequencing Data Identifies Disease-Associated Changes in DNA Methylation
Source: Genetics. 2017 Feb 16;205(4):1443–58. doi: 10.1534/genetics.116.195008 (PMC5378105; doi:10.1534/genetics.116.195008)

Supplementary Figure 2. Three-component mixture model used to distinguish between the null distribution (no differential methylation) and the alternatives. **(a)** Histogram of the genome-wide posterior differential methylation probabilities (PDMP) with kernel density plot superimposed (solid black line). **(b)** Mixture model plot superimposed to the kernel density plot. Blue dotted line shows the density of the mixture null component, while red and green dotted lines depict the "negative" and "positive" differentially methylated density, respectively.

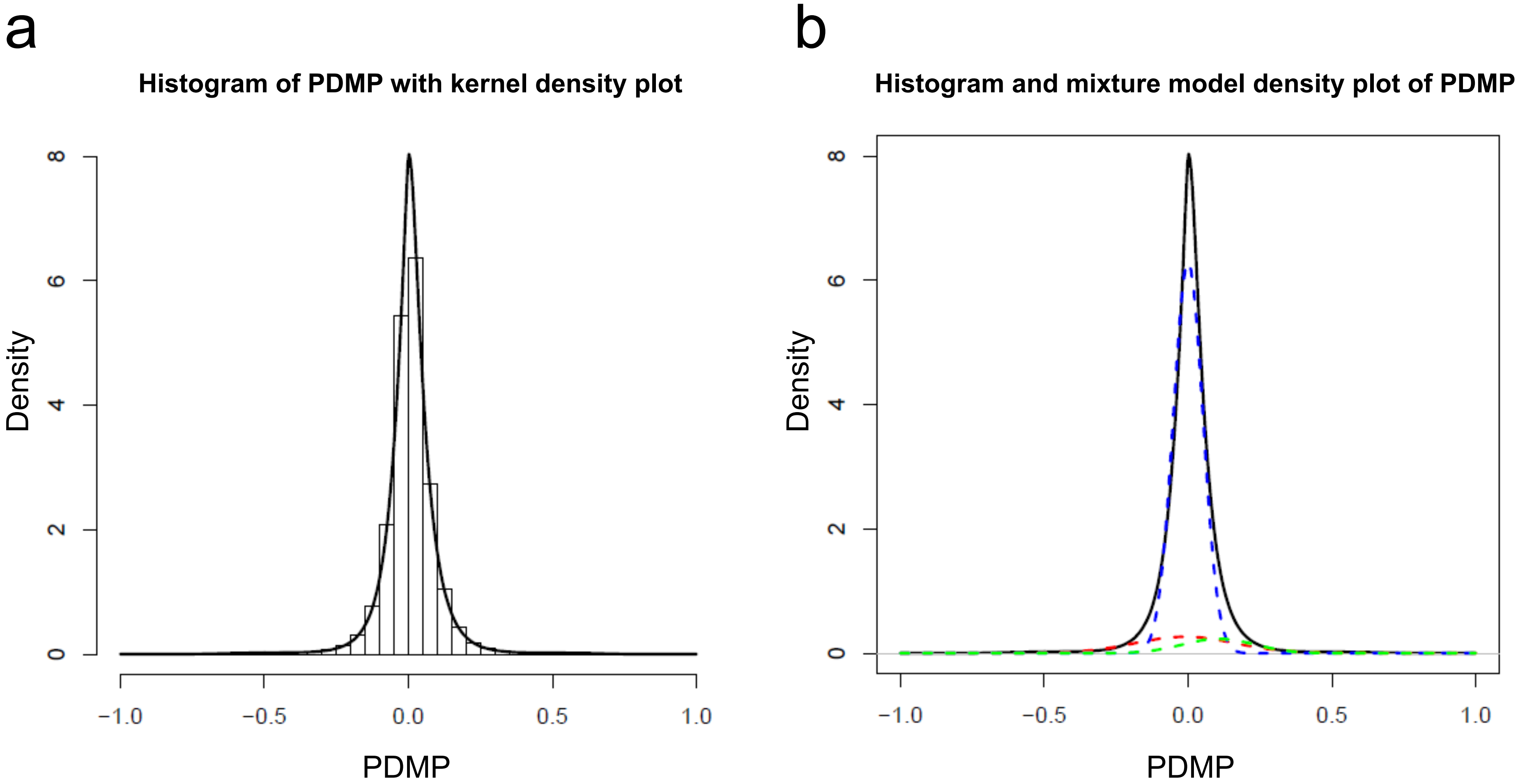

Supplement: Supplementary file 2 [file 1443FigureS2.pdf]
