# Supplementary material for: A Bayesian Approach for Analysis of Whole-Genome Bisulfite Sequencing Data Identifies Disease-Associated Changes in DNA Methylation
Source: Genetics. 2017 Feb 16;205(4):1443–58. doi: 10.1534/genetics.116.195008 (PMC5378105; doi:10.1534/genetics.116.195008)

Supplementary Figure 3. Size distribution of simulated DMRs used in the benchmarking experiments. Median size is 15 CpGs while average size is 24 CpGs with standard deviation 24 CpGs.

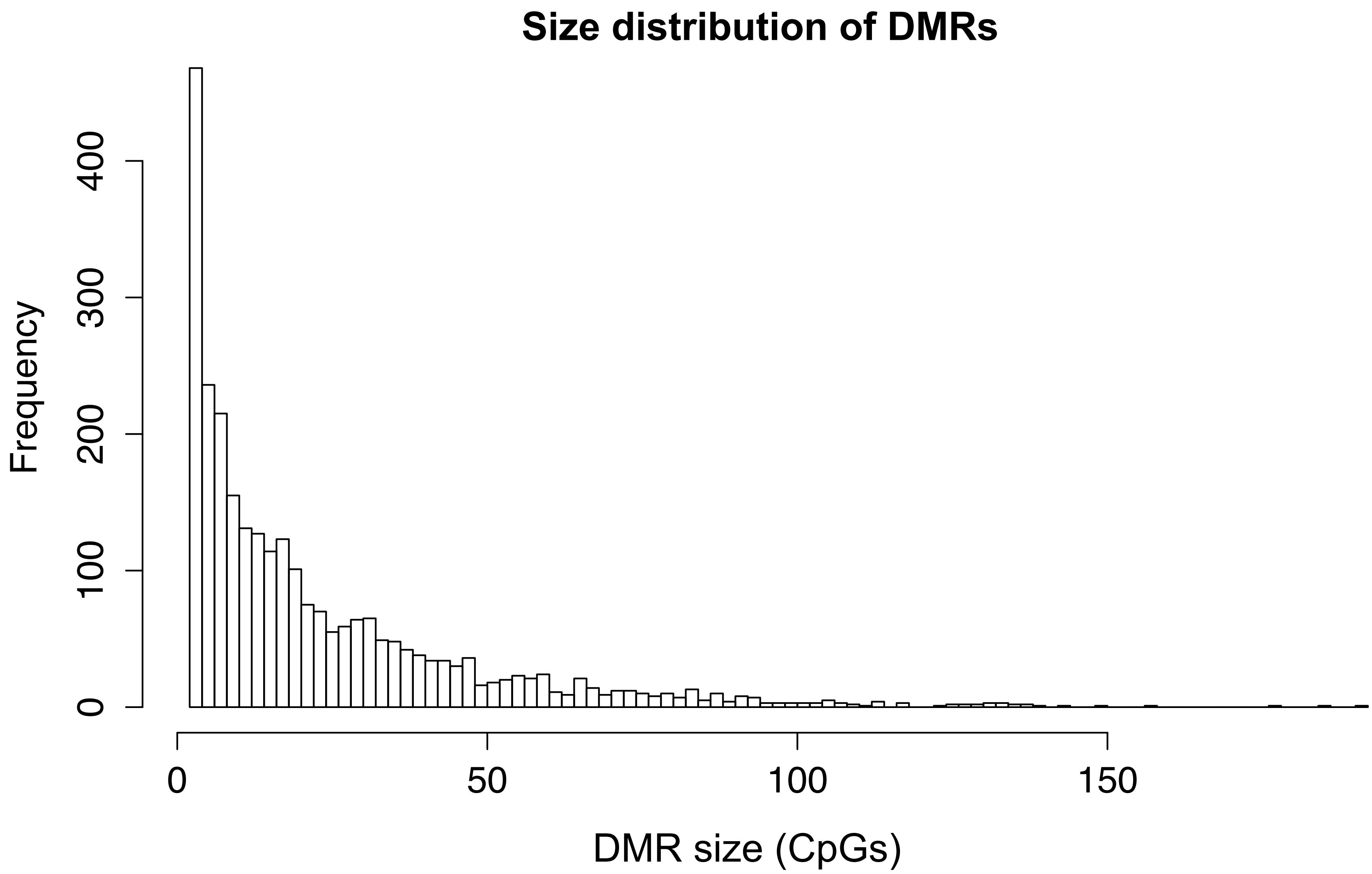

Supplement: Supplementary file 3 [file 1443FigureS3.pdf]
