# Supplementary material for: A Bayesian Approach for Analysis of Whole-Genome Bisulfite Sequencing Data Identifies Disease-Associated Changes in DNA Methylation
Source: Genetics. 2017 Feb 16;205(4):1443–58. doi: 10.1534/genetics.116.195008 (PMC5378105; doi:10.1534/genetics.116.195008)

Supplementary Figure 4. Histograms of d_i's and the posterior mean differential methylation between two groups (see Methods), obtained by ABBA using data from d_i's from a contiguous 45,300 CpG stretch of the genome (red) and the corresponding d_i's which were obtained by ABBA using the same stretch broken down into smaller chunks (blue).

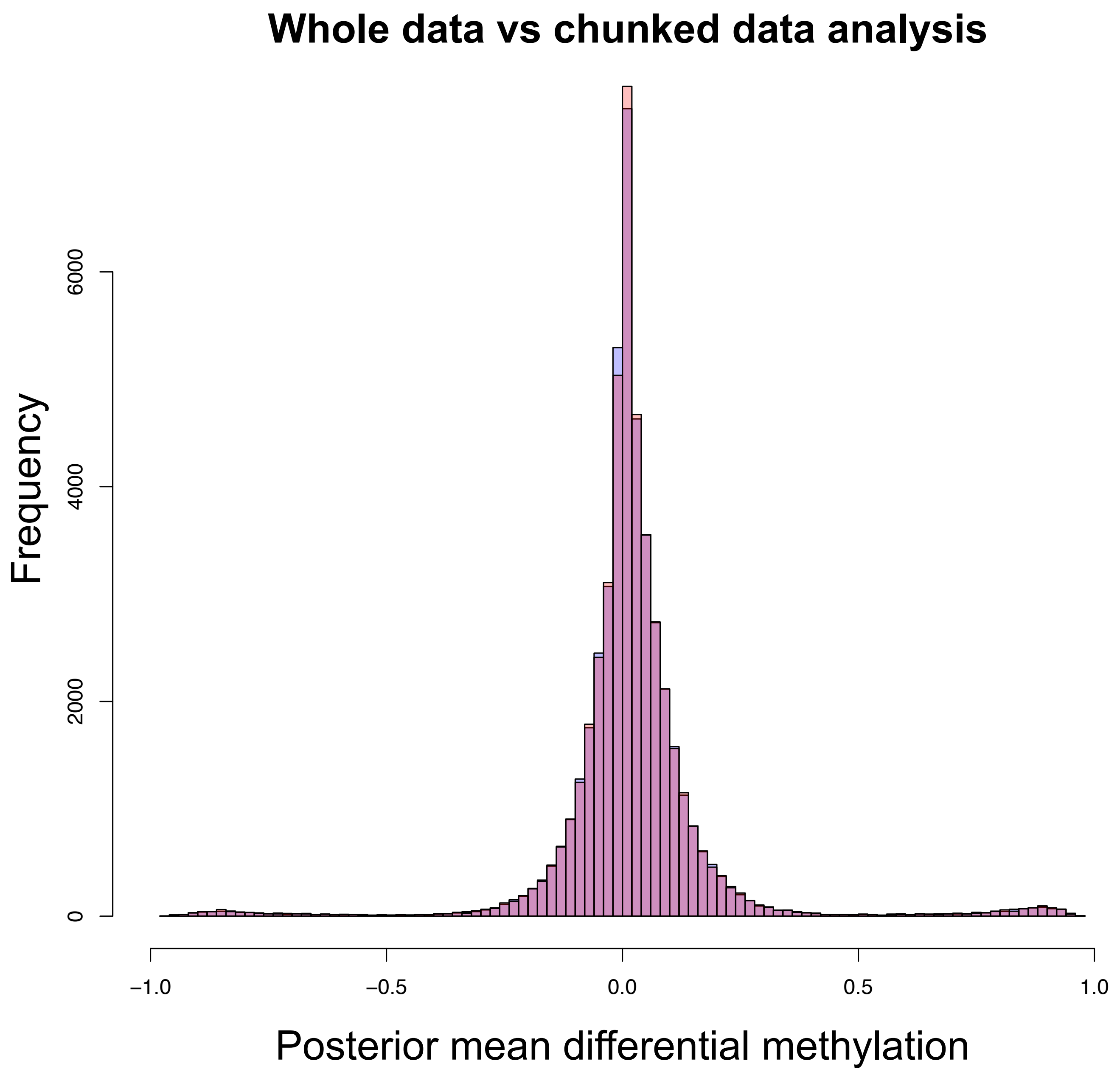

Supplement: Supplementary file 4 [file 1443FigureS4.pdf]
