# Supplementary material for: A Bayesian Approach for Analysis of Whole-Genome Bisulfite Sequencing Data Identifies Disease-Associated Changes in DNA Methylation
Source: Genetics. 2017 Feb 16;205(4):1443–58. doi: 10.1534/genetics.116.195008 (PMC5378105; doi:10.1534/genetics.116.195008)

**Supplementary Figure 6a:** ROC curves showing the performance of published techniques and ABBA over various experimentally relevant parameter settings, for a description of parameters see Methods. Parameters: $\underline{s_0=0.1}$, δ = 5%, and rows 1-3 refer to *r*=1 to *r*=3.

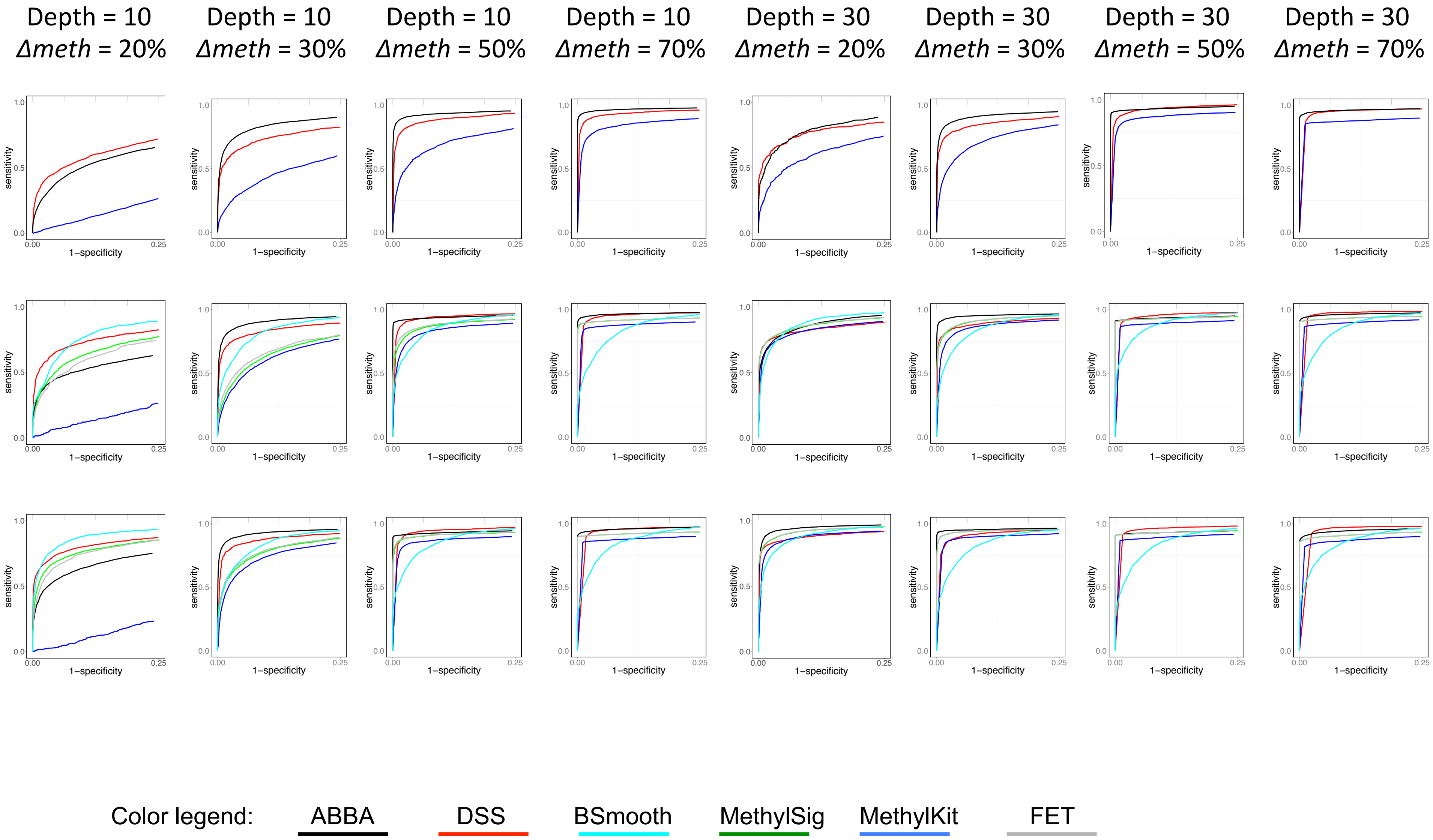

**Supplementary Figure 6b:** ROC curves showing the performance of published techniques and ABBA over various experimentally relevant parameter settings, for a description of parameters see Methods. Parameters: $s_0$=0.2, δ = 5%, and rows 1-3 refer to *r*=1 to *r*=3.

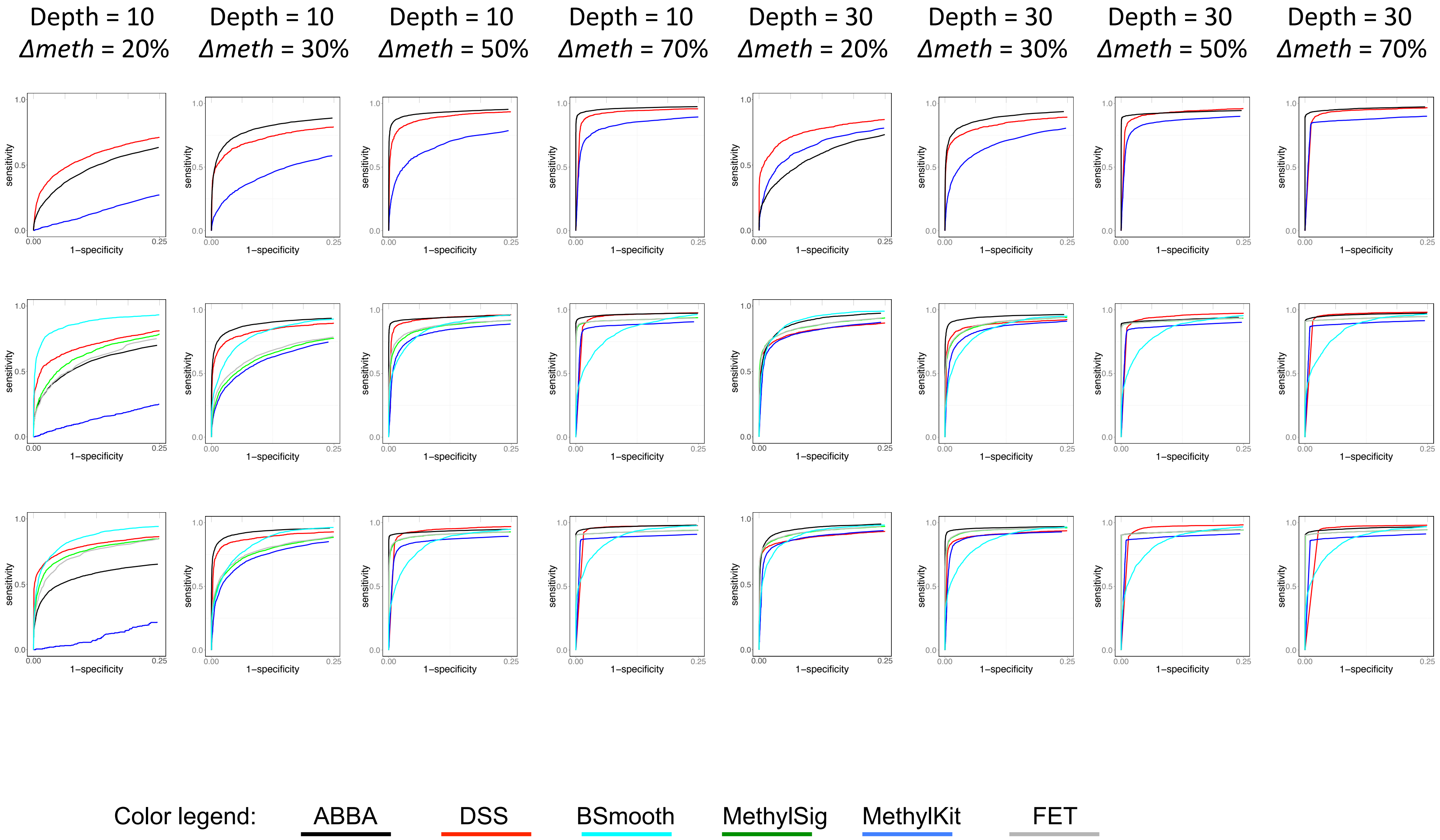

**Supplementary Figure 6c:** ROC curves showing the performance of published techniques and ABBA over various experimentally relevant parameter settings, for a description of parameters see Methods. Parameters: $\underline{s_0=0.3}$, $\delta$ = 5%, and rows 1-3 refer to *r*=1 to *r*=3.

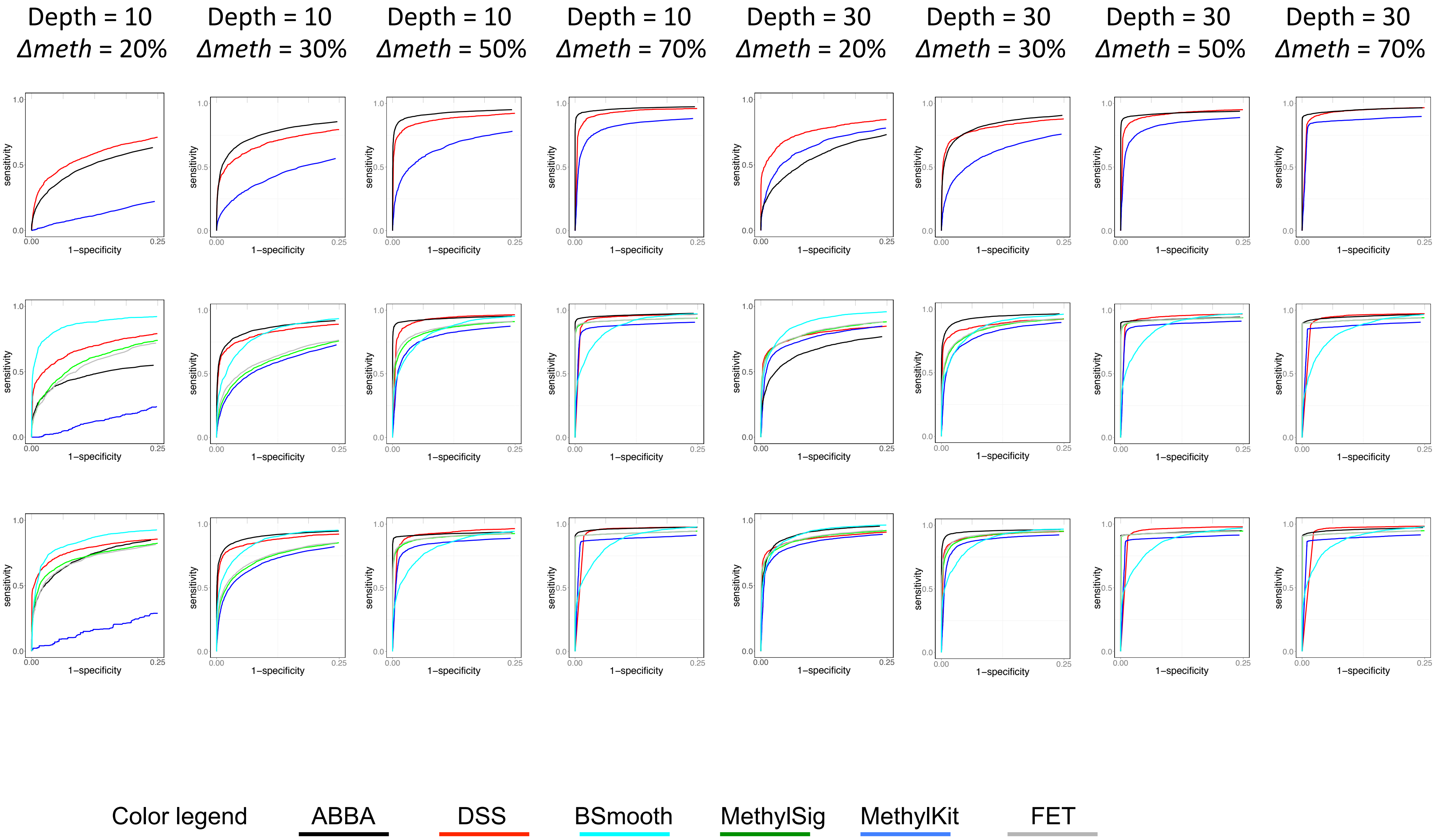

Supplement: Supplementary file 6 [file 1443FigureS6.pdf]
