# Supplementary material for: A Bayesian Approach for Analysis of Whole-Genome Bisulfite Sequencing Data Identifies Disease-Associated Changes in DNA Methylation
Source: Genetics. 2017 Feb 16;205(4):1443–58. doi: 10.1534/genetics.116.195008 (PMC5378105; doi:10.1534/genetics.116.195008)

Supplementary Figure 7a: ROC curves showing the performance of published techniques and ABBA over various experimentally relevant parameter settings, for a description of parameters see Methods. Parameters: s_0=0.1, δ = 10%, and rows 1-3 refer to *r*=1 to *r*=3.

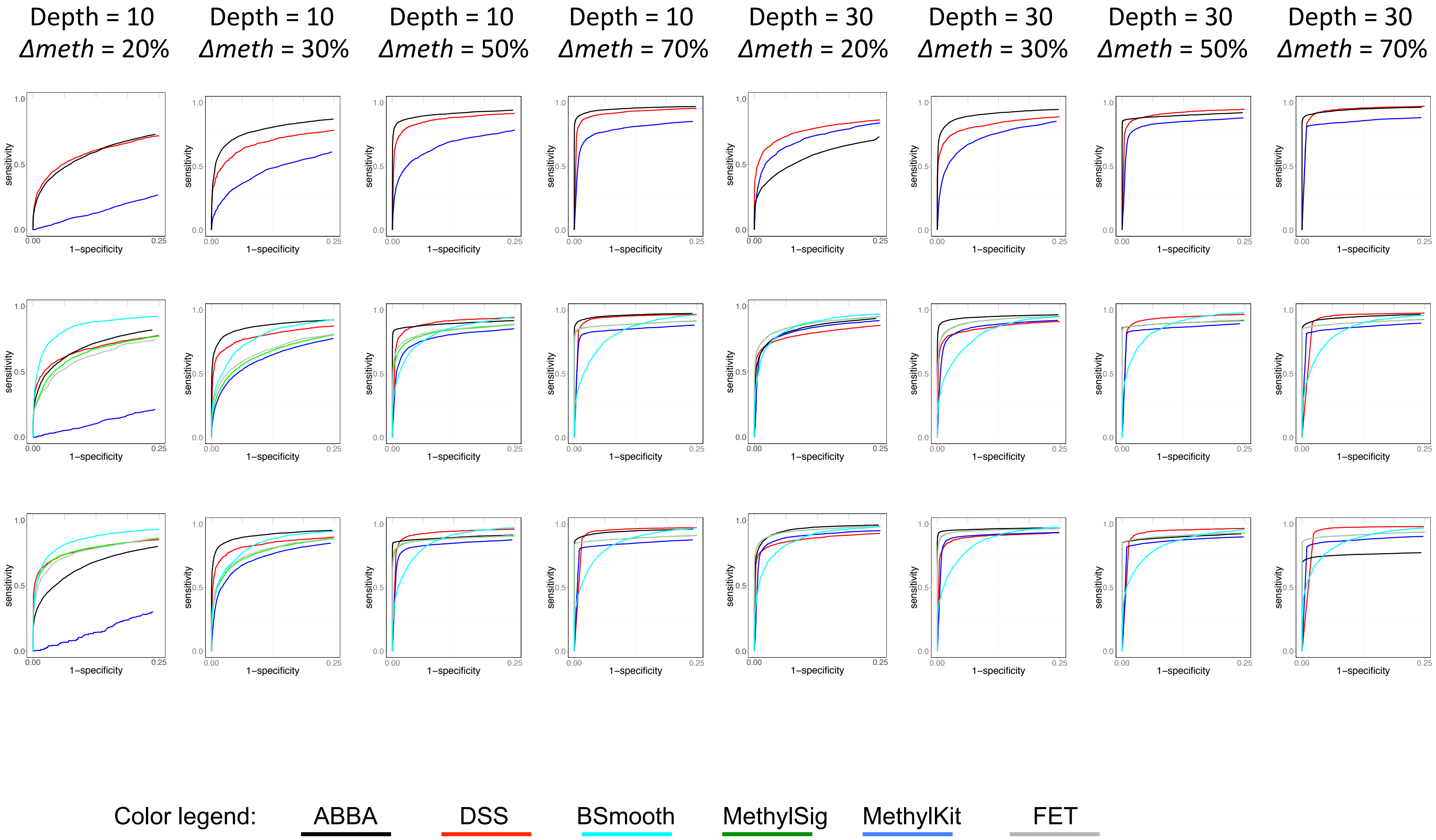

Supplementary Figure 7b: ROC curves showing the performance of published techniques and ABBA over various experimentally relevant parameter settings, for a description of parameters see Methods. Parameters: $\underline{s_0=0.2}$, δ = 10%, and rows 1-3 refer to *r*=1 to *r*=3.

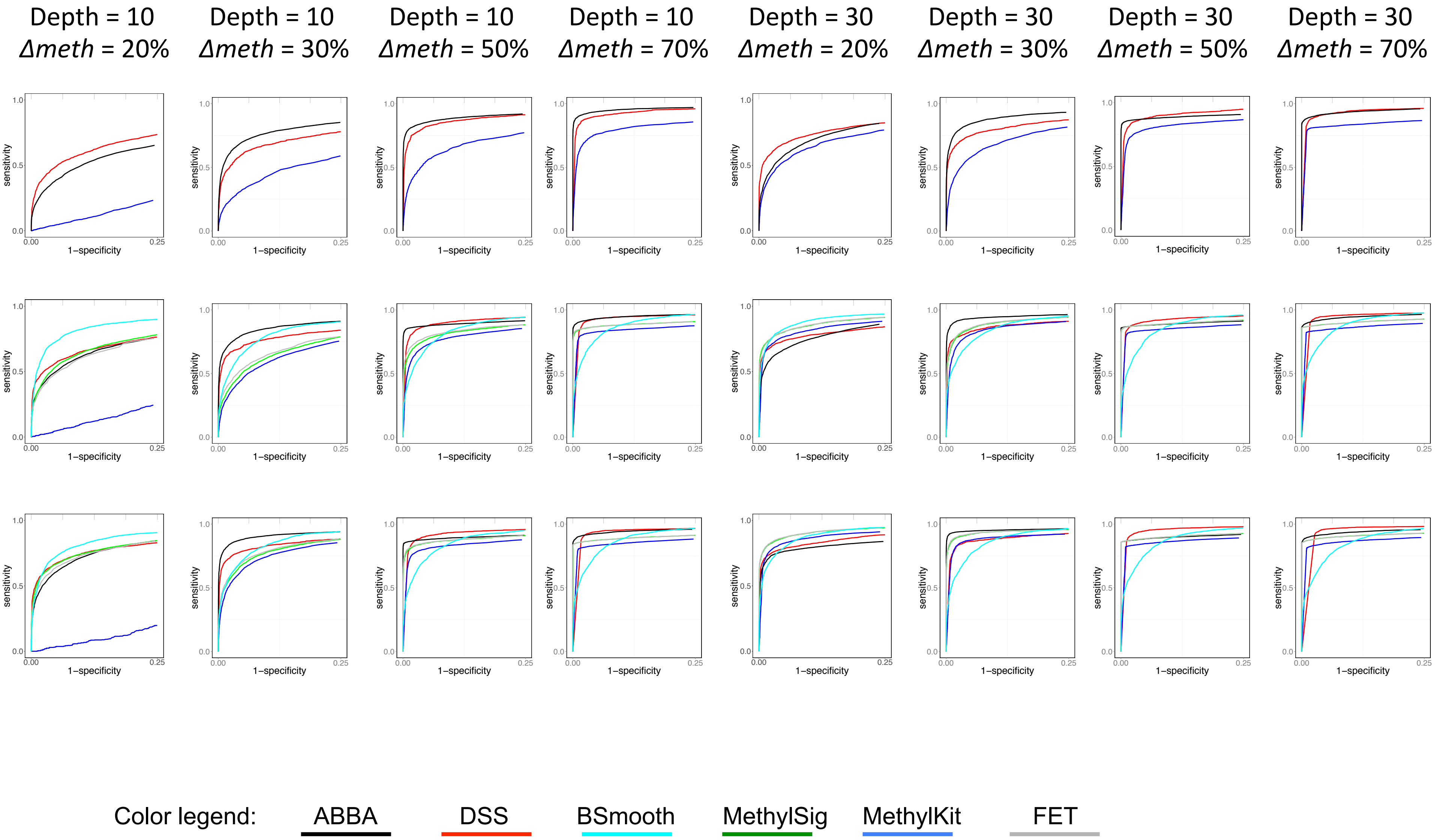

Supplementary Figure 7c: ROC curves showing the performance of published techniques and ABBA over various experimentally relevant parameter settings, for a description of parameters see Methods. Parameters: s_0=*0.3*, δ = 10%, and rows 1-3 refer to *r*=1 to *r*=3.

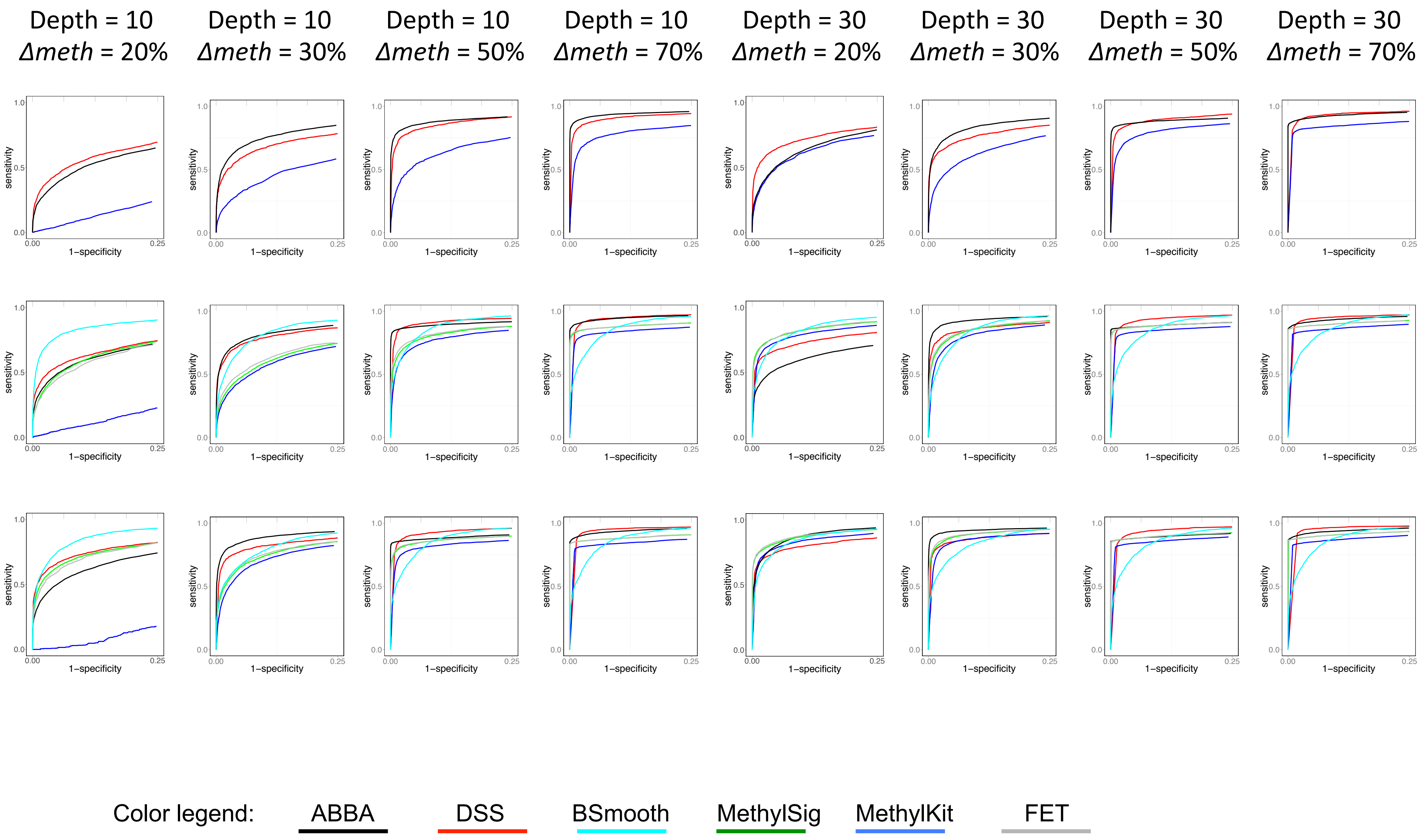

Supplement: Supplementary file 7 [file 1443FigureS7.pdf]
