# Supplementary material for: A Bayesian Approach for Analysis of Whole-Genome Bisulfite Sequencing Data Identifies Disease-Associated Changes in DNA Methylation
Source: Genetics. 2017 Feb 16;205(4):1443–58. doi: 10.1534/genetics.116.195008 (PMC5378105; doi:10.1534/genetics.116.195008)

Supplementary Figure 8. Representative examples for the ROC curves obtained by running metilene in comparison with other methods tested. Parameterizations for the simulations used to generate these ROC curves are as follows: (i) s_0 = 0.1, *Δmeth* = 30%, *r* = 1, average read depth per CpG of 10x, δ = 0; (ii) s_0 = 0.3, *Δmeth* = 30%, *r* = 3, average read depth per CpG of 10x, δ = 0; (iii) s_0 = 0.2, *Δmeth* = 70%, *r* = 2, average read depth per CpG of 30x, δ = 0; (iv) s_0 = 0.1, *Δmeth* = 70%, *r* = 1, average read depth per CpG of 30x, δ = 5%; (v) s_0 = 0.2, *Δmeth* = 30%, *r* = 2, average read depth per CpG of 10x, δ = 10%; (vi) s_0 = 0.3, *Δmeth* = 70%, *r* = 3, average read depth per CpG of 30x, δ = 10%. Yellow dots indicate the individual results obtained from metilene, which have been averaged and then interpolated to generate the ROC for this approach (magenta line).

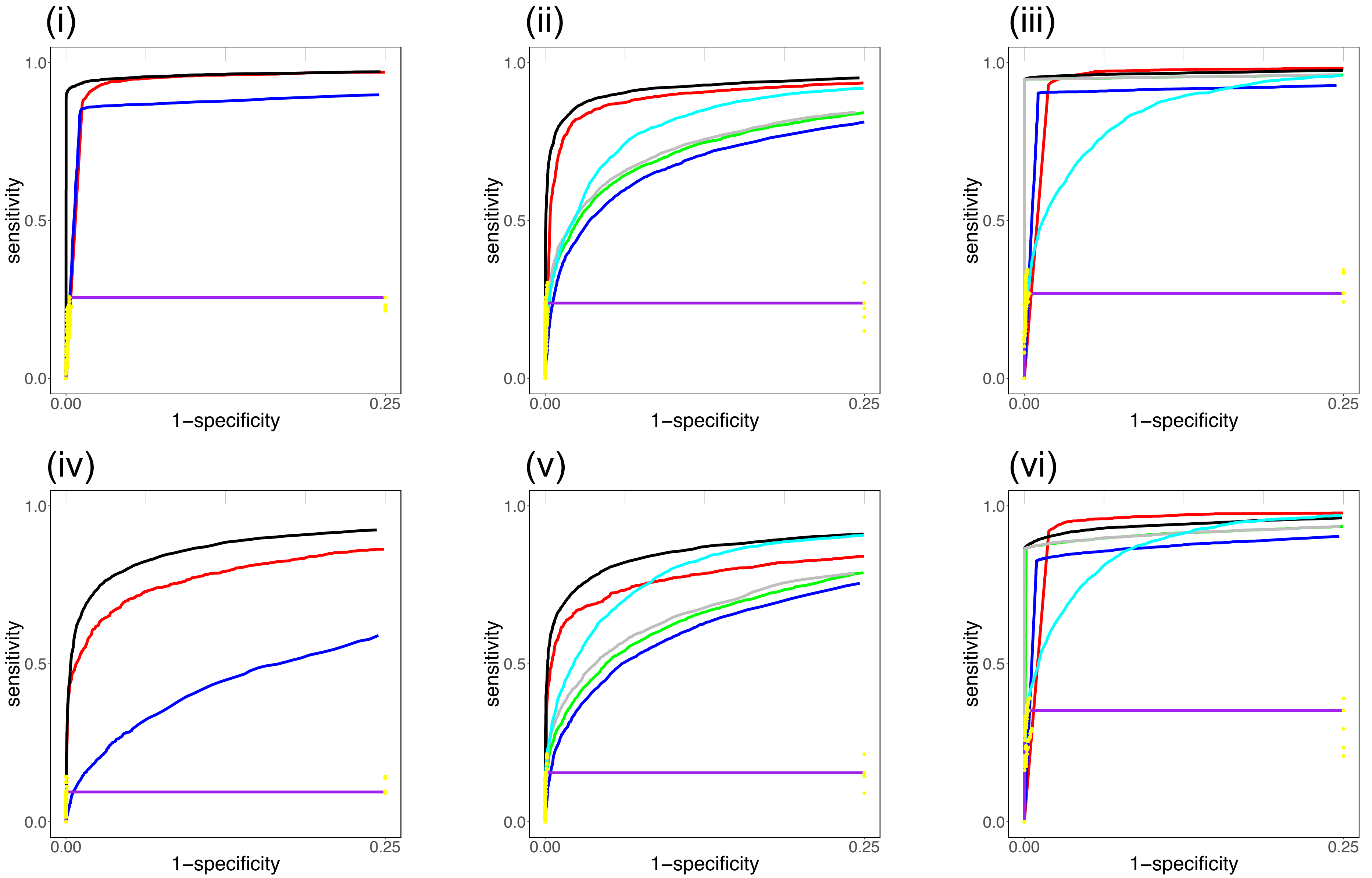

Supplement: Supplementary file 8 [file 1443FigureS8.pdf]
