# Supplementary material for: A Bayesian Approach for Analysis of Whole-Genome Bisulfite Sequencing Data Identifies Disease-Associated Changes in DNA Methylation
Source: Genetics. 2017 Feb 16;205(4):1443–58. doi: 10.1534/genetics.116.195008 (PMC5378105; doi:10.1534/genetics.116.195008)

Supplementary Figure 9a. Correlation between CpG read depths for varying CpG distances. Spearman's rank correlation (*y*-axis) of CpG read depths in eight rats on chromosome 1 containing 5,000 CpGs (see Methods for details on WGBS data generation in rat macrophages) is calculated between CpG sites that are up to 8,000 base pairs apart (*x*-axis). A strong positive spatial correlation can be observed, particularly for CpGs within <1kbp distance and this pattern is representative of what observed in the rest of the rat genome. This set of 5,000 CpGs on chromosome 1 was used to generate "data-derived" simulated dataset of DMRs using WGBSSuite (see main text and Methods for details).

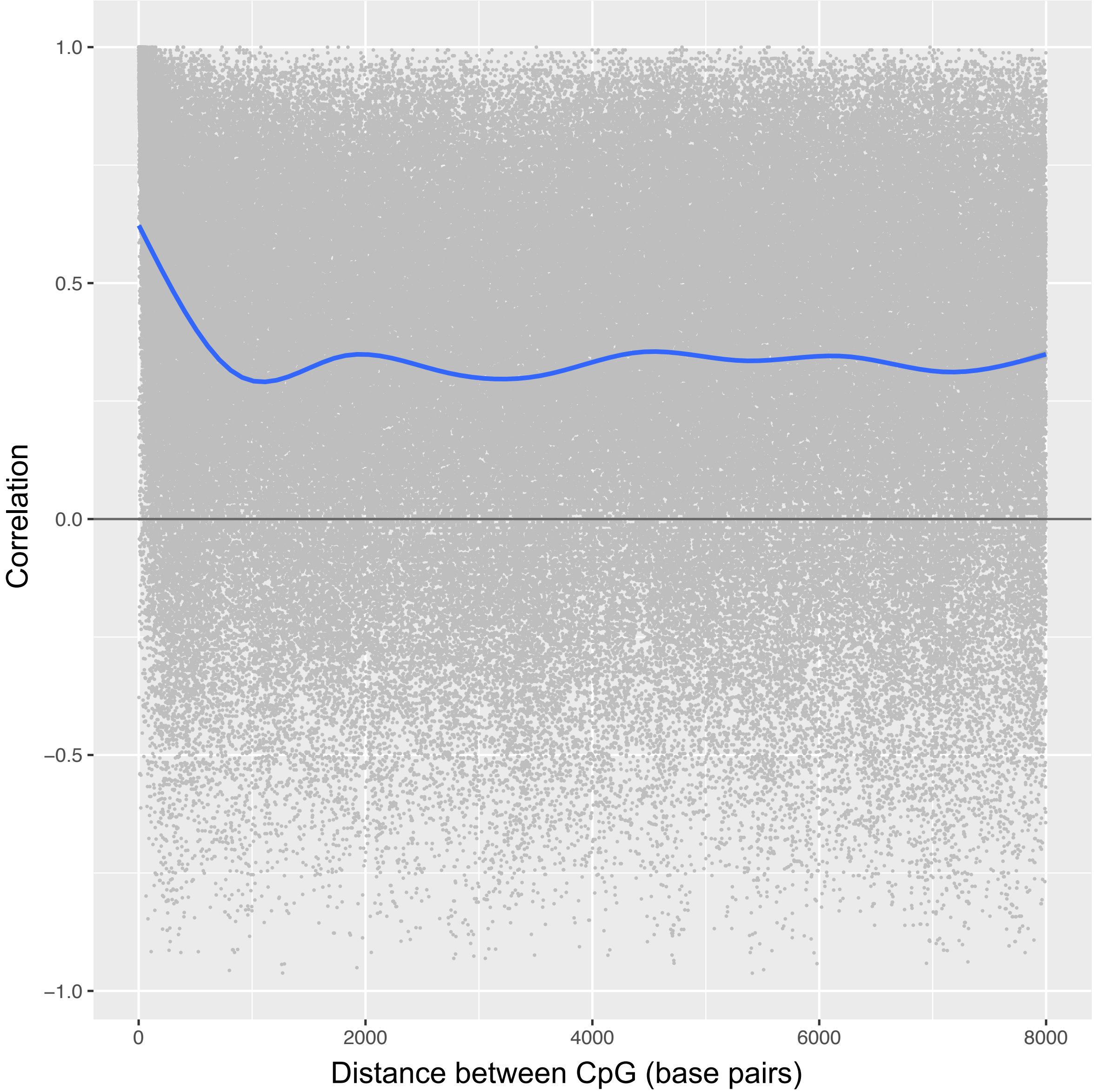

Supplementary Figure 9b. Representative ROC curves for the comparison between the benchmark results obtained using "data-derived read depth" and when the read depth was simulated using a Poisson distribution. In the "*Poisson-simulated read depth*" the number of reads per CpG site is simulated using a Poisson distribution (see Methods) whereas in the "*Data-derived read depth*" the read depth is taken from real WGBS data generated in the rat (see Methods for details on data generation). To avoid any bias in the comparison, in each case the parameters of the "*Poisson-simulated read depth*" were adapted to match the "*Data-derived read depth*" (average read depth 6.7636x). MethylKit performance is not represented here as this method did not return any results on this analysis, possibly due to the low read depth in these "*Data-derived read depth*" simulated data.

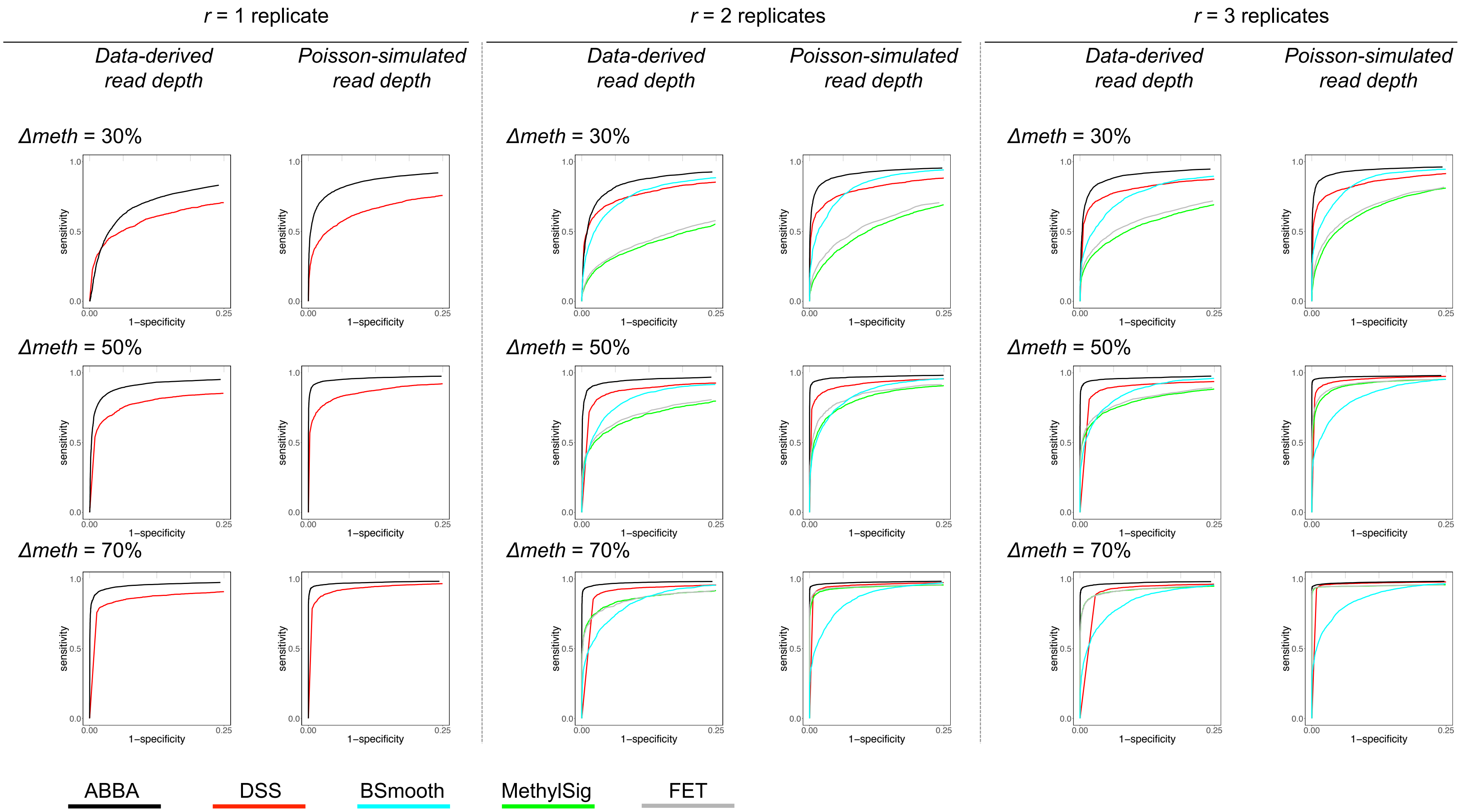

Supplement: Supplementary file 9 [file 1443FigureS9.pdf]
