# Supplementary material for: A Bayesian Approach for Analysis of Whole-Genome Bisulfite Sequencing Data Identifies Disease-Associated Changes in DNA Methylation
Source: Genetics. 2017 Feb 16;205(4):1443–58. doi: 10.1534/genetics.116.195008 (PMC5378105; doi:10.1534/genetics.116.195008)

Supplementary Figure 10. DSS analysis of WGBS in rat macrophages for different choices of window size. **(a)** The overlap and number of genes with an associated DMR identified by DSS for four window sizes (50 bp, 100 bp, 500 bp, 1,000 bp). Distribution of DMR lengths (bp) identified for the window sizes of **(b)** 50 bp, **(c)** 100 bp, **(d)** 500 bp and **(e)** 1,000 bp.

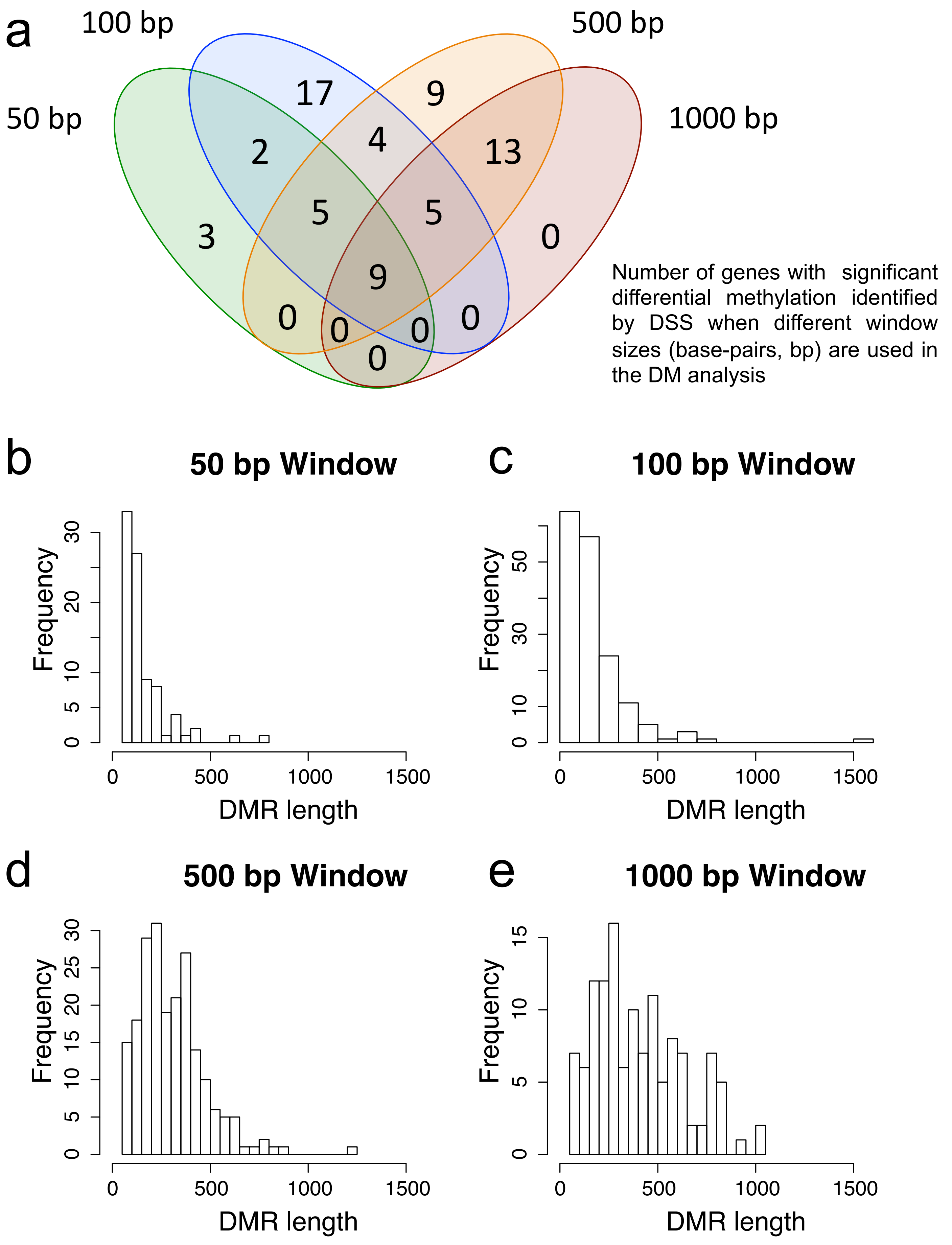

Supplement: Supplementary file 10 [file 1443FigureS10.pdf]
