# Supplementary material for: A Bayesian Approach for Analysis of Whole-Genome Bisulfite Sequencing Data Identifies Disease-Associated Changes in DNA Methylation
Source: Genetics. 2017 Feb 16;205(4):1443–58. doi: 10.1534/genetics.116.195008 (PMC5378105; doi:10.1534/genetics.116.195008)

Supplementary Figure 11. Schematic representation of the DMR identified by each method at the *Ifitm3* locus. WGBS of bone marrow derived macrophages from WKY and LEW rats were independently analyzed using ABBA, DSS, BSmooth, MethylKit and MethylSig. For each method we report the location of the DMR identified at 5% FDR level. Only ABBA, DSS and BSmooth identified differential methylation at the *Ifitm3* locus. MethylKit identified a signal for differential methylation which was not genome-wide significant. Amongst the methods that detected significant differential methylation at this locus, only ABBA and DSS identified differential methylation specifically overlapping with *Ifitm3* promoter where the TFBS and differential ChIP-seq signals were also identified (see Figure 3g). In contrast, BSmooth reported a large and rather unspecific region with differential methylation between WKY and LEW macrophages.

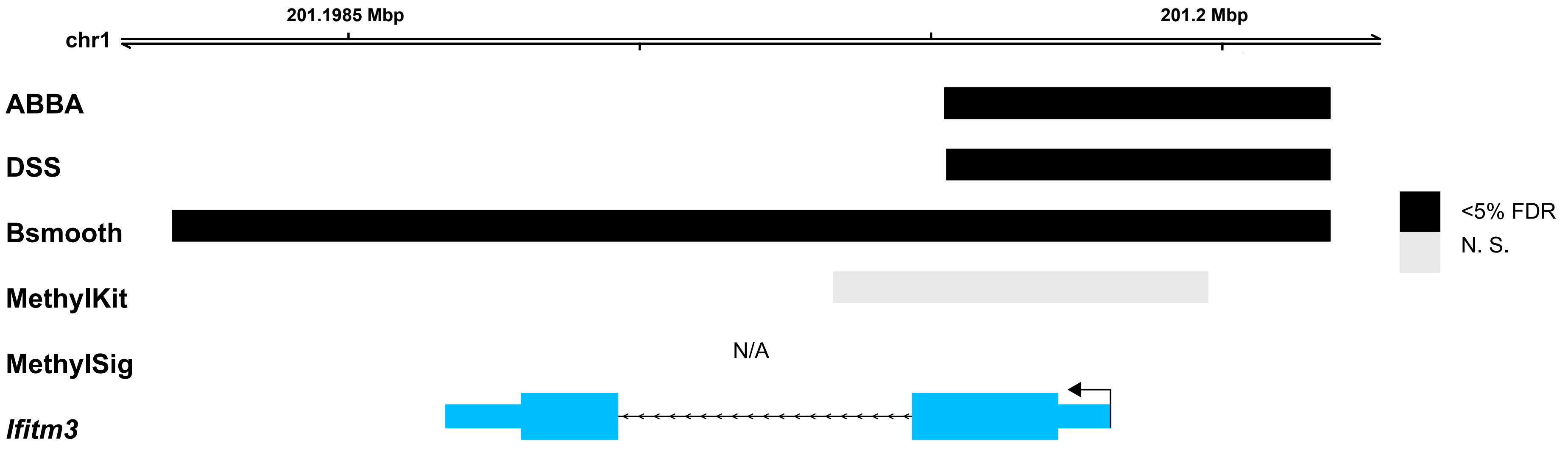

Supplement: Supplementary file 11 [file 1443FigureS11.pdf]
