# Supplementary material for: A Bayesian Approach for Analysis of Whole-Genome Bisulfite Sequencing Data Identifies Disease-Associated Changes in DNA Methylation
Source: Genetics. 2017 Feb 16;205(4):1443–58. doi: 10.1534/genetics.116.195008 (PMC5378105; doi:10.1534/genetics.116.195008)
Supplement: Supplementary file 12 [file 1443TableS1.pdf]

Supplementary Table 1. Posterior distribution of the precision parameters σ_g^{-2} and ρ_g^{-2} under INLA default $(0.1{,}0.1)$ and alternative $(0.01{,}0.01)$ parameterization for selected simulated examples: (a, b) refers to the parameterization of the precision densities while r, λ, s_0, $\Delta meth$ and δ refer to the number of replicates, the average read depth, the level of noise, the methylation probability difference and the level of random perturbation (see Methods for further details about the parameters used in the simulations).

Parameters simulations						Posterior density					
(a,b)	r	λ	s_0	$\Delta meth$	δ	g=2	Mean	Standard deviation	2.5% quantile	50% quantile	97.5% quantile
(0.1,0.1)	1	30	0.2	0.3	0.05	σ_g^{-2}	9.0729	0.6758	7.8368	9.0400	10.4911
						ρ_g^{-2}	1880.7466	100.6382	1689.5549	1878.6136	2084.5644
(0.1,0.1)	2	30	0.2	0.3	0.05	σ_g^{-2}	8.6061	0.4644	7.7401	8.5890	9.5655
						ρ_g^{-2}	1632.7565	78.6897	1481.9587	1631.5982	1790.8550
(0.1,0.1)	3	30	0.2	0.3	0.05	σ_g^{-2}	9.2996	0.5599	8.2602	9.2775	10.4607
						ρ_g^{-2}	1671.8780	82.8469	1514.0137	1670.3027	1839.2332
(0.1,0.1)	1	30	0.2	0.5	0.05	σ_g^{-2}	8.3296	0.6129	7.2066	8.3002	9.6136
						ρ_g^{-2}	1617.9111	85.0787	1456.0105	1616.1979	1790.1218
(0.1,0.1)	2	30	0.2	0.5	0.05	σ_g^{-2}	9.6289	0.5638	8.5684	9.6119	10.7827
						ρ_g^{-2}	1635.3649	77.6139	1487.8252	1633.6680	1792.5946
(0.1,0.1)	3	30	0.2	0.5	0.05	σ_g^{-2}	9.6923	0.5909	8.5810	9.6750	10.9025
						ρ_g^{-2}	1638.4463	78.6457	1489.5000	1636.4900	1798.3660
(0.1,0.1)	1	30	0.2	0.7	0.05	σ_g^{-2}	5.7587	0.3663	5.0799	5.7444	6.5169
						ρ_g^{-2}	1151.5099	58.9534	1039.3727	1150.2486	1270.9486
(0.1,0.1)	2	30	0.2	0.7	0.05	σ_g^{-2}	5.8216	0.2811	5.2898	5.8144	6.3947
						ρ_g^{-2}	1350.8436	65.9308	1225.7229	1349.3751	1484.6081
(0.1,0.1)	3	30	0.2	0.7	0.05	σ_g^{-2}	6.8412	0.3049	6.2625	6.8340	7.4611
						ρ_g^{-2}	1382.2827	62.8940	1262.7061	1380.9600	1509.7034

Parameters simulations						Posterior density					
(a,b)	r	λ	s_0	$\Delta meth$	δ	g=2	Mean	Standard deviation	2.5% quant	50% quant	97.5% quant
(0.01,0.01)	1	30	0.2	0.3	0.05	σ_g^{-2}	8.8469	0.6533	7.6596	8.8112	10.2276
						ρ_g^{-2}	1651.3671	84.8279	1489.6199	1649.9041	1822.5088
(0.01,0.01)	2	30	0.2	0.3	0.05	σ_g^{-2}	10.0552	0.5782	8.9752	10.0348	11.2487
						ρ_g^{-2}	1679.0064	76.4557	1532.5270	1677.8633	1832.4442
(0.01,0.01)	3	30	0.2	0.3	0.05	σ_g^{-2}	10.2526	0.5182	9.2755	10.2378	11.3132
						ρ_g^{-2}	1593.9606	70.6807	1459.0608	1592.7067	1736.4999
(0.01,0.01)	1	30	0.2	0.5	0.05	σ_g^{-2}	8.9345	0.6855	7.7005	8.8926	10.3932
						ρ_g^{-2}	1466.6565	73.4093	1328.4237	1464.4492	1617.0846
(0.01,0.01)	2	30	0.2	0.5	0.05	σ_g^{-2}	9.2826	0.4510	8.4315	9.2700	10.2048
						ρ_g^{-2}	1576.0755	70.7379	1441.3941	1574.7220	1719.0315
(0.01,0.01)	3	30	0.2	0.5	0.05	σ_g^{-2}	8.6662	0.4332	7.8520	8.6525	9.5555
						ρ_g^{-2}	1443.9412	67.0582	1315.8399	1442.7964	1578.9243
(0.01,0.01)	1	30	0.2	0.7	0.05	σ_g^{-2}	6.1100	0.3056	5.5347	6.1007	6.7367
						ρ_g^{-2}	1286.7485	63.4107	1166.3553	1285.3732	1415.2569
(0.01,0.01)	2	30	0.2	0.7	0.05	σ_g^{-2}	6.1109	0.2637	5.6098	6.1051	6.6460
						ρ_g^{-2}	1352.3358	62.3716	1233.9427	1350.9429	1478.9524
(0.01,0.01)	3	30	0.2	0.7	0.05	σ_g^{-2}	7.4169	0.3634	6.7317	7.4065	8.1605
						ρ_g^{-2}	1395.5104	65.6492	1270.7397	1394.1411	1528.4495
