# Supplementary material for: A Bayesian Approach for Analysis of Whole-Genome Bisulfite Sequencing Data Identifies Disease-Associated Changes in DNA Methylation
Source: Genetics. 2017 Feb 16;205(4):1443–58. doi: 10.1534/genetics.116.195008 (PMC5378105; doi:10.1534/genetics.116.195008)
Supplement: Supplementary file 13 [file 1443TableS2.pdf]

Supplementary Table 2. Sensitivity analysis of $\mathrm{E}(\pi_{irg})$, $[\mathrm{V}(\pi_{irg})]^{1/2}$ and $95\%\mathrm{CI}$ of π_{irg} for different values of the noise level ($s_0 = 0.1,0.2,0.3$) and selected values of $\pi_{rg} = 0.1, 0.5$ and 0.99. Values of $\mathrm{E}(\pi_{irg})$, $[\mathrm{V}(\pi_{irg})]^{1/2}$ and $95\%\mathrm{CI}$ of π_{irg} are obtained by stochastic simulation, generating 10^7 observations from the logistic-normal distribution $\mathrm{L}(\pi_{rg}, s_0)$.

	$\boldsymbol{\pi_{rg} = 0.1}$			$\boldsymbol{\pi_{rg} = 0.5}$			$\boldsymbol{\pi_{rg} = 0.99}$		
$\boldsymbol{s_0}$	$\mathrm{E}(\pi_{irg})$	$[\mathrm{V}(\pi_{irg})]^{\frac{1}{2}}$	$95\%\mathrm{CI}$	$\mathrm{E}(\pi_{irg})$	$[\mathrm{V}(\pi_{irg})]^{\frac{1}{2}}$	$95\%\mathrm{CI}$	$\mathrm{E}(\pi_{irg})$	$[\mathrm{V}(\pi_{irg})]^{\frac{1}{2}}$	$95\%\mathrm{CI}$
$\mathbf{0.1}$	0.1036	0.0295	0.0564<br>0.1711	0.5000	0.0775	0.3499<br>0.6501	0.9895	0.0034	0.9816<br>0.9946
$\mathbf{0.2}$	0.1072	0.0436	0.0442<br>0.2107	0.5000	0.1068	0.2937<br>0.7061	0.9890	0.0051	0.9763<br>0.9958
$\mathbf{0.3}$	0.1107	0.0548	0.0366<br>0.2453	0.5000	0.1281	0.2548<br>0.7452	0.9884	0.0067	0.9713<br>0.9966
