# Supplementary material for: A Bayesian Approach for Analysis of Whole-Genome Bisulfite Sequencing Data Identifies Disease-Associated Changes in DNA Methylation
Source: Genetics. 2017 Feb 16;205(4):1443–58. doi: 10.1534/genetics.116.195008 (PMC5378105; doi:10.1534/genetics.116.195008)
Supplement: Supplementary file 14 [file 1443TableS3.pdf]

**Supplementary Table 3**. List of differentially methylated regions (n=427 DMRs) identified at FDR <!
have additional details shown.

| | Differentially methylated regions (DMRs) | | | | |
|---|---|---|---|---|---|
| *Chr.* | *Start position (bp)* | *End position (bp)* | *Number of CpG* | *Delta in methylation (LEW-WKY)* | *Length (bp)* |
| 10 | 68,789,190 | 68,789,566 | 8 | 0.388 | 376 |
| 9 | 65,578,002 | 65,578,018 | 9 | 0.376 | 16 |
| 20 | 7,471,342 | 7,471,507 | 6 | -0.411 | 165 |
| 11 | 85,847,836 | 85,848,164 | 8 | 0.411 | 328 |
| 1 | 201,199,522 | 201,200,184 | 19 | -0.393 | 662 |
| 1 | 135,484,912 | 135,485,255 | 43 | -0.354 | 343 |
| 14 | 84,172,871 | 84,173,390 | 16 | 0.493 | 519 |
| 14 | 84,172,871 | 84,173,390 | 16 | 0.493 | 519 |
| 14 | 84,172,871 | 84,173,390 | 16 | 0.493 | 519 |
| 14 | 84,172,871 | 84,173,390 | 16 | 0.493 | 519 |
| 2 | 244,382,223 | 244,382,456 | 6 | -0.488 | 233 |
| 9 | 8,052,044 | 8,052,235 | 8 | -0.369 | 191 |
| 1 | 159,315,263 | 159,315,432 | 7 | -0.397 | 169 |
| 10 | 91,103,844 | 91,104,178 | 9 | -0.429 | 334 |
| 1 | 213,078,596 | 213,079,072 | 5 | -0.372 | 476 |
| 5 | 76,087,419 | 76,088,118 | 19 | -0.449 | 699 |
| 5 | 76,087,419 | 76,088,118 | 19 | -0.449 | 699 |
| 1 | 249,470,979 | 249,471,171 | 7 | 0.356 | 192 |
| 1 | 47,244,465 | 47,244,525 | 5 | 0.402 | 60 |
| 10 | 68,549,388 | 68,549,672 | 7 | -0.379 | 284 |
| 10 | 68,789,190 | 68,789,566 | 8 | 0.388 | 376 |
| 2 | 244,286,406 | 244,286,792 | 8 | 0.395 | 386 |
| 2 | 244,285,393 | 244,285,537 | 6 | -0.351 | 144 |
| 1 | 246,217,613 | 246,217,908 | 6 | -0.422 | 295 |
| 1 | 246,217,613 | 246,217,908 | 6 | -0.422 | 295 |
| 7 | 117,335,736 | 117,336,128 | 6 | 0.346 | 392 |
| 11 | 85,847,836 | 85,848,164 | 8 | 0.411 | 328 |
| 6 | 7,949,640 | 7,949,973 | 6 | 0.429 | 333 |
| 7 | 135,296,975 | 135,297,088 | 5 | -0.419 | 113 |
| 7 | 135,296,975 | 135,297,088 | 5 | -0.419 | 113 |
| 14 | 84,172,871 | 84,173,390 | 16 | 0.493 | 519 |
| 14 | 84,172,871 | 84,173,390 | 16 | 0.493 | 519 |
| 14 | 92,374,023 | 92,374,031 | 5 | 0.373 | 8 |

| | | | | | |
|---|---|---|---|---|---|
| 18 | 71,444,981 | 71,445,138 | 7 | -0.438 | 157 |
| 18 | 71,444,981 | 71,445,138 | 7 | -0.438 | 157 |
| 18 | 71,444,981 | 71,445,138 | 7 | -0.438 | 157 |
| 5 | 155,578,344 | 155,578,692 | 7 | 0.398 | 348 |
| 5 | 155,446,650 | 155,447,336 | 9 | 0.346 | 686 |
| 5 | 155,447,682 | 155,447,726 | 5 | 0.357 | 44 |
| 4 | 117,447,942 | 117,448,201 | 6 | 0.431 | 259 |
| 4 | 117,447,942 | 117,448,201 | 6 | 0.431 | 259 |
| 4 | 48,334,740 | 48,334,986 | 6 | 0.377 | 246 |
| 4 | 48,334,740 | 48,334,986 | 6 | 0.377 | 246 |
| 4 | 48,334,740 | 48,334,986 | 6 | 0.377 | 246 |
| 8 | 11,391,719 | 11,392,350 | 9 | -0.398 | 631 |
| 8 | 11,391,719 | 11,392,350 | 9 | -0.398 | 631 |
| 2 | 244,382,223 | 244,382,456 | 6 | -0.488 | 233 |
| 9 | 8,052,044 | 8,052,235 | 8 | -0.369 | 191 |
| 1 | 190,990,317 | 190,990,437 | 7 | -0.648 | 120 |
| 13 | 44,601,546 | 44,601,743 | 6 | -0.337 | 197 |
| 13 | 44,601,546 | 44,601,743 | 6 | -0.337 | 197 |
| 5 | 153,336,351 | 153,336,935 | 15 | -0.432 | 584 |
| 5 | 153,345,531 | 153,346,146 | 13 | -0.461 | 615 |
| 5 | 153,345,531 | 153,346,146 | 13 | -0.461 | 615 |
| 5 | 153,336,351 | 153,336,935 | 15 | -0.432 | 584 |
| 8 | 75,999,078 | 75,999,242 | 8 | 0.481 | 164 |
| 8 | 75,999,078 | 75,999,242 | 8 | 0.481 | 164 |
| 1 | 250,085,410 | 250,085,533 | 5 | 0.347 | 123 |
| 9 | 65,578,002 | 65,578,018 | 9 | 0.376 | 16 |
| 9 | 65,578,002 | 65,578,018 | 9 | 0.376 | 16 |
| 9 | 65,578,002 | 65,578,018 | 9 | 0.376 | 16 |
| 10 | 91,103,844 | 91,104,178 | 9 | -0.429 | 334 |
| 2 | 182,060,749 | 182,061,173 | 11 | -0.406 | 424 |
| 2 | 182,060,749 | 182,061,173 | 11 | -0.406 | 424 |
| 6 | 115,280,790 | 115,281,128 | 9 | -0.383 | 338 |
| 5 | 70,555,077 | 70,555,215 | 6 | -0.550 | 138 |
| 5 | 70,555,077 | 70,555,215 | 6 | -0.550 | 138 |
| 5 | 76,087,419 | 76,088,118 | 19 | -0.449 | 699 |
| 1 | 249,470,979 | 249,471,171 | 7 | 0.356 | 192 |
| 1 | 249,470,979 | 249,471,171 | 7 | 0.356 | 192 |
| 1 | 249,470,979 | 249,471,171 | 7 | 0.356 | 192 |

| | | | | | |
|---|---|---|---|---|---|
| 3 | 134,342,611 | 134,342,621 | 6 | 0.426 | 10 |
| 3 | 134,416,266 | 134,416,684 | 9 | 0.400 | 418 |
| 4 | 157,369,967 | 157,369,979 | 7 | 0.382 | 12 |
| 5 | 154,761,710 | 154,762,122 | 14 | 0.363 | 412 |
| 15 | 9,324,042 | 9,324,638 | 8 | -0.392 | 596 |
| 15 | 9,324,042 | 9,324,638 | 8 | -0.392 | 596 |
| 11 | 74,423,528 | 74,423,536 | 5 | 0.352 | 8 |
| 1 | 196,783,603 | 196,784,043 | 11 | 0.379 | 440 |
| 1 | 196,783,603 | 196,784,043 | 11 | 0.379 | 440 |
| 16 | 7,121,594 | 7,121,762 | 6 | -0.490 | 168 |
| 20 | 13,130,833 | 13,131,181 | 5 | -0.337 | 348 |
| 20 | 7,471,342 | 7,471,507 | 6 | -0.411 | 165 |
| 20 | 7,471,342 | 7,471,507 | 6 | -0.411 | 165 |
| 13 | 48,283,546 | 48,283,871 | 5 | 0.436 | 325 |
| 13 | 48,283,546 | 48,283,871 | 5 | 0.436 | 325 |
| 11 | 85,847,836 | 85,848,164 | 8 | 0.411 | 328 |
| 11 | 85,847,836 | 85,848,164 | 8 | 0.411 | 328 |
| 11 | 85,847,836 | 85,848,164 | 8 | 0.411 | 328 |
| 1 | 201,199,522 | 201,200,184 | 19 | -0.393 | 662 |
| 5 | 160,519,056 | 160,519,506 | 8 | -0.397 | 450 |
| 1 | 186,478,917 | 186,479,089 | 6 | -0.335 | 172 |
| 2 | 244,285,393 | 244,285,537 | 6 | -0.351 | 144 |
| 10 | 91,103,844 | 91,104,178 | 9 | -0.429 | 334 |
| 5 | 76,203,959 | 76,203,980 | 5 | 0.335 | 21 |
| 1 | 159,315,263 | 159,315,432 | 7 | -0.397 | 169 |
| 1 | 213,078,596 | 213,079,072 | 5 | -0.372 | 476 |
| 11 | 85,847,836 | 85,848,164 | 8 | 0.411 | 328 |
| 1 | 201,199,522 | 201,200,184 | 19 | -0.393 | 662 |
| 10 | 91,103,844 | 91,104,178 | 9 | -0.429 | 334 |
| 10 | 45,286,705 | 45,286,953 | 6 | -0.338 | 248 |
| 10 | 45,286,705 | 45,286,953 | 6 | -0.338 | 248 |
| 10 | 45,286,705 | 45,286,953 | 6 | -0.338 | 248 |
| 10 | 45,286,705 | 45,286,953 | 6 | -0.338 | 248 |
| 10 | 45,286,705 | 45,286,953 | 6 | -0.338 | 248 |
| 10 | 45,286,705 | 45,286,953 | 6 | -0.338 | 248 |
| 8 | 85,309,118 | 85,309,328 | 5 | 0.417 | 210 |
| 8 | 85,309,118 | 85,309,328 | 5 | 0.417 | 210 |
| 8 | 126,281,337 | 126,281,606 | 6 | 0.360 | 269 |

| | | | | | |
|---|---|---|---|---|---|
| 8 | 126,281,337 | 126,281,606 | 6 | 0.360 | 269 |
| 8 | 126,281,337 | 126,281,606 | 6 | 0.360 | 269 |
| 8 | 126,281,337 | 126,281,606 | 6 | 0.360 | 269 |
| 9 | 86,786,809 | 86,786,843 | 5 | -0.439 | 34 |
| 2 | 22,695,411 | 22,696,273 | 19 | 0.372 | 862 |
| 7 | 80,606,707 | 80,606,758 | 7 | -0.406 | 51 |
| 19 | 50,424,154 | 50,424,775 | 12 | -0.347 | 621 |
| 3 | 33,805,390 | 33,805,520 | 6 | 0.354 | 130 |
| 8 | 59,704,529 | 59,704,768 | 5 | -0.553 | 239 |
| X | 12,543,997 | 12,544,127 | 9 | -0.359 | 130 |
| X | 12,543,997 | 12,544,127 | 9 | -0.359 | 130 |
| 7 | 31,729,909 | 31,729,925 | 5 | 0.631 | 16 |
| 13 | 66,828,295 | 66,828,461 | 8 | 0.354 | 166 |
| 13 | 66,828,295 | 66,828,461 | 8 | 0.354 | 166 |
| 3 | 134,626,281 | 134,626,603 | 6 | -0.472 | 322 |
| 3 | 134,626,281 | 134,626,603 | 6 | -0.472 | 322 |
| 3 | 134,626,281 | 134,626,603 | 6 | -0.472 | 322 |
| 1 | 253,035,706 | 253,035,897 | 8 | -0.356 | 191 |
| 1 | 253,035,706 | 253,035,897 | 8 | -0.356 | 191 |
| 8 | 113,765,414 | 113,765,749 | 5 | 0.449 | 335 |
| 16 | 82,838,635 | 82,838,679 | 5 | 0.460 | 44 |
| 16 | 82,838,635 | 82,838,679 | 5 | 0.460 | 44 |
| 1 | 41,153,148 | 41,153,356 | 5 | 0.389 | 208 |
| 7 | 140,097,766 | 140,098,056 | 16 | -0.381 | 290 |
| 7 | 140,097,766 | 140,098,056 | 16 | -0.381 | 290 |
| 7 | 140,097,766 | 140,098,056 | 16 | -0.381 | 290 |
| 7 | 140,097,766 | 140,098,056 | 16 | -0.381 | 290 |
| 11 | 68,731,193 | 68,731,412 | 5 | 0.412 | 219 |
| 11 | 68,731,193 | 68,731,412 | 5 | 0.412 | 219 |
| 11 | 68,731,193 | 68,731,412 | 5 | 0.412 | 219 |
| 4 | 87,163,138 | 87,163,811 | 7 | -0.387 | 673 |
| 4 | 87,163,138 | 87,163,811 | 7 | -0.387 | 673 |
| 2 | 145,004,434 | 145,005,279 | 9 | -0.501 | 845 |
| 2 | 145,004,434 | 145,005,279 | 9 | -0.501 | 845 |
| 4 | 184,204,699 | 184,204,723 | 5 | 0.362 | 24 |
| 1 | 171,743,067 | 171,743,305 | 5 | -0.556 | 238 |
| 1 | 171,993,800 | 171,994,086 | 13 | 0.679 | 286 |
| 14 | 81,650,728 | 81,650,744 | 6 | 0.415 | 16 |

| | | | | | |
|---|---|---|---|---|---|
| 14 | 81,650,728 | 81,650,744 | 6 | 0.415 | 16 |
| 18 | 78,990,391 | 78,991,032 | 8 | 0.404 | 641 |
| 18 | 78,990,391 | 78,991,032 | 8 | 0.404 | 641 |
| 1 | 8,656,997 | 8,657,837 | 9 | 0.348 | 840 |
| 1 | 8,658,157 | 8,658,360 | 7 | 0.385 | 203 |
| 1 | 8,687,740 | 8,687,867 | 5 | -0.360 | 127 |
| 1 | 135,484,912 | 135,485,255 | 43 | -0.354 | 343 |
| 1 | 135,484,912 | 135,485,255 | 43 | -0.354 | 343 |
| 1 | 135,484,912 | 135,485,255 | 43 | -0.354 | 343 |
| X | 21,065,942 | 21,065,954 | 7 | 0.366 | 12 |
| X | 21,065,942 | 21,065,954 | 7 | 0.366 | 12 |
| X | 21,065,942 | 21,065,954 | 7 | 0.366 | 12 |
| X | 21,065,942 | 21,065,954 | 7 | 0.366 | 12 |
| X | 21,065,942 | 21,065,954 | 7 | 0.366 | 12 |
| X | 21,065,942 | 21,065,954 | 7 | 0.366 | 12 |
| 8 | 51,013,364 | 51,014,520 | 26 | -0.363 | 1156 |
| 4 | 78,570,503 | 78,570,515 | 6 | -0.361 | 12 |
| 4 | 78,570,503 | 78,570,515 | 6 | -0.361 | 12 |
| 4 | 78,570,503 | 78,570,515 | 6 | -0.361 | 12 |
| 1 | 201,692,359 | 201,692,898 | 6 | -0.431 | 539 |
| 1 | 201,692,359 | 201,692,898 | 6 | -0.431 | 539 |
| 1 | 201,692,359 | 201,692,898 | 6 | -0.431 | 539 |
| 7 | 127,373,180 | 127,373,588 | 10 | 0.343 | 408 |
| 7 | 127,373,180 | 127,373,588 | 10 | 0.343 | 408 |
| 7 | 127,373,180 | 127,373,588 | 10 | 0.343 | 408 |
| 7 | 127,373,180 | 127,373,588 | 10 | 0.343 | 408 |
| 7 | 127,373,180 | 127,373,588 | 10 | 0.343 | 408 |
| 7 | 127,373,180 | 127,373,588 | 10 | 0.343 | 408 |
| 7 | 127,373,180 | 127,373,588 | 10 | 0.343 | 408 |
| 7 | 127,373,180 | 127,373,588 | 10 | 0.343 | 408 |
| 1 | 260,004,778 | 260,005,055 | 5 | 0.433 | 277 |
| 1 | 260,004,778 | 260,005,055 | 5 | 0.433 | 277 |
| 1 | 260,004,778 | 260,005,055 | 5 | 0.433 | 277 |
| 1 | 13,385,349 | 13,385,499 | 8 | -0.393 | 150 |
| 1 | 13,482,975 | 13,483,125 | 7 | -0.389 | 150 |
| 6 | 99,479,034 | 99,479,581 | 17 | 0.353 | 547 |
| 12 | 35,455,412 | 35,455,802 | 6 | -0.440 | 390 |
| 19 | 57,252,546 | 57,252,576 | 5 | -0.351 | 30 |

| | | | | | |
|---|---|---|---|---|---|
| 19 | 57,252,546 | 57,252,576 | 5 | -0.351 | 30 |
| 19 | 57,252,546 | 57,252,576 | 5 | -0.351 | 30 |
| 19 | 57,252,546 | 57,252,576 | 5 | -0.351 | 30 |
| 5 | 62,025,389 | 62,025,405 | 6 | -0.421 | 16 |
| 1 | 220,502,532 | 220,502,663 | 5 | 0.350 | 131 |
| 1 | 220,502,532 | 220,502,663 | 5 | 0.350 | 131 |
| 14 | 44,839,477 | 44,839,645 | 6 | -0.433 | 168 |
| 7 | 32,705,794 | 32,706,239 | 6 | -0.364 | 445 |
| 16 | 82,879,993 | 82,880,104 | 5 | 0.422 | 111 |
| 11 | 68,600,018 | 68,600,266 | 8 | 0.430 | 248 |
| 11 | 68,600,018 | 68,600,266 | 8 | 0.430 | 248 |
| 11 | 68,600,018 | 68,600,266 | 8 | 0.430 | 248 |
| 11 | 68,600,018 | 68,600,266 | 8 | 0.430 | 248 |
| 3 | 10,211,987 | 10,212,102 | 6 | 0.379 | 115 |
| 8 | 77,853,419 | 77,853,528 | 5 | 0.609 | 109 |
| 9 | 86,654,956 | 86,654,984 | 7 | -0.648 | 28 |
| 9 | 86,654,956 | 86,654,984 | 7 | -0.648 | 28 |
| 6 | 23,978,590 | 23,978,604 | 8 | 0.346 | 14 |
| 16 | 47,600,289 | 47,600,899 | 9 | 0.362 | 610 |
| 16 | 47,600,289 | 47,600,899 | 9 | 0.362 | 610 |
| 16 | 47,600,289 | 47,600,899 | 9 | 0.362 | 610 |
| 19 | 49,647,060 | 49,647,070 | 6 | 0.579 | 10 |
| 2 | 145,004,434 | 145,005,279 | 9 | -0.501 | 845 |
| 15 | 3,439,316 | 3,439,583 | 9 | -0.349 | 267 |
| 15 | 3,418,049 | 3,418,278 | 6 | 0.511 | 229 |
| 3 | 77,663,111 | 77,663,297 | 6 | -0.350 | 186 |
| 2 | 235,373,912 | 235,374,163 | 7 | 0.345 | 251 |
| 20 | 9,687,919 | 9,688,761 | 13 | -0.346 | 842 |
| 1 | 168,052,289 | 168,052,458 | 6 | -0.447 | 169 |
| 12 | 12,549,437 | 12,549,914 | 14 | -0.446 | 477 |
| 10 | 109,943,346 | 109,943,368 | 10 | 0.493 | 22 |
| 1 | 226,659,436 | 226,659,448 | 7 | 0.391 | 12 |
| 1 | 135,046,891 | 135,047,432 | 13 | -0.363 | 541 |
| 2 | 1,577,669 | 1,578,072 | 17 | 0.522 | 403 |
| 2 | 1,577,669 | 1,578,072 | 17 | 0.522 | 403 |
| 2 | 1,577,669 | 1,578,072 | 17 | 0.522 | 403 |
| 2 | 1,577,669 | 1,578,072 | 17 | 0.522 | 403 |
| 12 | 6,607,459 | 6,607,658 | 6 | -0.334 | 199 |

| | | | | | |
|---|---|---|---|---|---|
| 3 | 24,798,384 | 24,798,968 | 6 | 0.354 | 584 |
| 15 | 53,811,079 | 53,811,208 | 7 | 0.385 | 129 |
| 8 | 114,018,335 | 114,018,391 | 5 | 0.375 | 56 |
| 8 | 114,018,335 | 114,018,391 | 5 | 0.375 | 56 |
| 8 | 114,018,335 | 114,018,391 | 5 | 0.375 | 56 |
| 6 | 109,561,731 | 109,561,820 | 5 | -0.339 | 89 |
| 6 | 109,561,731 | 109,561,820 | 5 | -0.339 | 89 |
| 1 | 248,664,997 | 248,665,070 | 7 | -0.439 | 73 |
| 1 | 248,664,997 | 248,665,070 | 7 | -0.439 | 73 |
| 2 | 22,695,411 | 22,696,273 | 19 | 0.372 | 862 |
| 3 | 2,646,042 | 2,646,338 | 8 | 0.354 | 296 |
| 5 | 57,621,245 | 57,621,429 | 5 | 0.372 | 184 |
| 5 | 57,621,245 | 57,621,429 | 5 | 0.372 | 184 |
| 5 | 57,621,245 | 57,621,429 | 5 | 0.372 | 184 |
| 5 | 57,621,245 | 57,621,429 | 5 | 0.372 | 184 |
| 17 | 64,161,533 | 64,161,805 | 7 | 0.347 | 272 |
| 17 | 64,161,533 | 64,161,805 | 7 | 0.347 | 272 |
| 5 | 128,444,856 | 128,444,864 | 5 | -0.453 | 8 |
| 5 | 128,444,856 | 128,444,864 | 5 | -0.453 | 8 |
| 5 | 128,444,856 | 128,444,864 | 5 | -0.453 | 8 |
| 4 | 174,510,556 | 174,510,687 | 6 | 0.422 | 131 |
| 4 | 174,377,854 | 174,377,909 | 8 | 0.553 | 55 |
| 7 | 138,463,613 | 138,463,755 | 7 | -0.415 | 142 |
| 7 | 138,463,613 | 138,463,755 | 7 | -0.415 | 142 |
| 12 | 1,553,097 | 1,553,639 | 7 | 0.336 | 542 |
| 12 | 40,645,872 | 40,646,855 | 13 | 0.451 | 983 |
| 9 | 104,717,293 | 104,717,671 | 8 | -0.410 | 378 |
| 3 | 117,132,673 | 117,132,776 | 7 | -0.559 | 103 |
| 2 | 35,616,505 | 35,616,811 | 5 | -0.445 | 306 |
| 16 | 19,179,611 | 19,179,700 | 5 | 0.396 | 89 |
| 16 | 19,179,611 | 19,179,700 | 5 | 0.396 | 89 |
| 16 | 19,179,611 | 19,179,700 | 5 | 0.396 | 89 |
| 12 | 9,798,096 | 9,798,528 | 5 | 0.382 | 432 |
| 20 | 11,657,538 | 11,657,779 | 5 | 0.341 | 241 |
| 5 | 159,782,313 | 159,782,474 | 5 | 0.421 | 161 |
| 6 | 123,615,494 | 123,615,931 | 5 | 0.344 | 437 |
| 2 | 140,674,350 | 140,674,943 | 6 | 0.451 | 593 |
| 2 | 140,674,350 | 140,674,943 | 6 | 0.451 | 593 |

| | | | | | |
|---|---|---|---|---|---|
| X | 80,860,449 | 80,860,804 | 5 | 0.549 | 355 |
| 14 | 80,780,951 | 80,781,311 | 7 | -0.376 | 360 |
| 14 | 80,780,951 | 80,781,311 | 7 | -0.376 | 360 |
| 14 | 80,780,951 | 80,781,311 | 7 | -0.376 | 360 |
| 12 | 34,936,105 | 34,936,445 | 12 | -0.392 | 340 |
| 12 | 34,936,105 | 34,936,445 | 12 | -0.392 | 340 |
| 12 | 34,936,105 | 34,936,445 | 12 | -0.392 | 340 |
| 12 | 34,936,105 | 34,936,445 | 12 | -0.392 | 340 |
| 12 | 34,936,105 | 34,936,445 | 12 | -0.392 | 340 |
| 16 | 81,097,893 | 81,097,901 | 5 | 0.428 | 8 |
| 1 | 78,484,675 | 78,484,717 | 11 | 0.516 | 42 |
| 4 | 124,660,465 | 124,660,479 | 7 | 0.479 | 14 |
| 3 | 146,662,303 | 146,662,850 | 6 | 0.348 | 547 |
| 3 | 146,662,303 | 146,662,850 | 6 | 0.348 | 547 |
| 10 | 78,981,800 | 78,981,895 | 5 | -0.492 | 95 |
| 13 | 80,183,729 | 80,184,045 | 7 | 0.398 | 316 |
| 8 | 123,565,017 | 123,565,083 | 5 | 0.411 | 66 |
| 8 | 51,883,825 | 51,883,881 | 5 | 0.386 | 56 |
| 10 | 65,245,219 | 65,245,537 | 8 | 0.368 | 318 |
| 10 | 65,245,219 | 65,245,537 | 8 | 0.368 | 318 |
| 10 | 65,245,219 | 65,245,537 | 8 | 0.368 | 318 |
| 5 | 77,941,785 | 77,941,939 | 6 | 0.354 | 154 |
| 5 | 77,935,978 | 77,936,652 | 9 | 0.484 | 674 |
| 5 | 77,941,785 | 77,941,939 | 6 | 0.354 | 154 |
| 5 | 77,935,978 | 77,936,652 | 9 | 0.484 | 674 |
| 18 | 72,578,834 | 72,578,888 | 9 | -0.350 | 54 |
| X | 12,543,997 | 12,544,127 | 9 | -0.359 | 130 |
| X | 12,543,997 | 12,544,127 | 9 | -0.359 | 130 |
| 10 | 65,609,330 | 65,609,916 | 6 | -0.381 | 586 |
| 10 | 65,609,330 | 65,609,916 | 6 | -0.381 | 586 |
| 10 | 65,609,330 | 65,609,916 | 6 | -0.381 | 586 |
| 10 | 65,609,330 | 65,609,916 | 6 | -0.381 | 586 |
| 1 | 189,786,754 | 189,786,764 | 6 | 0.477 | 10 |
| 1 | 189,786,754 | 189,786,764 | 6 | 0.477 | 10 |
| 1 | 189,786,754 | 189,786,764 | 6 | 0.477 | 10 |
| 1 | 189,786,754 | 189,786,764 | 6 | 0.477 | 10 |
| 10 | 90,071,670 | 90,071,742 | 5 | 0.375 | 72 |
| 10 | 90,071,670 | 90,071,742 | 5 | 0.375 | 72 |

| | | | | | |
|---|---|---|---|---|---|
| 10 | 90,071,670 | 90,071,742 | 5 | 0.375 | 72 |
| 10 | 90,071,670 | 90,071,742 | 5 | 0.375 | 72 |
| 5 | 152,236,112 | 152,236,164 | 7 | 0.349 | 52 |
| 5 | 152,236,112 | 152,236,164 | 7 | 0.349 | 52 |
| 5 | 152,236,112 | 152,236,164 | 7 | 0.349 | 52 |
| 8 | 59,855,879 | 59,856,223 | 15 | 0.440 | 344 |
| 10 | 98,799,254 | 98,799,270 | 8 | 0.380 | 16 |
| 15 | 59,738,222 | 59,738,294 | 7 | -0.404 | 72 |
| 15 | 59,738,222 | 59,738,294 | 7 | -0.404 | 72 |
| 2 | 38,088,240 | 38,088,351 | 5 | -0.385 | 111 |
| 2 | 38,088,240 | 38,088,351 | 5 | -0.385 | 111 |
| 2 | 181,737,034 | 181,737,215 | 8 | 0.661 | 181 |
| 13 | 86,648,810 | 86,649,168 | 10 | 0.527 | 358 |
| 9 | 11,843,013 | 11,843,287 | 5 | 0.357 | 274 |
| 9 | 11,843,013 | 11,843,287 | 5 | 0.357 | 274 |
| 3 | 18,998,300 | 18,998,479 | 6 | -0.407 | 179 |
| 18 | 72,117,718 | 72,117,864 | 7 | -0.374 | 146 |
| 18 | 72,117,718 | 72,117,864 | 7 | -0.374 | 146 |
| 17 | 10,919,087 | 10,919,658 | 8 | -0.479 | 571 |
| 9 | 73,664,086 | 73,664,354 | 6 | 0.430 | 268 |
| 9 | 73,664,086 | 73,664,354 | 6 | 0.430 | 268 |
| 9 | 73,664,086 | 73,664,354 | 6 | 0.430 | 268 |
| 1 | 85,260,131 | 85,260,301 | 10 | -0.356 | 170 |
| 1 | 85,260,131 | 85,260,301 | 10 | -0.356 | 170 |
| 5 | 130,486,383 | 130,486,577 | 5 | -0.344 | 194 |
| 5 | 130,486,383 | 130,486,577 | 5 | -0.344 | 194 |
| 5 | 130,486,383 | 130,486,577 | 5 | -0.344 | 194 |
| 4 | 131,861,600 | 131,861,612 | 7 | 0.542 | 12 |
| 5 | 157,136,872 | 157,137,311 | 5 | 0.366 | 439 |
| 14 | 5,382,213 | 5,382,382 | 7 | -0.472 | 169 |
| 3 | 106,195,835 | 106,195,926 | 11 | -0.347 | 91 |
| 3 | 106,195,835 | 106,195,926 | 11 | -0.347 | 91 |
| 3 | 106,195,835 | 106,195,926 | 11 | -0.347 | 91 |
| 9 | 38,628,169 | 38,628,423 | 5 | 0.467 | 254 |
| 13 | 91,565,229 | 91,565,370 | 18 | 0.378 | 141 |
| 13 | 91,565,229 | 91,565,370 | 18 | 0.378 | 141 |
| 1 | 78,860,567 | 78,860,597 | 7 | -0.359 | 30 |
| 9 | 63,532,978 | 63,533,535 | 6 | 0.352 | 557 |

| | | | | | |
|---|---|---|---|---|---|
| 9 | 63,532,978 | 63,533,535 | 6 | 0.352 | 557 |
| 2 | 178,059,485 | 178,060,000 | 14 | -0.539 | 515 |
| 12 | 43,695,393 | 43,695,467 | 8 | -0.448 | 74 |
| 12 | 43,695,393 | 43,695,467 | 8 | -0.448 | 74 |
| 7 | 137,954,632 | 137,954,786 | 6 | -0.493 | 154 |
| 7 | 137,954,632 | 137,954,786 | 6 | -0.493 | 154 |
| 2 | 34,066,465 | 34,066,636 | 5 | 0.601 | 171 |
| 1 | 135,132,836 | 135,133,153 | 7 | 0.350 | 317 |
| 1 | 233,041,339 | 233,041,381 | 6 | 0.339 | 42 |
| 3 | 106,583,026 | 106,583,192 | 5 | 0.397 | 166 |
| 3 | 106,583,026 | 106,583,192 | 5 | 0.397 | 166 |
| 3 | 106,583,026 | 106,583,192 | 5 | 0.397 | 166 |
| 3 | 106,583,026 | 106,583,192 | 5 | 0.397 | 166 |
| 15 | 12,704,935 | 12,705,277 | 17 | -0.369 | 342 |
| 14 | 6,269,076 | 6,269,525 | 5 | 0.344 | 449 |
| 14 | 6,269,076 | 6,269,525 | 5 | 0.344 | 449 |
| 9 | 6,207,562 | 6,208,474 | 12 | 0.440 | 912 |
| 2 | 180,479,749 | 180,479,914 | 11 | 0.346 | 165 |
| 2 | 180,479,749 | 180,479,914 | 11 | 0.346 | 165 |
| 2 | 180,479,749 | 180,479,914 | 11 | 0.346 | 165 |
| 10 | 109,175,668 | 109,175,915 | 5 | 0.365 | 247 |
| 18 | 66,163,003 | 66,163,230 | 6 | -0.549 | 227 |
| 18 | 66,163,003 | 66,163,230 | 6 | -0.549 | 227 |
| 17 | 20,918,246 | 20,918,432 | 5 | -0.340 | 186 |
| 17 | 20,918,246 | 20,918,432 | 5 | -0.340 | 186 |
| 4 | 171,094,497 | 171,094,719 | 8 | -0.408 | 222 |
| 4 | 171,094,497 | 171,094,719 | 8 | -0.408 | 222 |
| 3 | 146,234,643 | 146,234,787 | 5 | 0.371 | 144 |
| 3 | 156,326,796 | 156,327,497 | 13 | 0.406 | 701 |
| 3 | 156,326,796 | 156,327,497 | 13 | 0.406 | 701 |
| 3 | 156,326,796 | 156,327,497 | 13 | 0.406 | 701 |
| 3 | 156,326,796 | 156,327,497 | 13 | 0.406 | 701 |
| 3 | 156,326,796 | 156,327,497 | 13 | 0.406 | 701 |
| 3 | 156,326,796 | 156,327,497 | 13 | 0.406 | 701 |
| 2 | 61,695,657 | 61,696,249 | 6 | 0.446 | 592 |
| 2 | 61,695,657 | 61,696,249 | 6 | 0.446 | 592 |
| 7 | 131,754,601 | 131,754,725 | 6 | 0.351 | 124 |
| 7 | 131,754,601 | 131,754,725 | 6 | 0.351 | 124 |

| | | | | | |
|---|---|---|---|---|---|
| 7 | 131,754,601 | 131,754,725 | 6 | 0.351 | 124 |
| 14 | 13,939,390 | 13,939,747 | 11 | -0.480 | 357 |
| 14 | 13,939,390 | 13,939,747 | 11 | -0.480 | 357 |
| 3 | 106,195,835 | 106,195,926 | 11 | -0.347 | 91 |
| 8 | 119,646,817 | 119,647,031 | 6 | 0.349 | 214 |
| 8 | 119,646,817 | 119,647,031 | 6 | 0.349 | 214 |
| 12 | 27,416,189 | 27,416,464 | 5 | -0.381 | 275 |
| 8 | 115,452,205 | 115,452,697 | 11 | -0.374 | 492 |
| 20 | 28,107,316 | 28,107,691 | 5 | 0.435 | 375 |
| 20 | 28,107,316 | 28,107,691 | 5 | 0.435 | 375 |
| 20 | 28,107,316 | 28,107,691 | 5 | 0.435 | 375 |
| 20 | 28,107,316 | 28,107,691 | 5 | 0.435 | 375 |
| 2 | 44,238,738 | 44,239,261 | 14 | -0.579 | 523 |
| 2 | 44,238,738 | 44,239,261 | 14 | -0.579 | 523 |
| 2 | 44,238,738 | 44,239,261 | 14 | -0.579 | 523 |
| 12 | 15,707,093 | 15,707,797 | 14 | -0.454 | 704 |
| 12 | 15,764,023 | 15,764,382 | 11 | 0.525 | 359 |
| 12 | 15,790,165 | 15,790,299 | 5 | -0.387 | 134 |
| 12 | 15,790,165 | 15,790,299 | 5 | -0.387 | 134 |
| 20 | 13,999,066 | 13,999,393 | 9 | 0.447 | 327 |
| 20 | 13,999,066 | 13,999,393 | 9 | 0.447 | 327 |
| 20 | 13,615,192 | 13,615,788 | 12 | -0.394 | 596 |
| 20 | 13,615,192 | 13,615,788 | 12 | -0.394 | 596 |
| 16 | 79,495,038 | 79,495,122 | 6 | -0.442 | 84 |
| 15 | 24,229,425 | 24,229,701 | 11 | 0.378 | 276 |
| 15 | 24,355,228 | 24,355,439 | 9 | 0.362 | 211 |
| 9 | 102,117,225 | 102,117,401 | 5 | 0.349 | 176 |
| 16 | 75,913,439 | 75,913,779 | 7 | -0.485 | 340 |
| 16 | 75,912,740 | 75,912,945 | 5 | -0.406 | 205 |
| 3 | 74,824,354 | 74,825,183 | 20 | -0.369 | 829 |
| 3 | 74,828,873 | 74,829,413 | 7 | -0.359 | 540 |
| 3 | 74,824,354 | 74,825,183 | 20 | -0.369 | 829 |
| 3 | 74,828,873 | 74,829,413 | 7 | -0.359 | 540 |
| 3 | 74,824,354 | 74,825,183 | 20 | -0.369 | 829 |
| 3 | 74,824,354 | 74,825,183 | 20 | -0.369 | 829 |
| 3 | 74,824,354 | 74,825,183 | 20 | -0.369 | 829 |
| 3 | 74,824,354 | 74,825,183 | 20 | -0.369 | 829 |
| 1 | 84,804,987 | 84,805,205 | 20 | -0.336 | 218 |

| | | | | | |
|---|---|---|---|---|---|
| 1 | 26,632,252 | 26,632,340 | 7 | 0.374 | 88 |
| 6 | 110,369,199 | 110,369,777 | 6 | -0.423 | 578 |
| 6 | 110,369,199 | 110,369,777 | 6 | -0.423 | 578 |
| 16 | 81,401,972 | 81,402,211 | 6 | 0.339 | 239 |
| 16 | 81,401,972 | 81,402,211 | 6 | 0.339 | 239 |
| 16 | 81,401,972 | 81,402,211 | 6 | 0.339 | 239 |
| 16 | 81,401,972 | 81,402,211 | 6 | 0.339 | 239 |
| 4 | 9,571,948 | 9,572,255 | 8 | 0.525 | 307 |
| 4 | 9,571,948 | 9,572,255 | 8 | 0.525 | 307 |
| 2 | 9,683,275 | 9,683,540 | 5 | -0.388 | 265 |
| 2 | 9,683,275 | 9,683,540 | 5 | -0.388 | 265 |
| 5 | 169,268,017 | 169,268,674 | 15 | -0.405 | 657 |
| 8 | 32,210,435 | 32,210,821 | 6 | -0.446 | 386 |
| 8 | 32,210,435 | 32,210,821 | 6 | -0.446 | 386 |
| 1 | 15,755,426 | 15,755,560 | 5 | -0.524 | 134 |
| 1 | 15,755,426 | 15,755,560 | 5 | -0.524 | 134 |
| 4 | 10,024,644 | 10,024,770 | 5 | -0.468 | 126 |
| 4 | 10,024,644 | 10,024,770 | 5 | -0.468 | 126 |
| 4 | 10,024,644 | 10,024,770 | 5 | -0.468 | 126 |
| 4 | 10,024,644 | 10,024,770 | 5 | -0.468 | 126 |
| 4 | 165,692,804 | 165,694,575 | 31 | -0.490 | 1771 |
| 16 | 54,381,850 | 54,381,868 | 10 | 0.436 | 18 |
| 16 | 54,381,850 | 54,381,868 | 10 | 0.436 | 18 |
| 10 | 82,763,325 | 82,763,338 | 6 | -0.344 | 13 |
| 10 | 82,763,325 | 82,763,338 | 6 | -0.344 | 13 |
| 10 | 82,763,325 | 82,763,338 | 6 | -0.344 | 13 |
| 10 | 82,763,325 | 82,763,338 | 6 | -0.344 | 13 |
| 10 | 82,763,325 | 82,763,338 | 6 | -0.344 | 13 |
| 10 | 82,763,325 | 82,763,338 | 6 | -0.344 | 13 |
| 10 | 82,763,325 | 82,763,338 | 6 | -0.344 | 13 |
| 19 | 52,196,706 | 52,197,193 | 6 | 0.351 | 487 |
| 19 | 52,196,706 | 52,197,193 | 6 | 0.351 | 487 |
| 19 | 52,196,706 | 52,197,193 | 6 | 0.351 | 487 |
| 12 | 15,764,023 | 15,764,382 | 11 | 0.525 | 359 |
| 18 | 3,768,667 | 3,769,240 | 9 | 0.482 | 573 |
| 4 | 125,998,194 | 125,998,479 | 5 | 0.344 | 285 |
| 4 | 125,998,194 | 125,998,479 | 5 | 0.344 | 285 |
| 4 | 125,998,194 | 125,998,479 | 5 | 0.344 | 285 |

| | | | | | |
|---|---|---|---|---|---|
| 2 | 28,673,047 | 28,673,172 | 5 | 0.339 | 125 |
| 2 | 28,673,047 | 28,673,172 | 5 | 0.339 | 125 |
| 19 | 14,329,022 | 14,329,481 | 6 | -0.545 | 459 |
| 16 | 17,385,924 | 17,386,108 | 5 | 0.445 | 184 |
| 15 | 85,308,726 | 85,308,883 | 6 | -0.364 | 157 |
| 10 | 105,374,123 | 105,374,195 | 8 | -0.346 | 72 |
| 10 | 105,374,123 | 105,374,195 | 8 | -0.346 | 72 |
| 4 | 119,369,777 | 119,369,982 | 14 | -0.396 | 205 |
| 12 | 39,448,249 | 39,448,449 | 6 | -0.452 | 200 |
| 1 | 149,750,872 | 149,750,993 | 9 | -0.458 | 121 |
| 14 | 6,356,207 | 6,356,495 | 11 | -0.362 | 288 |
| 14 | 6,356,207 | 6,356,495 | 11 | -0.362 | 288 |
| 14 | 6,356,207 | 6,356,495 | 11 | -0.362 | 288 |
| 14 | 6,356,615 | 6,356,750 | 8 | -0.426 | 135 |
| 2 | 196,341,870 | 196,341,884 | 8 | -0.400 | 14 |
| 2 | 196,341,870 | 196,341,884 | 8 | -0.400 | 14 |
| 14 | 17,244,290 | 17,244,481 | 6 | 0.497 | 191 |
| 1 | 85,488,421 | 85,488,435 | 8 | 0.492 | 14 |
| 3 | 11,927,412 | 11,927,814 | 13 | 0.411 | 402 |
| 3 | 11,926,832 | 11,927,335 | 9 | 0.402 | 503 |
| 3 | 11,926,832 | 11,927,335 | 9 | 0.402 | 503 |
| 3 | 11,927,412 | 11,927,814 | 13 | 0.411 | 402 |
| 13 | 86,843,953 | 86,844,345 | 14 | 0.385 | 392 |
| 5 | 167,653,998 | 167,654,703 | 10 | 0.338 | 705 |
| 5 | 167,653,998 | 167,654,703 | 10 | 0.338 | 705 |
| 5 | 167,653,998 | 167,654,703 | 10 | 0.338 | 705 |
| 4 | 152,223,076 | 152,223,326 | 13 | 0.371 | 250 |
| 2 | 158,544,156 | 158,544,470 | 6 | -0.337 | 314 |
| 2 | 158,544,156 | 158,544,470 | 6 | -0.337 | 314 |
| 4 | 132,723,375 | 132,723,783 | 5 | 0.396 | 408 |
| 4 | 132,723,375 | 132,723,783 | 5 | 0.396 | 408 |
| 4 | 132,723,375 | 132,723,783 | 5 | 0.396 | 408 |
| 4 | 132,723,375 | 132,723,783 | 5 | 0.396 | 408 |
| 4 | 132,723,375 | 132,723,783 | 5 | 0.396 | 408 |
| 20 | 43,225,888 | 43,226,000 | 8 | -0.379 | 112 |
| 3 | 76,043,237 | 76,043,253 | 5 | -0.366 | 16 |
| 3 | 76,043,237 | 76,043,253 | 5 | -0.366 | 16 |
| 4 | 43,446,992 | 43,447,407 | 7 | 0.361 | 415 |

| | | | | | |
|---|---|---|---|---|---|
| 4 | 43,446,992 | 43,447,407 | 7 | 0.361 | 415 |
| 4 | 43,446,992 | 43,447,407 | 7 | 0.361 | 415 |
| 5 | 126,188,955 | 126,189,469 | 6 | 0.380 | 514 |
| 9 | 86,841,616 | 86,841,624 | 5 | -0.457 | 8 |
| 19 | 50,103,138 | 50,103,411 | 6 | 0.407 | 273 |
| 9 | 60,773,174 | 60,773,184 | 6 | -0.426 | 10 |
| 9 | 60,773,174 | 60,773,184 | 6 | -0.426 | 10 |
| 16 | 82,838,635 | 82,838,679 | 5 | 0.460 | 44 |
| 16 | 82,838,635 | 82,838,679 | 5 | 0.460 | 44 |
| 13 | 81,795,263 | 81,795,401 | 6 | 0.395 | 138 |
| 3 | 104,872,553 | 104,873,194 | 10 | 0.444 | 641 |
| 3 | 104,872,553 | 104,873,194 | 10 | 0.444 | 641 |
| 11 | 79,438,014 | 79,438,250 | 5 | 0.360 | 236 |
| 5 | 158,392,782 | 158,393,032 | 7 | -0.446 | 250 |
| 5 | 158,392,782 | 158,393,032 | 7 | -0.446 | 250 |
| 5 | 169,268,017 | 169,268,674 | 15 | -0.405 | 657 |
| 10 | 83,979,159 | 83,979,720 | 7 | -0.416 | 561 |
| 10 | 83,979,159 | 83,979,720 | 7 | -0.416 | 561 |
| 10 | 83,979,159 | 83,979,720 | 7 | -0.416 | 561 |
| 10 | 83,979,159 | 83,979,720 | 7 | -0.416 | 561 |
| 10 | 83,979,159 | 83,979,720 | 7 | -0.416 | 561 |
| 13 | 52,846,261 | 52,846,595 | 16 | 0.337 | 334 |
| 13 | 52,846,261 | 52,846,595 | 16 | 0.337 | 334 |
| 13 | 52,846,261 | 52,846,595 | 16 | 0.337 | 334 |
| 1 | 85,260,131 | 85,260,301 | 10 | -0.356 | 170 |
| 1 | 85,260,131 | 85,260,301 | 10 | -0.356 | 170 |
| 3 | 108,930,188 | 108,930,974 | 17 | 0.447 | 786 |
| 3 | 108,930,188 | 108,930,974 | 17 | 0.447 | 786 |
| 15 | 106,634,572 | 106,635,098 | 15 | 0.408 | 526 |
| 15 | 106,634,572 | 106,635,098 | 15 | 0.408 | 526 |
| 15 | 106,634,572 | 106,635,098 | 15 | 0.408 | 526 |
| 15 | 106,634,572 | 106,635,098 | 15 | 0.408 | 526 |
| 15 | 106,634,572 | 106,635,098 | 15 | 0.408 | 526 |
| 15 | 106,634,572 | 106,635,098 | 15 | 0.408 | 526 |
| 15 | 106,634,572 | 106,635,098 | 15 | 0.408 | 526 |
| 15 | 106,634,572 | 106,635,098 | 15 | 0.408 | 526 |
| 15 | 106,634,572 | 106,635,098 | 15 | 0.408 | 526 |
| 15 | 106,634,572 | 106,635,098 | 15 | 0.408 | 526 |

| | | | | | |
|---|---|---|---|---|---|
| 15 | 106,634,572 | 106,635,098 | 15 | 0.408 | 526 |
| 15 | 106,634,572 | 106,635,098 | 15 | 0.408 | 526 |
| 15 | 106,634,572 | 106,635,098 | 15 | 0.408 | 526 |
| 2 | 43,094,592 | 43,094,640 | 5 | -0.363 | 48 |
| 2 | 43,094,592 | 43,094,640 | 5 | -0.363 | 48 |
| 5 | 160,519,056 | 160,519,506 | 8 | -0.397 | 450 |
| 5 | 145,416,806 | 145,417,028 | 5 | 0.347 | 222 |
| 5 | 145,416,806 | 145,417,028 | 5 | 0.347 | 222 |
| 3 | 7,518,937 | 7,519,003 | 7 | -0.417 | 66 |
| 3 | 7,518,937 | 7,519,003 | 7 | -0.417 | 66 |
| 1 | 13,482,975 | 13,483,125 | 7 | -0.389 | 150 |
| 1 | 13,385,349 | 13,385,499 | 8 | -0.393 | 150 |
| 9 | 105,784,405 | 105,784,477 | 6 | -0.334 | 72 |
| 9 | 105,784,405 | 105,784,477 | 6 | -0.334 | 72 |
| 11 | 73,148,688 | 73,149,325 | 8 | -0.451 | 637 |
| 11 | 73,148,688 | 73,149,325 | 8 | -0.451 | 637 |
| 11 | 73,148,688 | 73,149,325 | 8 | -0.451 | 637 |
| 10 | 109,943,346 | 109,943,368 | 10 | 0.493 | 22 |
| 19 | 25,294,221 | 25,294,233 | 7 | -0.381 | 12 |
| 19 | 25,294,221 | 25,294,233 | 7 | -0.381 | 12 |
| 19 | 25,294,221 | 25,294,233 | 7 | -0.381 | 12 |
| 19 | 25,294,221 | 25,294,233 | 7 | -0.381 | 12 |
| 13 | 32,716,102 | 32,716,112 | 5 | 0.341 | 10 |
| 19 | 49,647,060 | 49,647,070 | 6 | 0.579 | 10 |
| 5 | 106,180,308 | 106,180,496 | 6 | -0.398 | 188 |
| 5 | 106,180,308 | 106,180,496 | 6 | -0.398 | 188 |
| 8 | 48,907,712 | 48,908,091 | 14 | 0.358 | 379 |
| 4 | 9,281,121 | 9,281,523 | 6 | -0.416 | 402 |
| 12 | 7,891,573 | 7,891,813 | 5 | 0.335 | 240 |
| 12 | 1,553,097 | 1,553,639 | 7 | 0.336 | 542 |
| 12 | 1,553,097 | 1,553,639 | 7 | 0.336 | 542 |
| 8 | 127,592,365 | 127,592,890 | 10 | -0.345 | 525 |
| 1 | 190,109,264 | 190,109,698 | 5 | 0.396 | 434 |
| 1 | 190,109,264 | 190,109,698 | 5 | 0.396 | 434 |
| 1 | 190,109,264 | 190,109,698 | 5 | 0.396 | 434 |
| 1 | 190,109,264 | 190,109,698 | 5 | 0.396 | 434 |
| 19 | 27,914,152 | 27,914,162 | 6 | 0.417 | 10 |
| 19 | 27,914,152 | 27,914,162 | 6 | 0.417 | 10 |

| | | | | | |
|---|---|---|---|---|---|
| 4 | 128,382,442 | 128,382,706 | 9 | -0.342 | 264 |
| 9 | 93,099,777 | 93,100,101 | 8 | -0.445 | 324 |
| 5 | 51,222,488 | 51,222,685 | 11 | -0.477 | 197 |
| 20 | 43,615,512 | 43,615,894 | 6 | -0.450 | 382 |
| 20 | 43,615,512 | 43,615,894 | 6 | -0.450 | 382 |
| 20 | 13,116,540 | 13,116,574 | 5 | -0.367 | 34 |
| 13 | 67,547,358 | 67,547,424 | 5 | 0.355 | 66 |
| 13 | 67,547,358 | 67,547,424 | 5 | 0.355 | 66 |
| 18 | 3,690,233 | 3,690,589 | 5 | -0.589 | 356 |
| X | 55,968,370 | 55,968,378 | 5 | 0.384 | 8 |
| 2 | 236,071,082 | 236,071,368 | 5 | 0.336 | 286 |
| 16 | 80,742,163 | 80,742,175 | 6 | 0.529 | 12 |
| 16 | 80,742,163 | 80,742,175 | 6 | 0.529 | 12 |
| 4 | 5,555,894 | 5,556,025 | 5 | 0.372 | 131 |
| 4 | 5,532,365 | 5,532,379 | 8 | 0.461 | 14 |
| 13 | 78,223,568 | 78,223,715 | 7 | 0.404 | 147 |
| 13 | 78,223,568 | 78,223,715 | 7 | 0.404 | 147 |
| 13 | 78,223,568 | 78,223,715 | 7 | 0.404 | 147 |
| 17 | 27,484,076 | 27,484,225 | 5 | -0.341 | 149 |
| 17 | 27,484,076 | 27,484,225 | 5 | -0.341 | 149 |
| 17 | 27,484,076 | 27,484,225 | 5 | -0.341 | 149 |
| 17 | 27,484,076 | 27,484,225 | 5 | -0.341 | 149 |
| 17 | 11,515,323 | 11,515,494 | 8 | -0.359 | 171 |
| 17 | 11,700,172 | 11,700,198 | 5 | -0.399 | 26 |
| 17 | 11,515,323 | 11,515,494 | 8 | -0.359 | 171 |
| 17 | 11,515,323 | 11,515,494 | 8 | -0.359 | 171 |
| 17 | 11,700,172 | 11,700,198 | 5 | -0.399 | 26 |
| 17 | 11,700,172 | 11,700,198 | 5 | -0.399 | 26 |
| 17 | 11,700,172 | 11,700,198 | 5 | -0.399 | 26 |
| 1 | 67,874,705 | 67,874,719 | 7 | 0.440 | 14 |
| 4 | 156,324,291 | 156,324,374 | 7 | -0.452 | 83 |
| 4 | 156,324,291 | 156,324,374 | 7 | -0.452 | 83 |
| 4 | 156,324,291 | 156,324,374 | 7 | -0.452 | 83 |
| 3 | 58,744,434 | 58,744,587 | 6 | -0.380 | 153 |
| 3 | 58,744,434 | 58,744,587 | 6 | -0.380 | 153 |
| 3 | 58,744,434 | 58,744,587 | 6 | -0.380 | 153 |
| 3 | 58,744,434 | 58,744,587 | 6 | -0.380 | 153 |
| 3 | 58,744,434 | 58,744,587 | 6 | -0.380 | 153 |

| | | | | | |
|---|---|---|---|---|---|
| 3 | 58,744,434 | 58,744,587 | 6 | -0.380 | 153 |
| 3 | 58,811,112 | 58,811,251 | 6 | -0.480 | 139 |
| 3 | 58,744,434 | 58,744,587 | 6 | -0.380 | 153 |
| 3 | 58,744,434 | 58,744,587 | 6 | -0.380 | 153 |
| 3 | 58,811,112 | 58,811,251 | 6 | -0.480 | 139 |
| 3 | 58,811,112 | 58,811,251 | 6 | -0.480 | 139 |
| 3 | 58,811,112 | 58,811,251 | 6 | -0.480 | 139 |
| 3 | 58,811,112 | 58,811,251 | 6 | -0.480 | 139 |
| 3 | 58,744,434 | 58,744,587 | 6 | -0.380 | 153 |
| 3 | 58,744,434 | 58,744,587 | 6 | -0.380 | 153 |
| 10 | 91,035,530 | 91,036,093 | 7 | 0.346 | 563 |
| 17 | 74,844,539 | 74,844,597 | 5 | 0.485 | 58 |
| 17 | 74,844,539 | 74,844,597 | 5 | 0.485 | 58 |
| 17 | 74,844,539 | 74,844,597 | 5 | 0.485 | 58 |
| 7 | 140,328,857 | 140,329,301 | 13 | 0.363 | 444 |
| 7 | 140,328,857 | 140,329,301 | 13 | 0.363 | 444 |
| 1 | 4,482,029 | 4,482,589 | 13 | -0.422 | 560 |
| 1 | 4,482,029 | 4,482,589 | 13 | -0.422 | 560 |
| 1 | 4,482,029 | 4,482,589 | 13 | -0.422 | 560 |
| 9 | 53,273,093 | 53,273,281 | 5 | 0.358 | 188 |
| 9 | 102,657,097 | 102,657,641 | 7 | 0.477 | 544 |
| 9 | 102,657,097 | 102,657,641 | 7 | 0.477 | 544 |
| 9 | 102,645,538 | 102,645,707 | 9 | 0.390 | 169 |
| 9 | 102,657,097 | 102,657,641 | 7 | 0.477 | 544 |
| 9 | 102,645,538 | 102,645,707 | 9 | 0.390 | 169 |
| 9 | 102,645,538 | 102,645,707 | 9 | 0.390 | 169 |
| 1 | 72,948,735 | 72,949,173 | 7 | 0.343 | 438 |
| 1 | 72,948,735 | 72,949,173 | 7 | 0.343 | 438 |
| 1 | 72,948,735 | 72,949,173 | 7 | 0.343 | 438 |
| 1 | 72,948,735 | 72,949,173 | 7 | 0.343 | 438 |
| 14 | 85,664,714 | 85,664,724 | 5 | 0.398 | 10 |
| 14 | 85,664,714 | 85,664,724 | 5 | 0.398 | 10 |
| 14 | 85,664,714 | 85,664,724 | 5 | 0.398 | 10 |
| 3 | 8,384,925 | 8,384,981 | 8 | 0.400 | 56 |
| 5 | 168,166,762 | 168,166,981 | 10 | 0.379 | 219 |
| 5 | 168,166,762 | 168,166,981 | 10 | 0.379 | 219 |
| 5 | 168,166,586 | 168,166,718 | 8 | 0.354 | 132 |
| 5 | 168,166,586 | 168,166,718 | 8 | 0.354 | 132 |

| | | | | | |
|---|---|---|---|---|---|
| 1 | 253,197,702 | 253,198,238 | 7 | -0.437 | 536 |
| 9 | 46,496,245 | 46,496,409 | 5 | 0.341 | 164 |
| 9 | 46,496,245 | 46,496,409 | 5 | 0.341 | 164 |
| 5 | 156,688,729 | 156,688,955 | 7 | 0.342 | 226 |
| 5 | 156,688,729 | 156,688,955 | 7 | 0.342 | 226 |
| 4 | 182,691,906 | 182,691,978 | 5 | -0.345 | 72 |
| 4 | 182,692,109 | 182,692,327 | 7 | -0.364 | 218 |
| 4 | 182,692,109 | 182,692,327 | 7 | -0.364 | 218 |
| 4 | 182,691,906 | 182,691,978 | 5 | -0.345 | 72 |
| 5 | 157,990,487 | 157,990,785 | 5 | 0.378 | 298 |
| 5 | 157,990,487 | 157,990,785 | 5 | 0.378 | 298 |
| 5 | 157,990,487 | 157,990,785 | 5 | 0.378 | 298 |
| 5 | 145,408,755 | 145,409,024 | 6 | 0.402 | 269 |
| 9 | 39,461,960 | 39,462,119 | 5 | -0.381 | 159 |
| 9 | 39,461,960 | 39,462,119 | 5 | -0.381 | 159 |
| 9 | 39,461,960 | 39,462,119 | 5 | -0.381 | 159 |
| 4 | 183,804,968 | 183,805,116 | 6 | 0.406 | 148 |
| 4 | 183,804,968 | 183,805,116 | 6 | 0.406 | 148 |
| 4 | 183,804,968 | 183,805,116 | 6 | 0.406 | 148 |
| 4 | 183,804,968 | 183,805,116 | 6 | 0.406 | 148 |
| 14 | 110,244,728 | 110,244,894 | 6 | -0.353 | 166 |
| 14 | 110,244,728 | 110,244,894 | 6 | -0.353 | 166 |
| 14 | 110,244,728 | 110,244,894 | 6 | -0.353 | 166 |
| 14 | 110,244,728 | 110,244,894 | 6 | -0.353 | 166 |
| 14 | 110,244,728 | 110,244,894 | 6 | -0.353 | 166 |
| 14 | 110,244,728 | 110,244,894 | 6 | -0.353 | 166 |
| 20 | 24,660,550 | 24,660,578 | 5 | 0.404 | 28 |
| 12 | 41,335,397 | 41,335,479 | 5 | -0.338 | 82 |
| 20 | 9,888,644 | 9,888,745 | 9 | 0.402 | 101 |
| 20 | 9,853,542 | 9,853,868 | 5 | 0.339 | 326 |
| 20 | 12,975,878 | 12,975,968 | 5 | -0.397 | 90 |
| 11 | 37,767,063 | 37,767,335 | 6 | -0.538 | 272 |
| 11 | 35,083,469 | 35,083,497 | 6 | -0.365 | 28 |
| 11 | 35,083,469 | 35,083,497 | 6 | -0.365 | 28 |
| 10 | 108,922,985 | 108,922,997 | 7 | 0.398 | 12 |
| 15 | 108,845,676 | 108,845,884 | 6 | -0.384 | 208 |
| 15 | 108,845,676 | 108,845,884 | 6 | -0.384 | 208 |
| 6 | 48,076,732 | 48,076,956 | 5 | -0.428 | 224 |

| | | | | | |
|---|---|---|---|---|---|
| 6 | 48,076,732 | 48,076,956 | 5 | -0.428 | 224 |
| 3 | 47,278,577 | 47,279,351 | 11 | 0.563 | 774 |
| 3 | 47,282,832 | 47,283,168 | 8 | 0.384 | 336 |
| 3 | 47,278,577 | 47,279,351 | 11 | 0.563 | 774 |
| 3 | 47,278,577 | 47,279,351 | 11 | 0.563 | 774 |
| 3 | 47,282,832 | 47,283,168 | 8 | 0.384 | 336 |
| 3 | 47,282,832 | 47,283,168 | 8 | 0.384 | 336 |
| 6 | 54,652,027 | 54,652,303 | 5 | 0.371 | 276 |
| 6 | 54,652,027 | 54,652,303 | 5 | 0.371 | 276 |
| 6 | 54,652,027 | 54,652,303 | 5 | 0.371 | 276 |
| 6 | 145,253,364 | 145,253,470 | 7 | 0.369 | 106 |
| 7 | 115,977,146 | 115,977,332 | 5 | -0.382 | 186 |
| 5 | 164,031,894 | 164,032,077 | 5 | -0.419 | 183 |
| 5 | 164,031,894 | 164,032,077 | 5 | -0.419 | 183 |
| 13 | 44,537,854 | 44,538,200 | 13 | 0.458 | 346 |
| 13 | 44,537,854 | 44,538,200 | 13 | 0.458 | 346 |
| 8 | 1,376,839 | 1,376,974 | 5 | -0.392 | 135 |
| 8 | 1,376,839 | 1,376,974 | 5 | -0.392 | 135 |
| 8 | 1,376,839 | 1,376,974 | 5 | -0.392 | 135 |
| 3 | 163,757,495 | 163,757,505 | 6 | -0.389 | 10 |
| 13 | 48,365,523 | 48,365,597 | 5 | -0.348 | 74 |
| 5 | 66,070,350 | 66,070,577 | 7 | 0.419 | 227 |
| 4 | 43,688,598 | 43,688,632 | 7 | -0.597 | 34 |
| 10 | 20,790,486 | 20,790,689 | 8 | -0.334 | 203 |
| 15 | 14,208,354 | 14,208,382 | 5 | 0.338 | 28 |
| 15 | 14,208,354 | 14,208,382 | 5 | 0.338 | 28 |
| 15 | 14,208,354 | 14,208,382 | 5 | 0.338 | 28 |
| 15 | 14,208,354 | 14,208,382 | 5 | 0.338 | 28 |
| 3 | 10,211,987 | 10,212,102 | 6 | 0.379 | 115 |
| 4 | 172,880,665 | 172,880,673 | 5 | -0.367 | 8 |
| 2 | 69,660,889 | 69,661,045 | 5 | -0.478 | 156 |
| 4 | 62,700,306 | 62,700,528 | 8 | -0.471 | 222 |
| 4 | 62,700,306 | 62,700,528 | 8 | -0.471 | 222 |
| 15 | 106,222,037 | 106,222,308 | 5 | -0.356 | 271 |
| 15 | 106,213,826 | 106,214,427 | 24 | -0.354 | 601 |
| 10 | 86,278,604 | 86,279,191 | 10 | 0.363 | 587 |
| 10 | 86,278,604 | 86,279,191 | 10 | 0.363 | 587 |
| 10 | 86,278,604 | 86,279,191 | 10 | 0.363 | 587 |

| | | | | | |
|---|---|---|---|---|---|
| 10 | 86,278,604 | 86,279,191 | 10 | 0.363 | 587 |
| 17 | 13,997,672 | 13,997,927 | 6 | -0.337 | 255 |
| 17 | 13,997,672 | 13,997,927 | 6 | -0.337 | 255 |
| 19 | 31,610,546 | 31,610,560 | 8 | 0.647 | 14 |
| 4 | 8,965,633 | 8,965,732 | 6 | -0.361 | 99 |
| 4 | 8,969,628 | 8,969,854 | 8 | -0.407 | 226 |
| 3 | 161,410,907 | 161,411,026 | 6 | -0.454 | 119 |
| 3 | 161,410,907 | 161,411,026 | 6 | -0.454 | 119 |
| 3 | 161,410,907 | 161,411,026 | 6 | -0.454 | 119 |
| 3 | 161,410,907 | 161,411,026 | 6 | -0.454 | 119 |
| 3 | 161,410,907 | 161,411,026 | 6 | -0.454 | 119 |
| 16 | 49,784,260 | 49,784,878 | 7 | 0.334 | 618 |
| 16 | 49,784,260 | 49,784,878 | 7 | 0.334 | 618 |
| 16 | 49,784,260 | 49,784,878 | 7 | 0.334 | 618 |
| 16 | 49,784,260 | 49,784,878 | 7 | 0.334 | 618 |
| 16 | 49,784,260 | 49,784,878 | 7 | 0.334 | 618 |
| 16 | 49,784,260 | 49,784,878 | 7 | 0.334 | 618 |
| 16 | 49,784,260 | 49,784,878 | 7 | 0.334 | 618 |
| 18 | 14,611,512 | 14,611,815 | 7 | 0.483 | 303 |
| 1 | 155,724,084 | 155,724,416 | 5 | -0.546 | 332 |
| 1 | 155,724,084 | 155,724,416 | 5 | -0.546 | 332 |
| 8 | 58,788,018 | 58,788,066 | 6 | 0.386 | 48 |
| 18 | 6,553,048 | 6,553,221 | 11 | -0.362 | 173 |
| 1 | 189,582,761 | 189,582,927 | 5 | 0.386 | 166 |
| 1 | 189,582,761 | 189,582,927 | 5 | 0.386 | 166 |
| 1 | 189,582,761 | 189,582,927 | 5 | 0.386 | 166 |
| 1 | 189,582,761 | 189,582,927 | 5 | 0.386 | 166 |
| 1 | 189,582,761 | 189,582,927 | 5 | 0.386 | 166 |
| 1 | 189,582,761 | 189,582,927 | 5 | 0.386 | 166 |
| 9 | 71,690,249 | 71,690,476 | 5 | 0.390 | 227 |
| 16 | 83,873,172 | 83,873,278 | 5 | 0.361 | 106 |
| 16 | 83,873,172 | 83,873,278 | 5 | 0.361 | 106 |
| 16 | 83,873,172 | 83,873,278 | 5 | 0.361 | 106 |
| 6 | 12,636,808 | 12,637,211 | 19 | -0.355 | 403 |
| 6 | 12,636,808 | 12,637,211 | 19 | -0.355 | 403 |
| 6 | 12,636,808 | 12,637,211 | 19 | -0.355 | 403 |
| 6 | 12,636,808 | 12,637,211 | 19 | -0.355 | 403 |
| 6 | 12,636,808 | 12,637,211 | 19 | -0.355 | 403 |

| | | | | | |
|---|---|---|---|---|---|
| 6 | 12,636,808 | 12,637,211 | 19 | -0.355 | 403 |
| 6 | 12,636,808 | 12,637,211 | 19 | -0.355 | 403 |
| 6 | 12,636,808 | 12,637,211 | 19 | -0.355 | 403 |
| 6 | 12,636,808 | 12,637,211 | 19 | -0.355 | 403 |
| 6 | 12,636,808 | 12,637,211 | 19 | -0.355 | 403 |
| 6 | 12,920,885 | 12,921,125 | 6 | 0.355 | 240 |
| 6 | 12,920,885 | 12,921,125 | 6 | 0.355 | 240 |
| 18 | 4,289,227 | 4,289,908 | 7 | -0.388 | 681 |
| 2 | 30,890,327 | 30,890,473 | 8 | -0.357 | 146 |
| 2 | 30,890,327 | 30,890,473 | 8 | -0.357 | 146 |
| 5 | 168,288,505 | 168,288,765 | 11 | 0.351 | 260 |
| 5 | 168,288,505 | 168,288,765 | 11 | 0.351 | 260 |
| 5 | 168,288,505 | 168,288,765 | 11 | 0.351 | 260 |
| 19 | 11,073,388 | 11,073,740 | 7 | 0.492 | 352 |
| 5 | 158,630,482 | 158,630,694 | 6 | -0.492 | 212 |
| 1 | 164,724,337 | 164,724,671 | 5 | 0.349 | 334 |
| 9 | 74,829,711 | 74,829,771 | 5 | 0.357 | 60 |
| 9 | 74,829,711 | 74,829,771 | 5 | 0.357 | 60 |
| 9 | 74,829,711 | 74,829,771 | 5 | 0.357 | 60 |
| 1 | 203,418,403 | 203,418,864 | 10 | 0.411 | 461 |
| 1 | 203,418,403 | 203,418,864 | 10 | 0.411 | 461 |
| 2 | 190,763,131 | 190,763,597 | 9 | -0.438 | 466 |
| 2 | 121,645,654 | 121,646,017 | 5 | -0.338 | 363 |
| 13 | 91,165,343 | 91,165,355 | 7 | 0.391 | 12 |
| 13 | 91,030,869 | 91,030,908 | 5 | 0.450 | 39 |
| 7 | 55,798,422 | 55,798,432 | 5 | -0.479 | 10 |
| 3 | 89,272,578 | 89,272,760 | 5 | 0.342 | 182 |
| 11 | 73,359,106 | 73,359,432 | 8 | 0.388 | 326 |
| 13 | 44,920,844 | 44,921,134 | 9 | 0.543 | 290 |
| 13 | 44,920,844 | 44,921,134 | 9 | 0.543 | 290 |
| 13 | 44,920,844 | 44,921,134 | 9 | 0.543 | 290 |
| 13 | 44,920,844 | 44,921,134 | 9 | 0.543 | 290 |
| 13 | 44,920,844 | 44,921,134 | 9 | 0.543 | 290 |
| 13 | 44,920,844 | 44,921,134 | 9 | 0.543 | 290 |
| 13 | 44,920,844 | 44,921,134 | 9 | 0.543 | 290 |
| 1 | 85,488,421 | 85,488,435 | 8 | 0.492 | 14 |
| 16 | 71,907,181 | 71,907,337 | 5 | -0.418 | 156 |
| 16 | 71,907,181 | 71,907,337 | 5 | -0.418 | 156 |

| | | | | | |
|---|---|---|---|---|---|
| 17 | 15,895,940 | 15,896,314 | 5 | -0.361 | 374 |
| 3 | 40,802,558 | 40,802,758 | 9 | -0.374 | 200 |
| 3 | 165,213,290 | 165,213,657 | 15 | 0.340 | 367 |
| 3 | 165,213,290 | 165,213,657 | 15 | 0.340 | 367 |
| 3 | 165,213,290 | 165,213,657 | 15 | 0.340 | 367 |
| 3 | 165,213,290 | 165,213,657 | 15 | 0.340 | 367 |
| 19 | 53,190,150 | 53,190,344 | 5 | 0.357 | 194 |
| 2 | 252,646,238 | 252,646,311 | 5 | 0.425 | 73 |
| 12 | 7,891,573 | 7,891,813 | 5 | 0.335 | 240 |
| 12 | 40,189,802 | 40,189,846 | 7 | -0.370 | 44 |
| 2 | 179,195,596 | 179,195,606 | 6 | -0.479 | 10 |
| 7 | 138,832,461 | 138,832,819 | 14 | -0.399 | 358 |
| 7 | 138,793,194 | 138,793,492 | 6 | -0.437 | 298 |
| 8 | 118,910,246 | 118,910,475 | 8 | 0.396 | 229 |
| 4 | 3,289,594 | 3,289,606 | 6 | 0.357 | 12 |
| 4 | 3,289,594 | 3,289,606 | 6 | 0.357 | 12 |
| 15 | 79,745,694 | 79,745,755 | 6 | -0.346 | 61 |
| 15 | 79,745,694 | 79,745,755 | 6 | -0.346 | 61 |
| 16 | 71,868,284 | 71,868,632 | 6 | 0.438 | 348 |
| 8 | 111,018,313 | 111,018,592 | 6 | 0.384 | 279 |
| 8 | 111,018,313 | 111,018,592 | 6 | 0.384 | 279 |
| 4 | 76,818,554 | 76,818,564 | 6 | 0.571 | 10 |
| 7 | 1,054,517 | 1,055,401 | 23 | -0.380 | 884 |
| 7 | 1,054,517 | 1,055,401 | 23 | -0.380 | 884 |
| 7 | 1,054,517 | 1,055,401 | 23 | -0.380 | 884 |
| 7 | 1,054,517 | 1,055,401 | 23 | -0.380 | 884 |
| 7 | 1,054,517 | 1,055,401 | 23 | -0.380 | 884 |
| 8 | 115,874,367 | 115,874,492 | 5 | -0.417 | 125 |
| 2 | 201,429,684 | 201,430,555 | 9 | -0.532 | 871 |
| 2 | 201,429,684 | 201,430,555 | 9 | -0.532 | 871 |
| 2 | 201,429,684 | 201,430,555 | 9 | -0.532 | 871 |
| 2 | 201,429,684 | 201,430,555 | 9 | -0.532 | 871 |
| 2 | 201,400,467 | 201,400,650 | 9 | -0.455 | 183 |
| 4 | 78,807,715 | 78,807,834 | 5 | -0.416 | 119 |
| 11 | 37,730,127 | 37,730,419 | 12 | 0.365 | 292 |
| 11 | 37,730,127 | 37,730,419 | 12 | 0.365 | 292 |
| 11 | 37,730,127 | 37,730,419 | 12 | 0.365 | 292 |
| 11 | 37,730,127 | 37,730,419 | 12 | 0.365 | 292 |

| | | | | | |
|---|---|---|---|---|---|
| 11 | 37,730,127 | 37,730,419 | 12 | 0.365 | 292 |
| 11 | 37,730,127 | 37,730,419 | 12 | 0.365 | 292 |
| 9 | 101,473,088 | 101,473,224 | 10 | -0.375 | 136 |
| 5 | 80,598,188 | 80,598,517 | 9 | -0.441 | 329 |
| 1 | 85,505,331 | 85,505,468 | 7 | -0.404 | 137 |
| 1 | 85,505,331 | 85,505,468 | 7 | -0.404 | 137 |
| 1 | 85,505,331 | 85,505,468 | 7 | -0.404 | 137 |
| 7 | 115,977,146 | 115,977,332 | 5 | -0.382 | 186 |
| 2 | 230,696,687 | 230,696,960 | 6 | -0.505 | 273 |
| 10 | 67,050,485 | 67,050,593 | 5 | -0.367 | 108 |
| 5 | 152,236,112 | 152,236,164 | 7 | 0.349 | 52 |
| 2 | 189,040,161 | 189,040,749 | 6 | -0.465 | 588 |
| 2 | 188,866,852 | 188,869,380 | 36 | -0.491 | 2528 |
| 2 | 188,866,852 | 188,869,380 | 36 | -0.491 | 2528 |
| 2 | 189,040,161 | 189,040,749 | 6 | -0.465 | 588 |
| 1 | 90,861,887 | 90,861,945 | 5 | 0.366 | 58 |
| 1 | 90,861,887 | 90,861,945 | 5 | 0.366 | 58 |
| 1 | 90,861,887 | 90,861,945 | 5 | 0.366 | 58 |
| 10 | 45,481,755 | 45,481,820 | 7 | -0.344 | 65 |
| 10 | 45,481,755 | 45,481,820 | 7 | -0.344 | 65 |
| 9 | 46,496,245 | 46,496,409 | 5 | 0.341 | 164 |
| 19 | 29,179,428 | 29,179,455 | 5 | 0.457 | 27 |
| 13 | 48,283,546 | 48,283,871 | 5 | 0.436 | 325 |
| 16 | 10,595,689 | 10,595,942 | 5 | -0.351 | 253 |
| 17 | 12,814,806 | 12,814,977 | 6 | -0.464 | 171 |
| 3 | 3,893,373 | 3,893,718 | 6 | 0.342 | 345 |
| 3 | 553,952 | 556,044 | 63 | -0.344 | 2092 |
| 13 | 81,795,263 | 81,795,401 | 6 | 0.395 | 138 |
| 1 | 165,837,009 | 165,837,361 | 6 | 0.381 | 352 |
| 2 | 614,483 | 614,731 | 9 | -0.441 | 248 |
| 20 | 10,204,465 | 10,204,620 | 5 | -0.339 | 155 |
| 2 | 188,866,852 | 188,869,380 | 36 | -0.491 | 2528 |
| 2 | 189,040,161 | 189,040,749 | 6 | -0.465 | 588 |
| 1 | 72,948,735 | 72,949,173 | 7 | 0.343 | 438 |
| 1 | 72,948,735 | 72,949,173 | 7 | 0.343 | 438 |
| 1 | 72,948,735 | 72,949,173 | 7 | 0.343 | 438 |
| 2 | 193,309,066 | 193,309,250 | 5 | -0.347 | 184 |
| 4 | 8,965,633 | 8,965,732 | 6 | -0.361 | 99 |

| | | | | | |
|---|---|---|---|---|---|
| 4 | 8,969,628 | 8,969,854 | 8 | -0.407 | 226 |
| 17 | 93,272,134 | 93,272,345 | 6 | -0.374 | 211 |
| 20 | 12,372,050 | 12,372,148 | 6 | 0.406 | 98 |
| 5 | 152,236,112 | 152,236,164 | 7 | 0.349 | 52 |
| 10 | 4,704,710 | 4,704,820 | 5 | 0.566 | 110 |
| 10 | 4,705,873 | 4,706,124 | 8 | -0.334 | 251 |
| 10 | 14,027,920 | 14,027,956 | 5 | 0.343 | 36 |
| 10 | 17,050,298 | 17,050,354 | 7 | -0.351 | 56 |
| 10 | 20,280,366 | 20,280,799 | 8 | 0.386 | 433 |
| 10 | 20,333,900 | 20,334,020 | 6 | 0.342 | 120 |
| 10 | 30,854,436 | 30,854,729 | 13 | -0.360 | 293 |
| 10 | 38,975,953 | 38,976,212 | 6 | 0.346 | 259 |
| 10 | 42,357,048 | 42,357,324 | 9 | -0.458 | 276 |
| 10 | 51,266,791 | 51,267,186 | 6 | 0.335 | 395 |
| 10 | 68,508,504 | 68,508,797 | 7 | -0.467 | 293 |
| 10 | 83,537,143 | 83,537,224 | 5 | 0.436 | 81 |
| 10 | 84,218,689 | 84,218,866 | 7 | 0.354 | 177 |
| 10 | 84,572,319 | 84,572,751 | 5 | 0.386 | 432 |
| 10 | 87,679,827 | 87,680,304 | 17 | -0.372 | 477 |
| 10 | 94,464,381 | 94,464,625 | 5 | -0.468 | 244 |
| 10 | 94,753,924 | 94,753,938 | 7 | -0.420 | 14 |
| 10 | 95,765,954 | 95,766,398 | 5 | -0.365 | 444 |
| 10 | 96,235,812 | 96,236,137 | 8 | 0.387 | 325 |
| 10 | 98,882,551 | 98,882,800 | 7 | -0.399 | 249 |
| 10 | 102,057,750 | 102,057,762 | 7 | 0.478 | 12 |
| 10 | 108,761,866 | 108,762,127 | 5 | 0.371 | 261 |
| 10 | 110,222,277 | 110,222,291 | 6 | 0.560 | 14 |
| 11 | 15,078,733 | 15,079,269 | 7 | -0.334 | 536 |
| 11 | 21,627,006 | 21,627,853 | 10 | -0.514 | 847 |
| 11 | 27,200,975 | 27,201,645 | 7 | 0.377 | 670 |
| 11 | 32,044,007 | 32,044,382 | 12 | 0.527 | 375 |
| 11 | 32,218,100 | 32,218,475 | 5 | -0.348 | 375 |
| 11 | 33,438,408 | 33,438,607 | 8 | -0.444 | 199 |
| 11 | 36,147,845 | 36,148,095 | 10 | 0.346 | 250 |
| 11 | 36,188,020 | 36,188,036 | 5 | -0.810 | 16 |
| 11 | 44,701,381 | 44,703,178 | 26 | -0.611 | 1797 |
| 11 | 53,916,032 | 53,917,466 | 31 | -0.795 | 1434 |
| 11 | 54,138,608 | 54,139,106 | 15 | -0.382 | 498 |

| | | | | | |
|---|---|---|---|---|---|
| 11 | 57,960,499 | 57,960,836 | 18 | -0.374 | 337 |
| 11 | 59,730,874 | 59,730,958 | 6 | 0.388 | 84 |
| 11 | 64,075,522 | 64,075,822 | 11 | -0.394 | 300 |
| 11 | 79,168,357 | 79,168,733 | 10 | 0.406 | 376 |
| 11 | 80,477,979 | 80,478,349 | 15 | -0.488 | 370 |
| 11 | 82,711,654 | 82,711,749 | 5 | -0.364 | 95 |
| 11 | 84,484,438 | 84,484,457 | 5 | 0.373 | 19 |
| 12 | 6,939,295 | 6,939,420 | 12 | -0.416 | 125 |
| 12 | 12,027,972 | 12,028,149 | 7 | 0.375 | 177 |
| 12 | 12,589,463 | 12,589,515 | 6 | 0.338 | 52 |
| 12 | 12,616,297 | 12,616,305 | 5 | -0.624 | 8 |
| 12 | 12,799,771 | 12,799,789 | 7 | -0.377 | 18 |
| 12 | 13,324,819 | 13,324,829 | 5 | -0.399 | 10 |
| 12 | 20,869,570 | 20,869,988 | 5 | -0.483 | 418 |
| 12 | 21,160,431 | 21,160,925 | 5 | 0.385 | 494 |
| 12 | 27,906,180 | 27,906,267 | 5 | 0.532 | 87 |
| 12 | 30,100,709 | 30,101,131 | 17 | -0.541 | 422 |
| 12 | 32,485,715 | 32,486,328 | 26 | -0.371 | 613 |
| 12 | 34,838,681 | 34,838,925 | 10 | 0.372 | 244 |
| 12 | 39,020,761 | 39,021,009 | 7 | -0.334 | 248 |
| 12 | 39,387,774 | 39,387,826 | 8 | -0.415 | 52 |
| 12 | 40,487,144 | 40,487,154 | 5 | -0.449 | 10 |
| 12 | 40,916,781 | 40,917,138 | 8 | 0.372 | 357 |
| 12 | 41,319,554 | 41,319,856 | 10 | -0.469 | 302 |
| 12 | 41,615,388 | 41,615,640 | 7 | -0.429 | 252 |
| 12 | 44,425,489 | 44,425,652 | 6 | 0.335 | 163 |
| 12 | 44,451,273 | 44,451,455 | 8 | 0.439 | 182 |
| 12 | 44,483,412 | 44,483,822 | 10 | -0.432 | 410 |
| 12 | 44,868,397 | 44,868,430 | 7 | -0.340 | 33 |
| 13 | 19,229,685 | 19,231,221 | 28 | -0.379 | 1536 |
| 13 | 31,484,465 | 31,484,580 | 6 | -0.345 | 115 |
| 13 | 33,369,484 | 33,369,742 | 5 | 0.453 | 258 |
| 13 | 41,259,346 | 41,259,386 | 8 | 0.379 | 40 |
| 13 | 45,143,860 | 45,143,954 | 5 | 0.391 | 94 |
| 13 | 47,059,752 | 47,060,075 | 11 | -0.378 | 323 |
| 13 | 48,702,882 | 48,703,649 | 11 | -0.352 | 767 |
| 13 | 55,368,926 | 55,369,197 | 5 | 0.395 | 271 |
| 13 | 69,744,528 | 69,744,812 | 13 | 0.546 | 284 |

| | | | | | |
|---|---|---|---|---|---|
| 13 | 70,083,675 | 70,084,459 | 11 | 0.547 | 784 |
| 13 | 76,909,765 | 76,909,785 | 6 | 0.398 | 20 |
| 13 | 80,055,156 | 80,055,258 | 5 | 0.367 | 102 |
| 13 | 88,389,109 | 88,389,927 | 9 | -0.404 | 818 |
| 13 | 95,067,019 | 95,067,118 | 5 | -0.378 | 99 |
| 13 | 104,927,224 | 104,927,434 | 5 | 0.346 | 210 |
| 13 | 105,199,049 | 105,199,643 | 6 | -0.384 | 594 |
| 14 | 5,264,048 | 5,264,774 | 16 | -0.397 | 726 |
| 14 | 8,848,826 | 8,849,166 | 5 | 0.361 | 340 |
| 14 | 8,872,116 | 8,872,822 | 10 | 0.425 | 706 |
| 14 | 13,562,332 | 13,562,646 | 5 | -0.362 | 314 |
| 14 | 14,102,188 | 14,102,200 | 7 | 0.818 | 12 |
| 14 | 14,711,019 | 14,711,031 | 5 | -0.678 | 12 |
| 14 | 16,717,433 | 16,717,856 | 6 | 0.340 | 423 |
| 14 | 16,816,996 | 16,817,170 | 7 | 0.389 | 174 |
| 14 | 17,107,153 | 17,107,275 | 7 | -0.367 | 122 |
| 14 | 17,107,283 | 17,107,407 | 9 | -0.406 | 124 |
| 14 | 17,701,035 | 17,701,421 | 18 | -0.603 | 386 |
| 14 | 17,931,577 | 17,931,585 | 5 | 0.355 | 8 |
| 14 | 20,865,764 | 20,865,952 | 8 | 0.394 | 188 |
| 14 | 21,367,134 | 21,367,540 | 14 | -0.548 | 406 |
| 14 | 24,261,664 | 24,262,226 | 9 | -0.580 | 562 |
| 14 | 26,833,884 | 26,834,167 | 5 | -0.911 | 283 |
| 14 | 61,805,958 | 61,806,157 | 8 | -0.394 | 199 |
| 14 | 62,352,722 | 62,353,082 | 10 | -0.370 | 360 |
| 14 | 63,661,940 | 63,662,185 | 5 | 0.470 | 245 |
| 14 | 86,083,003 | 86,083,377 | 13 | -0.344 | 374 |
| 14 | 86,128,204 | 86,128,572 | 12 | -0.425 | 368 |
| 14 | 86,435,883 | 86,435,897 | 7 | 0.591 | 14 |
| 14 | 88,296,583 | 88,296,906 | 6 | -0.376 | 323 |
| 14 | 88,456,467 | 88,457,301 | 9 | -0.368 | 834 |
| 14 | 89,443,242 | 89,443,380 | 6 | -0.450 | 138 |
| 14 | 101,655,758 | 101,656,269 | 6 | -0.468 | 511 |
| 14 | 108,176,127 | 108,176,225 | 7 | -0.339 | 98 |
| 15 | 5,869,135 | 5,869,804 | 7 | 0.339 | 669 |
| 15 | 8,850,693 | 8,850,787 | 5 | 0.391 | 94 |
| 15 | 15,142,740 | 15,142,755 | 6 | 0.394 | 15 |
| 15 | 18,456,178 | 18,456,535 | 6 | -0.494 | 357 |

| | | | | | |
|---|---|---|---|---|---|
| 15 | 21,572,516 | 21,572,943 | 6 | 0.353 | 427 |
| 15 | 22,824,298 | 22,824,989 | 20 | -0.380 | 691 |
| 15 | 23,313,384 | 23,313,502 | 6 | -0.340 | 118 |
| 15 | 24,595,187 | 24,595,329 | 9 | 0.356 | 142 |
| 15 | 27,300,849 | 27,301,185 | 29 | -0.427 | 336 |
| 15 | 42,365,123 | 42,365,285 | 10 | 0.408 | 162 |
| 15 | 44,744,991 | 44,745,001 | 6 | 0.340 | 10 |
| 15 | 55,987,460 | 55,987,529 | 5 | -0.358 | 69 |
| 15 | 56,743,948 | 56,744,265 | 12 | -0.357 | 317 |
| 15 | 57,758,396 | 57,758,780 | 13 | 0.365 | 384 |
| 15 | 58,094,873 | 58,094,883 | 5 | -0.525 | 10 |
| 15 | 72,760,851 | 72,760,872 | 6 | -0.465 | 21 |
| 15 | 77,335,380 | 77,335,962 | 8 | 0.371 | 582 |
| 15 | 81,111,332 | 81,111,610 | 5 | -0.380 | 278 |
| 15 | 84,304,359 | 84,304,586 | 7 | -0.411 | 227 |
| 15 | 88,029,688 | 88,029,738 | 5 | 0.463 | 50 |
| 15 | 93,841,409 | 93,841,786 | 15 | -0.553 | 377 |
| 16 | 7,149,404 | 7,149,414 | 6 | 0.398 | 10 |
| 16 | 7,598,049 | 7,598,360 | 9 | -0.339 | 311 |
| 16 | 7,598,461 | 7,598,995 | 18 | -0.412 | 534 |
| 16 | 8,151,034 | 8,151,253 | 5 | 0.340 | 219 |
| 16 | 9,530,413 | 9,530,648 | 6 | -0.360 | 235 |
| 16 | 11,630,080 | 11,630,161 | 5 | -0.457 | 81 |
| 16 | 18,685,521 | 18,685,789 | 5 | -0.399 | 268 |
| 16 | 48,001,588 | 48,001,848 | 6 | 0.365 | 260 |
| 16 | 60,244,096 | 60,244,459 | 12 | 0.446 | 363 |
| 16 | 61,736,320 | 61,737,309 | 13 | 0.391 | 989 |
| 16 | 63,650,013 | 63,650,447 | 9 | -0.397 | 434 |
| 16 | 76,304,525 | 76,304,591 | 7 | 0.376 | 66 |
| 16 | 79,453,377 | 79,454,242 | 16 | 0.373 | 865 |
| 16 | 80,610,801 | 80,611,175 | 6 | -0.357 | 374 |
| 17 | 1,242,459 | 1,242,752 | 8 | -0.345 | 293 |
| 17 | 1,557,714 | 1,557,730 | 9 | 0.541 | 16 |
| 17 | 12,122,893 | 12,123,384 | 9 | -0.353 | 491 |
| 17 | 13,124,225 | 13,124,237 | 7 | -0.596 | 12 |
| 17 | 13,947,230 | 13,947,377 | 8 | -0.371 | 147 |
| 17 | 14,266,533 | 14,266,705 | 10 | 0.456 | 172 |
| 17 | 19,823,963 | 19,824,163 | 8 | -0.375 | 200 |

| | | | | | |
|---|---|---|---|---|---|
| 17 | 26,821,021 | 26,821,080 | 5 | 0.358 | 59 |
| 17 | 58,993,622 | 58,993,876 | 7 | -0.785 | 254 |
| 17 | 72,627,642 | 72,627,825 | 5 | 0.337 | 183 |
| 17 | 75,206,219 | 75,206,306 | 6 | 0.376 | 87 |
| 17 | 79,063,217 | 79,063,229 | 7 | 0.505 | 12 |
| 17 | 81,236,769 | 81,236,777 | 5 | 0.549 | 8 |
| 17 | 81,418,722 | 81,418,895 | 5 | 0.391 | 173 |
| 17 | 83,795,869 | 83,796,031 | 7 | 0.385 | 162 |
| 17 | 83,934,322 | 83,934,693 | 12 | -0.343 | 371 |
| 17 | 85,778,403 | 85,778,473 | 11 | -0.561 | 70 |
| 17 | 94,501,309 | 94,502,043 | 9 | 0.375 | 734 |
| 17 | 96,381,883 | 96,381,973 | 5 | -0.440 | 90 |
| 18 | 3,782,949 | 3,783,278 | 5 | -0.370 | 329 |
| 18 | 4,917,207 | 4,917,554 | 28 | -0.456 | 347 |
| 18 | 7,849,957 | 7,850,135 | 9 | -0.579 | 178 |
| 18 | 12,232,712 | 12,233,066 | 9 | -0.350 | 354 |
| 18 | 14,833,566 | 14,834,032 | 5 | 0.423 | 466 |
| 18 | 61,491,059 | 61,491,396 | 7 | -0.365 | 337 |
| 18 | 66,411,741 | 66,412,317 | 19 | -0.441 | 576 |
| 18 | 67,276,918 | 67,277,191 | 8 | -0.510 | 273 |
| 18 | 71,065,359 | 71,065,604 | 7 | -0.494 | 245 |
| 18 | 79,319,839 | 79,320,044 | 5 | -0.377 | 205 |
| 18 | 79,328,188 | 79,328,210 | 10 | 0.415 | 22 |
| 18 | 79,537,672 | 79,538,013 | 15 | -0.405 | 341 |
| 18 | 80,057,747 | 80,058,442 | 12 | -0.436 | 695 |
| 18 | 80,287,471 | 80,287,763 | 6 | 0.363 | 292 |
| 19 | 9,534,677 | 9,535,007 | 11 | -0.361 | 330 |
| 19 | 9,601,004 | 9,601,594 | 11 | 0.412 | 590 |
| 19 | 9,601,995 | 9,602,372 | 8 | 0.470 | 377 |
| 19 | 9,658,005 | 9,658,053 | 5 | -0.359 | 48 |
| 19 | 10,947,815 | 10,948,338 | 7 | -0.383 | 523 |
| 19 | 14,393,360 | 14,393,522 | 6 | -0.392 | 162 |
| 19 | 16,123,980 | 16,124,086 | 6 | 0.395 | 106 |
| 19 | 28,552,417 | 28,552,691 | 5 | 0.388 | 274 |
| 19 | 31,186,730 | 31,186,883 | 5 | 0.392 | 153 |
| 19 | 31,259,974 | 31,260,020 | 5 | 0.409 | 46 |
| 19 | 51,166,797 | 51,166,881 | 7 | -0.368 | 84 |
| 19 | 53,062,836 | 53,062,972 | 5 | -0.494 | 136 |

| | | | | | |
|---|---|---|---|---|---|
| 19 | 54,469,315 | 54,470,018 | 18 | 0.365 | 703 |
| 19 | 55,536,781 | 55,537,070 | 10 | 0.415 | 289 |
| 19 | 56,794,214 | 56,794,321 | 7 | -0.340 | 107 |
| 1 | 3,545,143 | 3,545,197 | 5 | 0.368 | 54 |
| 1 | 16,183,742 | 16,183,875 | 6 | 0.356 | 133 |
| 1 | 23,871,506 | 23,871,728 | 13 | -0.344 | 222 |
| 1 | 29,503,064 | 29,503,322 | 5 | 0.349 | 258 |
| 1 | 29,687,833 | 29,688,292 | 18 | -0.414 | 459 |
| 1 | 32,562,798 | 32,562,965 | 5 | -0.876 | 167 |
| 1 | 33,608,227 | 33,608,305 | 5 | 0.338 | 78 |
| 1 | 41,995,038 | 41,995,219 | 8 | 0.357 | 181 |
| 1 | 43,138,772 | 43,139,161 | 10 | -0.372 | 389 |
| 1 | 44,423,113 | 44,423,149 | 9 | -0.404 | 36 |
| 1 | 47,450,193 | 47,450,467 | 6 | 0.378 | 274 |
| 1 | 48,693,899 | 48,694,020 | 5 | 0.418 | 121 |
| 1 | 57,633,053 | 57,633,130 | 6 | -0.622 | 77 |
| 1 | 68,921,090 | 68,921,620 | 6 | -0.817 | 530 |
| 1 | 73,359,669 | 73,359,894 | 6 | 0.342 | 225 |
| 1 | 86,771,899 | 86,772,247 | 8 | 0.432 | 348 |
| 1 | 90,559,345 | 90,559,377 | 6 | -0.517 | 32 |
| 1 | 90,920,533 | 90,920,599 | 5 | -0.394 | 66 |
| 1 | 109,295,714 | 109,296,091 | 7 | -0.490 | 377 |
| 1 | 113,685,199 | 113,685,474 | 5 | 0.334 | 275 |
| 1 | 122,387,400 | 122,387,744 | 5 | 0.475 | 344 |
| 1 | 123,029,087 | 123,029,330 | 6 | 0.356 | 243 |
| 1 | 135,917,173 | 135,917,281 | 6 | -0.337 | 108 |
| 1 | 135,960,566 | 135,961,604 | 13 | -0.421 | 1038 |
| 1 | 136,748,500 | 136,749,205 | 9 | 0.398 | 705 |
| 1 | 137,988,208 | 137,988,275 | 8 | -0.499 | 67 |
| 1 | 141,967,317 | 141,967,415 | 5 | -0.867 | 98 |
| 1 | 145,329,382 | 145,329,505 | 6 | -0.463 | 123 |
| 1 | 155,558,438 | 155,558,741 | 5 | -0.341 | 303 |
| 1 | 160,556,558 | 160,556,870 | 5 | 0.346 | 312 |
| 1 | 161,880,578 | 161,881,039 | 5 | -0.486 | 461 |
| 1 | 161,905,186 | 161,905,622 | 7 | -0.431 | 436 |
| 1 | 166,770,296 | 166,770,616 | 10 | -0.362 | 320 |
| 1 | 169,571,608 | 169,571,628 | 6 | -0.388 | 20 |
| 1 | 176,824,197 | 176,824,278 | 7 | -0.354 | 81 |

| | | | | | |
|---|---|---|---|---|---|
| 1 | 182,588,194 | 182,588,206 | 7 | 0.397 | 12 |
| 1 | 185,305,067 | 185,305,208 | 5 | -0.349 | 141 |
| 1 | 185,446,433 | 185,446,634 | 5 | -0.562 | 201 |
| 1 | 188,209,055 | 188,209,172 | 9 | 0.361 | 117 |
| 1 | 190,728,899 | 190,729,117 | 12 | -0.416 | 218 |
| 1 | 195,152,377 | 195,152,459 | 6 | 0.376 | 82 |
| 1 | 198,618,353 | 198,618,534 | 6 | 0.349 | 181 |
| 1 | 201,137,065 | 201,137,097 | 10 | 0.353 | 32 |
| 1 | 212,829,112 | 212,829,340 | 7 | 0.530 | 228 |
| 1 | 217,848,881 | 217,849,198 | 9 | 0.352 | 317 |
| 1 | 218,616,243 | 218,616,253 | 5 | 0.454 | 10 |
| 1 | 219,305,420 | 219,305,524 | 5 | 0.349 | 104 |
| 1 | 224,808,808 | 224,808,837 | 10 | -0.366 | 29 |
| 1 | 230,436,180 | 230,436,641 | 5 | -0.365 | 461 |
| 1 | 231,693,413 | 231,693,429 | 7 | 0.362 | 16 |
| 1 | 231,866,213 | 231,866,746 | 9 | 0.342 | 533 |
| 1 | 232,327,859 | 232,327,885 | 11 | 0.395 | 26 |
| 1 | 232,365,692 | 232,365,882 | 6 | -0.571 | 190 |
| 1 | 233,044,343 | 233,044,706 | 8 | -0.362 | 363 |
| 1 | 237,307,573 | 237,307,779 | 5 | 0.348 | 206 |
| 1 | 246,185,028 | 246,185,044 | 5 | 0.652 | 16 |
| 1 | 249,379,076 | 249,379,164 | 5 | 0.364 | 88 |
| 1 | 251,747,451 | 251,747,840 | 10 | 0.384 | 389 |
| 1 | 257,458,894 | 257,459,061 | 7 | -0.374 | 167 |
| 20 | 10,305,066 | 10,305,230 | 8 | 0.464 | 164 |
| 20 | 11,113,699 | 11,114,046 | 9 | -0.398 | 347 |
| 20 | 11,796,746 | 11,796,829 | 7 | -0.430 | 83 |
| 20 | 12,008,483 | 12,008,499 | 9 | -0.383 | 16 |
| 20 | 13,170,234 | 13,170,435 | 11 | 0.340 | 201 |
| 20 | 13,177,762 | 13,177,843 | 5 | -0.461 | 81 |
| 20 | 15,016,421 | 15,016,433 | 6 | -0.369 | 12 |
| 20 | 22,575,092 | 22,575,100 | 5 | 0.413 | 8 |
| 20 | 42,541,167 | 42,541,305 | 11 | -0.539 | 138 |
| 20 | 42,610,377 | 42,610,827 | 5 | -0.557 | 450 |
| 20 | 42,611,428 | 42,612,124 | 7 | -0.388 | 696 |
| 20 | 42,729,223 | 42,729,264 | 7 | 0.360 | 41 |
| 20 | 47,205,245 | 47,205,641 | 10 | -0.455 | 396 |
| 20 | 48,506,882 | 48,507,062 | 10 | 0.340 | 180 |

| | | | | | |
|---|---|---|---|---|---|
| 2 | 56,550 | 56,734 | 6 | -0.976 | 184 |
| 2 | 1,398,583 | 1,398,907 | 7 | 0.426 | 324 |
| 2 | 1,417,736 | 1,418,062 | 9 | 0.394 | 326 |
| 2 | 1,422,574 | 1,423,010 | 7 | 0.347 | 436 |
| 2 | 9,976,063 | 9,976,336 | 10 | 0.457 | 273 |
| 2 | 27,201,383 | 27,201,609 | 5 | 0.397 | 226 |
| 2 | 30,855,651 | 30,855,663 | 6 | -0.353 | 12 |
| 2 | 35,456,503 | 35,456,973 | 22 | 0.339 | 470 |
| 2 | 37,855,849 | 37,855,958 | 5 | 0.335 | 109 |
| 2 | 38,061,801 | 38,062,086 | 10 | 0.692 | 285 |
| 2 | 38,115,342 | 38,115,356 | 8 | 0.484 | 14 |
| 2 | 39,235,842 | 39,235,854 | 5 | -0.450 | 12 |
| 2 | 41,108,579 | 41,108,683 | 6 | -0.446 | 104 |
| 2 | 41,673,646 | 41,673,704 | 5 | 0.346 | 58 |
| 2 | 43,215,242 | 43,215,437 | 9 | -0.336 | 195 |
| 2 | 43,544,068 | 43,544,517 | 5 | -0.464 | 449 |
| 2 | 43,939,396 | 43,939,968 | 8 | -0.372 | 572 |
| 2 | 51,914,492 | 51,915,168 | 25 | -0.730 | 676 |
| 2 | 52,092,205 | 52,092,562 | 11 | -0.361 | 357 |
| 2 | 52,243,597 | 52,245,294 | 19 | -0.557 | 1697 |
| 2 | 61,992,979 | 61,993,150 | 5 | -0.350 | 171 |
| 2 | 78,257,140 | 78,257,317 | 5 | -0.377 | 177 |
| 2 | 79,234,925 | 79,234,933 | 5 | -0.423 | 8 |
| 2 | 95,084,230 | 95,084,564 | 8 | -0.410 | 334 |
| 2 | 105,636,120 | 105,636,252 | 9 | -0.372 | 132 |
| 2 | 108,272,982 | 108,273,415 | 6 | -0.335 | 433 |
| 2 | 115,511,994 | 115,512,568 | 9 | 0.346 | 574 |
| 2 | 116,343,828 | 116,343,905 | 8 | -0.363 | 77 |
| 2 | 116,375,575 | 116,375,637 | 8 | -0.346 | 62 |
| 2 | 116,712,918 | 116,713,053 | 8 | -0.541 | 135 |
| 2 | 116,713,108 | 116,713,412 | 10 | -0.461 | 304 |
| 2 | 123,692,625 | 123,692,663 | 5 | 0.392 | 38 |
| 2 | 128,066,725 | 128,067,333 | 8 | -0.565 | 608 |
| 2 | 139,848,274 | 139,848,613 | 6 | 0.374 | 339 |
| 2 | 141,230,225 | 141,230,341 | 7 | 0.382 | 116 |
| 2 | 143,978,899 | 143,979,077 | 5 | -0.382 | 178 |
| 2 | 148,133,691 | 148,133,853 | 7 | 0.338 | 162 |
| 2 | 157,126,015 | 157,126,111 | 5 | -0.448 | 96 |

| | | | | | |
|---|---|---|---|---|---|
| 2 | 158,660,017 | 158,660,357 | 10 | -0.431 | 340 |
| 2 | 175,522,666 | 175,522,678 | 7 | -0.414 | 12 |
| 2 | 178,932,423 | 178,932,501 | 8 | 0.334 | 78 |
| 2 | 180,346,831 | 180,347,515 | 8 | -0.369 | 684 |
| 2 | 186,902,654 | 186,902,960 | 8 | -0.903 | 306 |
| 2 | 190,871,855 | 190,872,024 | 5 | -0.433 | 169 |
| 2 | 191,064,065 | 191,064,367 | 8 | 0.355 | 302 |
| 2 | 191,221,265 | 191,221,559 | 14 | -0.367 | 294 |
| 2 | 194,148,517 | 194,148,623 | 5 | 0.378 | 106 |
| 2 | 195,756,917 | 195,757,044 | 6 | 0.369 | 127 |
| 2 | 195,818,334 | 195,818,485 | 5 | -0.462 | 151 |
| 2 | 196,575,140 | 196,575,390 | 5 | 0.361 | 250 |
| 2 | 211,523,568 | 211,523,774 | 6 | -0.747 | 206 |
| 2 | 214,093,970 | 214,094,330 | 7 | -0.361 | 360 |
| 2 | 221,948,929 | 221,949,202 | 7 | -0.352 | 273 |
| 2 | 227,102,981 | 227,103,088 | 5 | 0.406 | 107 |
| 2 | 227,794,362 | 227,794,391 | 5 | 0.387 | 29 |
| 2 | 227,988,449 | 227,988,574 | 5 | -0.347 | 125 |
| 2 | 230,341,162 | 230,341,294 | 6 | -0.355 | 132 |
| 2 | 231,590,502 | 231,591,567 | 17 | -0.344 | 1065 |
| 2 | 233,242,743 | 233,242,812 | 6 | -0.404 | 69 |
| 2 | 234,986,842 | 234,987,115 | 6 | 0.361 | 273 |
| 2 | 236,138,691 | 236,139,021 | 6 | 0.376 | 330 |
| 2 | 239,217,876 | 239,218,547 | 7 | -0.466 | 671 |
| 2 | 251,410,667 | 251,410,677 | 5 | 0.337 | 10 |
| 2 | 252,537,683 | 252,538,185 | 6 | 0.396 | 502 |
| 2 | 256,700,368 | 256,700,862 | 9 | -0.337 | 494 |
| 3 | 12,487,802 | 12,487,908 | 7 | -0.354 | 106 |
| 3 | 12,993,798 | 12,994,104 | 28 | -0.339 | 306 |
| 3 | 14,630,385 | 14,630,860 | 13 | 0.382 | 475 |
| 3 | 23,726,828 | 23,727,332 | 10 | -0.594 | 504 |
| 3 | 29,585,856 | 29,586,479 | 9 | 0.399 | 623 |
| 3 | 32,151,284 | 32,151,651 | 5 | 0.363 | 367 |
| 3 | 34,306,039 | 34,306,243 | 6 | -0.422 | 204 |
| 3 | 46,463,161 | 46,463,981 | 9 | 0.382 | 820 |
| 3 | 47,194,698 | 47,195,039 | 6 | 0.364 | 341 |
| 3 | 50,213,939 | 50,215,260 | 15 | -0.570 | 1321 |
| 3 | 57,799,122 | 57,799,141 | 5 | -0.396 | 19 |

| | | | | | |
|---|---|---|---|---|---|
| 3 | 64,084,076 | 64,084,383 | 6 | -0.702 | 307 |
| 3 | 64,086,232 | 64,086,370 | 6 | -0.344 | 138 |
| 3 | 67,183,130 | 67,183,201 | 8 | 0.337 | 71 |
| 3 | 67,941,667 | 67,941,953 | 6 | 0.579 | 286 |
| 3 | 69,154,149 | 69,154,175 | 8 | -0.667 | 26 |
| 3 | 72,442,837 | 72,443,281 | 5 | -0.360 | 444 |
| 3 | 76,929,695 | 76,929,810 | 5 | 0.582 | 115 |
| 3 | 77,083,124 | 77,083,146 | 6 | 0.339 | 22 |
| 3 | 93,674,356 | 93,674,827 | 5 | -0.391 | 471 |
| 3 | 93,694,490 | 93,694,588 | 5 | -0.438 | 98 |
| 3 | 96,435,433 | 96,435,505 | 12 | -0.520 | 72 |
| 3 | 104,118,099 | 104,118,109 | 6 | 0.501 | 10 |
| 3 | 114,616,843 | 114,616,980 | 5 | 0.392 | 137 |
| 3 | 117,352,061 | 117,352,272 | 17 | -0.402 | 211 |
| 3 | 117,521,016 | 117,521,200 | 5 | -0.370 | 184 |
| 3 | 119,574,254 | 119,574,918 | 9 | -0.515 | 664 |
| 3 | 120,089,243 | 120,089,379 | 7 | 0.387 | 136 |
| 3 | 126,276,367 | 126,276,570 | 5 | 0.347 | 203 |
| 3 | 132,344,167 | 132,344,434 | 8 | 0.362 | 267 |
| 3 | 134,300,110 | 134,300,300 | 9 | -0.377 | 190 |
| 3 | 137,267,374 | 137,267,689 | 5 | 0.384 | 315 |
| 3 | 140,335,458 | 140,335,494 | 12 | 0.483 | 36 |
| 3 | 154,337,007 | 154,337,738 | 15 | 0.352 | 731 |
| 3 | 156,721,483 | 156,722,103 | 8 | 0.341 | 620 |
| 3 | 160,619,387 | 160,619,710 | 10 | 0.402 | 323 |
| 3 | 161,729,565 | 161,729,636 | 6 | 0.366 | 71 |
| 3 | 162,064,561 | 162,064,761 | 5 | -0.507 | 200 |
| 3 | 162,091,892 | 162,091,906 | 7 | -0.374 | 14 |
| 3 | 163,836,387 | 163,837,926 | 25 | -0.697 | 1539 |
| 3 | 165,156,364 | 165,156,698 | 6 | -0.343 | 334 |
| 3 | 167,981,130 | 167,981,317 | 5 | -0.376 | 187 |
| 3 | 168,174,696 | 168,174,743 | 6 | -0.364 | 47 |
| 3 | 169,383,448 | 169,383,724 | 5 | 0.401 | 276 |
| 3 | 169,510,251 | 169,510,691 | 5 | 0.387 | 440 |
| 4 | 3,935,417 | 3,935,628 | 5 | -0.406 | 211 |
| 4 | 5,458,426 | 5,458,809 | 5 | 0.377 | 383 |
| 4 | 6,602,168 | 6,602,453 | 6 | -0.457 | 285 |
| 4 | 29,239,435 | 29,239,475 | 11 | 0.342 | 40 |

| | | | | | |
|---|---|---|---|---|---|
| 4 | 53,410,271 | 53,410,669 | 5 | -0.391 | 398 |
| 4 | 55,767,468 | 55,767,614 | 9 | 0.385 | 146 |
| 4 | 61,786,033 | 61,787,332 | 24 | -0.492 | 1299 |
| 4 | 72,734,940 | 72,735,530 | 9 | -0.371 | 590 |
| 4 | 75,488,281 | 75,488,849 | 8 | -0.432 | 568 |
| 4 | 79,854,309 | 79,854,508 | 6 | -0.418 | 199 |
| 4 | 85,219,080 | 85,219,435 | 9 | -0.371 | 355 |
| 4 | 86,942,587 | 86,942,727 | 7 | -0.407 | 140 |
| 4 | 90,102,528 | 90,102,945 | 5 | -0.429 | 417 |
| 4 | 111,866,079 | 111,866,103 | 6 | 0.353 | 24 |
| 4 | 116,151,441 | 116,151,580 | 12 | -0.569 | 139 |
| 4 | 117,691,037 | 117,691,315 | 8 | -0.381 | 278 |
| 4 | 117,886,680 | 117,887,020 | 6 | 0.342 | 340 |
| 4 | 121,517,259 | 121,517,269 | 5 | -0.352 | 10 |
| 4 | 126,400,182 | 126,400,194 | 6 | 0.337 | 12 |
| 4 | 128,706,610 | 128,706,631 | 7 | -0.450 | 21 |
| 4 | 130,650,847 | 130,651,235 | 5 | -0.418 | 388 |
| 4 | 134,971,817 | 134,972,029 | 7 | 0.457 | 212 |
| 4 | 140,992,173 | 140,992,289 | 5 | -0.529 | 116 |
| 4 | 160,549,806 | 160,550,357 | 12 | 0.354 | 551 |
| 4 | 172,691,852 | 172,691,862 | 5 | -0.428 | 10 |
| 4 | 182,402,686 | 182,402,696 | 5 | 0.501 | 10 |
| 4 | 182,757,073 | 182,757,548 | 7 | 0.352 | 475 |
| 4 | 184,007,716 | 184,007,732 | 5 | 0.371 | 16 |
| 5 | 5,440,468 | 5,440,584 | 5 | 0.344 | 116 |
| 5 | 26,924,860 | 26,924,911 | 5 | -0.434 | 51 |
| 5 | 36,022,764 | 36,023,361 | 7 | -0.597 | 597 |
| 5 | 36,077,115 | 36,077,268 | 5 | -0.507 | 153 |
| 5 | 36,247,948 | 36,248,255 | 6 | -0.620 | 307 |
| 5 | 36,278,391 | 36,278,435 | 7 | -0.345 | 44 |
| 5 | 39,451,332 | 39,451,493 | 7 | -0.574 | 161 |
| 5 | 57,654,069 | 57,654,187 | 8 | -0.388 | 118 |
| 5 | 58,275,643 | 58,275,834 | 6 | -0.560 | 191 |
| 5 | 67,318,448 | 67,318,726 | 9 | -0.462 | 278 |
| 5 | 73,779,818 | 73,779,828 | 6 | 0.436 | 10 |
| 5 | 79,777,986 | 79,778,245 | 8 | 0.510 | 259 |
| 5 | 79,798,372 | 79,798,529 | 5 | -0.392 | 157 |
| 5 | 83,361,352 | 83,361,594 | 10 | -0.864 | 242 |

| | | | | | |
|---|---|---|---|---|---|
| 5 | 88,728,693 | 88,729,130 | 6 | -0.410 | 437 |
| 5 | 96,950,610 | 96,950,819 | 5 | 0.342 | 209 |
| 5 | 100,677,585 | 100,677,783 | 5 | 0.373 | 198 |
| 5 | 102,656,488 | 102,656,549 | 5 | -1.000 | 61 |
| 5 | 102,707,853 | 102,707,977 | 5 | -0.350 | 124 |
| 5 | 102,733,734 | 102,733,878 | 9 | -0.586 | 144 |
| 5 | 105,613,567 | 105,613,772 | 8 | -0.402 | 205 |
| 5 | 105,746,565 | 105,746,988 | 6 | 0.396 | 423 |
| 5 | 106,633,031 | 106,633,125 | 6 | -0.339 | 94 |
| 5 | 108,019,292 | 108,019,646 | 5 | -0.804 | 354 |
| 5 | 113,641,785 | 113,641,902 | 5 | -0.399 | 117 |
| 5 | 115,246,541 | 115,246,741 | 6 | -0.452 | 200 |
| 5 | 117,567,960 | 117,568,285 | 6 | -0.393 | 325 |
| 5 | 118,070,742 | 118,070,938 | 5 | -0.568 | 196 |
| 5 | 120,153,733 | 120,154,068 | 12 | 0.393 | 335 |
| 5 | 123,013,340 | 123,013,578 | 5 | 0.430 | 238 |
| 5 | 126,550,471 | 126,550,718 | 5 | 0.372 | 247 |
| 5 | 127,020,379 | 127,020,502 | 5 | -0.336 | 123 |
| 5 | 128,594,695 | 128,594,705 | 6 | 0.485 | 10 |
| 5 | 128,896,139 | 128,897,021 | 12 | 0.437 | 882 |
| 5 | 138,971,438 | 138,971,699 | 9 | -0.452 | 261 |
| 5 | 140,934,001 | 140,934,207 | 6 | 0.440 | 206 |
| 5 | 141,797,853 | 141,798,055 | 5 | -0.488 | 202 |
| 5 | 155,893,583 | 155,893,861 | 5 | 0.356 | 278 |
| 5 | 156,245,625 | 156,245,854 | 7 | 0.349 | 229 |
| 5 | 160,566,625 | 160,566,924 | 5 | -0.386 | 299 |
| 5 | 165,497,390 | 165,497,668 | 11 | -0.385 | 278 |
| 5 | 166,878,934 | 166,879,727 | 15 | 0.376 | 793 |
| 5 | 168,064,748 | 168,064,764 | 5 | 0.462 | 16 |
| 5 | 168,616,463 | 168,616,813 | 5 | -0.344 | 350 |
| 5 | 169,075,201 | 169,075,215 | 7 | 0.433 | 14 |
| 5 | 169,973,593 | 169,973,692 | 7 | 0.375 | 99 |
| 5 | 170,073,577 | 170,073,601 | 7 | 0.444 | 24 |
| 5 | 171,346,780 | 171,346,839 | 5 | -0.349 | 59 |
| 5 | 171,741,306 | 171,741,755 | 15 | 0.461 | 449 |
| 6 | 3,958,378 | 3,958,474 | 6 | 0.356 | 96 |
| 6 | 3,958,488 | 3,958,768 | 7 | 0.346 | 280 |
| 6 | 11,116,063 | 11,116,423 | 7 | 0.375 | 360 |

| | | | | | |
|---|---|---|---|---|---|
| 6 | 19,707,101 | 19,707,109 | 5 | 0.415 | 8 |
| 6 | 23,137,375 | 23,137,614 | 5 | 0.442 | 239 |
| 6 | 27,023,172 | 27,023,461 | 7 | -0.454 | 289 |
| 6 | 36,456,612 | 36,457,147 | 7 | 0.343 | 535 |
| 6 | 70,760,115 | 70,760,215 | 5 | -0.356 | 100 |
| 6 | 77,907,512 | 77,907,578 | 5 | 0.360 | 66 |
| 6 | 90,839,015 | 90,839,150 | 5 | 0.426 | 135 |
| 6 | 90,911,155 | 90,911,486 | 9 | -0.340 | 331 |
| 6 | 95,232,527 | 95,232,839 | 19 | -0.366 | 312 |
| 6 | 98,964,414 | 98,964,779 | 6 | -0.420 | 365 |
| 6 | 104,674,252 | 104,674,432 | 7 | -0.412 | 180 |
| 6 | 104,798,507 | 104,798,577 | 5 | -0.373 | 70 |
| 6 | 115,224,254 | 115,224,995 | 16 | 0.442 | 741 |
| 6 | 116,773,361 | 116,773,536 | 5 | -0.531 | 175 |
| 6 | 117,003,602 | 117,003,834 | 6 | 0.363 | 232 |
| 6 | 129,210,761 | 129,210,777 | 9 | 0.473 | 16 |
| 6 | 129,219,304 | 129,219,632 | 7 | 0.385 | 328 |
| 6 | 129,423,482 | 129,423,623 | 6 | 0.360 | 141 |
| 6 | 129,614,102 | 129,614,114 | 6 | -0.476 | 12 |
| 6 | 130,375,980 | 130,376,000 | 9 | -0.339 | 20 |
| 6 | 130,949,892 | 130,949,900 | 5 | -0.544 | 8 |
| 6 | 135,431,998 | 135,432,006 | 5 | -0.518 | 8 |
| 6 | 135,902,803 | 135,903,168 | 7 | -0.434 | 365 |
| 6 | 144,584,469 | 144,584,963 | 5 | -0.387 | 494 |
| 6 | 144,710,452 | 144,710,842 | 15 | -0.538 | 390 |
| 7 | 864,109 | 864,189 | 5 | -0.385 | 80 |
| 7 | 4,615,246 | 4,626,524 | 144 | -0.404 | 11278 |
| 7 | 4,626,647 | 4,632,113 | 59 | -0.403 | 5466 |
| 7 | 4,867,932 | 4,868,647 | 15 | -0.388 | 715 |
| 7 | 9,520,101 | 9,520,210 | 6 | -0.336 | 109 |
| 7 | 13,204,449 | 13,204,588 | 5 | 0.476 | 139 |
| 7 | 21,161,066 | 21,161,078 | 6 | -0.394 | 12 |
| 7 | 30,480,469 | 30,480,796 | 5 | 0.397 | 327 |
| 7 | 32,585,526 | 32,586,089 | 17 | -0.353 | 563 |
| 7 | 32,634,381 | 32,634,595 | 5 | 0.349 | 214 |
| 7 | 32,983,071 | 32,983,400 | 7 | -0.351 | 329 |
| 7 | 33,651,342 | 33,651,557 | 5 | 0.544 | 215 |
| 7 | 35,711,969 | 35,712,290 | 11 | -0.893 | 321 |

| | | | | | |
|---|---|---|---|---|---|
| 7 | 36,263,457 | 36,263,674 | 13 | -0.408 | 217 |
| 7 | 36,914,777 | 36,914,842 | 5 | 0.367 | 65 |
| 7 | 37,262,681 | 37,262,736 | 5 | -0.369 | 55 |
| 7 | 50,099,901 | 50,100,060 | 11 | 0.374 | 159 |
| 7 | 50,390,326 | 50,391,256 | 10 | 0.497 | 930 |
| 7 | 50,599,893 | 50,600,183 | 5 | -0.359 | 290 |
| 7 | 66,255,539 | 66,255,551 | 6 | 0.335 | 12 |
| 7 | 66,442,386 | 66,443,050 | 10 | 0.419 | 664 |
| 7 | 74,383,994 | 74,384,372 | 7 | 0.461 | 378 |
| 7 | 75,696,412 | 75,696,446 | 5 | 0.438 | 34 |
| 7 | 87,976,093 | 87,976,109 | 7 | -0.691 | 16 |
| 7 | 110,904,010 | 110,904,024 | 8 | -0.456 | 14 |
| 7 | 116,068,038 | 116,068,348 | 6 | 0.366 | 310 |
| 7 | 119,492,534 | 119,492,548 | 8 | 0.505 | 14 |
| 7 | 130,636,608 | 130,636,875 | 5 | 0.440 | 267 |
| 7 | 132,254,684 | 132,254,885 | 7 | -0.351 | 201 |
| 7 | 133,087,047 | 133,087,274 | 5 | 0.415 | 227 |
| 7 | 133,293,707 | 133,294,009 | 8 | -0.498 | 302 |
| 7 | 133,328,784 | 133,329,853 | 15 | -0.403 | 1069 |
| 7 | 134,669,819 | 134,670,271 | 12 | 0.496 | 452 |
| 7 | 134,804,618 | 134,805,218 | 12 | -0.396 | 600 |
| 7 | 135,168,139 | 135,168,421 | 10 | 0.385 | 282 |
| 7 | 135,169,059 | 135,169,279 | 8 | -0.412 | 220 |
| 7 | 135,223,267 | 135,224,824 | 18 | -0.335 | 1557 |
| 7 | 136,489,031 | 136,489,244 | 5 | 0.380 | 213 |
| 7 | 136,622,250 | 136,623,000 | 20 | 0.559 | 750 |
| 7 | 136,623,062 | 136,623,428 | 6 | 0.351 | 366 |
| 7 | 137,046,707 | 137,046,811 | 8 | -0.342 | 104 |
| 7 | 139,143,178 | 139,143,186 | 5 | -0.358 | 8 |
| 7 | 139,322,835 | 139,323,756 | 16 | 0.433 | 921 |
| 7 | 141,802,638 | 141,803,341 | 8 | -0.382 | 703 |
| 8 | 10,681,179 | 10,681,373 | 10 | 0.425 | 194 |
| 8 | 11,929,643 | 11,930,143 | 8 | 0.395 | 500 |
| 8 | 18,049,989 | 18,050,035 | 8 | 0.653 | 46 |
| 8 | 23,491,151 | 23,491,379 | 5 | 0.357 | 228 |
| 8 | 57,390,468 | 57,390,607 | 7 | -0.397 | 139 |
| 8 | 59,006,344 | 59,006,370 | 13 | 0.341 | 26 |
| 8 | 60,551,619 | 60,551,943 | 10 | 0.364 | 324 |

| | | | | | |
|---|---|---|---|---|---|
| 8 | 66,010,174 | 66,010,606 | 9 | -0.335 | 432 |
| 8 | 72,378,554 | 72,378,782 | 6 | 0.347 | 228 |
| 8 | 84,094,124 | 84,094,136 | 7 | -0.852 | 12 |
| 8 | 87,710,920 | 87,711,201 | 7 | 0.463 | 281 |
| 8 | 95,291,672 | 95,291,744 | 5 | -0.377 | 72 |
| 8 | 97,369,493 | 97,370,050 | 7 | -0.402 | 557 |
| 8 | 107,728,813 | 107,728,956 | 7 | -0.356 | 143 |
| 8 | 110,984,569 | 110,985,050 | 6 | 0.443 | 481 |
| 8 | 111,007,217 | 111,007,241 | 7 | -0.440 | 24 |
| 8 | 115,319,814 | 115,320,386 | 25 | -0.550 | 572 |
| 8 | 115,320,552 | 115,320,639 | 6 | -0.394 | 87 |
| 8 | 116,821,213 | 116,821,223 | 6 | -0.512 | 10 |
| 8 | 117,966,482 | 117,966,773 | 5 | -0.387 | 291 |
| 8 | 118,694,160 | 118,694,426 | 8 | -0.376 | 266 |
| 8 | 123,108,620 | 123,108,628 | 5 | 0.355 | 8 |
| 8 | 123,839,405 | 123,839,890 | 5 | 0.337 | 485 |
| 8 | 125,603,518 | 125,604,479 | 27 | -0.415 | 961 |
| 8 | 127,442,152 | 127,442,221 | 6 | -0.485 | 69 |
| 8 | 127,913,397 | 127,913,874 | 9 | -0.386 | 477 |
| 9 | 3,433,147 | 3,433,480 | 6 | -0.336 | 333 |
| 9 | 6,408,584 | 6,408,592 | 5 | -0.563 | 8 |
| 9 | 8,717,866 | 8,718,141 | 5 | -0.451 | 275 |
| 9 | 8,875,311 | 8,875,355 | 6 | -0.492 | 44 |
| 9 | 9,068,595 | 9,068,727 | 5 | 0.334 | 132 |
| 9 | 9,068,734 | 9,069,023 | 10 | 0.342 | 289 |
| 9 | 13,520,111 | 13,520,597 | 5 | -0.389 | 486 |
| 9 | 19,752,500 | 19,752,911 | 11 | 0.520 | 411 |
| 9 | 25,700,037 | 25,700,144 | 5 | -0.364 | 107 |
| 9 | 29,832,205 | 29,832,825 | 8 | 0.407 | 620 |
| 9 | 38,792,278 | 38,792,298 | 6 | 0.452 | 20 |
| 9 | 64,775,087 | 64,775,099 | 7 | 0.348 | 12 |
| 9 | 65,796,674 | 65,796,797 | 5 | 0.483 | 123 |
| 9 | 74,930,298 | 74,930,421 | 6 | 0.435 | 123 |
| 9 | 85,229,996 | 85,230,391 | 15 | -0.440 | 395 |
| 9 | 87,182,604 | 87,184,000 | 19 | -0.347 | 1396 |
| 9 | 102,523,054 | 102,523,254 | 5 | 0.343 | 200 |
| 9 | 107,966,795 | 107,967,231 | 7 | -0.337 | 436 |
| 9 | 108,248,635 | 108,248,647 | 6 | 0.404 | 12 |

| | | | | | |
|---|---|---|---|---|---|
| X | 2,783,483 | 2,783,759 | 6 | -0.394 | 276 |
| X | 5,747,985 | 5,748,050 | 5 | -0.401 | 65 |
| X | 12,801,245 | 12,801,257 | 5 | 0.743 | 12 |
| X | 23,068,502 | 23,068,709 | 9 | -0.344 | 207 |
| X | 26,492,702 | 26,492,853 | 5 | 0.344 | 151 |
| X | 42,894,473 | 42,894,597 | 5 | 0.378 | 124 |
| X | 57,083,752 | 57,084,010 | 5 | -0.459 | 258 |
| X | 90,977,361 | 90,977,561 | 7 | 0.359 | 200 |
| X | 117,037,932 | 117,038,068 | 5 | 0.406 | 136 |
| X | 143,652,989 | 143,653,051 | 6 | 0.363 | 62 |
| X | 153,574,856 | 153,575,048 | 7 | 0.401 | 192 |

5% between WKY and LEW rat strains that overlap with genes (n=420 genes). The overlappin

| | | | | **Overl** |
|---|---|---|---|---|
| *Average CpG density (SD)* | *Feature* | *Chr.* | *Start (bp)* | *End (bp)* |
| 0.021 (0.152) | downstream | 10 | 68,789,450 | 68,790,450 |
| 0.563 (0.089) | downstream | 9 | 65,577,305 | 65,578,305 |
| 0.036 (0.098) | downstream | 20 | 7,471,071 | 7,472,071 |
| 0.024 (0.114) | exon | 11 | 85,848,019 | 85,848,083 |
| 0.029 (0.096) | exon | 1 | 201,199,467 | 201,199,807 |
| 0.125 (0.074) | exon | 1 | 135,484,781 | 135,485,301 |
| 0.031 (0.112) | exon | 14 | 84,172,756 | 84,173,275 |
| 0.031 (0.112) | exon | 14 | 84,173,369 | 84,173,441 |
| 0.031 (0.112) | exon | 14 | 84,173,369 | 84,173,441 |
| 0.031 (0.112) | exon | 14 | 84,172,756 | 84,173,275 |
| 0.026 (0.2) | exon | 2 | 244,382,399 | 244,382,521 |
| 0.042 (0.128) | exon | 9 | 8,052,189 | 8,052,522 |
| 0.041 (0.104) | exon | 1 | 159,315,191 | 159,315,581 |
| 0.027 (0.089) | exon | 10 | 91,104,033 | 91,104,084 |
| 0.011 (0.179) | exon | 1 | 213,077,889 | 213,079,869 |
| 0.027 (0.087) | exon | 5 | 76,087,276 | 76,087,478 |
| 0.027 (0.087) | exon | 5 | 76,088,075 | 76,088,189 |
| 0.036 (0.09) | exon | 1 | 249,471,145 | 249,471,156 |
| 0.083 (0.098) | exon | 1 | 47,244,366 | 47,244,660 |
| 0.025 (0.087) | intron | 10 | 68,548,047 | 68,553,118 |
| 0.021 (0.152) | intron | 10 | 68,722,987 | 68,791,526 |
| 0.021 (0.145) | intron | 2 | 244,235,672 | 244,289,742 |
| 0.042 (0.118) | intron | 2 | 244,235,672 | 244,289,742 |
| 0.02 (0.124) | intron | 1 | 246,215,664 | 246,218,699 |
| 0.02 (0.124) | intron | 1 | 246,215,664 | 246,218,699 |
| 0.015 (0.079) | intron | 7 | 117,329,002 | 117,338,049 |
| 0.024 (0.114) | intron | 11 | 85,847,323 | 85,848,019 |
| 0.018 (0.171) | intron | 6 | 7,949,298 | 7,951,836 |
| 0.044 (0.162) | intron | 7 | 135,287,491 | 135,297,578 |
| 0.044 (0.162) | intron | 7 | 135,287,491 | 135,297,578 |
| 0.031 (0.112) | intron | 14 | 84,173,275 | 84,173,369 |
| 0.031 (0.112) | intron | 14 | 84,173,275 | 84,173,369 |
| 0.625 (0.056) | intron | 14 | 92,364,769 | 92,376,887 |

| | | | | |
|---|---|---|---|---|
| 0.045 (0.111) | intron | 18 | 71,444,017 | 71,455,837 |
| 0.045 (0.111) | intron | 18 | 71,444,017 | 71,455,837 |
| 0.045 (0.111) | intron | 18 | 71,444,017 | 71,455,837 |
| 0.02 (0.063) | intron | 5 | 155,543,388 | 155,605,323 |
| 0.013 (0.105) | intron | 5 | 155,440,926 | 155,449,302 |
| 0.114 (0.057) | intron | 5 | 155,440,926 | 155,449,302 |
| 0.023 (0.08) | intron | 4 | 117,437,900 | 117,451,617 |
| 0.023 (0.08) | intron | 4 | 117,437,900 | 117,451,617 |
| 0.024 (0.136) | intron | 4 | 48,325,959 | 48,358,013 |
| 0.024 (0.136) | intron | 4 | 48,325,959 | 48,358,013 |
| 0.024 (0.136) | intron | 4 | 48,325,959 | 48,358,013 |
| 0.014 (0.112) | intron | 8 | 11,383,950 | 11,392,901 |
| 0.014 (0.112) | intron | 8 | 11,383,950 | 11,392,901 |
| 0.026 (0.2) | intron | 2 | 244,380,689 | 244,382,399 |
| 0.042 (0.128) | intron | 9 | 8,020,548 | 8,052,189 |
| 0.058 (0.08) | intron | 1 | 190,987,715 | 191,006,715 |
| 0.03 (0.114) | intron | 13 | 44,600,786 | 44,602,200 |
| 0.03 (0.114) | intron | 13 | 44,600,786 | 44,602,200 |
| 0.026 (0.141) | intron | 5 | 153,335,732 | 153,338,124 |
| 0.021 (0.149) | intron | 5 | 153,342,063 | 153,348,585 |
| 0.021 (0.149) | intron | 5 | 153,342,063 | 153,348,585 |
| 0.026 (0.141) | intron | 5 | 153,335,706 | 153,338,040 |
| 0.049 (0.18) | intron | 8 | 75,990,972 | 76,013,957 |
| 0.049 (0.18) | intron | 8 | 75,990,972 | 76,013,957 |
| 0.041 (0.143) | intron | 1 | 250,082,269 | 250,087,870 |
| 0.563 (0.089) | intron | 9 | 65,553,730 | 65,578,381 |
| 0.563 (0.089) | intron | 9 | 65,553,730 | 65,578,381 |
| 0.563 (0.089) | intron | 9 | 65,553,730 | 65,578,381 |
| 0.027 (0.089) | intron | 10 | 91,104,084 | 91,105,285 |
| 0.026 (0.141) | intron | 2 | 182,060,632 | 182,061,216 |
| 0.026 (0.141) | intron | 2 | 182,060,632 | 182,061,216 |
| 0.027 (0.15) | intron | 6 | 115,268,772 | 115,328,727 |
| 0.043 (0.146) | intron | 5 | 70,549,289 | 70,560,923 |
| 0.043 (0.146) | intron | 5 | 70,549,289 | 70,560,923 |
| 0.027 (0.087) | intron | 5 | 76,087,478 | 76,088,075 |
| 0.036 (0.09) | intron | 1 | 249,471,156 | 249,471,388 |
| 0.036 (0.09) | intron | 1 | 249,470,656 | 249,471,145 |
| 0.036 (0.09) | intron | 1 | 249,470,667 | 249,471,388 |

| | | | | |
|---|---|---|---|---|
| 0.6 (0.061) | intron | 3 | 134,321,160 | 134,393,674 |
| 0.022 (0.118) | intron | 3 | 134,393,767 | 134,438,882 |
| 0.583 (0.051) | intron | 4 | 157,364,830 | 157,390,255 |
| 0.034 (0.113) | intron | 5 | 154,760,616 | 154,767,213 |
| 0.013 (0.078) | intron | 15 | 9,309,684 | 9,345,242 |
| 0.013 (0.078) | intron | 15 | 9,309,684 | 9,345,242 |
| 0.625 (0.049) | intron | 11 | 74,287,138 | 74,463,588 |
| 0.025 (0.054) | intron | 1 | 196,693,498 | 196,826,546 |
| 0.025 (0.054) | intron | 1 | 196,693,498 | 196,826,546 |
| 0.036 (0.189) | intron | 16 | 7,111,440 | 7,135,606 |
| 0.014 (0.114) | intron | 20 | 13,129,801 | 13,133,120 |
| 0.036 (0.098) | intron | 20 | 7,468,464 | 7,472,048 |
| 0.036 (0.098) | intron | 20 | 7,468,464 | 7,472,048 |
| 0.015 (0.069) | intron | 13 | 48,282,035 | 48,285,837 |
| 0.015 (0.069) | intron | 13 | 48,282,035 | 48,285,837 |
| 0.024 (0.114) | upstream | 11 | 85,847,310 | 85,848,310 |
| 0.024 (0.114) | upstream | 11 | 85,848,083 | 85,849,083 |
| 0.024 (0.114) | upstream | 11 | 85,847,307 | 85,848,307 |
| 0.029 (0.096) | upstream | 1 | 201,199,807 | 201,200,807 |
| 0.018 (0.195) | upstream | 5 | 160,518,285 | 160,519,285 |
| 0.035 (0.167) | upstream | 1 | 186,478,580 | 186,479,580 |
| 0.042 (0.118) | upstream | 2 | 244,284,602 | 244,285,602 |
| 0.027 (0.089) | upstream | 10 | 91,103,033 | 91,104,033 |
| 0.238 (0.1) | upstream | 5 | 76,203,391 | 76,204,391 |
| 0.041 (0.104) | 3'UTR | 1 | 159,315,191 | 159,315,300 |
| 0.011 (0.179) | 3'UTR | 1 | 213,078,112 | 213,079,869 |
| 0.024 (0.114) | 5'UTR | 11 | 85,848,019 | 85,848,083 |
| 0.029 (0.096) | 5'UTR | 1 | 201,199,716 | 201,199,807 |
| 0.027 (0.089) | 5'UTR | 10 | 91,104,033 | 91,104,041 |
| 0.024 (0.135) | 3'UTR | 10 | 45,286,929 | 45,286,982 |
| 0.024 (0.135) | 3'UTR | 10 | 45,286,929 | 45,286,982 |
| 0.024 (0.135) | exon | 10 | 45,286,929 | 45,287,160 |
| 0.024 (0.135) | downstream | 10 | 45,285,929 | 45,286,929 |
| 0.024 (0.135) | downstream | 10 | 45,285,929 | 45,286,929 |
| 0.024 (0.135) | exon | 10 | 45,286,929 | 45,287,160 |
| 0.024 (0.15) | intron | 8 | 85,307,103 | 85,311,432 |
| 0.024 (0.15) | intron | 8 | 85,307,103 | 85,311,432 |
| 0.022 (0.174) | intron | 8 | 126,279,014 | 126,282,878 |

| | | | | |
|---|---|---|---|---|
| 0.022 (0.174) | intron | 8 | 126,279,120 | 126,283,450 |
| 0.022 (0.174) | intron | 8 | 126,279,120 | 126,283,450 |
| 0.022 (0.174) | intron | 8 | 126,279,014 | 126,282,878 |
| 0.147 (0.168) | intron | 9 | 86,782,434 | 86,787,460 |
| 0.022 (0.099) | intron | 2 | 22,618,723 | 22,828,298 |
| 0.137 (0.122) | intron | 7 | 80,591,341 | 80,630,420 |
| 0.019 (0.117) | upstream | 19 | 50,423,220 | 50,424,220 |
| 0.046 (0.107) | intron | 3 | 33,804,788 | 33,805,745 |
| 0.021 (0.086) | intron | 8 | 59,693,483 | 59,719,851 |
| 0.069 (0.013) | intron | X | 12,543,816 | 12,544,015 |
| 0.069 (0.013) | exon | X | 12,544,015 | 12,544,142 |
| 0.313 (0.062) | intron | 7 | 31,692,197 | 31,751,845 |
| 0.048 (0.084) | intron | 13 | 66,815,875 | 66,877,144 |
| 0.048 (0.084) | intron | 13 | 66,815,875 | 66,877,144 |
| 0.019 (0.185) | intron | 3 | 134,607,698 | 134,634,443 |
| 0.019 (0.185) | intron | 3 | 134,620,247 | 134,627,108 |
| 0.019 (0.185) | intron | 3 | 134,607,698 | 134,634,443 |
| 0.042 (0.149) | intron | 1 | 253,034,913 | 253,036,034 |
| 0.042 (0.149) | intron | 1 | 253,034,913 | 253,036,034 |
| 0.015 (0.097) | intron | 8 | 113,746,748 | 113,769,247 |
| 0.114 (0.066) | intron | 16 | 82,837,706 | 82,839,028 |
| 0.114 (0.066) | intron | 16 | 82,837,706 | 82,839,028 |
| 0.024 (0.077) | intron | 1 | 41,149,867 | 41,153,367 |
| 0.055 (0.089) | exon | 7 | 140,097,985 | 140,098,111 |
| 0.055 (0.089) | intron | 7 | 140,097,522 | 140,097,985 |
| 0.055 (0.089) | exon | 7 | 140,097,985 | 140,098,111 |
| 0.055 (0.089) | intron | 7 | 140,097,522 | 140,097,985 |
| 0.023 (0.099) | intron | 11 | 68,726,257 | 68,742,527 |
| 0.023 (0.099) | intron | 11 | 68,726,257 | 68,742,527 |
| 0.023 (0.099) | intron | 11 | 68,726,257 | 68,742,527 |
| 0.01 (0.066) | intron | 4 | 87,155,045 | 87,169,017 |
| 0.01 (0.066) | intron | 4 | 87,155,045 | 87,169,017 |
| 0.011 (0.176) | intron | 2 | 144,945,936 | 145,010,043 |
| 0.011 (0.176) | intron | 2 | 144,945,936 | 145,010,043 |
| 0.208 (0.086) | intron | 4 | 184,203,011 | 184,212,697 |
| 0.021 (0.235) | intron | 1 | 171,700,961 | 171,772,539 |
| 0.045 (0.25) | intron | 1 | 171,891,689 | 171,997,453 |
| 0.375 (0.154) | intron | 14 | 81,650,298 | 81,650,828 |

| | | | | |
|---|---|---|---|---|
| 0.375 (0.154) | intron | 14 | 81,650,298 | 81,650,828 |
| 0.012 (0.103) | downstream | 18 | 78,989,890 | 78,990,890 |
| 0.012 (0.103) | intron | 18 | 78,989,888 | 79,025,405 |
| 0.011 (0.123) | intron | 1 | 8,649,950 | 8,688,527 |
| 0.034 (0.135) | intron | 1 | 8,649,950 | 8,688,527 |
| 0.039 (0.11) | intron | 1 | 8,649,950 | 8,688,527 |
| 0.125 (0.074) | 5'UTR | 1 | 135,485,114 | 135,485,459 |
| 0.125 (0.074) | upstream | 1 | 135,484,114 | 135,485,114 |
| 0.125 (0.074) | exon | 1 | 135,485,114 | 135,486,265 |
| 0.583 (0.003) | intron | X | 21,045,000 | 21,079,841 |
| 0.583 (0.003) | intron | X | 21,045,000 | 21,079,841 |
| 0.583 (0.003) | intron | X | 21,045,000 | 21,079,841 |
| 0.583 (0.003) | intron | X | 21,045,000 | 21,079,841 |
| 0.583 (0.003) | intron | X | 21,045,000 | 21,079,841 |
| 0.583 (0.003) | intron | X | 21,045,000 | 21,079,841 |
| 0.022 (0.108) | intron | 8 | 50,765,940 | 51,047,329 |
| 0.5 (0.088) | intron | 4 | 78,564,894 | 78,573,208 |
| 0.5 (0.088) | intron | 4 | 78,564,894 | 78,573,208 |
| 0.5 (0.088) | intron | 4 | 78,564,894 | 78,573,208 |
| 0.011 (0.129) | intron | 1 | 201,690,424 | 201,693,172 |
| 0.011 (0.129) | intron | 1 | 201,684,635 | 201,701,650 |
| 0.011 (0.129) | intron | 1 | 201,684,635 | 201,693,172 |
| 0.025 (0.101) | exon | 7 | 127,373,065 | 127,374,408 |
| 0.025 (0.101) | 3'UTR | 7 | 127,373,400 | 127,374,408 |
| 0.025 (0.101) | exon | 7 | 127,373,065 | 127,374,408 |
| 0.025 (0.101) | 3'UTR | 7 | 127,373,218 | 127,374,408 |
| 0.025 (0.101) | exon | 7 | 127,373,065 | 127,373,258 |
| 0.025 (0.101) | exon | 7 | 127,373,343 | 127,373,383 |
| 0.025 (0.101) | downstream | 7 | 127,373,383 | 127,374,383 |
| 0.025 (0.101) | intron | 7 | 127,373,258 | 127,373,343 |
| 0.018 (0.082) | intron | 1 | 259,905,837 | 260,092,911 |
| 0.018 (0.082) | intron | 1 | 259,905,837 | 260,092,911 |
| 0.018 (0.082) | intron | 1 | 259,905,837 | 260,092,911 |
| 0.053 (0.039) | intron | 1 | 13,376,038 | 13,559,232 |
| 0.047 (0.108) | intron | 1 | 13,376,038 | 13,559,232 |
| 0.031 (0.096) | intron | 6 | 99,471,678 | 99,480,585 |
| 0.015 (0.134) | intron | 12 | 35,454,179 | 35,458,050 |
| 0.167 (0.104) | intron | 19 | 57,248,885 | 57,274,659 |

| | | | | |
|---|---|---|---|---|
| 0.167 (0.104) | intron | 19 | 57,248,885 | 57,274,659 |
| 0.167 (0.104) | intron | 19 | 57,248,885 | 57,274,659 |
| 0.167 (0.104) | intron | 19 | 57,248,885 | 57,274,659 |
| 0.375 (0.022) | intron | 5 | 62,016,637 | 62,049,827 |
| 0.038 (0.112) | intron | 1 | 220,498,867 | 220,506,364 |
| 0.038 (0.112) | intron | 1 | 220,498,867 | 220,506,364 |
| 0.036 (0.161) | intron | 14 | 44,839,309 | 44,840,121 |
| 0.013 (0.115) | intron | 7 | 32,705,736 | 32,707,148 |
| 0.045 (0.095) | intron | 16 | 82,871,355 | 82,894,022 |
| 0.032 (0.163) | intron | 11 | 68,599,290 | 68,601,164 |
| 0.032 (0.163) | intron | 11 | 68,599,290 | 68,601,164 |
| 0.032 (0.163) | intron | 11 | 68,599,290 | 68,601,164 |
| 0.032 (0.163) | intron | 11 | 68,599,290 | 68,601,164 |
| 0.052 (0.138) | upstream | 3 | 10,211,694 | 10,212,694 |
| 0.046 (0.23) | intron | 8 | 77,850,725 | 77,858,939 |
| 0.25 (0.164) | intron | 9 | 86,651,188 | 86,655,112 |
| 0.25 (0.164) | intron | 9 | 86,651,188 | 86,655,112 |
| 0.571 (0.063) | intron | 6 | 23,977,510 | 23,980,967 |
| 0.015 (0.149) | intron | 16 | 47,596,609 | 47,600,596 |
| 0.015 (0.149) | exon | 16 | 47,600,596 | 47,600,727 |
| 0.015 (0.149) | intron | 16 | 47,600,727 | 47,602,781 |
| 0.6 (0.16) | intron | 19 | 49,629,613 | 49,691,466 |
| 0.011 (0.176) | upstream | 2 | 145,005,130 | 145,006,130 |
| 0.034 (0.133) | intron | 15 | 3,415,943 | 3,458,817 |
| 0.026 (0.093) | intron | 15 | 3,415,943 | 3,458,817 |
| 0.032 (0.112) | intron | 3 | 77,624,199 | 77,717,310 |
| 0.028 (0.05) | intron | 2 | 235,371,474 | 235,380,224 |
| 0.015 (0.109) | upstream | 20 | 9,688,660 | 9,689,660 |
| 0.036 (0.139) | intron | 1 | 168,045,108 | 168,089,032 |
| 0.029 (0.116) | intron | 12 | 12,535,900 | 12,567,849 |
| 0.455 (0.051) | upstream | 10 | 109,942,773 | 109,943,773 |
| 0.583 (0.128) | intron | 1 | 226,654,537 | 226,659,515 |
| 0.024 (0.169) | intron | 1 | 135,025,262 | 135,050,301 |
| 0.042 (0.14) | intron | 2 | 1,558,626 | 1,595,396 |
| 0.042 (0.14) | intron | 2 | 1,558,626 | 1,595,396 |
| 0.042 (0.14) | intron | 2 | 1,558,626 | 1,595,396 |
| 0.042 (0.14) | intron | 2 | 1,558,626 | 1,595,396 |
| 0.03 (0.079) | intron | 12 | 6,604,681 | 6,612,090 |

| | | | | |
|---|---|---|---|---|
| 0.01 (0.152) | intron | 3 | 24,734,751 | 24,810,331 |
| 0.054 (0.121) | exon | 15 | 53,811,021 | 53,811,212 |
| 0.089 (0.111) | intron | 8 | 114,012,992 | 114,019,690 |
| 0.089 (0.111) | intron | 8 | 114,012,992 | 114,019,690 |
| 0.089 (0.111) | intron | 8 | 114,012,992 | 114,019,690 |
| 0.056 (0.09) | 3'UTR | 6 | 109,561,707 | 109,562,001 |
| 0.056 (0.09) | exon | 6 | 109,561,065 | 109,562,001 |
| 0.096 (0.125) | intron | 1 | 248,664,435 | 248,665,342 |
| 0.096 (0.125) | intron | 1 | 248,664,435 | 248,665,342 |
| 0.022 (0.099) | intron | 2 | 22,687,096 | 22,701,683 |
| 0.027 (0.113) | intron | 3 | 2,645,870 | 2,647,269 |
| 0.027 (0.08) | intron | 5 | 57,620,719 | 57,621,598 |
| 0.027 (0.08) | intron | 5 | 57,620,719 | 57,621,598 |
| 0.027 (0.08) | intron | 5 | 57,620,719 | 57,621,598 |
| 0.027 (0.08) | intron | 5 | 57,620,719 | 57,621,598 |
| 0.026 (0.148) | intron | 17 | 64,157,087 | 64,204,634 |
| 0.026 (0.148) | intron | 17 | 64,157,087 | 64,204,634 |
| 0.625 (0.121) | intron | 5 | 128,442,772 | 128,446,879 |
| 0.625 (0.121) | intron | 5 | 128,442,975 | 128,446,879 |
| 0.625 (0.121) | intron | 5 | 128,442,772 | 128,446,879 |
| 0.046 (0.11) | intron | 4 | 174,510,179 | 174,517,020 |
| 0.145 (0.061) | intron | 4 | 174,376,532 | 174,488,913 |
| 0.049 (0.15) | intron | 7 | 138,463,656 | 138,464,406 |
| 0.049 (0.15) | exon | 7 | 138,463,549 | 138,463,656 |
| 0.013 (0.083) | downstream | 12 | 1,553,463 | 1,554,463 |
| 0.013 (0.195) | intron | 12 | 40,583,816 | 40,651,575 |
| 0.021 (0.125) | intron | 9 | 104,714,347 | 104,726,659 |
| 0.068 (0.121) | intron | 3 | 117,090,646 | 117,151,362 |
| 0.016 (0.2) | intron | 2 | 35,603,245 | 35,707,142 |
| 0.056 (0.116) | 3'UTR | 16 | 19,179,148 | 19,179,650 |
| 0.056 (0.116) | downstream | 16 | 19,179,650 | 19,180,650 |
| 0.056 (0.116) | exon | 16 | 19,178,940 | 19,179,650 |
| 0.012 (0.167) | intron | 12 | 9,797,694 | 9,798,800 |
| 0.021 (0.112) | intron | 20 | 11,616,869 | 11,678,343 |
| 0.031 (0.137) | intron | 5 | 159,780,898 | 159,782,540 |
| 0.011 (0.122) | intron | 6 | 123,589,623 | 123,653,535 |
| 0.01 (0.152) | intron | 2 | 140,584,549 | 140,738,868 |
| 0.01 (0.152) | intron | 2 | 140,584,549 | 140,738,868 |

| | | | | |
|---|---|---|---|---|
| 0.014 (0.188) | intron | X | 80,846,526 | 80,907,538 |
| 0.019 (0.096) | intron | 14 | 80,776,852 | 80,782,391 |
| 0.019 (0.096) | intron | 14 | 80,776,852 | 80,782,391 |
| 0.019 (0.096) | intron | 14 | 80,776,852 | 80,782,391 |
| 0.035 (0.142) | intron | 12 | 34,934,904 | 34,938,069 |
| 0.035 (0.142) | intron | 12 | 34,934,904 | 34,938,069 |
| 0.035 (0.142) | intron | 12 | 34,935,321 | 34,938,069 |
| 0.035 (0.142) | intron | 12 | 34,934,904 | 34,936,793 |
| 0.035 (0.142) | downstream | 12 | 34,935,563 | 34,936,563 |
| 0.625 (0.081) | intron | 16 | 81,090,223 | 81,098,542 |
| 0.262 (0.073) | intron | 1 | 78,480,949 | 78,494,441 |
| 0.5 (0.022) | downstream | 4 | 124,660,294 | 124,661,294 |
| 0.011 (0.157) | intron | 3 | 146,653,456 | 146,664,067 |
| 0.011 (0.157) | intron | 3 | 146,653,456 | 146,664,067 |
| 0.053 (0.107) | intron | 10 | 78,979,638 | 78,991,816 |
| 0.022 (0.113) | intron | 13 | 80,177,457 | 80,213,960 |
| 0.076 (0.127) | intron | 8 | 123,556,239 | 123,571,967 |
| 0.089 (0.127) | upstream | 8 | 51,883,785 | 51,884,785 |
| 0.025 (0.111) | intron | 10 | 65,218,672 | 65,246,003 |
| 0.025 (0.111) | intron | 10 | 65,218,672 | 65,246,003 |
| 0.025 (0.111) | intron | 10 | 65,218,672 | 65,246,003 |
| 0.039 (0.091) | intron | 5 | 77,924,410 | 77,977,885 |
| 0.013 (0.139) | intron | 5 | 77,924,410 | 77,977,885 |
| 0.039 (0.091) | intron | 5 | 77,924,410 | 77,977,885 |
| 0.013 (0.139) | intron | 5 | 77,924,410 | 77,977,885 |
| 0.167 (0.063) | intron | 18 | 72,565,723 | 72,584,152 |
| 0.069 (0.013) | intron | X | 12,515,257 | 12,551,212 |
| 0.069 (0.013) | intron | X | 12,515,257 | 12,551,212 |
| 0.01 (0.119) | intron | 10 | 65,609,143 | 65,613,695 |
| 0.01 (0.119) | intron | 10 | 65,609,143 | 65,613,695 |
| 0.01 (0.119) | intron | 10 | 65,609,143 | 65,613,695 |
| 0.01 (0.119) | intron | 10 | 65,609,143 | 65,613,695 |
| 0.6 (0.095) | intron | 1 | 189,755,119 | 189,788,495 |
| 0.6 (0.095) | intron | 1 | 189,755,119 | 189,788,495 |
| 0.6 (0.095) | intron | 1 | 189,755,119 | 189,788,495 |
| 0.6 (0.095) | intron | 1 | 189,755,119 | 189,788,495 |
| 0.069 (0.113) | exon | 10 | 90,070,934 | 90,071,771 |
| 0.069 (0.113) | 3'UTR | 10 | 90,071,028 | 90,071,771 |

| | | | | |
|---|---|---|---|---|
| 0.069 (0.113) | exon | 10 | 90,070,934 | 90,071,771 |
| 0.069 (0.113) | 3'UTR | 10 | 90,071,028 | 90,071,771 |
| 0.135 (0.083) | intron | 5 | 152,231,528 | 152,238,917 |
| 0.135 (0.083) | intron | 5 | 152,231,528 | 152,238,917 |
| 0.135 (0.083) | intron | 5 | 152,228,709 | 152,238,917 |
| 0.044 (0.143) | exon | 8 | 59,853,528 | 59,856,758 |
| 0.5 (0.092) | intron | 10 | 98,798,200 | 98,800,419 |
| 0.097 (0.176) | intron | 15 | 59,737,832 | 59,738,588 |
| 0.097 (0.176) | intron | 15 | 59,737,832 | 59,738,588 |
| 0.045 (0.152) | intron | 2 | 38,086,862 | 38,092,580 |
| 0.045 (0.152) | intron | 2 | 38,087,907 | 38,092,580 |
| 0.044 (0.158) | intron | 2 | 181,717,115 | 181,758,689 |
| 0.028 (0.164) | intron | 13 | 86,647,396 | 86,652,737 |
| 0.018 (0.074) | intron | 9 | 11,792,562 | 11,867,438 |
| 0.018 (0.074) | intron | 9 | 11,792,566 | 11,869,774 |
| 0.034 (0.155) | upstream | 3 | 18,997,796 | 18,998,796 |
| 0.048 (0.069) | intron | 18 | 72,095,827 | 72,126,368 |
| 0.048 (0.069) | intron | 18 | 72,106,414 | 72,126,368 |
| 0.014 (0.074) | intron | 17 | 10,912,765 | 10,928,325 |
| 0.022 (0.072) | intron | 9 | 73,660,984 | 73,691,525 |
| 0.022 (0.072) | intron | 9 | 73,613,804 | 73,691,525 |
| 0.022 (0.072) | intron | 9 | 73,612,584 | 73,691,525 |
| 0.059 (0.108) | exon | 1 | 85,260,167 | 85,260,559 |
| 0.059 (0.108) | intron | 1 | 85,258,954 | 85,260,167 |
| 0.026 (0.081) | intron | 5 | 130,481,767 | 130,511,046 |
| 0.026 (0.081) | intron | 5 | 130,481,767 | 130,511,046 |
| 0.026 (0.081) | intron | 5 | 130,481,767 | 130,511,046 |
| 0.583 (0.037) | intron | 4 | 131,857,923 | 131,862,992 |
| 0.011 (0.14) | intron | 5 | 157,130,908 | 157,143,798 |
| 0.041 (0.11) | intron | 14 | 5,368,591 | 5,393,619 |
| 0.121 (0.071) | intron | 3 | 106,191,114 | 106,208,447 |
| 0.121 (0.071) | intron | 3 | 106,191,114 | 106,208,447 |
| 0.121 (0.071) | intron | 3 | 106,191,114 | 106,208,447 |
| 0.02 (0.164) | intron | 9 | 38,627,575 | 38,629,960 |
| 0.128 (0.116) | exon | 13 | 91,565,218 | 91,567,831 |
| 0.128 (0.116) | 5'UTR | 13 | 91,565,218 | 91,565,286 |
| 0.233 (0.061) | exon | 1 | 78,860,378 | 78,860,966 |
| 0.011 (0.108) | intron | 9 | 63,527,488 | 63,536,577 |

| | | | | |
|---|---|---|---|---|
| 0.011 (0.108) | intron | 9 | 63,527,488 | 63,536,577 |
| 0.027 (0.124) | intron | 2 | 178,058,123 | 178,062,868 |
| 0.108 (0.081) | intron | 12 | 43,692,138 | 43,695,504 |
| 0.108 (0.081) | intron | 12 | 43,690,982 | 43,695,504 |
| 0.039 (0.129) | intron | 7 | 137,947,832 | 137,960,093 |
| 0.039 (0.129) | intron | 7 | 137,947,832 | 137,960,093 |
| 0.029 (0.12) | intron | 2 | 34,051,765 | 34,080,899 |
| 0.022 (0.091) | intron | 1 | 135,132,675 | 135,133,279 |
| 0.143 (0.159) | intron | 1 | 233,038,799 | 233,041,491 |
| 0.03 (0.153) | intron | 3 | 106,583,014 | 106,583,115 |
| 0.03 (0.153) | intron | 3 | 106,583,014 | 106,583,115 |
| 0.03 (0.153) | exon | 3 | 106,583,115 | 106,583,254 |
| 0.03 (0.153) | exon | 3 | 106,583,115 | 106,583,254 |
| 0.05 (0.067) | intron | 15 | 12,687,016 | 12,717,468 |
| 0.011 (0.153) | intron | 14 | 6,268,444 | 6,269,533 |
| 0.011 (0.153) | intron | 14 | 6,268,444 | 6,269,533 |
| 0.013 (0.189) | downstream | 9 | 6,207,477 | 6,208,477 |
| 0.067 (0.075) | exon | 2 | 180,479,631 | 180,479,834 |
| 0.067 (0.075) | intron | 2 | 180,479,834 | 180,479,909 |
| 0.067 (0.075) | exon | 2 | 180,479,909 | 180,480,036 |
| 0.02 (0.134) | intron | 10 | 109,149,622 | 109,175,964 |
| 0.026 (0.097) | intron | 18 | 66,139,400 | 66,193,405 |
| 0.026 (0.097) | intron | 18 | 66,139,400 | 66,193,405 |
| 0.027 (0.107) | intron | 17 | 20,916,166 | 20,918,403 |
| 0.027 (0.107) | exon | 17 | 20,918,403 | 20,918,490 |
| 0.036 (0.158) | intron | 4 | 171,010,238 | 171,098,607 |
| 0.036 (0.158) | intron | 4 | 171,010,238 | 171,098,607 |
| 0.035 (0.173) | intron | 3 | 146,234,573 | 146,238,306 |
| 0.019 (0.136) | exon | 3 | 156,326,776 | 156,326,867 |
| 0.019 (0.136) | intron | 3 | 156,326,867 | 156,328,358 |
| 0.019 (0.136) | 5'UTR | 3 | 156,326,776 | 156,326,867 |
| 0.019 (0.136) | exon | 3 | 156,326,776 | 156,326,867 |
| 0.019 (0.136) | intron | 3 | 156,326,867 | 156,328,358 |
| 0.019 (0.136) | 5'UTR | 3 | 156,326,776 | 156,326,867 |
| 0.01 (0.074) | intron | 2 | 61,695,707 | 61,697,098 |
| 0.01 (0.074) | exon | 2 | 61,695,576 | 61,695,707 |
| 0.048 (0.17) | intron | 7 | 131,747,848 | 131,760,879 |
| 0.048 (0.17) | intron | 7 | 131,747,848 | 131,760,879 |

| | | | | |
|---|---|---|---|---|
| 0.048 (0.17) | intron | 7 | 131,752,351 | 131,760,879 |
| 0.031 (0.179) | intron | 14 | 13,939,309 | 13,954,624 |
| 0.031 (0.179) | intron | 14 | 13,939,064 | 13,954,628 |
| 0.121 (0.071) | intron | 3 | 106,188,289 | 106,278,507 |
| 0.028 (0.099) | intron | 8 | 119,616,788 | 119,647,912 |
| 0.028 (0.099) | intron | 8 | 119,616,788 | 119,647,912 |
| 0.018 (0.075) | intron | 12 | 27,413,248 | 27,421,591 |
| 0.022 (0.107) | exon | 8 | 115,451,785 | 115,453,260 |
| 0.013 (0.13) | intron | 20 | 28,107,188 | 28,107,594 |
| 0.013 (0.13) | exon | 20 | 28,107,594 | 28,107,984 |
| 0.013 (0.13) | exon | 20 | 28,107,594 | 28,107,984 |
| 0.013 (0.13) | intron | 20 | 28,107,188 | 28,107,594 |
| 0.027 (0.14) | intron | 2 | 44,237,811 | 44,240,432 |
| 0.027 (0.14) | intron | 2 | 44,237,811 | 44,240,432 |
| 0.027 (0.14) | intron | 2 | 44,237,811 | 44,240,432 |
| 0.02 (0.18) | intron | 12 | 15,690,956 | 15,784,246 |
| 0.031 (0.128) | intron | 12 | 15,690,956 | 15,784,246 |
| 0.037 (0.154) | downstream | 12 | 15,789,813 | 15,790,813 |
| 0.037 (0.154) | downstream | 12 | 15,789,813 | 15,790,813 |
| 0.028 (0.135) | exon | 20 | 13,999,047 | 13,999,122 |
| 0.028 (0.135) | intron | 20 | 13,999,122 | 13,999,604 |
| 0.02 (0.1) | intron | 20 | 13,614,757 | 13,619,739 |
| 0.02 (0.1) | intron | 20 | 13,614,757 | 13,619,739 |
| 0.071 (0.149) | intron | 16 | 79,486,202 | 79,496,322 |
| 0.04 (0.115) | intron | 15 | 24,228,064 | 24,276,974 |
| 0.043 (0.108) | intron | 15 | 24,352,940 | 24,362,751 |
| 0.028 (0.07) | intron | 9 | 102,065,810 | 102,156,203 |
| 0.021 (0.118) | intron | 16 | 75,892,115 | 75,918,571 |
| 0.024 (0.134) | intron | 16 | 75,892,115 | 75,918,571 |
| 0.024 (0.074) | exon | 3 | 74,824,772 | 74,825,000 |
| 0.013 (0.102) | intron | 3 | 74,827,210 | 74,830,852 |
| 0.024 (0.074) | intron | 3 | 74,823,839 | 74,824,772 |
| 0.013 (0.102) | intron | 3 | 74,827,213 | 74,830,852 |
| 0.024 (0.074) | intron | 3 | 74,825,000 | 74,826,961 |
| 0.024 (0.074) | intron | 3 | 74,825,000 | 74,826,961 |
| 0.024 (0.074) | exon | 3 | 74,824,772 | 74,825,000 |
| 0.024 (0.074) | intron | 3 | 74,823,767 | 74,824,772 |
| 0.092 (0.075) | exon | 1 | 84,804,420 | 84,805,787 |

| | | | | |
|---|---|---|---|---|
| 0.08 (0.098) | intron | 1 | 26,631,354 | 26,694,449 |
| 0.01 (0.151) | intron | 6 | 110,357,060 | 110,369,744 |
| 0.01 (0.151) | exon | 6 | 110,369,744 | 110,369,809 |
| 0.025 (0.112) | intron | 16 | 81,395,464 | 81,404,438 |
| 0.025 (0.112) | intron | 16 | 81,395,464 | 81,404,438 |
| 0.025 (0.112) | intron | 16 | 81,395,464 | 81,404,438 |
| 0.025 (0.112) | intron | 16 | 81,395,464 | 81,404,438 |
| 0.026 (0.119) | intron | 4 | 9,571,584 | 9,574,378 |
| 0.026 (0.119) | intron | 4 | 9,571,584 | 9,574,378 |
| 0.019 (0.065) | intron | 2 | 9,682,119 | 9,687,370 |
| 0.019 (0.065) | intron | 2 | 9,682,119 | 9,687,370 |
| 0.023 (0.1) | upstream | 5 | 169,268,006 | 169,269,006 |
| 0.016 (0.192) | intron | 8 | 32,194,706 | 32,221,105 |
| 0.016 (0.192) | intron | 8 | 32,194,706 | 32,221,105 |
| 0.037 (0.236) | intron | 1 | 15,753,542 | 15,756,047 |
| 0.037 (0.236) | intron | 1 | 15,753,542 | 15,756,047 |
| 0.04 (0.102) | intron | 4 | 10,015,983 | 10,041,066 |
| 0.04 (0.102) | intron | 4 | 10,015,983 | 10,041,066 |
| 0.04 (0.102) | intron | 4 | 10,015,983 | 10,041,066 |
| 0.04 (0.102) | intron | 4 | 10,015,983 | 10,041,066 |
| 0.018 (0.17) | intron | 4 | 165,637,620 | 165,699,271 |
| 0.556 (0.007) | intron | 16 | 54,381,762 | 54,382,444 |
| 0.556 (0.007) | intron | 16 | 54,381,762 | 54,382,444 |
| 0.462 (0.066) | downstream | 10 | 82,762,795 | 82,763,795 |
| 0.462 (0.066) | downstream | 10 | 82,762,795 | 82,763,795 |
| 0.462 (0.066) | downstream | 10 | 82,762,795 | 82,763,795 |
| 0.462 (0.066) | downstream | 10 | 82,762,795 | 82,763,795 |
| 0.462 (0.066) | downstream | 10 | 82,762,795 | 82,763,795 |
| 0.462 (0.066) | downstream | 10 | 82,762,795 | 82,763,795 |
| 0.462 (0.066) | downstream | 10 | 82,762,795 | 82,763,795 |
| 0.012 (0.151) | intron | 19 | 52,193,737 | 52,213,642 |
| 0.012 (0.151) | intron | 19 | 52,193,737 | 52,213,642 |
| 0.012 (0.151) | intron | 19 | 52,193,758 | 52,213,642 |
| 0.031 (0.128) | intron | 12 | 15,756,528 | 15,767,821 |
| 0.016 (0.184) | intron | 18 | 3,756,080 | 3,777,647 |
| 0.018 (0.084) | intron | 4 | 125,978,279 | 126,001,652 |
| 0.018 (0.084) | intron | 4 | 125,978,279 | 126,001,652 |
| 0.018 (0.084) | intron | 4 | 125,978,279 | 126,001,652 |

| | | | | |
|---|---|---|---|---|
| 0.04 (0.081) | intron | 2 | 28,670,545 | 28,729,097 |
| 0.04 (0.081) | intron | 2 | 28,670,545 | 28,729,097 |
| 0.013 (0.15) | intron | 19 | 14,328,297 | 14,332,448 |
| 0.027 (0.124) | intron | 16 | 17,368,195 | 17,397,935 |
| 0.038 (0.035) | intron | 15 | 85,266,906 | 85,379,826 |
| 0.111 (0.096) | upstream | 10 | 105,373,626 | 105,374,626 |
| 0.111 (0.096) | upstream | 10 | 105,373,626 | 105,374,626 |
| 0.068 (0.13) | upstream | 4 | 119,369,308 | 119,370,308 |
| 0.03 (0.137) | intron | 12 | 39,445,327 | 39,449,931 |
| 0.074 (0.112) | intron | 1 | 149,750,764 | 149,751,978 |
| 0.038 (0.123) | intron | 14 | 6,356,219 | 6,356,418 |
| 0.038 (0.123) | exon | 14 | 6,356,101 | 6,356,219 |
| 0.038 (0.123) | exon | 14 | 6,356,418 | 6,356,540 |
| 0.059 (0.117) | intron | 14 | 6,356,540 | 6,359,542 |
| 0.571 (0.076) | intron | 2 | 196,340,660 | 196,345,693 |
| 0.571 (0.076) | intron | 2 | 196,340,660 | 196,345,693 |
| 0.031 (0.141) | intron | 14 | 17,225,123 | 17,280,553 |
| 0.571 (0.096) | downstream | 1 | 85,488,088 | 85,489,088 |
| 0.032 (0.171) | intron | 3 | 11,923,267 | 11,927,989 |
| 0.018 (0.167) | intron | 3 | 11,923,267 | 11,927,989 |
| 0.018 (0.167) | intron | 3 | 11,923,267 | 11,927,989 |
| 0.032 (0.171) | intron | 3 | 11,923,267 | 11,927,989 |
| 0.036 (0.127) | intron | 13 | 86,816,883 | 86,901,066 |
| 0.014 (0.112) | intron | 5 | 167,638,744 | 167,659,981 |
| 0.014 (0.112) | intron | 5 | 167,638,744 | 167,659,981 |
| 0.014 (0.112) | intron | 5 | 167,638,744 | 167,659,981 |
| 0.052 (0.123) | intron | 4 | 152,176,236 | 152,276,168 |
| 0.019 (0.13) | intron | 2 | 158,488,991 | 158,591,838 |
| 0.019 (0.13) | intron | 2 | 158,488,991 | 158,591,838 |
| 0.012 (0.148) | intron | 4 | 132,665,867 | 132,724,051 |
| 0.012 (0.148) | intron | 4 | 132,716,626 | 132,724,051 |
| 0.012 (0.148) | intron | 4 | 132,716,626 | 132,724,051 |
| 0.012 (0.148) | intron | 4 | 132,716,626 | 132,724,051 |
| 0.012 (0.148) | intron | 4 | 132,665,867 | 132,724,051 |
| 0.071 (0.121) | intron | 20 | 43,225,875 | 43,226,507 |
| 0.313 (0.148) | intron | 3 | 76,042,814 | 76,048,656 |
| 0.313 (0.148) | intron | 3 | 76,042,814 | 76,048,656 |
| 0.017 (0.097) | intron | 4 | 43,357,843 | 43,477,628 |

| | | | | |
|---|---|---|---|---|
| 0.017 (0.097) | intron | 4 | 43,357,843 | 43,477,628 |
| 0.017 (0.097) | intron | 4 | 43,357,843 | 43,477,628 |
| 0.012 (0.08) | intron | 5 | 126,183,176 | 126,195,248 |
| 0.625 (0.008) | intron | 9 | 86,839,028 | 86,869,591 |
| 0.022 (0.135) | intron | 19 | 50,097,754 | 50,111,592 |
| 0.6 (0.07) | intron | 9 | 60,772,757 | 60,785,544 |
| 0.6 (0.07) | intron | 9 | 60,772,757 | 60,785,544 |
| 0.114 (0.066) | downstream | 16 | 82,837,695 | 82,838,695 |
| 0.114 (0.066) | downstream | 16 | 82,837,695 | 82,838,695 |
| 0.043 (0.141) | intron | 13 | 81,707,854 | 81,796,317 |
| 0.016 (0.115) | intron | 3 | 104,871,163 | 104,873,356 |
| 0.016 (0.115) | intron | 3 | 104,871,163 | 104,873,356 |
| 0.021 (0.104) | intron | 11 | 79,435,065 | 79,438,311 |
| 0.028 (0.168) | intron | 5 | 158,287,483 | 158,437,690 |
| 0.028 (0.168) | intron | 5 | 158,287,483 | 158,437,690 |
| 0.023 (0.1) | upstream | 5 | 169,268,007 | 169,269,007 |
| 0.012 (0.162) | intron | 10 | 83,978,012 | 83,980,894 |
| 0.012 (0.162) | intron | 10 | 83,978,012 | 83,980,894 |
| 0.012 (0.162) | intron | 10 | 83,976,206 | 83,980,894 |
| 0.012 (0.162) | intron | 10 | 83,978,012 | 83,980,894 |
| 0.012 (0.162) | intron | 10 | 83,976,206 | 83,980,894 |
| 0.048 (0.102) | exon | 13 | 52,845,991 | 52,849,771 |
| 0.048 (0.102) | exon | 13 | 52,845,991 | 52,848,984 |
| 0.048 (0.102) | intron | 13 | 52,840,725 | 52,851,042 |
| 0.059 (0.108) | upstream | 1 | 85,260,147 | 85,261,147 |
| 0.059 (0.108) | upstream | 1 | 85,260,147 | 85,261,147 |
| 0.022 (0.13) | exon | 3 | 108,930,762 | 108,931,041 |
| 0.022 (0.13) | intron | 3 | 108,927,992 | 108,930,762 |
| 0.029 (0.113) | intron | 15 | 106,634,703 | 106,635,107 |
| 0.029 (0.113) | 3'UTR | 15 | 106,634,564 | 106,634,703 |
| 0.029 (0.113) | exon | 15 | 106,634,564 | 106,634,703 |
| 0.029 (0.113) | intron | 15 | 106,634,703 | 106,635,107 |
| 0.029 (0.113) | exon | 15 | 106,634,564 | 106,634,703 |
| 0.029 (0.113) | exon | 15 | 106,634,572 | 106,634,703 |
| 0.029 (0.113) | exon | 15 | 106,634,564 | 106,634,703 |
| 0.029 (0.113) | exon | 15 | 106,634,564 | 106,634,703 |
| 0.029 (0.113) | intron | 15 | 106,634,703 | 106,635,107 |
| 0.029 (0.113) | intron | 15 | 106,634,703 | 106,635,107 |

| | | | | |
|---|---|---|---|---|
| 0.029 (0.113) | intron | 15 | 106,634,703 | 106,635,107 |
| 0.029 (0.113) | intron | 15 | 106,634,703 | 106,635,107 |
| 0.029 (0.113) | exon | 15 | 106,634,564 | 106,634,703 |
| 0.104 (0.075) | intron | 2 | 43,092,302 | 43,124,987 |
| 0.104 (0.075) | intron | 2 | 43,092,302 | 43,124,987 |
| 0.018 (0.195) | downstream | 5 | 160,518,857 | 160,519,857 |
| 0.023 (0.079) | intron | 5 | 145,416,611 | 145,425,173 |
| 0.023 (0.079) | intron | 5 | 145,410,946 | 145,425,173 |
| 0.106 (0.097) | intron | 3 | 7,496,643 | 7,524,043 |
| 0.106 (0.097) | intron | 3 | 7,516,437 | 7,524,043 |
| 0.047 (0.108) | intron | 1 | 13,385,900 | 13,572,433 |
| 0.053 (0.039) | intron | 1 | 13,383,168 | 13,385,786 |
| 0.083 (0.165) | downstream | 9 | 105,784,268 | 105,785,268 |
| 0.083 (0.165) | downstream | 9 | 105,784,268 | 105,785,268 |
| 0.013 (0.134) | 3'UTR | 11 | 73,147,651 | 73,148,932 |
| 0.013 (0.134) | exon | 11 | 73,147,651 | 73,148,932 |
| 0.013 (0.134) | upstream | 11 | 73,148,932 | 73,149,932 |
| 0.455 (0.051) | intron | 10 | 109,849,240 | 110,020,353 |
| 0.583 (0.041) | upstream | 19 | 25,293,433 | 25,294,433 |
| 0.583 (0.041) | upstream | 19 | 25,293,433 | 25,294,433 |
| 0.583 (0.041) | upstream | 19 | 25,293,433 | 25,294,433 |
| 0.583 (0.041) | intron | 19 | 25,293,432 | 25,300,699 |
| 0.5 (0.105) | intron | 13 | 32,714,833 | 32,716,476 |
| 0.6 (0.16) | intron | 19 | 49,645,537 | 49,649,660 |
| 0.032 (0.182) | intron | 5 | 106,164,165 | 106,297,994 |
| 0.032 (0.182) | intron | 5 | 106,164,165 | 106,297,994 |
| 0.037 (0.113) | upstream | 8 | 48,907,693 | 48,908,693 |
| 0.015 (0.126) | intron | 4 | 9,276,573 | 9,285,502 |
| 0.021 (0.109) | intron | 12 | 7,885,613 | 7,904,181 |
| 0.013 (0.083) | upstream | 12 | 1,552,353 | 1,553,353 |
| 0.013 (0.083) | upstream | 12 | 1,552,353 | 1,553,353 |
| 0.019 (0.093) | intron | 8 | 127,592,040 | 127,593,372 |
| 0.012 (0.19) | intron | 1 | 190,108,640 | 190,109,663 |
| 0.012 (0.19) | intron | 1 | 190,108,640 | 190,109,663 |
| 0.012 (0.19) | exon | 1 | 190,109,663 | 190,109,778 |
| 0.012 (0.19) | exon | 1 | 190,109,663 | 190,109,778 |
| 0.6 (0.1) | intron | 19 | 27,908,780 | 27,922,589 |
| 0.6 (0.1) | intron | 19 | 27,908,780 | 27,922,589 |

| | | | | |
|---|---|---|---|---|
| 0.034 (0.074) | intron | 4 | 128,356,111 | 128,596,806 |
| 0.025 (0.133) | upstream | 9 | 93,099,368 | 93,100,368 |
| 0.056 (0.198) | intron | 5 | 51,219,898 | 51,223,453 |
| 0.016 (0.161) | intron | 20 | 43,586,897 | 43,642,495 |
| 0.016 (0.161) | intron | 20 | 43,586,897 | 43,642,495 |
| 0.147 (0.1) | exon | 20 | 13,116,298 | 13,116,709 |
| 0.076 (0.038) | intron | 13 | 67,544,405 | 67,578,397 |
| 0.076 (0.038) | intron | 13 | 67,544,405 | 67,578,397 |
| 0.014 (0.117) | intron | 18 | 3,687,839 | 3,692,202 |
| 0.625 (0.002) | intron | X | 55,966,136 | 55,968,717 |
| 0.017 (0.051) | upstream | 2 | 236,070,887 | 236,071,887 |
| 0.5 (0.177) | intron | 16 | 80,710,530 | 80,761,951 |
| 0.5 (0.177) | intron | 16 | 80,710,530 | 80,761,951 |
| 0.038 (0.16) | intron | 4 | 5,500,612 | 5,580,631 |
| 0.571 (0.102) | intron | 4 | 5,500,612 | 5,580,631 |
| 0.048 (0.073) | intron | 13 | 78,222,704 | 78,224,216 |
| 0.048 (0.073) | intron | 13 | 78,222,704 | 78,224,216 |
| 0.048 (0.073) | intron | 13 | 78,222,704 | 78,224,216 |
| 0.034 (0.084) | intron | 17 | 27,481,205 | 27,497,055 |
| 0.034 (0.084) | intron | 17 | 27,473,936 | 27,497,055 |
| 0.034 (0.084) | intron | 17 | 27,474,054 | 27,497,055 |
| 0.034 (0.084) | intron | 17 | 27,473,936 | 27,497,055 |
| 0.047 (0.124) | intron | 17 | 11,498,091 | 11,552,622 |
| 0.192 (0.067) | intron | 17 | 11,630,315 | 11,736,990 |
| 0.047 (0.124) | intron | 17 | 11,498,091 | 11,552,622 |
| 0.047 (0.124) | intron | 17 | 11,498,091 | 11,552,622 |
| 0.192 (0.067) | intron | 17 | 11,630,315 | 11,736,990 |
| 0.192 (0.067) | intron | 17 | 11,630,315 | 11,736,990 |
| 0.192 (0.067) | intron | 17 | 11,681,664 | 11,736,990 |
| 0.5 (0.157) | intron | 1 | 67,874,609 | 67,874,759 |
| 0.084 (0.033) | exon | 4 | 156,324,272 | 156,324,435 |
| 0.084 (0.033) | exon | 4 | 156,324,272 | 156,324,435 |
| 0.084 (0.033) | exon | 4 | 156,324,272 | 156,324,435 |
| 0.039 (0.159) | exon | 3 | 58,744,323 | 58,744,470 |
| 0.039 (0.159) | exon | 3 | 58,744,323 | 58,744,470 |
| 0.039 (0.159) | exon | 3 | 58,744,323 | 58,744,470 |
| 0.039 (0.159) | exon | 3 | 58,744,323 | 58,744,470 |
| 0.039 (0.159) | exon | 3 | 58,744,323 | 58,744,470 |

| | | | | |
|---|---|---|---|---|
| 0.039 (0.159) | intron | 3 | 58,744,470 | 58,785,045 |
| 0.043 (0.156) | intron | 3 | 58,810,635 | 58,811,530 |
| 0.039 (0.159) | intron | 3 | 58,744,470 | 58,785,045 |
| 0.039 (0.159) | intron | 3 | 58,744,470 | 58,785,045 |
| 0.043 (0.156) | intron | 3 | 58,810,635 | 58,811,530 |
| 0.043 (0.156) | intron | 3 | 58,785,096 | 58,811,530 |
| 0.043 (0.156) | intron | 3 | 58,810,635 | 58,811,530 |
| 0.043 (0.156) | intron | 3 | 58,810,635 | 58,811,530 |
| 0.039 (0.159) | intron | 3 | 58,744,470 | 58,785,045 |
| 0.039 (0.159) | intron | 3 | 58,744,470 | 58,785,045 |
| 0.012 (0.161) | intron | 10 | 91,035,059 | 91,039,974 |
| 0.086 (0.13) | intron | 17 | 74,844,313 | 74,844,601 |
| 0.086 (0.13) | intron | 17 | 74,844,313 | 74,846,966 |
| 0.086 (0.13) | intron | 17 | 74,844,313 | 74,845,291 |
| 0.029 (0.107) | exon | 7 | 140,328,789 | 140,329,260 |
| 0.029 (0.107) | upstream | 7 | 140,329,260 | 140,330,260 |
| 0.023 (0.144) | intron | 1 | 4,481,813 | 4,485,520 |
| 0.023 (0.144) | intron | 1 | 4,481,813 | 4,485,520 |
| 0.023 (0.144) | intron | 1 | 4,481,813 | 4,485,520 |
| 0.027 (0.072) | intron | 9 | 53,272,498 | 53,273,627 |
| 0.013 (0.123) | intron | 9 | 102,649,335 | 102,686,954 |
| 0.013 (0.123) | intron | 9 | 102,649,335 | 102,686,954 |
| 0.053 (0.108) | upstream | 9 | 102,644,928 | 102,645,928 |
| 0.013 (0.123) | intron | 9 | 102,649,610 | 102,665,146 |
| 0.053 (0.108) | intron | 9 | 102,642,627 | 102,649,145 |
| 0.053 (0.108) | intron | 9 | 102,642,627 | 102,649,145 |
| 0.016 (0.127) | upstream | 1 | 72,948,877 | 72,949,877 |
| 0.016 (0.127) | upstream | 1 | 72,948,877 | 72,949,877 |
| 0.016 (0.127) | exon | 1 | 72,948,535 | 72,948,877 |
| 0.016 (0.127) | exon | 1 | 72,948,535 | 72,948,877 |
| 0.5 (0.142) | intron | 14 | 85,663,902 | 85,666,933 |
| 0.5 (0.142) | intron | 14 | 85,663,902 | 85,666,933 |
| 0.5 (0.142) | intron | 14 | 85,663,902 | 85,666,933 |
| 0.143 (0.153) | intron | 3 | 8,370,842 | 8,426,807 |
| 0.046 (0.134) | intron | 5 | 168,164,792 | 168,168,799 |
| 0.046 (0.134) | intron | 5 | 168,164,792 | 168,168,799 |
| 0.061 (0.09) | intron | 5 | 168,164,792 | 168,168,799 |
| 0.061 (0.09) | intron | 5 | 168,164,792 | 168,168,799 |

| | | | | |
|---|---|---|---|---|
| 0.013 (0.155) | intron | 1 | 253,196,383 | 253,202,466 |
| 0.03 (0.037) | intron | 9 | 46,494,201 | 46,497,029 |
| 0.03 (0.037) | intron | 9 | 46,494,201 | 46,496,964 |
| 0.031 (0.062) | intron | 5 | 156,684,154 | 156,691,135 |
| 0.031 (0.062) | intron | 5 | 156,684,154 | 156,691,135 |
| 0.069 (0.148) | intron | 4 | 182,691,556 | 182,725,790 |
| 0.032 (0.116) | intron | 4 | 182,691,556 | 182,692,779 |
| 0.032 (0.116) | intron | 4 | 182,691,556 | 182,725,790 |
| 0.069 (0.148) | intron | 4 | 182,691,556 | 182,692,779 |
| 0.017 (0.146) | intron | 5 | 157,980,961 | 158,028,775 |
| 0.017 (0.146) | intron | 5 | 157,980,961 | 158,028,775 |
| 0.017 (0.146) | intron | 5 | 157,980,961 | 158,028,775 |
| 0.022 (0.17) | downstream | 5 | 145,408,365 | 145,409,365 |
| 0.031 (0.079) | intron | 9 | 39,461,535 | 39,462,297 |
| 0.031 (0.079) | intron | 9 | 39,461,535 | 39,462,297 |
| 0.031 (0.079) | intron | 9 | 39,461,535 | 39,462,297 |
| 0.041 (0.148) | intron | 4 | 183,804,324 | 183,806,886 |
| 0.041 (0.148) | intron | 4 | 183,804,324 | 183,806,886 |
| 0.041 (0.148) | intron | 4 | 183,804,324 | 183,806,886 |
| 0.041 (0.148) | intron | 4 | 183,804,324 | 183,806,886 |
| 0.036 (0.123) | intron | 14 | 110,243,555 | 110,244,792 |
| 0.036 (0.123) | exon | 14 | 110,244,792 | 110,244,853 |
| 0.036 (0.123) | exon | 14 | 110,244,857 | 110,244,875 |
| 0.036 (0.123) | intron | 14 | 110,243,555 | 110,245,294 |
| 0.036 (0.123) | intron | 14 | 110,244,853 | 110,244,857 |
| 0.036 (0.123) | upstream | 14 | 110,244,875 | 110,245,875 |
| 0.179 (0.087) | intron | 20 | 24,636,640 | 24,686,081 |
| 0.061 (0.096) | intron | 12 | 41,330,784 | 41,337,390 |
| 0.089 (0.106) | intron | 20 | 9,887,131 | 9,891,454 |
| 0.015 (0.162) | intron | 20 | 9,822,887 | 9,866,651 |
| 0.056 (0.178) | intron | 20 | 12,967,638 | 12,984,417 |
| 0.022 (0.153) | downstream | 11 | 37,766,795 | 37,767,795 |
| 0.214 (0.151) | intron | 11 | 35,041,368 | 35,115,579 |
| 0.214 (0.151) | intron | 11 | 35,041,368 | 35,115,579 |
| 0.583 (0.1) | intron | 10 | 108,922,366 | 108,923,036 |
| 0.029 (0.098) | intron | 15 | 108,786,762 | 108,905,107 |
| 0.029 (0.098) | intron | 15 | 108,786,762 | 108,905,107 |
| 0.022 (0.091) | intron | 6 | 48,071,965 | 48,078,157 |

| | | | | |
|---|---|---|---|---|
| 0.022 (0.091) | intron | 6 | 48,071,965 | 48,078,157 |
| 0.014 (0.152) | intron | 3 | 47,275,915 | 47,279,666 |
| 0.024 (0.115) | intron | 3 | 47,280,824 | 47,294,338 |
| 0.014 (0.152) | intron | 3 | 47,275,915 | 47,279,666 |
| 0.014 (0.152) | intron | 3 | 47,275,915 | 47,279,666 |
| 0.024 (0.115) | intron | 3 | 47,280,824 | 47,294,338 |
| 0.024 (0.115) | intron | 3 | 47,280,824 | 47,294,338 |
| 0.018 (0.051) | 3'UTR | 6 | 54,651,838 | 54,652,276 |
| 0.018 (0.051) | downstream | 6 | 54,652,276 | 54,653,276 |
| 0.018 (0.051) | exon | 6 | 54,651,788 | 54,652,276 |
| 0.066 (0.087) | intron | 6 | 145,244,691 | 145,261,934 |
| 0.027 (0.062) | intron | 7 | 115,930,383 | 116,037,650 |
| 0.027 (0.178) | intron | 5 | 163,939,195 | 164,082,629 |
| 0.027 (0.178) | intron | 5 | 163,939,195 | 164,082,629 |
| 0.038 (0.113) | intron | 13 | 44,538,198 | 44,546,898 |
| 0.038 (0.113) | exon | 13 | 44,536,741 | 44,538,198 |
| 0.037 (0.11) | intron | 8 | 1,269,573 | 1,435,970 |
| 0.037 (0.11) | intron | 8 | 1,269,573 | 1,435,970 |
| 0.037 (0.11) | intron | 8 | 1,269,573 | 1,435,970 |
| 0.6 (0.146) | intron | 3 | 163,749,510 | 163,773,319 |
| 0.068 (0.055) | downstream | 13 | 48,364,610 | 48,365,610 |
| 0.031 (0.084) | intron | 5 | 66,066,568 | 66,083,419 |
| 0.206 (0.084) | intron | 4 | 43,687,337 | 43,689,437 |
| 0.039 (0.128) | intron | 10 | 20,788,615 | 20,795,295 |
| 0.179 (0.053) | intron | 15 | 14,202,559 | 14,214,979 |
| 0.179 (0.053) | intron | 15 | 14,199,300 | 14,214,979 |
| 0.179 (0.053) | intron | 15 | 14,202,559 | 14,214,979 |
| 0.179 (0.053) | intron | 15 | 14,201,671 | 14,214,979 |
| 0.052 (0.138) | intron | 3 | 10,165,847 | 10,238,819 |
| 0.625 (0.123) | intron | 4 | 172,835,454 | 172,914,497 |
| 0.032 (0.033) | intron | 2 | 69,651,452 | 69,672,089 |
| 0.036 (0.096) | intron | 4 | 62,698,741 | 62,701,567 |
| 0.036 (0.096) | intron | 4 | 62,698,741 | 62,701,567 |
| 0.018 (0.063) | intron | 15 | 106,213,318 | 106,225,444 |
| 0.04 (0.083) | intron | 15 | 106,213,318 | 106,225,444 |
| 0.017 (0.095) | intron | 10 | 86,278,079 | 86,279,208 |
| 0.017 (0.095) | intron | 10 | 86,278,079 | 86,279,208 |
| 0.017 (0.095) | intron | 10 | 86,278,079 | 86,279,208 |

| | | | | |
|---|---|---|---|---|
| 0.017 (0.095) | intron | 10 | 86,278,079 | 86,279,208 |
| 0.024 (0.148) | intron | 17 | 13,997,649 | 14,000,874 |
| 0.024 (0.148) | intron | 17 | 13,997,649 | 14,000,696 |
| 0.571 (0.072) | intron | 19 | 31,582,511 | 31,627,694 |
| 0.061 (0.1) | intron | 4 | 8,962,287 | 8,966,958 |
| 0.035 (0.063) | intron | 4 | 8,967,114 | 8,973,526 |
| 0.05 (0.061) | intron | 3 | 161,406,089 | 161,418,720 |
| 0.05 (0.061) | intron | 3 | 161,399,780 | 161,418,720 |
| 0.05 (0.061) | intron | 3 | 161,406,089 | 161,413,562 |
| 0.05 (0.061) | intron | 3 | 161,406,089 | 161,413,562 |
| 0.05 (0.061) | intron | 3 | 161,399,780 | 161,428,828 |
| 0.011 (0.101) | intron | 16 | 49,778,862 | 49,785,500 |
| 0.011 (0.101) | intron | 16 | 49,778,862 | 49,785,500 |
| 0.011 (0.101) | intron | 16 | 49,778,862 | 49,785,500 |
| 0.011 (0.101) | intron | 16 | 49,778,862 | 49,785,500 |
| 0.011 (0.101) | intron | 16 | 49,778,862 | 49,785,500 |
| 0.011 (0.101) | intron | 16 | 49,778,862 | 49,785,500 |
| 0.011 (0.101) | intron | 16 | 49,778,862 | 49,785,500 |
| 0.023 (0.18) | intron | 18 | 14,586,920 | 14,618,341 |
| 0.015 (0.225) | intron | 1 | 155,723,968 | 155,725,640 |
| 0.015 (0.225) | intron | 1 | 155,723,968 | 155,725,640 |
| 0.125 (0.07) | exon | 8 | 58,788,000 | 58,788,147 |
| 0.064 (0.111) | intron | 18 | 6,449,624 | 6,628,189 |
| 0.03 (0.142) | intron | 1 | 189,565,033 | 189,584,733 |
| 0.03 (0.142) | intron | 1 | 189,565,033 | 189,584,733 |
| 0.03 (0.142) | intron | 1 | 189,565,033 | 189,584,733 |
| 0.03 (0.142) | intron | 1 | 189,550,452 | 189,584,733 |
| 0.03 (0.142) | intron | 1 | 189,565,033 | 189,584,733 |
| 0.03 (0.142) | intron | 1 | 189,563,712 | 189,584,733 |
| 0.022 (0.091) | intron | 9 | 71,642,183 | 71,735,472 |
| 0.047 (0.156) | intron | 16 | 83,851,374 | 83,878,030 |
| 0.047 (0.156) | intron | 16 | 83,851,374 | 83,878,030 |
| 0.047 (0.156) | intron | 16 | 83,851,374 | 83,878,030 |
| 0.047 (0.107) | intron | 6 | 12,632,510 | 12,637,003 |
| 0.047 (0.107) | exon | 6 | 12,637,003 | 12,637,189 |
| 0.047 (0.107) | exon | 6 | 12,637,003 | 12,637,189 |
| 0.047 (0.107) | intron | 6 | 12,632,510 | 12,637,003 |
| 0.047 (0.107) | exon | 6 | 12,637,003 | 12,637,189 |

| | | | | |
|---|---|---|---|---|
| 0.047 (0.107) | intron | 6 | 12,636,224 | 12,637,003 |
| 0.047 (0.107) | intron | 6 | 12,637,189 | 12,639,690 |
| 0.047 (0.107) | intron | 6 | 12,637,189 | 12,639,690 |
| 0.047 (0.107) | intron | 6 | 12,632,510 | 12,639,690 |
| 0.047 (0.107) | intron | 6 | 12,637,189 | 12,639,690 |
| 0.025 (0.154) | intron | 6 | 12,865,526 | 12,925,920 |
| 0.025 (0.154) | intron | 6 | 12,865,526 | 12,925,920 |
| 0.01 (0.187) | intron | 18 | 4,285,134 | 4,291,057 |
| 0.055 (0.092) | intron | 2 | 30,890,355 | 30,891,147 |
| 0.055 (0.092) | exon | 2 | 30,889,816 | 30,890,355 |
| 0.042 (0.071) | intron | 5 | 168,286,088 | 168,291,305 |
| 0.042 (0.071) | intron | 5 | 168,286,088 | 168,291,305 |
| 0.042 (0.071) | intron | 5 | 168,286,088 | 168,291,305 |
| 0.02 (0.141) | intron | 19 | 11,071,931 | 11,078,711 |
| 0.028 (0.068) | intron | 5 | 158,594,087 | 158,643,754 |
| 0.015 (0.162) | intron | 1 | 164,722,312 | 164,772,757 |
| 0.083 (0.117) | exon | 9 | 74,829,625 | 74,829,810 |
| 0.083 (0.117) | exon | 9 | 74,829,625 | 74,829,810 |
| 0.083 (0.117) | exon | 9 | 74,829,625 | 74,829,810 |
| 0.022 (0.146) | intron | 1 | 203,383,855 | 203,426,071 |
| 0.022 (0.146) | intron | 1 | 203,383,721 | 203,459,580 |
| 0.019 (0.108) | upstream | 2 | 190,762,376 | 190,763,376 |
| 0.014 (0.117) | intron | 2 | 121,630,327 | 121,679,188 |
| 0.583 (0.03) | intron | 13 | 90,933,659 | 91,210,244 |
| 0.128 (0.156) | intron | 13 | 90,933,659 | 91,210,244 |
| 0.5 (0.042) | intron | 7 | 55,782,260 | 55,806,767 |
| 0.027 (0.126) | intron | 3 | 89,254,471 | 89,380,782 |
| 0.025 (0.121) | intron | 11 | 73,333,482 | 73,363,854 |
| 0.031 (0.073) | downstream | 13 | 44,919,902 | 44,920,902 |
| 0.031 (0.073) | intron | 13 | 44,920,958 | 44,922,588 |
| 0.031 (0.073) | intron | 13 | 44,910,268 | 44,922,588 |
| 0.031 (0.073) | exon | 13 | 44,920,902 | 44,920,958 |
| 0.031 (0.073) | downstream | 13 | 44,919,902 | 44,920,902 |
| 0.031 (0.073) | exon | 13 | 44,920,902 | 44,920,958 |
| 0.031 (0.073) | intron | 13 | 44,920,958 | 44,922,588 |
| 0.571 (0.096) | intron | 1 | 85,487,946 | 85,488,827 |
| 0.032 (0.179) | exon | 16 | 71,907,118 | 71,907,214 |
| 0.032 (0.179) | intron | 16 | 71,907,214 | 71,912,273 |

| | | | | |
|---|---|---|---|---|
| 0.013 (0.089) | upstream | 17 | 15,895,367 | 15,896,367 |
| 0.045 (0.061) | intron | 3 | 40,778,183 | 40,856,713 |
| 0.041 (0.087) | 5'UTR | 3 | 165,213,540 | 165,213,573 |
| 0.041 (0.087) | intron | 3 | 165,213,573 | 165,213,665 |
| 0.041 (0.087) | upstream | 3 | 165,212,540 | 165,213,540 |
| 0.041 (0.087) | exon | 3 | 165,213,540 | 165,213,573 |
| 0.026 (0.118) | intron | 19 | 53,188,864 | 53,190,417 |
| 0.068 (0.093) | intron | 2 | 252,633,539 | 252,672,255 |
| 0.021 (0.109) | upstream | 12 | 7,891,407 | 7,892,407 |
| 0.159 (0.007) | intron | 12 | 40,151,228 | 40,225,769 |
| 0.6 (0.043) | intron | 2 | 179,088,913 | 179,227,647 |
| 0.039 (0.148) | intron | 7 | 138,827,694 | 138,847,150 |
| 0.02 (0.15) | intron | 7 | 138,688,918 | 138,827,484 |
| 0.035 (0.109) | intron | 8 | 118,909,081 | 118,919,789 |
| 0.5 (0.023) | intron | 4 | 3,235,088 | 3,344,872 |
| 0.5 (0.023) | intron | 4 | 3,235,088 | 3,565,245 |
| 0.098 (0.019) | intron | 15 | 79,582,819 | 79,771,222 |
| 0.098 (0.019) | intron | 15 | 79,582,819 | 79,771,222 |
| 0.017 (0.127) | intron | 16 | 71,866,438 | 71,870,250 |
| 0.022 (0.18) | intron | 8 | 111,017,958 | 111,019,312 |
| 0.022 (0.18) | upstream | 8 | 111,018,335 | 111,019,335 |
| 0.6 (0.017) | intron | 4 | 76,734,402 | 76,857,149 |
| 0.026 (0.165) | exon | 7 | 1,055,388 | 1,055,442 |
| 0.026 (0.165) | intron | 7 | 1,055,376 | 1,055,388 |
| 0.026 (0.165) | exon | 7 | 1,054,484 | 1,054,649 |
| 0.026 (0.165) | intron | 7 | 1,054,649 | 1,055,208 |
| 0.026 (0.165) | exon | 7 | 1,055,208 | 1,055,376 |
| 0.04 (0.133) | intron | 8 | 115,861,900 | 115,876,541 |
| 0.01 (0.11) | exon | 2 | 201,430,495 | 201,430,637 |
| 0.01 (0.11) | intron | 2 | 201,422,022 | 201,430,289 |
| 0.01 (0.11) | exon | 2 | 201,430,289 | 201,430,409 |
| 0.01 (0.11) | intron | 2 | 201,430,409 | 201,430,495 |
| 0.049 (0.051) | intron | 2 | 201,387,321 | 201,401,002 |
| 0.042 (0.15) | upstream | 4 | 78,807,400 | 78,808,400 |
| 0.041 (0.112) | exon | 11 | 37,730,168 | 37,730,235 |
| 0.041 (0.112) | intron | 11 | 37,730,164 | 37,730,168 |
| 0.041 (0.112) | exon | 11 | 37,730,260 | 37,730,343 |
| 0.041 (0.112) | intron | 11 | 37,730,235 | 37,730,260 |

| | | | | |
|---|---|---|---|---|
| 0.041 (0.112) | upstream | 11 | 37,730,343 | 37,731,343 |
| 0.041 (0.112) | exon | 11 | 37,730,113 | 37,730,164 |
| 0.074 (0.135) | intron | 9 | 101,293,218 | 101,532,533 |
| 0.027 (0.04) | upstream | 5 | 80,597,573 | 80,598,573 |
| 0.051 (0.122) | upstream | 1 | 85,504,431 | 85,505,431 |
| 0.051 (0.122) | exon | 1 | 85,505,431 | 85,505,538 |
| 0.051 (0.122) | 5'UTR | 1 | 85,505,431 | 85,505,538 |
| 0.027 (0.062) | upstream | 7 | 115,976,941 | 115,977,941 |
| 0.022 (0.047) | intron | 2 | 230,675,795 | 230,697,881 |
| 0.046 (0.045) | intron | 10 | 67,049,926 | 67,065,480 |
| 0.135 (0.083) | intron | 5 | 152,218,302 | 152,379,095 |
| 0.01 (0.212) | intron | 2 | 189,024,362 | 189,052,866 |
| 0.014 (0.075) | intron | 2 | 188,861,572 | 189,024,297 |
| 0.014 (0.075) | intron | 2 | 188,861,572 | 189,024,297 |
| 0.01 (0.212) | intron | 2 | 189,024,362 | 189,052,866 |
| 0.086 (0.117) | intron | 1 | 90,861,908 | 90,861,910 |
| 0.086 (0.117) | exon | 1 | 90,861,683 | 90,861,908 |
| 0.086 (0.117) | exon | 1 | 90,861,910 | 90,862,006 |
| 0.108 (0.086) | 3'UTR | 10 | 45,481,617 | 45,482,421 |
| 0.108 (0.086) | exon | 10 | 45,481,617 | 45,482,421 |
| 0.03 (0.037) | intron | 9 | 46,467,172 | 46,654,691 |
| 0.185 (0.046) | intron | 19 | 29,168,011 | 29,191,275 |
| 0.015 (0.069) | intron | 13 | 48,146,379 | 48,292,609 |
| 0.02 (0.114) | intron | 16 | 10,475,925 | 10,602,541 |
| 0.035 (0.2) | intron | 17 | 12,811,981 | 12,854,073 |
| 0.017 (0.145) | intron | 3 | 3,893,296 | 3,893,837 |
| 0.03 (0.095) | intron | 3 | 430,993 | 579,427 |
| 0.043 (0.141) | upstream | 13 | 81,795,197 | 81,796,197 |
| 0.017 (0.09) | downstream | 1 | 165,836,402 | 165,837,402 |
| 0.036 (0.009) | downstream | 2 | 613,895 | 614,895 |
| 0.032 (0.125) | downstream | 20 | 10,204,247 | 10,205,247 |
| 0.014 (0.075) | intron | 2 | 188,861,069 | 189,028,299 |
| 0.01 (0.212) | intron | 2 | 189,028,334 | 189,088,428 |
| 0.016 (0.127) | intron | 1 | 72,661,541 | 72,987,734 |
| 0.016 (0.127) | intron | 1 | 72,824,787 | 72,987,777 |
| 0.016 (0.127) | intron | 1 | 72,932,853 | 72,959,825 |
| 0.027 (0.116) | intron | 2 | 193,306,924 | 193,314,220 |
| 0.061 (0.1) | intron | 4 | 8,943,021 | 8,977,746 |

| | | | | |
|---|---|---|---|---|
| 0.035 (0.063) | intron | 4 | 8,943,021 | 8,977,746 |
| 0.028 (0.127) | downstream | 17 | 93,271,782 | 93,272,782 |
| 0.061 (0.112) | downstream | 20 | 12,371,390 | 12,372,390 |
| 0.135 (0.083) | upstream | 5 | 152,235,563 | 152,236,563 |
| 0.223 (0.045) | N/A | | | |
| 0.122 (0.031) | N/A | | | |
| 0.058 (0.138) | N/A | | | |
| 0.064 (0.125) | N/A | | | |
| 0.170 (0.018) | N/A | | | |
| 0.073 (0.05) | N/A | | | |
| 0.121 (0.044) | N/A | | | |
| 0.112 (0.023) | N/A | | | |
| 0.003 (0.032) | N/A | | | |
| 0.085 (0.015) | N/A | | | |
| 0.154 (0.023) | N/A | | | |
| 0.140 (0.061) | N/A | | | |
| 0.093 (0.039) | N/A | | | |
| 0.152 (0.011) | N/A | | | |
| 0.076 (0.035) | N/A | | | |
| 0.103 (0.020) | N/A | | | |
| 0.098 (0.5) | N/A | | | |
| 0.156 (0.011) | N/A | | | |
| 0.162 (0.024) | N/A | | | |
| 0.089 (0.028) | N/A | | | |
| 0.128 (0.583) | N/A | | | |
| 0.029 (0.019) | N/A | | | |
| 0.089 (0.428) | N/A | | | |
| 0.150 (0.013) | N/A | | | |
| 0.085 (0.011) | N/A | | | |
| 0.153 (0.010) | N/A | | | |
| 0.185 (0.032) | N/A | | | |
| 0.062 (0.013) | N/A | | | |
| 0.144 (0.040) | N/A | | | |
| 0.112 (0.04) | N/A | | | |
| 0.022 (0.312) | N/A | | | |
| 0.193 (0.014) | N/A | | | |
| 0.022 (0.021) | N/A | | | |
| 0.023 (0.030) | N/A | | | |

| | |
|---|---|
| 0.061 (0.053) | N/A |
| 0.104 (0.071) | N/A |
| 0.152 (0.036) | N/A |
| 0.069 (0.026) | N/A |
| 0.120 (0.040) | N/A |
| 0.067 (0.052) | N/A |
| 0.170 (0.263) | N/A |
| 0.068 (0.096) | N/A |
| 0.119 (0.039) | N/A |
| 0.072 (0.115) | N/A |
| 0.013 (0.625) | N/A |
| 0.152 (0.388) | N/A |
| 0.161 (0.5) | N/A |
| 0.117 (0.011) | N/A |
| 0.094 (0.010) | N/A |
| 0.202 (0.057) | N/A |
| 0.191 (0.040) | N/A |
| 0.083 (0.042) | N/A |
| 0.123 (0.040) | N/A |
| 0.111 (0.028) | N/A |
| 0.174 (0.153) | N/A |
| 0.039 (0.5) | N/A |
| 0.082 (0.022) | N/A |
| 0.144 (0.033) | N/A |
| 0.192 (0.027) | N/A |
| 0.117 (0.036) | N/A |
| 0.137 (0.043) | N/A |
| 0.210 (0.024) | N/A |
| 0.086 (0.212) | N/A |
| 0.101 (0.018) | N/A |
| 0.066 (0.052) | N/A |
| 0.116 (0.019) | N/A |
| 0.129 (0.2) | N/A |
| 0.146 (0.053) | N/A |
| 0.068 (0.034) | N/A |
| 0.169 (0.014) | N/A |
| 0.159 (0.018) | N/A |
| 0.146 (0.045) | N/A |

| | |
|---|---|
| 0.065 (0.014) | N/A |
| 0.072 (0.3) | N/A |
| 0.087 (0.049) | N/A |
| 0.183 (0.011) | N/A |
| 0.084 (0.050) | N/A |
| 0.149 (0.023) | N/A |
| 0.125 (0.010) | N/A |
| 0.122 (0.022) | N/A |
| 0.114 (0.014) | N/A |
| 0.114 (0.014) | N/A |
| 0.130 (0.015) | N/A |
| 0.027 (0.583) | N/A |
| 0.017 (0.416) | N/A |
| 0.105 (0.014) | N/A |
| 0.083 (0.040) | N/A |
| 0.136 (0.057) | N/A |
| 0.082 (0.072) | N/A |
| 0.122 (0.046) | N/A |
| 0.012 (0.625) | N/A |
| 0.104 (0.042) | N/A |
| 0.032 (0.034) | N/A |
| 0.181 (0.016) | N/A |
| 0.054 (0.017) | N/A |
| 0.066 (0.040) | N/A |
| 0.091 (0.027) | N/A |
| 0.049 (0.020) | N/A |
| 0.088 (0.034) | N/A |
| 0.078 (0.032) | N/A |
| 0.194 (0.5) | N/A |
| 0.108 (0.018) | N/A |
| 0.069 (0.010) | N/A |
| 0.081 (0.043) | N/A |
| 0.143 (0.011) | N/A |
| 0.117 (0.071) | N/A |
| 0.042 (0.010) | N/A |
| 0.096 (0.053) | N/A |
| 0.087 (0.4) | N/A |
| 0.077 (0.016) | N/A |

| | |
|---|---|
| 0.120 (0.014) | N/A |
| 0.122 (0.028) | N/A |
| 0.125 (0.050) | N/A |
| 0.115 (0.063) | N/A |
| 0.075 (0.086) | N/A |
| 0.065 (0.061) | N/A |
| 0.104 (0.6) | N/A |
| 0.140 (0.072) | N/A |
| 0.163 (0.037) | N/A |
| 0.104 (0.033) | N/A |
| 0.036 (0.5) | N/A |
| 0.103 (0.285) | N/A |
| 0.060 (0.013) | N/A |
| 0.174 (0.017) | N/A |
| 0.090 (0.030) | N/A |
| 0.195 (0.1) | N/A |
| 0.072 (0.039) | N/A |
| 0.137 (0.6) | N/A |
| 0.111 (0.028) | N/A |
| 0.129 (0.033) | N/A |
| 0.166 (0.022) | N/A |
| 0.100 (0.025) | N/A |
| 0.179 (0.061) | N/A |
| 0.182 (0.018) | N/A |
| 0.118 (0.023) | N/A |
| 0.158 (0.033) | N/A |
| 0.176 (0.013) | N/A |
| 0.111 (0.020) | N/A |
| 0.111 (0.106) | N/A |
| 0.155 (0.018) | N/A |
| 0.129 (0.016) | N/A |
| 0.111 (0.027) | N/A |
| 0.068 (0.562) | N/A |
| 0.045 (0.018) | N/A |
| 0.026 (0.583) | N/A |
| 0.117 (0.054) | N/A |
| 0.107 (0.058) | N/A |
| 0.112 (0.04) | N/A |

| | |
|---|---|
| 0.055 (0.084) | N/A |
| 0.062 (0.027) | N/A |
| 0.073 (0.027) | N/A |
| 0.132 (0.068) | N/A |
| 0.050 (0.583) | N/A |
| 0.086 (0.625) | N/A |
| 0.194 (0.028) | N/A |
| 0.106 (0.043) | N/A |
| 0.113 (0.032) | N/A |
| 0.190 (0.157) | N/A |
| 0.119 (0.012) | N/A |
| 0.087 (0.055) | N/A |
| 0.181 (0.015) | N/A |
| 0.158 (0.080) | N/A |
| 0.161 (0.050) | N/A |
| 0.101 (0.025) | N/A |
| 0.135 (0.010) | N/A |
| 0.167 (0.020) | N/A |
| 0.101 (0.032) | N/A |
| 0.121 (0.029) | N/A |
| 0.086 (0.028) | N/A |
| 0.118 (0.024) | N/A |
| 0.147 (0.454) | N/A |
| 0.136 (0.043) | N/A |
| 0.134 (0.017) | N/A |
| 0.111 (0.020) | N/A |
| 0.115 (0.033) | N/A |
| 0.151 (0.018) | N/A |
| 0.052 (0.021) | N/A |
| 0.082 (0.104) | N/A |
| 0.162 (0.013) | N/A |
| 0.136 (0.037) | N/A |
| 0.052 (0.056) | N/A |
| 0.085 (0.018) | N/A |
| 0.133 (0.032) | N/A |
| 0.079 (0.108) | N/A |
| 0.131 (0.083) | N/A |
| 0.125 (0.036) | N/A |

| | |
|---|---|
| 0.101 (0.025) | N/A |
| 0.085 (0.034) | N/A |
| 0.099 (0.065) | N/A |
| 0.174 (0.092) | N/A |
| 0.131 (0.045) | N/A |
| 0.059 (0.058) | N/A |
| 0.165 (0.019) | N/A |
| 0.138 (0.039) | N/A |
| 0.040 (0.029) | N/A |
| 0.051 (0.064) | N/A |
| 0.099 (0.044) | N/A |
| 0.091 (0.025) | N/A |
| 0.024 (0.25) | N/A |
| 0.133 (0.021) | N/A |
| 0.041 (0.041) | N/A |
| 0.097 (0.077) | N/A |
| 0.008 (0.011) | N/A |
| 0.114 (0.026) | N/A |
| 0.163 (0.022) | N/A |
| 0.166 (0.187) | N/A |
| 0.118 (0.075) | N/A |
| 0.041 (0.018) | N/A |
| 0.045 (0.018) | N/A |
| 0.165 (0.014) | N/A |
| 0.093 (0.024) | N/A |
| 0.143 (0.055) | N/A |
| 0.167 (0.012) | N/A |
| 0.160 (0.012) | N/A |
| 0.179 (0.119) | N/A |
| 0.023 (0.051) | N/A |
| 0.141 (0.048) | N/A |
| 0.159 (0.016) | N/A |
| 0.024 (0.016) | N/A |
| 0.070 (0.010) | N/A |
| 0.008 (0.016) | N/A |
| 0.121 (0.031) | N/A |
| 0.050 (0.3) | N/A |
| 0.110 (0.086) | N/A |

| | |
|---|---|
| 0.072 (0.583) | N/A |
| 0.134 (0.035) | N/A |
| 0.159 (0.024) | N/A |
| 0.091 (0.076) | N/A |
| 0.027 (0.055) | N/A |
| 0.143 (0.073) | N/A |
| 0.161 (0.033) | N/A |
| 0.081 (0.312) | N/A |
| 0.188 (0.030) | N/A |
| 0.120 (0.028) | N/A |
| 0.049 (0.5) | N/A |
| 0.044 (0.048) | N/A |
| 0.047 (0.344) | N/A |
| 0.130 (0.010) | N/A |
| 0.041 (0.437) | N/A |
| 0.069 (0.016) | N/A |
| 0.118 (0.423) | N/A |
| 0.068 (0.031) | N/A |
| 0.106 (0.022) | N/A |
| 0.105 (0.024) | N/A |
| 0.030 (0.312) | N/A |
| 0.051 (0.056) | N/A |
| 0.123 (0.025) | N/A |
| 0.143 (0.041) | N/A |
| 0.198 (0.048) | N/A |
| 0.137 (0.025) | N/A |
| 0.206 (0.084) | N/A |
| 0.120 (0.562) | N/A |
| 0.084 (0.054) | N/A |
| 0.124 (0.061) | N/A |
| 0.157 (0.5) | N/A |
| 0.073 (0.625) | N/A |
| 0.139 (0.079) | N/A |
| 0.246 (0.011) | N/A |
| 0.135 (0.010) | N/A |
| 0.042 (0.170) | N/A |
| 0.116 (0.025) | N/A |
| 0.100 (0.055) | N/A |

| | |
|---|---|
| 0.002 (0.032) | N/A |
| 0.212 (0.021) | N/A |
| 0.099 (0.027) | N/A |
| 0.163 (0.016) | N/A |
| 0.124 (0.036) | N/A |
| 0.120 (0.022) | N/A |
| 0.102 (0.5) | N/A |
| 0.108 (0.046) | N/A |
| 0.085 (0.045) | N/A |
| 0.155 (0.035) | N/A |
| 0.022 (0.571) | N/A |
| 0.177 (0.416) | N/A |
| 0.100 (0.057) | N/A |
| 0.092 (0.086) | N/A |
| 0.105 (0.046) | N/A |
| 0.141 (0.011) | N/A |
| 0.122 (0.013) | N/A |
| 0.132 (0.036) | N/A |
| 0.087 (0.030) | N/A |
| 0.153 (0.011) | N/A |
| 0.115 (0.029) | N/A |
| 0.060 (0.028) | N/A |
| 0.187 (0.625) | N/A |
| 0.128 (0.023) | N/A |
| 0.088 (0.068) | N/A |
| 0.051 (0.013) | N/A |
| 0.120 (0.015) | N/A |
| 0.100 (0.103) | N/A |
| 0.166 (0.129) | N/A |
| 0.082 (0.059) | N/A |
| 0.107 (0.032) | N/A |
| 0.182 (0.131) | N/A |
| 0.184 (0.013) | N/A |
| 0.070 (0.017) | N/A |
| 0.038 (0.060) | N/A |
| 0.116 (0.028) | N/A |
| 0.128 (0.043) | N/A |
| 0.192 (0.052) | N/A |

| | |
|---|---|
| 0.047 (0.029) | N/A |
| 0.072 (0.583) | N/A |
| 0.069 (0.102) | N/A |
| 0.144 (0.011) | N/A |
| 0.014 (0.026) | N/A |
| 0.128 (0.029) | N/A |
| 0.141 (0.026) | N/A |
| 0.096 (0.047) | N/A |
| 0.068 (0.047) | N/A |
| 0.154 (0.047) | N/A |
| 0.136 (0.033) | N/A |
| 0.105 (0.02) | N/A |
| 0.009 (0.029) | N/A |
| 0.156 (0.019) | N/A |
| 0.082 (0.025) | N/A |
| 0.136 (0.046) | N/A |
| 0.103 (0.172) | N/A |
| 0.171 (0.04) | N/A |
| 0.093 (0.045) | N/A |
| 0.037 (0.015) | N/A |
| 0.097 (0.086) | N/A |
| 0.142 (0.021) | N/A |
| 0.109 (0.018) | N/A |
| 0.060 (0.010) | N/A |
| 0.025 (0.5) | N/A |
| 0.126 (0.011) | N/A |
| 0.067 (0.018) | N/A |
| 0.164 (0.066) | N/A |
| 0.089 (0.091) | N/A |
| 0.100 (0.027) | N/A |
| 0.043 (0.019) | N/A |
| 0.137 (0.014) | N/A |
| 0.067 (0.013) | N/A |
| 0.146 (0.029) | N/A |
| 0.180 (0.010) | N/A |
| 0.121 (0.017) | N/A |
| 0.127 (0.011) | N/A |
| 0.121 (0.263) | N/A |

| | |
|---|---|
| 0.246 (0.019) | N/A |
| 0.066 (0.043) | N/A |
| 0.042 (0.112) | N/A |
| 0.209 (0.020) | N/A |
| 0.019 (0.307) | N/A |
| 0.063 (0.011) | N/A |
| 0.111 (0.043) | N/A |
| 0.156 (0.272) | N/A |
| 0.153 (0.010) | N/A |
| 0.033 (0.051) | N/A |
| 0.048 (0.166) | N/A |
| 0.154 (0.6) | N/A |
| 0.082 (0.036) | N/A |
| 0.117 (0.080) | N/A |
| 0.088 (0.027) | N/A |
| 0.189 (0.013) | N/A |
| 0.047 (0.051) | N/A |
| 0.152 (0.024) | N/A |
| 0.133 (0.029) | N/A |
| 0.078 (0.047) | N/A |
| 0.090 (0.015) | N/A |
| 0.173 (0.333) | N/A |
| 0.086 (0.020) | N/A |
| 0.084 (0.012) | N/A |
| 0.106 (0.030) | N/A |
| 0.104 (0.084) | N/A |
| 0.245 (0.025) | N/A |
| 0.070 (0.5) | N/A |
| 0.078 (0.016) | N/A |
| 0.071 (0.017) | N/A |
| 0.150 (0.026) | N/A |
| 0.146 (0.127) | N/A |
| 0.137 (0.018) | N/A |
| 0.108 (0.011) | N/A |
| 0.101 (0.023) | N/A |
| 0.060 (0.013) | N/A |
| 0.152 (0.021) | N/A |
| 0.147 (0.275) | N/A |

| | |
|---|---|
| 0.149 (0.012) | N/A |
| 0.102 (0.061) | N/A |
| 0.148 (0.018) | N/A |
| 0.141 (0.015) | N/A |
| 0.179 (0.014) | N/A |
| 0.142 (0.030) | N/A |
| 0.049 (0.025) | N/A |
| 0.144 (0.05) | N/A |
| 0.150 (0.011) | N/A |
| 0.097 (0.25) | N/A |
| 0.042 (0.086) | N/A |
| 0.146 (0.028) | N/A |
| 0.109 (0.017) | N/A |
| 0.067 (0.5) | N/A |
| 0.152 (0.5) | N/A |
| 0.150 (0.333) | N/A |
| 0.171 (0.012) | N/A |
| 0.162 (0.033) | N/A |
| 0.068 (0.043) | N/A |
| 0.078 (0.021) | N/A |
| 0.031 (0.5) | N/A |
| 0.175 (0.5) | N/A |
| 0.086 (0.014) | N/A |
| 0.143 (0.312) | N/A |
| 0.165 (0.043) | N/A |
| 0.075 (0.098) | N/A |
| 0.191 (0.011) | N/A |
| 0.116 (0.032) | N/A |
| 0.185 (0.019) | N/A |
| 0.074 (0.159) | N/A |
| 0.139 (0.043) | N/A |
| 0.141 (0.067) | N/A |
| 0.055 (0.031) | N/A |
| 0.196 (0.032) | N/A |
| 0.071 (0.6) | N/A |
| 0.099 (0.030) | N/A |
| 0.150 (0.031) | N/A |
| 0.366 (0.041) | N/A |

| | |
|---|---|
| 0.140 (0.013) | N/A |
| 0.123 (0.023) | N/A |
| 0.127 (0.025) | N/A |
| 0 (0.081) | N/A |
| 0.113 (0.040) | N/A |
| 0.147 (0.062) | N/A |
| 0.111 (0.039) | N/A |
| 0.093 (0.014) | N/A |
| 0.130 (0.063) | N/A |
| 0.063 (0.014) | N/A |
| 0.160 (0.042) | N/A |
| 0.079 (0.03) | N/A |
| 0.158 (0.018) | N/A |
| 0.103 (0.025) | N/A |
| 0.074 (0.035) | N/A |
| 0.118 (0.021) | N/A |
| 0.106 (0.020) | N/A |
| 0.090 (0.040) | N/A |
| 0.048 (0.6) | N/A |
| 0.174 (0.013) | N/A |
| 0.160 (0.034) | N/A |
| 0.119 (0.029) | N/A |
| 0.155 (0.024) | N/A |
| 0.148 (0.017) | N/A |
| 0.098 (0.030) | N/A |
| 0.176 (0.016) | N/A |
| 0.103 (0.039) | N/A |
| 0.146 (0.018) | N/A |
| 0.229 (0.312) | N/A |
| 0.132 (0.014) | N/A |
| 0.136 (0.5) | N/A |
| 0.187 (0.070) | N/A |
| 0.038 (0.291) | N/A |
| 0.084 (0.084) | N/A |
| 0.088 (0.033) | N/A |
| 0.067 (0.062) | N/A |
| 0.065 (0.025) | N/A |
| 0.063 (0.019) | N/A |

| | |
|---|---|
| 0.022 (0.625) | N/A |
| 0.121 (0.020) | N/A |
| 0.157 (0.024) | N/A |
| 0.074 (0.013) | N/A |
| 0.153 (0.05) | N/A |
| 0.034 (0.075) | N/A |
| 0.192 (0.037) | N/A |
| 0.071 (0.027) | N/A |
| 0.112 (0.060) | N/A |
| 0.146 (0.016) | N/A |
| 0.144 (0.038) | N/A |
| 0.025 (0.071) | N/A |
| 0.183 (0.021) | N/A |
| 0.182 (0.028) | N/A |
| 0.083 (0.025) | N/A |
| 0.176 (0.562) | N/A |
| 0.087 (0.021) | N/A |
| 0.151 (0.042) | N/A |
| 0.162 (0.5) | N/A |
| 0.042 (0.45) | N/A |
| 0.174 (0.625) | N/A |
| 0.017 (0.625) | N/A |
| 0.141 (0.019) | N/A |
| 0.177 (0.010) | N/A |
| 0.145 (0.038) | N/A |
| 0.006 (0.062) | N/A |
| 0.043 (0.012) | N/A |
| 0.048 (0.010) | N/A |
| 0.042 (0.020) | N/A |
| 0.086 (0.055) | N/A |
| 0.078 (0.035) | N/A |
| 0.116 (0.5) | N/A |
| 0.031 (0.015) | N/A |
| 0.145 (0.030) | N/A |
| 0.138 (0.023) | N/A |
| 0.118 (0.021) | N/A |
| 0.173 (0.023) | N/A |
| 0.093 (0.034) | N/A |

| | |
|---|---|
| 0.004 (0.059) | N/A |
| 0.128 (0.076) | N/A |
| 0.047 (0.090) | N/A |
| 0.084 (0.069) | N/A |
| 0.142 (0.010) | N/A |
| 0.044 (0.017) | N/A |
| 0.134 (0.5) | N/A |
| 0.163 (0.015) | N/A |
| 0.089 (0.018) | N/A |
| 0.087 (0.147) | N/A |
| 0.035 (0.437) | N/A |
| 0.130 (0.571) | N/A |
| 0.129 (0.019) | N/A |
| 0.034 (0.571) | N/A |
| 0.189 (0.018) | N/A |
| 0.104 (0.034) | N/A |
| 0.065 (0.022) | N/A |
| 0.145 (0.026) | N/A |
| 0.091 (0.014) | N/A |
| 0.134 (0.026) | N/A |
| 0.123 (0.02) | N/A |
| 0.086 (0.035) | N/A |
| 0.151 (0.036) | N/A |
| 0.120 (0.011) | N/A |
| 0.078 (0.023) | N/A |
| 0.137 (0.026) | N/A |
| 0.126 (0.016) | N/A |
| 0.054 (0.076) | N/A |
| 0.078 (0.625) | N/A |
| 0.129 (0.017) | N/A |
| 0.128 (0.011) | N/A |
| 0.111 (0.051) | N/A |
| 0.125 (0.016) | N/A |
| 0.090 (0.173) | N/A |
| 0.155 (0.021) | N/A |
| 0.139 (0.050) | N/A |
| 0.049 (0.5) | N/A |
| 0.083 (0.030) | N/A |

| | |
|---|---|
| 0.122 (0.020) | N/A |
| 0.141 (0.026) | N/A |
| 0.020 (0.583) | N/A |
| 0.127 (0.024) | N/A |
| 0.062 (0.069) | N/A |
| 0.180 (0.012) | N/A |
| 0.126 (0.048) | N/A |
| 0.194 (0.012) | N/A |
| 0.051 (0.291) | N/A |
| 0.091 (0.043) | N/A |
| 0.148 (0.068) | N/A |
| 0.029 (0.6) | N/A |
| 0.060 (0.017) | N/A |
| 0.080 (0.030) | N/A |
| 0.066 (0.625) | N/A |
| 0.138 (0.010) | N/A |
| 0.126 (0.028) | N/A |
| 0.089 (0.086) | N/A |
| 0.181 (0.018) | N/A |
| 0.165 (0.018) | N/A |
| 0.047 (0.625) | N/A |
| 0.170 (0.018) | N/A |
| 0.118 (0.136) | N/A |
| 0.103 (0.037) | N/A |
| 0.085 (0.034) | N/A |
| 0.131 (0.010) | N/A |
| 0.201 (0.026) | N/A |
| 0.175 (0.046) | N/A |
| 0.149 (0.012) | N/A |
| 0.078 (0.3) | N/A |
| 0.152 (0.583) | N/A |
| 0.164 (0.040) | N/A |
| 0.054 (0.048) | N/A |
| 0.152 (0.037) | N/A |
| 0.095 (0.013) | N/A |
| 0.113 (0.025) | N/A |
| 0.112 (0.016) | N/A |
| 0.077 (0.5) | N/A |

| | |
|---|---|
| 0.127 (0.021) | N/A |
| 0.000 (0.076) | N/A |
| 0.002 (0.416) | N/A |
| 0.112 (0.043) | N/A |
| 0.162 (0.033) | N/A |
| 0.046 (0.040) | N/A |
| 0.161 (0.019) | N/A |
| 0.033 (0.035) | N/A |
| 0.158 (0.036) | N/A |
| 0.034 (0.096) | N/A |
| 0.105 (0.036) | N/A |

ıg DMRs are identified as those located within +- 1kbp from the gene bounda

**apping genomic feature**

| *Strand* | *Overlap with DMR (bp)* | *Overlapping Transcript ID* |
|---|---|---|
| - | 116 | ENSRNOT00000044178 |
| - | 16 | ENSRNOT00000064708 |
| - | 165 | ENSRNOT00000068644 |
| - | 64 | ENSRNOT00000068327 |
| - | 285 | ENSRNOT00000020265 |
| - | 343 | ENSRNOT00000033491 |
| - | 404 | ENSRNOT00000026481 |
| - | 21 | ENSRNOT00000026481 |
| - | 21 | ENSRNOT00000026477 |
| - | 404 | ENSRNOT00000026477 |
| + | 57 | ENSRNOT00000020452 |
| - | 46 | ENSRNOT00000018138 |
| - | 169 | ENSRNOT00000026962 |
| + | 51 | ENSRNOT00000028277 |
| + | 476 | ENSRNOT00000028094 |
| - | 59 | ENSRNOT00000047200 |
| - | 43 | ENSRNOT00000047200 |
| - | 11 | ENSRNOT00000051086 |
| + | 60 | ENSRNOT00000017601 |
| + | 284 | ENSRNOT00000007312 |
| - | 376 | ENSRNOT00000004609 |
| + | 386 | ENSRNOT00000020230 |
| + | 144 | ENSRNOT00000020230 |
| + | 295 | ENSRNOT00000051058 |
| + | 295 | ENSRNOT00000043981 |
| + | 392 | ENSRNOT00000017181 |
| - | 183 | ENSRNOT00000068327 |
| + | 333 | ENSRNOT00000006832 |
| - | 113 | ENSRNOT00000065049 |
| - | 113 | ENSRNOT00000008138 |
| - | 94 | ENSRNOT00000026477 |
| - | 94 | ENSRNOT00000026481 |
| + | 8 | ENSRNOT00000057067 |

| | | |
|---|---|---|
| + | 157 | ENSRNOT00000058724 |
| + | 157 | ENSRNOT00000019512 |
| + | 157 | ENSRNOT00000019452 |
| - | 348 | ENSRNOT00000016983 |
| - | 686 | ENSRNOT00000016983 |
| - | 44 | ENSRNOT00000016983 |
| + | 259 | ENSRNOT00000014241 |
| + | 259 | ENSRNOT00000014249 |
| + | 246 | ENSRNOT00000066976 |
| + | 246 | ENSRNOT00000038566 |
| + | 246 | ENSRNOT00000065975 |
| - | 631 | ENSRNOT00000061615 |
| - | 631 | ENSRNOT00000011955 |
| + | 176 | ENSRNOT00000020452 |
| - | 145 | ENSRNOT00000018138 |
| + | 120 | ENSRNOT00000027999 |
| + | 197 | ENSRNOT00000009241 |
| + | 197 | ENSRNOT00000048391 |
| - | 584 | ENSRNOT00000022956 |
| - | 615 | ENSRNOT00000022956 |
| - | 615 | ENSRNOT00000068378 |
| - | 584 | ENSRNOT00000068378 |
| - | 164 | ENSRNOT00000068191 |
| - | 164 | ENSRNOT00000023153 |
| + | 123 | ENSRNOT00000048843 |
| - | 16 | ENSRNOT00000057944 |
| - | 16 | ENSRNOT00000017257 |
| - | 16 | ENSRNOT00000067589 |
| + | 94 | ENSRNOT00000028277 |
| + | 424 | ENSRNOT00000068701 |
| + | 424 | ENSRNOT00000028225 |
| - | 338 | ENSRNOT00000005881 |
| - | 138 | ENSRNOT00000064280 |
| - | 138 | ENSRNOT00000024564 |
| - | 597 | ENSRNOT00000047200 |
| - | 15 | ENSRNOT00000051086 |
| - | 166 | ENSRNOT00000051086 |
| - | 192 | ENSRNOT00000018447 |

| | | |
|---|---|---|
| + | 10 | ENSRNOT00000064695 |
| + | 418 | ENSRNOT00000064695 |
| - | 12 | ENSRNOT00000038551 |
| - | 412 | ENSRNOT00000014458 |
| - | 596 | ENSRNOT00000061402 |
| - | 596 | ENSRNOT00000008989 |
| + | 8 | ENSRNOT00000002650 |
| + | 440 | ENSRNOT00000021537 |
| + | 440 | ENSRNOT00000067152 |
| + | 168 | ENSRNOT00000061063 |
| + | 348 | ENSRNOT00000031400 |
| - | 165 | ENSRNOT00000036713 |
| - | 165 | ENSRNOT00000000629 |
| - | 325 | ENSRNOT00000033667 |
| - | 325 | ENSRNOT00000009198 |
| - | 328 | ENSRNOT00000067359 |
| - | 81 | ENSRNOT00000068327 |
| - | 328 | ENSRNOT00000002542 |
| - | 377 | ENSRNOT00000020265 |
| - | 229 | ENSRNOT00000016176 |
| - | 172 | ENSRNOT00000055012 |
| - | 144 | ENSRNOT00000053457 |
| + | 189 | ENSRNOT00000028277 |
| - | 21 | ENSRNOT00000047200 |
| - | 37 | ENSRNOT00000026962 |
| + | 476 | ENSRNOT00000028094 |
| - | 64 | ENSRNOT00000068327 |
| - | 91 | ENSRNOT00000020265 |
| + | 8 | ENSRNOT00000028277 |
| - | 24 | ENSRNOT00000033222 |
| - | 24 | ENSRNOT00000038125 |
| - | 24 | ENSRNOT00000033222 |
| - | 224 | ENSRNOT00000033222 |
| - | 224 | ENSRNOT00000038125 |
| - | 24 | ENSRNOT00000038125 |
| + | 210 | ENSRNOT00000064856 |
| + | 210 | ENSRNOT00000016428 |
| - | 269 | ENSRNOT00000065691 |

| | | |
|---|---|---|
| - | 269 | ENSRNOT00000066757 |
| - | 269 | ENSRNOT00000064688 |
| - | 269 | ENSRNOT00000026073 |
| + | 34 | ENSRNOT00000024733 |
| + | 862 | ENSRNOT00000018259 |
| - | 51 | ENSRNOT00000005584 |
| - | 66 | ENSRNOT00000023405 |
| - | 130 | ENSRNOT00000008425 |
| + | 239 | ENSRNOT00000063930 |
| - | 18 | ENSRNOT00000013745 |
| - | 112 | ENSRNOT00000013745 |
| + | 16 | ENSRNOT00000010316 |
| - | 166 | ENSRNOT00000059807 |
| - | 166 | ENSRNOT00000003230 |
| + | 322 | ENSRNOT00000034522 |
| + | 322 | ENSRNOT00000055574 |
| + | 322 | ENSRNOT00000014713 |
| - | 191 | ENSRNOT00000045074 |
| - | 191 | ENSRNOT00000016645 |
| + | 335 | ENSRNOT00000027552 |
| + | 44 | ENSRNOT00000019993 |
| + | 44 | ENSRNOT00000064045 |
| + | 208 | ENSRNOT00000024967 |
| - | 71 | ENSRNOT00000055351 |
| - | 219 | ENSRNOT00000029697 |
| - | 71 | ENSRNOT00000029697 |
| - | 219 | ENSRNOT00000055351 |
| - | 219 | ENSRNOT00000002447 |
| - | 219 | ENSRNOT00000002446 |
| - | 219 | ENSRNOT00000049974 |
| + | 673 | ENSRNOT00000058659 |
| + | 673 | ENSRNOT00000008885 |
| - | 845 | ENSRNOT00000020916 |
| - | 845 | ENSRNOT00000020883 |
| - | 24 | ENSRNOT00000002486 |
| + | 238 | ENSRNOT00000042088 |
| + | 286 | ENSRNOT00000042088 |
| - | 16 | ENSRNOT00000016153 |

| | | |
|---|---|---|
| - | 16 | ENSRNOT00000015894 |
| + | 499 | ENSRNOT00000064010 |
| + | 641 | ENSRNOT00000033405 |
| - | 840 | ENSRNOT00000046539 |
| - | 203 | ENSRNOT00000046539 |
| - | 127 | ENSRNOT00000046539 |
| + | 141 | ENSRNOT00000020690 |
| + | 202 | ENSRNOT00000020690 |
| + | 141 | ENSRNOT00000020690 |
| + | 12 | ENSRNOT00000065824 |
| + | 12 | ENSRNOT00000004119 |
| + | 12 | ENSRNOT00000066243 |
| + | 12 | ENSRNOT00000064999 |
| + | 12 | ENSRNOT00000004187 |
| + | 12 | ENSRNOT00000066344 |
| + | 1156 | ENSRNOT00000052392 |
| - | 12 | ENSRNOT00000039655 |
| - | 12 | ENSRNOT00000042964 |
| - | 12 | ENSRNOT00000013828 |
| - | 539 | ENSRNOT00000044390 |
| - | 539 | ENSRNOT00000026390 |
| - | 539 | ENSRNOT00000026215 |
| + | 408 | ENSRNOT00000065326 |
| + | 188 | ENSRNOT00000048501 |
| + | 408 | ENSRNOT00000048501 |
| + | 370 | ENSRNOT00000065326 |
| + | 78 | ENSRNOT00000046957 |
| + | 40 | ENSRNOT00000046957 |
| + | 205 | ENSRNOT00000046957 |
| + | 85 | ENSRNOT00000046957 |
| + | 277 | ENSRNOT00000019746 |
| + | 277 | ENSRNOT00000054685 |
| + | 277 | ENSRNOT00000054684 |
| + | 150 | ENSRNOT00000015145 |
| + | 150 | ENSRNOT00000015145 |
| + | 547 | ENSRNOT00000010588 |
| - | 390 | ENSRNOT00000036137 |
| + | 30 | ENSRNOT00000042623 |

| | | |
|---|---|---|
| + | 30 | ENSRNOT00000057824 |
| + | 30 | ENSRNOT00000046488 |
| + | 30 | ENSRNOT00000050595 |
| - | 16 | ENSRNOT00000015338 |
| + | 131 | ENSRNOT00000054795 |
| + | 131 | ENSRNOT00000066846 |
| + | 168 | ENSRNOT00000003327 |
| - | 445 | ENSRNOT00000066322 |
| + | 111 | ENSRNOT00000031109 |
| + | 248 | ENSRNOT00000068022 |
| + | 248 | ENSRNOT00000064066 |
| + | 248 | ENSRNOT00000049292 |
| + | 248 | ENSRNOT00000066299 |
| + | 115 | ENSRNOT00000037742 |
| + | 109 | ENSRNOT00000010012 |
| + | 28 | ENSRNOT00000051338 |
| + | 28 | ENSRNOT00000023859 |
| - | 14 | ENSRNOT00000035320 |
| + | 307 | ENSRNOT00000017966 |
| + | 131 | ENSRNOT00000017966 |
| + | 172 | ENSRNOT00000017966 |
| + | 10 | ENSRNOT00000020341 |
| + | 149 | ENSRNOT00000040631 |
| - | 267 | ENSRNOT00000016709 |
| - | 229 | ENSRNOT00000016709 |
| - | 186 | ENSRNOT00000011650 |
| + | 251 | ENSRNOT00000013993 |
| + | 101 | ENSRNOT00000001544 |
| - | 169 | ENSRNOT00000038398 |
| - | 477 | ENSRNOT00000001466 |
| - | 22 | ENSRNOT00000054959 |
| - | 12 | ENSRNOT00000034216 |
| + | 541 | ENSRNOT00000023506 |
| - | 403 | ENSRNOT00000062055 |
| - | 403 | ENSRNOT00000062054 |
| - | 403 | ENSRNOT00000014709 |
| - | 403 | ENSRNOT00000062052 |
| + | 199 | ENSRNOT00000001219 |

| | | |
|---|---|---|
| + | 584 | ENSRNOT00000049884 |
| + | 129 | ENSRNOT00000020836 |
| + | 56 | ENSRNOT00000067241 |
| + | 56 | ENSRNOT00000066305 |
| + | 56 | ENSRNOT00000028039 |
| + | 89 | ENSRNOT00000010712 |
| + | 89 | ENSRNOT00000010712 |
| - | 73 | ENSRNOT00000020026 |
| - | 73 | ENSRNOT00000040740 |
| - | 862 | ENSRNOT00000031909 |
| + | 296 | ENSRNOT00000008161 |
| - | 184 | ENSRNOT00000060765 |
| - | 184 | ENSRNOT00000008465 |
| - | 184 | ENSRNOT00000065299 |
| - | 184 | ENSRNOT00000060764 |
| - | 272 | ENSRNOT00000025458 |
| - | 272 | ENSRNOT00000067799 |
| + | 8 | ENSRNOT00000067191 |
| + | 8 | ENSRNOT00000068060 |
| + | 8 | ENSRNOT00000014091 |
| + | 131 | ENSRNOT00000040576 |
| + | 55 | ENSRNOT00000040576 |
| + | 99 | ENSRNOT00000026199 |
| + | 43 | ENSRNOT00000026199 |
| - | 176 | ENSRNOT00000035142 |
| - | 983 | ENSRNOT00000001503 |
| - | 378 | ENSRNOT00000017915 |
| - | 103 | ENSRNOT00000038748 |
| - | 306 | ENSRNOT00000017832 |
| + | 39 | ENSRNOT00000026210 |
| + | 50 | ENSRNOT00000026210 |
| + | 39 | ENSRNOT00000026210 |
| - | 432 | ENSRNOT00000001315 |
| + | 241 | ENSRNOT00000001642 |
| + | 161 | ENSRNOT00000010183 |
| - | 437 | ENSRNOT00000006604 |
| - | 593 | ENSRNOT00000044481 |
| - | 593 | ENSRNOT00000042956 |

| | | |
|---|---|---|
| - | 355 | ENSRNOT00000018147 |
| - | 360 | ENSRNOT00000011693 |
| - | 360 | ENSRNOT00000049798 |
| - | 360 | ENSRNOT00000064166 |
| + | 340 | ENSRNOT00000064300 |
| + | 340 | ENSRNOT00000001773 |
| + | 340 | ENSRNOT00000001774 |
| + | 340 | ENSRNOT00000041200 |
| + | 340 | ENSRNOT00000001778 |
| - | 8 | ENSRNOT00000026281 |
| + | 42 | ENSRNOT00000012082 |
| + | 14 | ENSRNOT00000038244 |
| + | 547 | ENSRNOT00000040939 |
| + | 547 | ENSRNOT00000027067 |
| - | 95 | ENSRNOT00000041119 |
| + | 316 | ENSRNOT00000058897 |
| + | 66 | ENSRNOT00000015113 |
| - | 56 | ENSRNOT00000007789 |
| - | 318 | ENSRNOT00000017218 |
| - | 318 | ENSRNOT00000048407 |
| - | 318 | ENSRNOT00000067659 |
| + | 154 | ENSRNOT00000059419 |
| + | 674 | ENSRNOT00000059419 |
| + | 154 | ENSRNOT00000059421 |
| + | 674 | ENSRNOT00000059421 |
| - | 54 | ENSRNOT00000024716 |
| + | 130 | ENSRNOT00000014376 |
| + | 130 | ENSRNOT00000014250 |
| + | 586 | ENSRNOT00000047858 |
| + | 586 | ENSRNOT00000044938 |
| + | 586 | ENSRNOT00000046262 |
| + | 586 | ENSRNOT00000018480 |
| - | 10 | ENSRNOT00000054950 |
| - | 10 | ENSRNOT00000067315 |
| - | 10 | ENSRNOT00000033517 |
| - | 10 | ENSRNOT00000033490 |
| + | 72 | ENSRNOT00000055237 |
| + | 72 | ENSRNOT00000055238 |

| | | |
|---|---|---|
| + | 72 | ENSRNOT00000055238 |
| + | 72 | ENSRNOT00000055237 |
| + | 52 | ENSRNOT00000056191 |
| + | 52 | ENSRNOT00000066549 |
| + | 52 | ENSRNOT00000015685 |
| - | 344 | ENSRNOT00000068658 |
| - | 16 | ENSRNOT00000005257 |
| - | 72 | ENSRNOT00000044063 |
| - | 72 | ENSRNOT00000058079 |
| - | 111 | ENSRNOT00000019189 |
| - | 111 | ENSRNOT00000019225 |
| + | 181 | ENSRNOT00000028117 |
| - | 358 | ENSRNOT00000029202 |
| + | 274 | ENSRNOT00000061012 |
| + | 274 | ENSRNOT00000061013 |
| - | 179 | ENSRNOT00000021176 |
| + | 146 | ENSRNOT00000024950 |
| + | 146 | ENSRNOT00000066336 |
| + | 571 | ENSRNOT00000037597 |
| + | 268 | ENSRNOT00000020049 |
| + | 268 | ENSRNOT00000019834 |
| + | 268 | ENSRNOT00000046229 |
| + | 134 | ENSRNOT00000051259 |
| + | 36 | ENSRNOT00000051259 |
| - | 194 | ENSRNOT00000068624 |
| - | 194 | ENSRNOT00000068067 |
| - | 194 | ENSRNOT00000065280 |
| - | 12 | ENSRNOT00000039353 |
| - | 439 | ENSRNOT00000021036 |
| - | 169 | ENSRNOT00000002896 |
| + | 91 | ENSRNOT00000066905 |
| + | 91 | ENSRNOT00000006996 |
| + | 91 | ENSRNOT00000056392 |
| - | 254 | ENSRNOT00000018684 |
| + | 141 | ENSRNOT00000057938 |
| + | 57 | ENSRNOT00000057938 |
| - | 30 | ENSRNOT00000023457 |
| - | 557 | ENSRNOT00000058075 |

| | | |
|---|---|---|
| - | 557 | ENSRNOT00000032782 |
| + | 515 | ENSRNOT00000015802 |
| + | 74 | ENSRNOT00000044751 |
| + | 74 | ENSRNOT00000000883 |
| + | 154 | ENSRNOT00000040072 |
| + | 154 | ENSRNOT00000064373 |
| - | 171 | ENSRNOT00000034425 |
| - | 317 | ENSRNOT00000022962 |
| - | 42 | ENSRNOT00000021544 |
| - | 89 | ENSRNOT00000056264 |
| - | 89 | ENSRNOT00000056265 |
| - | 77 | ENSRNOT00000056265 |
| - | 77 | ENSRNOT00000056264 |
| - | 342 | ENSRNOT00000010103 |
| - | 449 | ENSRNOT00000042532 |
| - | 449 | ENSRNOT00000061237 |
| + | 912 | ENSRNOT00000015210 |
| + | 85 | ENSRNOT00000036143 |
| + | 75 | ENSRNOT00000036143 |
| + | 5 | ENSRNOT00000036143 |
| + | 247 | ENSRNOT00000005337 |
| + | 227 | ENSRNOT00000020431 |
| + | 227 | ENSRNOT00000058918 |
| - | 157 | ENSRNOT00000019862 |
| - | 29 | ENSRNOT00000019862 |
| + | 222 | ENSRNOT00000067108 |
| + | 222 | ENSRNOT00000007889 |
| - | 144 | ENSRNOT00000026120 |
| - | 71 | ENSRNOT00000055146 |
| - | 630 | ENSRNOT00000055146 |
| - | 71 | ENSRNOT00000025238 |
| - | 71 | ENSRNOT00000025238 |
| - | 630 | ENSRNOT00000025238 |
| - | 71 | ENSRNOT00000055146 |
| + | 542 | ENSRNOT00000036633 |
| + | 50 | ENSRNOT00000036633 |
| - | 124 | ENSRNOT00000055697 |
| - | 124 | ENSRNOT00000057020 |

| | | |
|---|---|---|
| - | 124 | ENSRNOT00000058349 |
| - | 357 | ENSRNOT00000002795 |
| - | 357 | ENSRNOT00000060868 |
| - | 91 | ENSRNOT00000007299 |
| + | 214 | ENSRNOT00000015643 |
| + | 214 | ENSRNOT00000064852 |
| - | 275 | ENSRNOT00000038092 |
| - | 492 | ENSRNOT00000044467 |
| - | 278 | ENSRNOT00000040284 |
| - | 97 | ENSRNOT00000038537 |
| - | 97 | ENSRNOT00000040284 |
| - | 278 | ENSRNOT00000038537 |
| + | 523 | ENSRNOT00000066098 |
| + | 523 | ENSRNOT00000013314 |
| + | 523 | ENSRNOT00000067666 |
| + | 704 | ENSRNOT00000001734 |
| + | 359 | ENSRNOT00000001734 |
| + | 134 | ENSRNOT00000065683 |
| + | 134 | ENSRNOT00000001734 |
| - | 56 | ENSRNOT00000001766 |
| - | 271 | ENSRNOT00000001766 |
| - | 596 | ENSRNOT00000001669 |
| - | 596 | ENSRNOT00000001666 |
| - | 84 | ENSRNOT00000016745 |
| + | 276 | ENSRNOT00000016629 |
| + | 211 | ENSRNOT00000016629 |
| - | 176 | ENSRNOT00000064431 |
| + | 340 | ENSRNOT00000022774 |
| + | 205 | ENSRNOT00000022774 |
| - | 228 | ENSRNOT00000049832 |
| - | 540 | ENSRNOT00000057301 |
| - | 418 | ENSRNOT00000057301 |
| - | 540 | ENSRNOT00000049832 |
| - | 183 | ENSRNOT00000057301 |
| - | 183 | ENSRNOT00000049832 |
| - | 228 | ENSRNOT00000057301 |
| - | 418 | ENSRNOT00000049832 |
| + | 218 | ENSRNOT00000028151 |

| | | |
|---|---|---|
| + | 88 | ENSRNOT00000017831 |
| + | 545 | ENSRNOT00000013602 |
| + | 33 | ENSRNOT00000013602 |
| - | 239 | ENSRNOT00000033358 |
| - | 239 | ENSRNOT00000046143 |
| - | 239 | ENSRNOT00000043170 |
| - | 239 | ENSRNOT00000023352 |
| + | 307 | ENSRNOT00000045288 |
| + | 307 | ENSRNOT00000017907 |
| - | 265 | ENSRNOT00000066186 |
| - | 265 | ENSRNOT00000061967 |
| - | 657 | ENSRNOT00000013385 |
| - | 386 | ENSRNOT00000068037 |
| - | 386 | ENSRNOT00000011812 |
| - | 134 | ENSRNOT00000044431 |
| - | 134 | ENSRNOT00000018556 |
| - | 126 | ENSRNOT00000063877 |
| - | 126 | ENSRNOT00000065670 |
| - | 126 | ENSRNOT00000037017 |
| - | 126 | ENSRNOT00000066643 |
| - | 1771 | ENSRNOT00000009904 |
| - | 18 | ENSRNOT00000046032 |
| - | 18 | ENSRNOT00000014202 |
| + | 13 | ENSRNOT00000055627 |
| + | 13 | ENSRNOT00000055624 |
| + | 13 | ENSRNOT00000055626 |
| + | 13 | ENSRNOT00000003777 |
| + | 13 | ENSRNOT00000068702 |
| + | 13 | ENSRNOT00000055623 |
| + | 13 | ENSRNOT00000055625 |
| + | 487 | ENSRNOT00000025899 |
| + | 487 | ENSRNOT00000025905 |
| + | 487 | ENSRNOT00000025894 |
| - | 359 | ENSRNOT00000001733 |
| - | 573 | ENSRNOT00000016167 |
| - | 285 | ENSRNOT00000013232 |
| - | 285 | ENSRNOT00000057098 |
| - | 285 | ENSRNOT00000049657 |

| | | |
|---|---|---|
| - | 125 | ENSRNOT00000065143 |
| - | 125 | ENSRNOT00000064881 |
| + | 459 | ENSRNOT00000000824 |
| - | 184 | ENSRNOT00000014772 |
| - | 157 | ENSRNOT00000034519 |
| - | 72 | ENSRNOT00000042253 |
| - | 72 | ENSRNOT00000029001 |
| - | 205 | ENSRNOT00000020749 |
| - | 200 | ENSRNOT00000001485 |
| + | 121 | ENSRNOT00000014271 |
| - | 199 | ENSRNOT00000002916 |
| - | 12 | ENSRNOT00000002916 |
| - | 77 | ENSRNOT00000002916 |
| - | 135 | ENSRNOT00000002916 |
| - | 14 | ENSRNOT00000041566 |
| - | 14 | ENSRNOT00000021268 |
| - | 191 | ENSRNOT00000052035 |
| - | 14 | ENSRNOT00000028222 |
| - | 402 | ENSRNOT00000034151 |
| - | 503 | ENSRNOT00000034151 |
| - | 503 | ENSRNOT00000060186 |
| - | 402 | ENSRNOT00000060186 |
| - | 392 | ENSRNOT00000035400 |
| + | 705 | ENSRNOT00000055511 |
| + | 705 | ENSRNOT00000055512 |
| + | 705 | ENSRNOT00000024443 |
| - | 250 | ENSRNOT00000042418 |
| + | 314 | ENSRNOT00000057473 |
| + | 314 | ENSRNOT00000060222 |
| + | 408 | ENSRNOT00000050449 |
| + | 408 | ENSRNOT00000043875 |
| + | 408 | ENSRNOT00000051121 |
| + | 408 | ENSRNOT00000039382 |
| + | 408 | ENSRNOT00000045335 |
| + | 112 | ENSRNOT00000000737 |
| + | 16 | ENSRNOT00000022764 |
| + | 16 | ENSRNOT00000067318 |
| + | 415 | ENSRNOT00000042575 |

| | | |
|---|---|---|
| + | 415 | ENSRNOT00000060641 |
| + | 415 | ENSRNOT00000010219 |
| + | 514 | ENSRNOT00000011237 |
| + | 8 | ENSRNOT00000035338 |
| - | 273 | ENSRNOT00000021954 |
| + | 10 | ENSRNOT00000038688 |
| + | 10 | ENSRNOT00000040778 |
| - | 44 | ENSRNOT00000065043 |
| - | 44 | ENSRNOT00000021637 |
| + | 138 | ENSRNOT00000006986 |
| + | 641 | ENSRNOT00000056469 |
| + | 641 | ENSRNOT00000009222 |
| - | 236 | ENSRNOT00000043660 |
| + | 250 | ENSRNOT00000025000 |
| + | 250 | ENSRNOT00000025090 |
| + | 657 | ENSRNOT00000013535 |
| - | 561 | ENSRNOT00000065888 |
| - | 561 | ENSRNOT00000031274 |
| - | 561 | ENSRNOT00000031266 |
| - | 561 | ENSRNOT00000066696 |
| - | 561 | ENSRNOT00000031252 |
| + | 334 | ENSRNOT00000016797 |
| + | 334 | ENSRNOT00000059146 |
| + | 334 | ENSRNOT00000060206 |
| + | 154 | ENSRNOT00000051422 |
| + | 154 | ENSRNOT00000028265 |
| + | 212 | ENSRNOT00000023017 |
| + | 574 | ENSRNOT00000023017 |
| - | 395 | ENSRNOT00000064705 |
| - | 131 | ENSRNOT00000067426 |
| - | 131 | ENSRNOT00000016659 |
| - | 395 | ENSRNOT00000067426 |
| - | 131 | ENSRNOT00000064705 |
| - | 131 | ENSRNOT00000066811 |
| - | 131 | ENSRNOT00000064896 |
| - | 131 | ENSRNOT00000067426 |
| - | 395 | ENSRNOT00000064896 |
| - | 395 | ENSRNOT00000066811 |

| | | |
|---|---|---|
| - | 395 | ENSRNOT00000016659 |
| - | 395 | ENSRNOT00000045519 |
| - | 131 | ENSRNOT00000045519 |
| - | 48 | ENSRNOT00000065473 |
| - | 48 | ENSRNOT00000017968 |
| - | 450 | ENSRNOT00000022820 |
| + | 222 | ENSRNOT00000066832 |
| + | 222 | ENSRNOT00000037246 |
| + | 66 | ENSRNOT00000063893 |
| + | 66 | ENSRNOT00000013809 |
| + | 150 | ENSRNOT00000044049 |
| + | 150 | ENSRNOT00000044049 |
| - | 72 | ENSRNOT00000056366 |
| - | 72 | ENSRNOT00000015938 |
| - | 244 | ENSRNOT00000002330 |
| - | 244 | ENSRNOT00000002330 |
| - | 393 | ENSRNOT00000002330 |
| - | 22 | ENSRNOT00000054970 |
| - | 12 | ENSRNOT00000003694 |
| - | 12 | ENSRNOT00000067921 |
| - | 12 | ENSRNOT00000032105 |
| - | 12 | ENSRNOT00000068563 |
| - | 10 | ENSRNOT00000039049 |
| + | 10 | ENSRNOT00000019923 |
| - | 188 | ENSRNOT00000010017 |
| - | 188 | ENSRNOT00000010827 |
| - | 379 | ENSRNOT00000024030 |
| + | 402 | ENSRNOT00000017374 |
| + | 240 | ENSRNOT00000001248 |
| - | 256 | ENSRNOT00000061821 |
| - | 256 | ENSRNOT00000001384 |
| + | 525 | ENSRNOT00000043784 |
| + | 399 | ENSRNOT00000067742 |
| + | 399 | ENSRNOT00000054933 |
| + | 35 | ENSRNOT00000067742 |
| + | 35 | ENSRNOT00000054933 |
| - | 10 | ENSRNOT00000025013 |
| - | 10 | ENSRNOT00000024981 |

| | | |
|---|---|---|
| - | 264 | ENSRNOT00000051360 |
| + | 324 | ENSRNOT00000025711 |
| - | 197 | ENSRNOT00000011085 |
| + | 382 | ENSRNOT00000000733 |
| + | 382 | ENSRNOT00000000732 |
| - | 34 | ENSRNOT00000032290 |
| - | 66 | ENSRNOT00000067135 |
| - | 66 | ENSRNOT00000003196 |
| + | 356 | ENSRNOT00000039377 |
| - | 8 | ENSRNOT00000043666 |
| + | 286 | ENSRNOT00000068829 |
| + | 12 | ENSRNOT00000024334 |
| + | 12 | ENSRNOT00000067508 |
| + | 131 | ENSRNOT00000012111 |
| + | 14 | ENSRNOT00000012111 |
| - | 147 | ENSRNOT00000063902 |
| - | 147 | ENSRNOT00000004097 |
| - | 147 | ENSRNOT00000065286 |
| - | 149 | ENSRNOT00000046523 |
| - | 149 | ENSRNOT00000037539 |
| - | 149 | ENSRNOT00000029196 |
| - | 149 | ENSRNOT00000067594 |
| - | 171 | ENSRNOT00000048712 |
| - | 26 | ENSRNOT00000048712 |
| - | 171 | ENSRNOT00000025461 |
| - | 171 | ENSRNOT00000042145 |
| - | 26 | ENSRNOT00000042145 |
| - | 26 | ENSRNOT00000025461 |
| - | 26 | ENSRNOT00000066498 |
| - | 14 | ENSRNOT00000031172 |
| - | 83 | ENSRNOT00000044012 |
| - | 83 | ENSRNOT00000013355 |
| - | 83 | ENSRNOT00000013621 |
| - | 36 | ENSRNOT00000065515 |
| - | 36 | ENSRNOT00000008301 |
| - | 36 | ENSRNOT00000050355 |
| - | 36 | ENSRNOT00000064338 |
| - | 36 | ENSRNOT00000045862 |

| | | |
|---|---|---|
| - | 117 | ENSRNOT00000064338 |
| - | 139 | ENSRNOT00000045862 |
| - | 117 | ENSRNOT00000050355 |
| - | 117 | ENSRNOT00000008301 |
| - | 139 | ENSRNOT00000008301 |
| - | 139 | ENSRNOT00000064338 |
| - | 139 | ENSRNOT00000050355 |
| - | 139 | ENSRNOT00000065515 |
| - | 117 | ENSRNOT00000045862 |
| - | 117 | ENSRNOT00000065515 |
| - | 563 | ENSRNOT00000055194 |
| - | 58 | ENSRNOT00000022356 |
| - | 58 | ENSRNOT00000068174 |
| - | 58 | ENSRNOT00000067899 |
| - | 403 | ENSRNOT00000012640 |
| - | 41 | ENSRNOT00000012640 |
| - | 560 | ENSRNOT00000049699 |
| - | 560 | ENSRNOT00000040559 |
| - | 560 | ENSRNOT00000044778 |
| - | 188 | ENSRNOT00000017967 |
| + | 544 | ENSRNOT00000049486 |
| + | 544 | ENSRNOT00000021758 |
| + | 169 | ENSRNOT00000056430 |
| + | 544 | ENSRNOT00000056430 |
| + | 169 | ENSRNOT00000021758 |
| + | 169 | ENSRNOT00000049486 |
| - | 296 | ENSRNOT00000026540 |
| - | 296 | ENSRNOT00000046708 |
| - | 142 | ENSRNOT00000046708 |
| - | 142 | ENSRNOT00000026540 |
| + | 10 | ENSRNOT00000011822 |
| + | 10 | ENSRNOT00000057407 |
| + | 10 | ENSRNOT00000011717 |
| + | 56 | ENSRNOT00000019157 |
| - | 219 | ENSRNOT00000024932 |
| - | 219 | ENSRNOT00000045629 |
| - | 132 | ENSRNOT00000024932 |
| - | 132 | ENSRNOT00000045629 |

| | | |
|---|---|---|
| - | 536 | ENSRNOT00000031925 |
| - | 164 | ENSRNOT00000052121 |
| - | 164 | ENSRNOT00000019465 |
| + | 226 | ENSRNOT00000019231 |
| + | 226 | ENSRNOT00000067616 |
| - | 72 | ENSRNOT00000055523 |
| - | 218 | ENSRNOT00000021193 |
| - | 218 | ENSRNOT00000055523 |
| - | 72 | ENSRNOT00000021193 |
| + | 298 | ENSRNOT00000010002 |
| + | 298 | ENSRNOT00000066151 |
| + | 298 | ENSRNOT00000010308 |
| + | 269 | ENSRNOT00000011565 |
| + | 159 | ENSRNOT00000064366 |
| + | 159 | ENSRNOT00000065397 |
| + | 159 | ENSRNOT00000019673 |
| - | 148 | ENSRNOT00000002460 |
| - | 148 | ENSRNOT00000032767 |
| - | 148 | ENSRNOT00000040645 |
| - | 148 | ENSRNOT00000064443 |
| - | 64 | ENSRNOT00000056779 |
| - | 61 | ENSRNOT00000056779 |
| - | 18 | ENSRNOT00000056779 |
| - | 166 | ENSRNOT00000005306 |
| - | 4 | ENSRNOT00000056779 |
| - | 19 | ENSRNOT00000056779 |
| - | 28 | ENSRNOT00000057950 |
| + | 82 | ENSRNOT00000001507 |
| + | 101 | ENSRNOT00000001559 |
| + | 326 | ENSRNOT00000001559 |
| + | 90 | ENSRNOT00000045375 |
| - | 272 | ENSRNOT00000002206 |
| - | 28 | ENSRNOT00000002262 |
| - | 28 | ENSRNOT00000066940 |
| - | 12 | ENSRNOT00000005067 |
| + | 208 | ENSRNOT00000065321 |
| + | 208 | ENSRNOT00000006264 |
| - | 224 | ENSRNOT00000006716 |

| | | |
|---|---|---|
| - | 224 | ENSRNOT00000066904 |
| - | 774 | ENSRNOT00000058962 |
| - | 336 | ENSRNOT00000064182 |
| - | 774 | ENSRNOT00000065151 |
| - | 774 | ENSRNOT00000064182 |
| - | 336 | ENSRNOT00000065151 |
| - | 336 | ENSRNOT00000058962 |
| + | 249 | ENSRNOT00000006652 |
| + | 27 | ENSRNOT00000006652 |
| + | 249 | ENSRNOT00000006652 |
| - | 106 | ENSRNOT00000007233 |
| - | 186 | ENSRNOT00000008414 |
| + | 183 | ENSRNOT00000049597 |
| + | 183 | ENSRNOT00000040097 |
| + | 2 | ENSRNOT00000009102 |
| + | 344 | ENSRNOT00000009102 |
| - | 135 | ENSRNOT00000009514 |
| - | 135 | ENSRNOT00000009542 |
| - | 135 | ENSRNOT00000066187 |
| - | 10 | ENSRNOT00000009656 |
| + | 74 | ENSRNOT00000046801 |
| + | 227 | ENSRNOT00000009987 |
| - | 34 | ENSRNOT00000010427 |
| - | 203 | ENSRNOT00000011061 |
| + | 28 | ENSRNOT00000012169 |
| + | 28 | ENSRNOT00000012153 |
| + | 28 | ENSRNOT00000066645 |
| + | 28 | ENSRNOT00000061228 |
| + | 115 | ENSRNOT00000060556 |
| - | 8 | ENSRNOT00000011697 |
| + | 156 | ENSRNOT00000013152 |
| - | 222 | ENSRNOT00000059892 |
| - | 222 | ENSRNOT00000014977 |
| + | 271 | ENSRNOT00000015271 |
| + | 601 | ENSRNOT00000015271 |
| - | 587 | ENSRNOT00000064769 |
| - | 587 | ENSRNOT00000016070 |
| - | 587 | ENSRNOT00000064689 |

| | | |
|---|---|---|
| - | 587 | ENSRNOT00000015988 |
| + | 255 | ENSRNOT00000016252 |
| + | 255 | ENSRNOT00000051737 |
| - | 14 | ENSRNOT00000016558 |
| - | 99 | ENSRNOT00000017103 |
| - | 226 | ENSRNOT00000017103 |
| - | 119 | ENSRNOT00000055113 |
| - | 119 | ENSRNOT00000017414 |
| - | 119 | ENSRNOT00000055111 |
| - | 119 | ENSRNOT00000055112 |
| - | 119 | ENSRNOT00000064976 |
| - | 618 | ENSRNOT00000066692 |
| - | 618 | ENSRNOT00000018094 |
| - | 618 | ENSRNOT00000067721 |
| - | 618 | ENSRNOT00000029628 |
| - | 618 | ENSRNOT00000067293 |
| - | 618 | ENSRNOT00000029640 |
| - | 618 | ENSRNOT00000040781 |
| - | 303 | ENSRNOT00000041125 |
| + | 332 | ENSRNOT00000020513 |
| + | 332 | ENSRNOT00000052015 |
| - | 48 | ENSRNOT00000020332 |
| - | 173 | ENSRNOT00000021961 |
| - | 166 | ENSRNOT00000065207 |
| - | 166 | ENSRNOT00000022289 |
| - | 166 | ENSRNOT00000022253 |
| - | 166 | ENSRNOT00000022331 |
| - | 166 | ENSRNOT00000068183 |
| - | 166 | ENSRNOT00000065448 |
| - | 227 | ENSRNOT00000022076 |
| - | 106 | ENSRNOT00000064714 |
| - | 106 | ENSRNOT00000022742 |
| - | 106 | ENSRNOT00000067202 |
| - | 195 | ENSRNOT00000045535 |
| - | 186 | ENSRNOT00000022481 |
| - | 186 | ENSRNOT00000064845 |
| - | 195 | ENSRNOT00000022481 |
| - | 186 | ENSRNOT00000045535 |

| | | |
|---|---|---|
| - | 195 | ENSRNOT00000064845 |
| - | 22 | ENSRNOT00000022481 |
| - | 22 | ENSRNOT00000045535 |
| - | 403 | ENSRNOT00000048394 |
| - | 22 | ENSRNOT00000064845 |
| - | 240 | ENSRNOT00000066320 |
| - | 240 | ENSRNOT00000065005 |
| + | 681 | ENSRNOT00000022744 |
| - | 118 | ENSRNOT00000023869 |
| - | 28 | ENSRNOT00000023869 |
| - | 260 | ENSRNOT00000025106 |
| - | 260 | ENSRNOT00000066120 |
| - | 260 | ENSRNOT00000055498 |
| - | 352 | ENSRNOT00000025377 |
| - | 212 | ENSRNOT00000025488 |
| + | 334 | ENSRNOT00000026607 |
| + | 60 | ENSRNOT00000027337 |
| + | 60 | ENSRNOT00000063959 |
| + | 60 | ENSRNOT00000027304 |
| + | 461 | ENSRNOT00000027875 |
| + | 461 | ENSRNOT00000027871 |
| - | 245 | ENSRNOT00000028745 |
| + | 363 | ENSRNOT00000037606 |
| - | 12 | ENSRNOT00000050812 |
| - | 39 | ENSRNOT00000050812 |
| - | 10 | ENSRNOT00000030847 |
| - | 182 | ENSRNOT00000039555 |
| + | 326 | ENSRNOT00000035049 |
| - | 58 | ENSRNOT00000060968 |
| - | 176 | ENSRNOT00000030543 |
| - | 290 | ENSRNOT00000068003 |
| - | 56 | ENSRNOT00000030543 |
| - | 58 | ENSRNOT00000030543 |
| - | 56 | ENSRNOT00000060968 |
| - | 176 | ENSRNOT00000060968 |
| + | 14 | ENSRNOT00000041301 |
| + | 33 | ENSRNOT00000032116 |
| + | 123 | ENSRNOT00000032116 |

| | | |
|---|---|---|
| - | 374 | ENSRNOT00000037527 |
| - | 200 | ENSRNOT00000033090 |
| + | 33 | ENSRNOT00000035916 |
| + | 84 | ENSRNOT00000035916 |
| + | 250 | ENSRNOT00000035916 |
| + | 33 | ENSRNOT00000035916 |
| + | 194 | ENSRNOT00000033352 |
| - | 73 | ENSRNOT00000030341 |
| + | 240 | ENSRNOT00000032733 |
| - | 44 | ENSRNOT00000046190 |
| + | 10 | ENSRNOT00000051275 |
| + | 358 | ENSRNOT00000048023 |
| + | 298 | ENSRNOT00000048023 |
| - | 229 | ENSRNOT00000047198 |
| - | 12 | ENSRNOT00000047321 |
| - | 12 | ENSRNOT00000045773 |
| - | 61 | ENSRNOT00000063800 |
| - | 61 | ENSRNOT00000051739 |
| + | 348 | ENSRNOT00000051363 |
| + | 279 | ENSRNOT00000047563 |
| + | 257 | ENSRNOT00000056411 |
| + | 10 | ENSRNOT00000041804 |
| - | 13 | ENSRNOT00000041400 |
| - | 12 | ENSRNOT00000041400 |
| - | 132 | ENSRNOT00000041400 |
| - | 559 | ENSRNOT00000041400 |
| - | 168 | ENSRNOT00000041400 |
| + | 125 | ENSRNOT00000044742 |
| + | 60 | ENSRNOT00000041562 |
| + | 605 | ENSRNOT00000041562 |
| + | 120 | ENSRNOT00000041562 |
| + | 86 | ENSRNOT00000041562 |
| + | 183 | ENSRNOT00000041562 |
| - | 119 | ENSRNOT00000041621 |
| - | 67 | ENSRNOT00000044101 |
| - | 4 | ENSRNOT00000044101 |
| - | 83 | ENSRNOT00000044101 |
| - | 25 | ENSRNOT00000044101 |

| | | |
|---|---|---|
| - | 76 | ENSRNOT00000044101 |
| - | 37 | ENSRNOT00000044101 |
| - | 136 | ENSRNOT00000045096 |
| - | 329 | ENSRNOT00000052466 |
| + | 100 | ENSRNOT00000053436 |
| + | 37 | ENSRNOT00000053436 |
| + | 37 | ENSRNOT00000053436 |
| + | 186 | ENSRNOT00000054626 |
| - | 273 | ENSRNOT00000055537 |
| + | 108 | ENSRNOT00000030805 |
| + | 52 | ENSRNOT00000056189 |
| - | 588 | ENSRNOT00000048323 |
| - | 2528 | ENSRNOT00000039881 |
| - | 2528 | ENSRNOT00000048323 |
| - | 588 | ENSRNOT00000039881 |
| - | 2 | ENSRNOT00000056545 |
| - | 21 | ENSRNOT00000056545 |
| - | 35 | ENSRNOT00000056545 |
| - | 65 | ENSRNOT00000058362 |
| - | 65 | ENSRNOT00000058362 |
| + | 164 | ENSRNOT00000044620 |
| - | 27 | ENSRNOT00000059896 |
| - | 325 | ENSRNOT00000047460 |
| + | 253 | ENSRNOT00000060766 |
| + | 171 | ENSRNOT00000061184 |
| - | 345 | ENSRNOT00000061847 |
| - | 2092 | ENSRNOT00000062085 |
| + | 138 | ENSRNOT00000062668 |
| + | 352 | ENSRNOT00000063224 |
| + | 248 | ENSRNOT00000063309 |
| + | 155 | ENSRNOT00000063363 |
| - | 2528 | ENSRNOT00000068767 |
| - | 588 | ENSRNOT00000068767 |
| - | 438 | ENSRNOT00000036574 |
| - | 438 | ENSRNOT00000068611 |
| - | 438 | ENSRNOT00000066225 |
| - | 184 | ENSRNOT00000068511 |
| + | 99 | ENSRNOT00000065039 |

| | | |
|---|---|---|
| + | 226 | ENSRNOT00000065039 |
| + | 211 | ENSRNOT00000064004 |
| + | 98 | ENSRNOT00000070178 |
| - | 52 | ENSRNOT00000070402 |

ıries (Rat Genome RN4)

| Overlapping gene | | |
|---|---|---|
| *Overlapping Gene ID* | *Gene symbol (RN4)* | *Gene description (RN4)* |
| ENSRNOG00000003276 | *Myo1d* | Myosin-Id |
| ENSRNOG00000028149 | *LOC367289* | Uncharacterized protein |
| ENSRNOG00000000522 | *Cpne5* | copine-5 |
| ENSRNOG00000001861 | *Ydjc* | YdjC homolog |
| ENSRNOG00000015078 | *Ifitm3* | Interferon-induced transmembra |
| ENSRNOG00000026857 | *Kif7* | Uncharacterized protein |
| ENSRNOG00000019451 | *Smtn* | smoothelin |
| ENSRNOG00000019451 | *Smtn* | smoothelin |
| ENSRNOG00000019451 | *Smtn* | smoothelin |
| ENSRNOG00000019451 | *Smtn* | smoothelin |
| ENSRNOG00000015089 | *Mcoln2* | mucolipin-2 |
| ENSRNOG00000040109 | *Treml1* | trem-like transcript 1 protein |
| ENSRNOG00000019902 | *Folr1* | folate receptor alpha |
| ENSRNOG00000020837 | *D4A4H1_RAT* | Uncharacterized protein |
| ENSRNOG00000020702 | *Cybasc3* | Cytochrome b ascorbate-depende |
| ENSRNOG00000033110 | *Svep1* | Sushi |
| ENSRNOG00000033110 | *Svep1* | Sushi |
| ENSRNOG00000013552 | *Scd1* | Acyl-CoA desaturase 1 |
| ENSRNOG00000013190 | *Rnaset2* | ribonuclease T2 |
| ENSRNOG00000005515 | *Rhbdl3* | rhomboid-related protein 3 |
| ENSRNOG00000003276 | *Myo1d* | Myosin-Id |
| ENSRNOG00000015024 | *E9PT54_RAT* | mucolipin-3 |
| ENSRNOG00000015024 | *E9PT54_RAT* | mucolipin-3 |
| ENSRNOG00000013615 | *Dntt* | DNA nucleotidylexotransferase |
| ENSRNOG00000013615 | *Dntt* | DNA nucleotidylexotransferase |
| ENSRNOG00000012886 | *Maff* | transcription factor MafF |
| ENSRNOG00000001861 | *Ydjc* | YdjC homolog |
| ENSRNOG00000005124 | *Plekhh2* | pleckstrin homology domain-cont |
| ENSRNOG00000005291 | *Slc38a1* | Sodium-coupled neutral amino ac |
| ENSRNOG00000005291 | *Slc38a1* | Sodium-coupled neutral amino ac |
| ENSRNOG00000019451 | *Smtn* | smoothelin |
| ENSRNOG00000019451 | *Smtn* | smoothelin |
| ENSRNOG00000037621 | *RGD1309870* | -- |

| | | |
|---|---|---|
| ENSRNOG00000014104 | *Myo5b* | Myosin-Vb |
| ENSRNOG00000014104 | *Myo5b* | Myosin-Vb |
| ENSRNOG00000014104 | *Myo5b* | Myosin-Vb |
| ENSRNOG00000012531 | *Ephb2* | ephrin type-B receptor 2 |
| ENSRNOG00000012531 | *Ephb2* | ephrin type-B receptor 2 |
| ENSRNOG00000012531 | *Ephb2* | ephrin type-B receptor 2 |
| ENSRNOG00000010378 | *Slc4a5* | Electrogenic sodium bicarbonate |
| ENSRNOG00000010378 | *Slc4a5* | Electrogenic sodium bicarbonate |
| ENSRNOG00000021840 | *LOC500046* | Uncharacterized protein |
| ENSRNOG00000021840 | *LOC500046* | Uncharacterized protein |
| ENSRNOG00000021840 | *LOC500046* | Uncharacterized protein |
| ENSRNOG00000008990 | *F1LZQ6_RAT* | Angiomotin-like 1 (Predicted)Unc |
| ENSRNOG00000008990 | *F1LZQ6_RAT* | Angiomotin-like 1 (Predicted)Unc |
| ENSRNOG00000015089 | *Mcoln2* | mucolipin-2 |
| ENSRNOG00000040109 | *Treml1* | trem-like transcript 1 protein |
| ENSRNOG00000020624 | *Acadsb* | Short/branched chain specific acy |
| ENSRNOG00000006963 | *Ctse* | Cathepsin E |
| ENSRNOG00000006963 | *Ctse* | Cathepsin E |
| ENSRNOG00000017087 | *Man1c1* | mannosyl-oligosaccharide 1 |
| ENSRNOG00000017087 | *Man1c1* | mannosyl-oligosaccharide 1 |
| ENSRNOG00000017087 | *Man1c1* | mannosyl-oligosaccharide 1 |
| ENSRNOG00000017087 | *Man1c1* | mannosyl-oligosaccharide 1 |
| ENSRNOG00000017202 | *Gcom1* | GRINL1A complex locus protein 1 |
| ENSRNOG00000017202 | *Gcom1* | GRINL1A complex locus protein 1 |
| ENSRNOG00000014969 | *Lzts2* | Leucine zipper putative tumor su[illegible] |
| ENSRNOG00000028149 | *LOC367289* | Uncharacterized protein |
| ENSRNOG00000028149 | *LOC367289* | Uncharacterized protein |
| ENSRNOG00000028149 | *LOC367289* | Uncharacterized protein |
| ENSRNOG00000020837 | *D4A4H1_RAT* | Uncharacterized protein |
| ENSRNOG00000020797 | *D3ZSK0_RAT* | Uncharacterized protein |
| ENSRNOG00000020797 | *D3ZSK0_RAT* | Uncharacterized protein |
| ENSRNOG00000004458 | *Ston2* | stonin-2 |
| ENSRNOG00000018126 | *F1LNL3_RAT* | ATP-binding cassette sub-family [illegible] |
| ENSRNOG00000018126 | *F1LNL3_RAT* | ATP-binding cassette sub-family [illegible] |
| ENSRNOG00000033110 | *Svep1* | Sushi |
| ENSRNOG00000013552 | *Scd1* | Acyl-CoA desaturase 1 |
| ENSRNOG00000013552 | *Scd1* | Acyl-CoA desaturase 1 |
| ENSRNOG00000013552 | *Scd1* | Acyl-CoA desaturase 1 |

| | | |
|---|---|---|
| ENSRNOG00000010131 | *Rin2* | ras and Rab interactor 2 |
| ENSRNOG00000010131 | *Rin2* | ras and Rab interactor 2 |
| ENSRNOG00000027841 | *D3ZGN7_RAT* | Uncharacterized protein |
| ENSRNOG00000010873 | *RGD1308048* | Uncharacterized protein |
| ENSRNOG00000006649 | *Thrb* | Thyroid hormone receptor beta |
| ENSRNOG00000006649 | *Thrb* | Thyroid hormone receptor beta |
| ENSRNOG00000001931 | *Fgf12* | Fibroblast growth factor 12 |
| ENSRNOG00000016038 | *Mgmt* | Methylated-DNA--protein-cystein |
| ENSRNOG00000016038 | *Mgmt* | Methylated-DNA--protein-cystein |
| ENSRNOG00000019656 | *Btd* | Biotinidase |
| ENSRNOG00000028344 | *Mmp11* | Stromelysin-3 |
| ENSRNOG00000000522 | *Cpne5* | copine-5 |
| ENSRNOG00000000522 | *Cpne5* | copine-5 |
| ENSRNOG00000006720 | *Rnpep* | Aminopeptidase B |
| ENSRNOG00000006720 | *Rnpep* | Aminopeptidase B |
| ENSRNOG00000001861 | *Ydjc* | YdjC homolog |
| ENSRNOG00000001861 | *Ydjc* | YdjC homolog |
| ENSRNOG00000001861 | *Ydjc* | YdjC homolog |
| ENSRNOG00000015078 | *Ifitm3* | Interferon-induced transmembra |
| ENSRNOG00000011970 | *Tmem82* | Putative uncharacterized protein |
| ENSRNOG00000017804 | *Sept1* | Septin-1 |
| ENSRNOG00000035334 | *U1* | U1 spliceosomal RNA |
| ENSRNOG00000020837 | *D4A4H1_RAT* | Uncharacterized protein |
| ENSRNOG00000033110 | *Svep1* | Sushi |
| ENSRNOG00000019902 | *Folr1* | folate receptor alpha |
| ENSRNOG00000020702 | *Cybasc3* | Cytochrome b ascorbate-depend[illegible] |
| ENSRNOG00000001861 | *Ydjc* | YdjC homolog |
| ENSRNOG00000015078 | *Ifitm3* | Interferon-induced transmembra |
| ENSRNOG00000020837 | *D4A4H1_RAT* | Uncharacterized protein |
| ENSRNOG00000022790 | *F1M8C9_RAT* | Uncharacterized protein |
| ENSRNOG00000022790 | *F1M8C9_RAT* | Uncharacterized protein |
| ENSRNOG00000022790 | *F1M8C9_RAT* | Uncharacterized protein |
| ENSRNOG00000022790 | *F1M8C9_RAT* | Uncharacterized protein |
| ENSRNOG00000022790 | *F1M8C9_RAT* | Uncharacterized protein |
| ENSRNOG00000022790 | *F1M8C9_RAT* | Uncharacterized protein |
| ENSRNOG00000011852 | *Myo6* | Uncharacterized protein |
| ENSRNOG00000011852 | *Myo6* | Uncharacterized protein |
| ENSRNOG00000019271 | *Ulk4* | Uncharacterized protein |

| | | |
|---|---|---|
| ENSRNOG00000019271 | *Ulk4* | Uncharacterized protein |
| ENSRNOG00000019271 | *Ulk4* | Uncharacterized protein |
| ENSRNOG00000019271 | *Ulk4* | Uncharacterized protein |
| ENSRNOG00000018185 | *Sag* | S-arrestin |
| ENSRNOG00000013521 | *Dhfr* | Dihydrofolate reductase |
| ENSRNOG00000004200 | *Golsyn* | Uncharacterized protein |
| ENSRNOG00000017342 | *Zdhhc7* | Palmitoyltransferase ZDHHC7 |
| ENSRNOG00000006783 | *F1M1J1_RAT* | Nebulin (Predicted)Uncharacteriz |
| ENSRNOG00000016413 | *Pstpip1* | proline-serine-threonine phospha |
| ENSRNOG00000010208 | *Timp1* | Metalloproteinase inhibitor 1 |
| ENSRNOG00000010208 | *Timp1* | Metalloproteinase inhibitor 1 |
| ENSRNOG00000007713 | *Tmcc3* | transmembrane and coiled-coil d |
| ENSRNOG00000028236 | *RGD1309104* | Uncharacterized protein |
| ENSRNOG00000028236 | *RGD1309104* | Uncharacterized protein |
| ENSRNOG00000011028 | *D3ZV66_RAT* | Uncharacterized protein |
| ENSRNOG00000011028 | *D3ZV66_RAT* | Uncharacterized protein |
| ENSRNOG00000011028 | *D3ZV66_RAT* | Uncharacterized protein |
| ENSRNOG00000012110 | *Col17a1* | collagen alpha-1(XVII) chain |
| ENSRNOG00000012110 | *Col17a1* | collagen alpha-1(XVII) chain |
| ENSRNOG00000020284 | *Prkar2a* | cAMP-dependent protein kinase t |
| ENSRNOG00000014526 | *D4A5Z3_RAT* | Uncharacterized protein |
| ENSRNOG00000014526 | *D4A5Z3_RAT* | Uncharacterized protein |
| ENSRNOG00000018321 | *Sytl3* | synaptotagmin-like protein 3 |
| ENSRNOG00000025994 | *Krt80* | Keratin |
| ENSRNOG00000025994 | *Krt80* | Keratin |
| ENSRNOG00000025994 | *Krt80* | Keratin |
| ENSRNOG00000025994 | *Krt80* | Keratin |
| ENSRNOG00000001795 | *D4AC54_RAT* | integrin |
| ENSRNOG00000001795 | *D4AC54_RAT* | integrin |
| ENSRNOG00000001795 | *D4AC54_RAT* | integrin |
| ENSRNOG00000006646 | *Ecop* | EGFR-coamplified and overexpres |
| ENSRNOG00000006646 | *Ecop* | EGFR-coamplified and overexpres |
| ENSRNOG00000014747 | *F1LX73_RAT* | Uncharacterized protein |
| ENSRNOG00000014747 | *F1LX73_RAT* | Uncharacterized protein |
| ENSRNOG00000001817 | *Tm7sf3* | Transmembrane 7 superfamily m |
| ENSRNOG00000034303 | *Spon1* | Spondin-1 |
| ENSRNOG00000034303 | *Spon1* | Spondin-1 |
| ENSRNOG00000011847 | *Grk4* | G protein-coupled receptor kinas |

| | | |
|---|---|---|
| ENSRNOG00000011847 | *Grk4* | G protein-coupled receptor kinas |
| ENSRNOG00000016516 | *MBP_RAT* | Myelin basic protein S |
| ENSRNOG00000016516 | *MBP_RAT* | Myelin basic protein S |
| ENSRNOG00000010753 | *Aig1* | androgen-induced gene 1 protein |
| ENSRNOG00000010753 | *Aig1* | androgen-induced gene 1 protein |
| ENSRNOG00000010753 | *Aig1* | androgen-induced gene 1 protein |
| ENSRNOG00000015367 | *D3ZYR3_RAT* | Uncharacterized protein |
| ENSRNOG00000015367 | *D3ZYR3_RAT* | Uncharacterized protein |
| ENSRNOG00000015367 | *D3ZYR3_RAT* | Uncharacterized protein |
| ENSRNOG00000003054 | *Cask* | Peripheral plasma membrane pro |
| ENSRNOG00000003054 | *Cask* | Peripheral plasma membrane pro |
| ENSRNOG00000003054 | *Cask* | Peripheral plasma membrane pro |
| ENSRNOG00000003054 | *Cask* | Peripheral plasma membrane pro |
| ENSRNOG00000003054 | *Cask* | Peripheral plasma membrane pro |
| ENSRNOG00000003054 | *Cask* | Peripheral plasma membrane pro |
| ENSRNOG00000018778 | *Cadm1* | cell adhesion molecule 1 |
| ENSRNOG00000010011 | *Osbpl3* | Oxysterol-binding protein |
| ENSRNOG00000010011 | *Osbpl3* | Oxysterol-binding protein |
| ENSRNOG00000010011 | *Osbpl3* | Oxysterol-binding protein |
| ENSRNOG00000019351 | *Chid1* | Chitinase domain-containing prot |
| ENSRNOG00000019351 | *Chid1* | Chitinase domain-containing prot |
| ENSRNOG00000019351 | *Chid1* | Chitinase domain-containing prot |
| ENSRNOG00000033627 | *Panx2* | Pannexin-2 |
| ENSRNOG00000033627 | *Panx2* | Pannexin-2 |
| ENSRNOG00000033627 | *Panx2* | Pannexin-2 |
| ENSRNOG00000033627 | *Panx2* | Pannexin-2 |
| ENSRNOG00000033627 | *Panx2* | Pannexin-2 |
| ENSRNOG00000033627 | *Panx2* | Pannexin-2 |
| ENSRNOG00000033627 | *Panx2* | Pannexin-2 |
| ENSRNOG00000033627 | *Panx2* | Pannexin-2 |
| ENSRNOG00000014705 | *Rbm20* | probable RNA-binding protein 20 |
| ENSRNOG00000014705 | *Rbm20* | probable RNA-binding protein 20 |
| ENSRNOG00000014705 | *Rbm20* | probable RNA-binding protein 20 |
| ENSRNOG00000011386 | *RGD1308124* | Uncharacterized protein |
| ENSRNOG00000011386 | *RGD1308124* | Uncharacterized protein |
| ENSRNOG00000007660 | *Fntb* | Protein farnesyltransferase subur |
| ENSRNOG00000021440 | *Pptc7* | protein phosphatase PTC7 homol |
| ENSRNOG00000032437 | *Pard3* | Partitioning defective 3 homolog |

| | | |
|---|---|---|
| ENSRNOG00000032437 | *Pard3* | Partitioning defective 3 homolog |
| ENSRNOG00000032437 | *Pard3* | Partitioning defective 3 homolog |
| ENSRNOG00000032437 | *Pard3* | Partitioning defective 3 homolog |
| ENSRNOG00000011503 | *RGD1565350* | Uncharacterized protein |
| ENSRNOG00000042788 | *F1M260_RAT* | Uncharacterized protein |
| ENSRNOG00000042788 | *F1M260_RAT* | Uncharacterized protein |
| ENSRNOG00000002408 | *Rbm47* | RNA-binding protein 47 |
| ENSRNOG00000042740 | *Mrpl42* | 28S ribosomal protein L42 |
| ENSRNOG00000023991 | *Rab20* | ras-related protein Rab-20 |
| ENSRNOG00000001706 | *Kalrn* | Kalirin |
| ENSRNOG00000001706 | *Kalrn* | Kalirin |
| ENSRNOG00000001706 | *Kalrn* | Kalirin |
| ENSRNOG00000001706 | *Kalrn* | Kalirin |
| ENSRNOG00000023589 | *Gpr107* | protein GPR107 |
| ENSRNOG00000007533 | *Rab27a* | Ras-related protein Rab-27A |
| ENSRNOG00000017020 | *Inpp5d* | Phosphatidylinositol-3 |
| ENSRNOG00000017020 | *Inpp5d* | Phosphatidylinositol-3 |
| ENSRNOG00000026316 | *Wdr43* | WD repeat domain 43 |
| ENSRNOG00000013248 | *Wwc2* | protein WWC2 |
| ENSRNOG00000013248 | *Wwc2* | protein WWC2 |
| ENSRNOG00000013248 | *Wwc2* | protein WWC2 |
| ENSRNOG00000015084 | *Necab2* | N-terminal EF-hand calcium-bindi |
| ENSRNOG00000032941 | *Mab21l1* | protein mab-21-like 1 |
| ENSRNOG00000012325 | *Adk* | Adenosine kinase |
| ENSRNOG00000012325 | *Adk* | Adenosine kinase |
| ENSRNOG00000008758 | *Tspan18* | tetraspanin-18 |
| ENSRNOG00000010487 | *Dnajb14* | dnaJ homolog subfamily B memb |
| ENSRNOG00000001169 | *Slc37a1* | glycerol-3-phosphate transporter |
| ENSRNOG00000027097 | *F1M683_RAT* | Uncharacterized protein |
| ENSRNOG00000001104 | *Foxk1* | forkhead box K1 |
| ENSRNOG00000036690 | *Dysfip1* | dysferlin-interacting protein 1 |
| ENSRNOG00000024620 | *F1M9P7_RAT* | Uncharacterized protein |
| ENSRNOG00000017120 | *Abhd2* | abhydrolase domain-containing p |
| ENSRNOG00000010286 | *Cast* | Calpastatin |
| ENSRNOG00000010286 | *Cast* | Calpastatin |
| ENSRNOG00000010286 | *Cast* | Calpastatin |
| ENSRNOG00000010286 | *Cast* | Calpastatin |
| ENSRNOG00000000916 | *Katnal1* | Katanin p60 ATPase-containing su |

| | | |
|---|---|---|
| ENSRNOG00000031168 | *Arhgap15* | Rho GTPase-activating protein 15 |
| ENSRNOG00000015054 | *Rcbtb2* | RCC1 and BTB domain-containing |
| ENSRNOG00000020656 | *Pfkfb4* | 6-phosphofructo-2-kinase/fructo: |
| ENSRNOG00000020656 | *Pfkfb4* | 6-phosphofructo-2-kinase/fructo: |
| ENSRNOG00000020656 | *Pfkfb4* | 6-phosphofructo-2-kinase/fructo: |
| ENSRNOG00000008015 | *Fos* | Proto-oncogene c-Fos |
| ENSRNOG00000008015 | *Fos* | Proto-oncogene c-Fos |
| ENSRNOG00000014903 | *Zfyve27* | zinc finger FYVE domain-containir |
| ENSRNOG00000014903 | *Zfyve27* | zinc finger FYVE domain-containir |
| ENSRNOG00000024136 | *Fam151b* | family with sequence similarity 1! |
| ENSRNOG00000006079 | *Psd4* | PH and SEC7 domain-containing p |
| ENSRNOG00000006384 | *Ddx58* | DEAD (Asp-Glu-Ala-Asp) box polyp |
| ENSRNOG00000006384 | *Ddx58* | DEAD (Asp-Glu-Ala-Asp) box polyp |
| ENSRNOG00000006384 | *Ddx58* | DEAD (Asp-Glu-Ala-Asp) box polyp |
| ENSRNOG00000006384 | *Ddx58* | DEAD (Asp-Glu-Ala-Asp) box polyp |
| ENSRNOG00000018760 | *Mpp7* | MAGUK p55 subfamily member 7 |
| ENSRNOG00000018760 | *Mpp7* | MAGUK p55 subfamily member 7 |
| ENSRNOG00000010512 | *Yipf1* | Protein YIPF1 |
| ENSRNOG00000010512 | *Yipf1* | Protein YIPF1 |
| ENSRNOG00000010512 | *Yipf1* | Protein YIPF1 |
| ENSRNOG00000006231 | *Ptpro* | receptor-type tyrosine-protein ph |
| ENSRNOG00000006231 | *Ptpro* | receptor-type tyrosine-protein ph |
| ENSRNOG00000019213 | *Gpd1* | Glycerol-3-phosphate dehydroger |
| ENSRNOG00000019213 | *Gpd1* | Glycerol-3-phosphate dehydroger |
| ENSRNOG00000028460 | *Lrrc8e* | Leucine-rich repeat-containing pr |
| ENSRNOG00000001138 | *Taok3* | Serine/threonine-protein kinase T |
| ENSRNOG00000013340 | *Twsg1* | twisted gastrulation homolog 1 |
| ENSRNOG00000026061 | *RGD1566226* | Putative uncharacterized protein |
| ENSRNOG00000013252 | *Sdccag10* | Peptidyl-prolyl cis-trans isomeras |
| ENSRNOG00000019228 | *Pik3r2* | Phosphatidylinositol 3-kinase regu |
| ENSRNOG00000019228 | *Pik3r2* | Phosphatidylinositol 3-kinase regu |
| ENSRNOG00000019228 | *Pik3r2* | Phosphatidylinositol 3-kinase regu |
| ENSRNOG00000000991 | *Arpc1b* | Actin-related protein 2/3 complex |
| ENSRNOG00000001227 | *Adarb1* | Double-stranded RNA-specific edi |
| ENSRNOG00000007574 | *Padi2* | Protein-arginine deiminase type-2 |
| ENSRNOG00000004709 | *Foxn3* | forkhead box protein N3 |
| ENSRNOG00000013333 | *D3ZDF2_RAT* | Uncharacterized protein |
| ENSRNOG00000013333 | *D3ZDF2_RAT* | Uncharacterized protein |

| | | |
|---|---|---|
| ENSRNOG00000013322 | *DPOLA_RAT* | DNA polymerase alpha catalytic s |
| ENSRNOG00000008474 | *Acox3* | Peroxisomal acyl-coenzyme A oxi |
| ENSRNOG00000008474 | *Acox3* | Peroxisomal acyl-coenzyme A oxi |
| ENSRNOG00000008474 | *Acox3* | Peroxisomal acyl-coenzyme A oxi |
| ENSRNOG00000001309 | *Camkk2* | Calcium/calmodulin-dependent p |
| ENSRNOG00000001309 | *Camkk2* | Calcium/calmodulin-dependent p |
| ENSRNOG00000001309 | *Camkk2* | Calcium/calmodulin-dependent p |
| ENSRNOG00000001309 | *Camkk2* | Calcium/calmodulin-dependent p |
| ENSRNOG00000001309 | *Camkk2* | Calcium/calmodulin-dependent p |
| ENSRNOG00000019346 | *Tmco3* | transmembrane and coiled-coil d |
| ENSRNOG00000008815 | *Fbxo46* | F-box only protein 46 |
| ENSRNOG00000025818 | *D3ZYP0_RAT* | Uncharacterized protein |
| ENSRNOG00000019948 | *Phf20* | PHD finger protein 20 |
| ENSRNOG00000019948 | *Phf20* | PHD finger protein 20 |
| ENSRNOG00000032768 | *D3Z9G8_RAT* | syntaxin-binding protein 4 |
| ENSRNOG00000002898 | *Nme7* | Nucleoside diphosphate kinase 7 |
| ENSRNOG00000043167 | *Itga9* | Uncharacterized protein |
| ENSRNOG00000005930 | *Nnmt* | nicotinamide N-methyltransferas |
| ENSRNOG00000012818 | *Ksr1* | kinase suppressor of Ras 1 |
| ENSRNOG00000012818 | *Ksr1* | kinase suppressor of Ras 1 |
| ENSRNOG00000012818 | *Ksr1* | kinase suppressor of Ras 1 |
| ENSRNOG00000017132 | *D4A060_RAT* | sorting nexin-30 |
| ENSRNOG00000017132 | *D4A060_RAT* | sorting nexin-30 |
| ENSRNOG00000017132 | *D4A060_RAT* | sorting nexin-30 |
| ENSRNOG00000017132 | *D4A060_RAT* | sorting nexin-30 |
| ENSRNOG00000018342 | *D3ZD47_RAT* | Uncharacterized protein |
| ENSRNOG00000010365 | *Syn1* | Synapsin-1 |
| ENSRNOG00000010365 | *Syn1* | Synapsin-1 |
| ENSRNOG00000013780 | *NF1_RAT* | Neurofibromin |
| ENSRNOG00000013780 | *NF1_RAT* | Neurofibromin |
| ENSRNOG00000013780 | *NF1_RAT* | Neurofibromin |
| ENSRNOG00000013780 | *NF1_RAT* | Neurofibromin |
| ENSRNOG00000024414 | *Ate1* | arginyl-tRNA--protein transferase |
| ENSRNOG00000024414 | *Ate1* | arginyl-tRNA--protein transferase |
| ENSRNOG00000024414 | *Ate1* | arginyl-tRNA--protein transferase |
| ENSRNOG00000024414 | *Ate1* | arginyl-tRNA--protein transferase |
| ENSRNOG00000036814 | *D4ACY5_RAT* | Uncharacterized protein |
| ENSRNOG00000036814 | *D4ACY5_RAT* | Uncharacterized protein |

| | | |
|---|---|---|
| ENSRNOG00000036814 | *D4ACY5_RAT* | Uncharacterized protein |
| ENSRNOG00000036814 | *D4ACY5_RAT* | Uncharacterized protein |
| ENSRNOG00000011254 | *Fam76a* | family with sequence similarity 76 |
| ENSRNOG00000011254 | *Fam76a* | family with sequence similarity 76 |
| ENSRNOG00000011254 | *Fam76a* | family with sequence similarity 76 |
| ENSRNOG00000042519 | *RGD1312026* | similar to RIKEN cDNA C230081A1 |
| ENSRNOG00000003931 | *Arsg* | Arylsulfatase G |
| ENSRNOG00000010065 | *D4ADL8_RAT* | Uncharacterized protein |
| ENSRNOG00000010065 | *D4ADL8_RAT* | Uncharacterized protein |
| ENSRNOG00000014000 | *Kif2a* | Kinesin-like protein KIF2A |
| ENSRNOG00000014000 | *Kif2a* | Kinesin-like protein KIF2A |
| ENSRNOG00000020706 | *Kcnn3* | Small conductance calcium-activa |
| ENSRNOG00000024632 | *Atf6* | cyclic AMP-dependent transcripti |
| ENSRNOG00000020193 | *Runx2* | Runt-related transcription factor |
| ENSRNOG00000020193 | *Runx2* | Runt-related transcription factor |
| ENSRNOG00000015145 | *Ppp6c* | Serine/threonine-protein phosph |
| ENSRNOG00000018425 | *Dym* | Dymeclin |
| ENSRNOG00000018425 | *Dym* | Dymeclin |
| ENSRNOG00000018400 | *D4AEL2_RAT* | Uncharacterized protein |
| ENSRNOG00000014806 | *F1M1E4_RAT* | myofibrillogenesis regulator 1 iso |
| ENSRNOG00000014806 | *F1M1E4_RAT* | myofibrillogenesis regulator 1 iso |
| ENSRNOG00000014806 | *F1M1E4_RAT* | myofibrillogenesis regulator 1 iso |
| ENSRNOG00000025216 | *Alkbh6* | probable alpha-ketoglutarate-dep |
| ENSRNOG00000025216 | *Alkbh6* | probable alpha-ketoglutarate-dep |
| ENSRNOG00000033593 | *Osbpl9* | oxysterol binding protein-like 9 |
| ENSRNOG00000033593 | *Osbpl9* | oxysterol binding protein-like 9 |
| ENSRNOG00000033593 | *Osbpl9* | oxysterol binding protein-like 9 |
| ENSRNOG00000024533 | *Aer61* | Uncharacterized glycosyltransfera |
| ENSRNOG00000015677 | *Cda* | cytidine deaminase |
| ENSRNOG00000002122 | *Lrrc8c* | Leucine-rich repeat-containing pr |
| ENSRNOG00000005183 | *Rtf1* | RNA polymerase-associated prote |
| ENSRNOG00000005183 | *Rtf1* | RNA polymerase-associated prote |
| ENSRNOG00000005183 | *Rtf1* | RNA polymerase-associated prote |
| ENSRNOG00000013946 | *D3ZI66_RAT* | Uncharacterized protein |
| ENSRNOG00000038079 | *Uba2* | ubiquitin-like modifier activating |
| ENSRNOG00000038079 | *Uba2* | ubiquitin-like modifier activating |
| ENSRNOG00000017463 | *Bloc1s3* | Uncharacterized protein |
| ENSRNOG00000023760 | *Plekhm3* | Uncharacterized protein |

| | | |
|---|---|---|
| ENSRNOG00000023760 | *Plekhm3* | Uncharacterized protein |
| ENSRNOG00000011752 | *Sh3d19* | Similar to SH3 domain protein D1 |
| ENSRNOG00000000695 | *F1LWM1_RAT* | Uncharacterized protein |
| ENSRNOG00000000695 | *F1LWM1_RAT* | Uncharacterized protein |
| ENSRNOG00000032055 | *Spats2* | spermatogenesis-associated serir |
| ENSRNOG00000032055 | *Spats2* | spermatogenesis-associated serir |
| ENSRNOG00000027002 | *D4A8R8_RAT* | Uncharacterized protein |
| ENSRNOG00000016897 | *Rlbp1* | retinaldehyde-binding protein 1 |
| ENSRNOG00000015932 | *RGD1306839* | similar to RIKEN cDNA 5033414D( |
| ENSRNOG00000034298 | *F1LXE0_RAT* | Uncharacterized protein |
| ENSRNOG00000034298 | *F1LXE0_RAT* | Uncharacterized protein |
| ENSRNOG00000034298 | *F1LXE0_RAT* | Uncharacterized protein |
| ENSRNOG00000034298 | *F1LXE0_RAT* | Uncharacterized protein |
| ENSRNOG00000007246 | *Atxn7* | Uncharacterized protein |
| ENSRNOG00000030216 | *Abcg3l1* | ATP-binding cassette |
| ENSRNOG00000030216 | *Abcg3l1* | ATP-binding cassette |
| ENSRNOG00000011321 | *Rftn1* | raft-linking protein |
| ENSRNOG00000026059 | *Paqr6* | progestin and adipoQ receptor fa |
| ENSRNOG00000026059 | *Paqr6* | progestin and adipoQ receptor fa |
| ENSRNOG00000026059 | *Paqr6* | progestin and adipoQ receptor fa |
| ENSRNOG00000003821 | *D3ZDU2_RAT* | raptor |
| ENSRNOG00000012405 | *Tcf4* | Transcription factor 4 |
| ENSRNOG00000012405 | *Tcf4* | Transcription factor 4 |
| ENSRNOG00000014616 | *Iars* | isoleucyl-tRNA synthetase |
| ENSRNOG00000014616 | *Iars* | isoleucyl-tRNA synthetase |
| ENSRNOG00000005984 | *Etv6* | transcription factor ETV6 |
| ENSRNOG00000005984 | *Etv6* | transcription factor ETV6 |
| ENSRNOG00000019299 | *Edem2* | ER degradation-enhancing alpha- |
| ENSRNOG00000018649 | *Slc35c2* | solute carrier family 35 member ( |
| ENSRNOG00000018649 | *Slc35c2* | solute carrier family 35 member ( |
| ENSRNOG00000018649 | *Slc35c2* | solute carrier family 35 member ( |
| ENSRNOG00000018649 | *Slc35c2* | solute carrier family 35 member ( |
| ENSRNOG00000018649 | *Slc35c2* | solute carrier family 35 member ( |
| ENSRNOG00000018649 | *Slc35c2* | solute carrier family 35 member ( |
| ENSRNOG00000022929 | *MTMRC_RAT* | Myotubularin-related protein 12 |
| ENSRNOG00000022929 | *MTMRC_RAT* | Myotubularin-related protein 12 |
| ENSRNOG00000005234 | *Glt8d3* | Glucoside xylosyltransferase 1 |
| ENSRNOG00000005234 | *Glt8d3* | Glucoside xylosyltransferase 1 |

| | | |
|---|---|---|
| ENSRNOG00000005234 | *Glt8d3* | Glucoside xylosyltransferase 1 |
| ENSRNOG00000002040 | *Bmp2k* | Uncharacterized protein |
| ENSRNOG00000002040 | *Bmp2k* | Uncharacterized protein |
| ENSRNOG00000005483 | *Rpap1* | RNA polymerase II-associated pro |
| ENSRNOG00000011435 | *Osbpl10* | Oxysterol-binding protein |
| ENSRNOG00000011435 | *Osbpl10* | Oxysterol-binding protein |
| ENSRNOG00000024352 | *Tyw1* | tRNA wybutosine-synthesizing pr |
| ENSRNOG00000033234 | *Ccrl2* | C-C chemokine receptor-like 2 |
| ENSRNOG00000000567 | *Unc5b* | Netrin receptor UNC5B |
| ENSRNOG00000000567 | *Unc5b* | Netrin receptor UNC5B |
| ENSRNOG00000000567 | *Unc5b* | Netrin receptor UNC5B |
| ENSRNOG00000000567 | *Unc5b* | Netrin receptor UNC5B |
| ENSRNOG00000009980 | *Ppap2a* | Lipid phosphate phosphohydrolas |
| ENSRNOG00000009980 | *Ppap2a* | Lipid phosphate phosphohydrolas |
| ENSRNOG00000009980 | *Ppap2a* | Lipid phosphate phosphohydrolas |
| ENSRNOG00000001289 | *LOC498154* | Uncharacterized protein C7orf50 |
| ENSRNOG00000001289 | *LOC498154* | Uncharacterized protein C7orf50 |
| ENSRNOG00000001289 | *LOC498154* | Uncharacterized protein C7orf50 |
| ENSRNOG00000001289 | *LOC498154* | Uncharacterized protein C7orf50 |
| ENSRNOG00000001304 | *Bcr* | Uncharacterized protein |
| ENSRNOG00000001304 | *Bcr* | Uncharacterized protein |
| ENSRNOG00000001242 | *Gstt3* | Glutathione S-transferase theta-1 |
| ENSRNOG00000001242 | *Gstt3* | Glutathione S-transferase theta-1 |
| ENSRNOG00000012561 | *F1M0Z4_RAT* | Uncharacterized protein |
| ENSRNOG00000012363 | *Peli2* | protein pellino homolog 2 |
| ENSRNOG00000012363 | *Peli2* | protein pellino homolog 2 |
| ENSRNOG00000013875 | *Fbxl17* | F-box/LRR-repeat protein 17 |
| ENSRNOG00000016696 | *Angpt2* | Angiopoietin-2 |
| ENSRNOG00000016696 | *Angpt2* | Angiopoietin-2 |
| ENSRNOG00000034025 | *D4A4T5_RAT* | Uncharacterized protein |
| ENSRNOG00000034025 | *D4A4T5_RAT* | Uncharacterized protein |
| ENSRNOG00000034025 | *D4A4T5_RAT* | Uncharacterized protein |
| ENSRNOG00000034025 | *D4A4T5_RAT* | Uncharacterized protein |
| ENSRNOG00000034025 | *D4A4T5_RAT* | Uncharacterized protein |
| ENSRNOG00000034025 | *D4A4T5_RAT* | Uncharacterized protein |
| ENSRNOG00000034025 | *D4A4T5_RAT* | Uncharacterized protein |
| ENSRNOG00000034025 | *D4A4T5_RAT* | Uncharacterized protein |
| ENSRNOG00000042096 | *LOC100366081* | RCG54037Uncharacterized protei |

| | | |
|---|---|---|
| ENSRNOG00000013323 | *Rnf217* | probable E3 ubiquitin-protein liga |
| ENSRNOG00000010227 | *RGD1308907* | similar to FLJ20689 (RGD1308907 |
| ENSRNOG00000010227 | *RGD1308907* | similar to FLJ20689 (RGD1308907 |
| ENSRNOG00000028426 | *Mcf2l* | Guanine nucleotide exchange fac |
| ENSRNOG00000028426 | *Mcf2l* | Guanine nucleotide exchange fac |
| ENSRNOG00000028426 | *Mcf2l* | Guanine nucleotide exchange fac |
| ENSRNOG00000028426 | *Mcf2l* | Guanine nucleotide exchange fac |
| ENSRNOG00000013135 | *Ptpn12* | tyrosine-protein phosphatase nor |
| ENSRNOG00000013135 | *Ptpn12* | tyrosine-protein phosphatase nor |
| ENSRNOG00000016306 | *Gpr98* | Uncharacterized protein |
| ENSRNOG00000016306 | *Gpr98* | Uncharacterized protein |
| ENSRNOG00000009708 | *Tas1r1* | Taste receptor type 1 member 1 |
| ENSRNOG00000008904 | *Fli1* | Friend leukemia integration 1 trai |
| ENSRNOG00000008904 | *Fli1* | Friend leukemia integration 1 trai |
| ENSRNOG00000013436 | *Pde7b* | cAMP-specific 3' |
| ENSRNOG00000013436 | *Pde7b* | cAMP-specific 3' |
| ENSRNOG00000013962 | *Magi2* | Membrane-associated guanylate |
| ENSRNOG00000013962 | *Magi2* | Membrane-associated guanylate |
| ENSRNOG00000013962 | *Magi2* | Membrane-associated guanylate |
| ENSRNOG00000013962 | *Magi2* | Membrane-associated guanylate |
| ENSRNOG00000007310 | *Klrb1a* | Killer cell lectin-like receptor subf |
| ENSRNOG00000010155 | *Pcm1* | pericentriolar material 1 |
| ENSRNOG00000010155 | *Pcm1* | pericentriolar material 1 |
| ENSRNOG00000002749 | *E9PU51_RAT* | C-Jun-amino-terminal kinase-inte |
| ENSRNOG00000002749 | *E9PU51_RAT* | C-Jun-amino-terminal kinase-inte |
| ENSRNOG00000002749 | *E9PU51_RAT* | C-Jun-amino-terminal kinase-inte |
| ENSRNOG00000002749 | *E9PU51_RAT* | C-Jun-amino-terminal kinase-inte |
| ENSRNOG00000002749 | *E9PU51_RAT* | C-Jun-amino-terminal kinase-inte |
| ENSRNOG00000002749 | *E9PU51_RAT* | C-Jun-amino-terminal kinase-inte |
| ENSRNOG00000002749 | *E9PU51_RAT* | C-Jun-amino-terminal kinase-inte |
| ENSRNOG00000019140 | *Banp* | protein BANP |
| ENSRNOG00000019140 | *Banp* | protein BANP |
| ENSRNOG00000019140 | *Banp* | protein BANP |
| ENSRNOG00000001288 | *Gpr146* | probable G-protein coupled recep |
| ENSRNOG00000012016 | *Cdig2* | Niemann-Pick disease |
| ENSRNOG00000009726 | *Grip2* | Glutamate receptor-interacting p |
| ENSRNOG00000009726 | *Grip2* | Glutamate receptor-interacting p |
| ENSRNOG00000009726 | *Grip2* | Glutamate receptor-interacting p |

| | | |
|---|---|---|
| ENSRNOG00000016544 | *Rgnef* | Rho-guanine nucleotide exchange |
| ENSRNOG00000016544 | *Rgnef* | Rho-guanine nucleotide exchange |
| ENSRNOG00000000661 | *Hps4* | Hermansky-Pudlak syndrome 4 ho |
| ENSRNOG00000010813 | *Tspan14* | tetraspanin-14 |
| ENSRNOG00000033425 | *F1M2T8_RAT* | Uncharacterized protein |
| ENSRNOG00000021380 | *Fads6* | fatty acid desaturase 6 |
| ENSRNOG00000021380 | *Fads6* | fatty acid desaturase 6 |
| ENSRNOG00000015455 | *Spr* | Sepiapterin reductase |
| ENSRNOG00000001123 | *RGD1562310* | Putative uncharacterized protein |
| ENSRNOG00000010630 | *Prcp* | lysosomal Pro-X carboxypeptidase |
| ENSRNOG00000002146 | *Pkd2* | polycystin-2 |
| ENSRNOG00000002146 | *Pkd2* | polycystin-2 |
| ENSRNOG00000002146 | *Pkd2* | polycystin-2 |
| ENSRNOG00000002146 | *Pkd2* | polycystin-2 |
| ENSRNOG00000015821 | *Cd2* | T-cell surface antigen CD2 |
| ENSRNOG00000015821 | *Cd2* | T-cell surface antigen CD2 |
| ENSRNOG00000002256 | *Art3* | ecto-ADP-ribosyltransferase 3 |
| ENSRNOG00000020799 | *Polr2i* | DNA-directed RNA polymerase II s |
| ENSRNOG00000022312 | *Lrsam1* | E3 ubiquitin-protein ligase LRSAM |
| ENSRNOG00000022312 | *Lrsam1* | E3 ubiquitin-protein ligase LRSAM |
| ENSRNOG00000022312 | *Lrsam1* | E3 ubiquitin-protein ligase LRSAM |
| ENSRNOG00000022312 | *Lrsam1* | E3 ubiquitin-protein ligase LRSAM |
| ENSRNOG00000003138 | *Fcgr2a* | Low affinity immunoglobulin gam |
| ENSRNOG00000017940 | *Rere* | Arginine-glutamic acid dipeptide r |
| ENSRNOG00000017940 | *Rere* | Arginine-glutamic acid dipeptide r |
| ENSRNOG00000017940 | *Rere* | Arginine-glutamic acid dipeptide r |
| ENSRNOG00000011614 | *Tmcc1* | Uncharacterized protein |
| ENSRNOG00000009276 | *Schip1* | schwannomin interacting protein |
| ENSRNOG00000009276 | *Schip1* | schwannomin interacting protein |
| ENSRNOG00000008658 | *Mitf* | Microphthalmia-associated transc |
| ENSRNOG00000008658 | *Mitf* | Microphthalmia-associated transc |
| ENSRNOG00000008658 | *Mitf* | Microphthalmia-associated transc |
| ENSRNOG00000008658 | *Mitf* | Microphthalmia-associated transc |
| ENSRNOG00000008658 | *Mitf* | Microphthalmia-associated transc |
| ENSRNOG00000000599 | *F1LTF8_RAT* | Uncharacterized protein |
| ENSRNOG00000016610 | *Arhgap1* | Rho GTPase activating protein 1 |
| ENSRNOG00000016610 | *Arhgap1* | Rho GTPase activating protein 1 |
| ENSRNOG00000007474 | *ST7* | Suppressor of tumorigenicity 7 pr |

| | | |
|---|---|---|
| ENSRNOG00000007474 | *ST7* | Suppressor of tumorigenicity 7 pr |
| ENSRNOG00000007474 | *ST7* | Suppressor of tumorigenicity 7 pr |
| ENSRNOG00000008116 | *Ppap2b* | Lipid phosphate phosphohydrolas |
| ENSRNOG00000023238 | *Dgkd* | Uncharacterized protein |
| ENSRNOG00000016257 | *Cotl1* | Coactosin-like protein |
| ENSRNOG00000024345 | *Pard3b* | partitioning defective 3 homolog |
| ENSRNOG00000024345 | *Pard3b* | partitioning defective 3 homolog |
| ENSRNOG00000015021 | *Carkd* | carbohydrate kinase domain-cont |
| ENSRNOG00000015021 | *Carkd* | carbohydrate kinase domain-cont |
| ENSRNOG00000005262 | *F1LUM3_RAT* | Uncharacterized protein |
| ENSRNOG00000006027 | *Eif2ak4* | eukaryotic translation initiation fa |
| ENSRNOG00000006027 | *Eif2ak4* | eukaryotic translation initiation fa |
| ENSRNOG00000028895 | *Rtp4* | receptor-transporting protein 4 |
| ENSRNOG00000042920 | *Aldh4a1* | Delta-1-pyrroline-5-carboxylate d |
| ENSRNOG00000042920 | *Aldh4a1* | Delta-1-pyrroline-5-carboxylate d |
| ENSRNOG00000010109 | *Nol9* | Nol9 protein |
| ENSRNOG00000022664 | *Myst2* | Histone acetyltransferase MYST2 |
| ENSRNOG00000022664 | *Myst2* | Histone acetyltransferase MYST2 |
| ENSRNOG00000022664 | *Myst2* | Histone acetyltransferase MYST2 |
| ENSRNOG00000022664 | *Myst2* | Histone acetyltransferase MYST2 |
| ENSRNOG00000022664 | *Myst2* | Histone acetyltransferase MYST2 |
| ENSRNOG00000012318 | *Aspm* | asp (abnormal spindle) homolog |
| ENSRNOG00000012318 | *Aspm* | asp (abnormal spindle) homolog |
| ENSRNOG00000012318 | *Aspm* | asp (abnormal spindle) homolog |
| ENSRNOG00000020830 | *RGD1304580* | Nesprin-4 |
| ENSRNOG00000020830 | *RGD1304580* | Nesprin-4 |
| ENSRNOG00000017123 | *B2m* | Beta-2-microglobulin |
| ENSRNOG00000017123 | *B2m* | Beta-2-microglobulin |
| ENSRNOG00000011969 | *Dock9* | Dedicator of cytokinesis protein 9 |
| ENSRNOG00000011969 | *Dock9* | Dedicator of cytokinesis protein 9 |
| ENSRNOG00000011969 | *Dock9* | Dedicator of cytokinesis protein 9 |
| ENSRNOG00000011969 | *Dock9* | Dedicator of cytokinesis protein 9 |
| ENSRNOG00000011969 | *Dock9* | Dedicator of cytokinesis protein 9 |
| ENSRNOG00000011969 | *Dock9* | Dedicator of cytokinesis protein 9 |
| ENSRNOG00000011969 | *Dock9* | Dedicator of cytokinesis protein 9 |
| ENSRNOG00000011969 | *Dock9* | Dedicator of cytokinesis protein 9 |
| ENSRNOG00000011969 | *Dock9* | Dedicator of cytokinesis protein 9 |
| ENSRNOG00000011969 | *Dock9* | Dedicator of cytokinesis protein 9 |

| | | |
|---|---|---|
| ENSRNOG00000011969 | *Dock9* | Dedicator of cytokinesis protein 9 |
| ENSRNOG00000011969 | *Dock9* | Dedicator of cytokinesis protein 9 |
| ENSRNOG00000011969 | *Dock9* | Dedicator of cytokinesis protein 9 |
| ENSRNOG00000013177 | *Map3k1* | Mitogen-activated protein kinase |
| ENSRNOG00000013177 | *Map3k1* | Mitogen-activated protein kinase |
| ENSRNOG00000021526 | *Slc25a34* | Solute carrier family 25 member 3 |
| ENSRNOG00000026978 | *Oscp1* | Protein OSCP1 |
| ENSRNOG00000026978 | *Oscp1* | Protein OSCP1 |
| ENSRNOG00000010219 | *GNDS_RAT* | Ral guanine nucleotide dissociatic |
| ENSRNOG00000010219 | *GNDS_RAT* | Ral guanine nucleotide dissociatic |
| ENSRNOG00000040148 | *F1M4U8_RAT* | Uncharacterized protein |
| ENSRNOG00000040148 | *F1M4U8_RAT* | Uncharacterized protein |
| ENSRNOG00000011700 | *Ptprm* | receptor-type tyrosine-protein ph |
| ENSRNOG00000011700 | *Ptprm* | receptor-type tyrosine-protein ph |
| ENSRNOG00000001713 | -- | -- |
| ENSRNOG00000001713 | -- | -- |
| ENSRNOG00000001713 | -- | -- |
| ENSRNOG00000036697 | *Mafg* | Transcription factor MafG |
| ENSRNOG00000002559 | *Cacna1a* | Voltage-dependent P/Q-type calc |
| ENSRNOG00000002559 | *Cacna1a* | Voltage-dependent P/Q-type calc |
| ENSRNOG00000002559 | *Cacna1a* | Voltage-dependent P/Q-type calc |
| ENSRNOG00000002559 | *Cacna1a* | Voltage-dependent P/Q-type calc |
| ENSRNOG00000025479 | *F1LVW3_RAT* | Uncharacterized protein |
| ENSRNOG00000014522 | *Mlycd* | Malonyl-CoA decarboxylase |
| ENSRNOG00000008169 | *Slc24a2* | Sodium/potassium/calcium excha |
| ENSRNOG00000008169 | *Slc24a2* | Sodium/potassium/calcium excha |
| ENSRNOG00000017628 | *Tagln* | Transgelin |
| ENSRNOG00000012932 | *LOC499980* | Coiled-coil domain-containing prc |
| ENSRNOG00000000940 | *Flt1* | Vascular endothelial growth facto |
| ENSRNOG00000001047 | *Map2k7* | Dual specificity mitogen-activatec |
| ENSRNOG00000001047 | *Map2k7* | Dual specificity mitogen-activatec |
| ENSRNOG00000004104 | *E9PTG0_RAT* | zinc finger protein 167 |
| ENSRNOG00000036675 | *Btbd16* | BTB/POZ domain-containing prot |
| ENSRNOG00000036675 | *Btbd16* | BTB/POZ domain-containing prot |
| ENSRNOG00000036675 | *Btbd16* | BTB/POZ domain-containing prot |
| ENSRNOG00000036675 | *Btbd16* | BTB/POZ domain-containing prot |
| ENSRNOG00000018382 | *Inpp4b* | Type II inositol-3 |
| ENSRNOG00000018382 | *Inpp4b* | Type II inositol-3 |

| | | |
|---|---|---|
| ENSRNOG00000030542 | *D4AEE8_RAT* | Uncharacterized protein |
| ENSRNOG00000019012 | *D2hgdh* | D-2-hydroxyglutarate dehydroger |
| ENSRNOG00000008314 | *Orc3l* | Origin recognition complex subur |
| ENSRNOG00000000596 | *Fyn* | Tyrosine-protein kinase Fyn |
| ENSRNOG00000000596 | *Fyn* | Tyrosine-protein kinase Fyn |
| ENSRNOG00000028394 | *Vpreb3* | pre-B lymphocyte protein 3 |
| ENSRNOG00000002347 | *Rgl1* | ral guanine nucleotide dissociatio |
| ENSRNOG00000002347 | *Rgl1* | ral guanine nucleotide dissociatio |
| ENSRNOG00000023376 | *Riok3* | serine/threonine-protein kinase F |
| ENSRNOG00000025290 | *RGD1560603* | Uncharacterized protein |
| ENSRNOG00000043562 | -- | -- |
| ENSRNOG00000017671 | *Rasa3* | Ras GTPase-activating protein 3 |
| ENSRNOG00000017671 | *Rasa3* | Ras GTPase-activating protein 3 |
| ENSRNOG00000009085 | *Prkag2* | 5'-AMP-activated protein kinase s |
| ENSRNOG00000009085 | *Prkag2* | 5'-AMP-activated protein kinase s |
| ENSRNOG00000003042 | *Mettl13* | methyltransferase-like protein 13 |
| ENSRNOG00000003042 | *Mettl13* | methyltransferase-like protein 13 |
| ENSRNOG00000003042 | *Mettl13* | methyltransferase-like protein 13 |
| ENSRNOG00000014264 | *Phactr1* | Phosphatase and actin regulator [illegible] |
| ENSRNOG00000014264 | *Phactr1* | Phosphatase and actin regulator [illegible] |
| ENSRNOG00000014264 | *Phactr1* | Phosphatase and actin regulator [illegible] |
| ENSRNOG00000014264 | *Phactr1* | Phosphatase and actin regulator [illegible] |
| ENSRNOG00000018839 | *Ntrk2* | BDNF/NT-3 growth factors recept |
| ENSRNOG00000018839 | *Ntrk2* | BDNF/NT-3 growth factors recept |
| ENSRNOG00000018839 | *Ntrk2* | BDNF/NT-3 growth factors recept |
| ENSRNOG00000018839 | *Ntrk2* | BDNF/NT-3 growth factors recept |
| ENSRNOG00000018839 | *Ntrk2* | BDNF/NT-3 growth factors recept |
| ENSRNOG00000018839 | *Ntrk2* | BDNF/NT-3 growth factors recept |
| ENSRNOG00000018839 | *Ntrk2* | BDNF/NT-3 growth factors recept |
| ENSRNOG00000017673 | *Brsk1* | BR serine/threonine-protein kinas |
| ENSRNOG00000009956 | *WNK1_RAT* | Serine/threonine-protein kinase \ |
| ENSRNOG00000009956 | *WNK1_RAT* | Serine/threonine-protein kinase \ |
| ENSRNOG00000009956 | *WNK1_RAT* | Serine/threonine-protein kinase \ |
| ENSRNOG00000024457 | *Cyct* | Cytochrome c |
| ENSRNOG00000024457 | *Cyct* | Cytochrome c |
| ENSRNOG00000024457 | *Cyct* | Cytochrome c |
| ENSRNOG00000024457 | *Cyct* | Cytochrome c |
| ENSRNOG00000024457 | *Cyct* | Cytochrome c |

| | | |
|---|---|---|
| ENSRNOG00000024457 | *Cyct* | Cytochrome c |
| ENSRNOG00000024457 | *Cyct* | Cytochrome c |
| ENSRNOG00000024457 | *Cyct* | Cytochrome c |
| ENSRNOG00000024457 | *Cyct* | Cytochrome c |
| ENSRNOG00000024457 | *Cyct* | Cytochrome c |
| ENSRNOG00000024457 | *Cyct* | Cytochrome c |
| ENSRNOG00000024457 | *Cyct* | Cytochrome c |
| ENSRNOG00000024457 | *Cyct* | Cytochrome c |
| ENSRNOG00000024457 | *Cyct* | Cytochrome c |
| ENSRNOG00000024457 | *Cyct* | Cytochrome c |
| ENSRNOG00000036798 | *Dusp3* | dual specificity phosphatase 3 |
| ENSRNOG00000016511 | *Pitrm1* | presequence protease |
| ENSRNOG00000016511 | *Pitrm1* | presequence protease |
| ENSRNOG00000016511 | *Pitrm1* | presequence protease |
| ENSRNOG00000043203 | *K2C75_RAT* | Keratin |
| ENSRNOG00000043203 | *K2C75_RAT* | Keratin |
| ENSRNOG00000013351 | *STXB5_RAT* | Syntaxin-binding protein 5 |
| ENSRNOG00000013351 | *STXB5_RAT* | Syntaxin-binding protein 5 |
| ENSRNOG00000013351 | *STXB5_RAT* | Syntaxin-binding protein 5 |
| ENSRNOG00000013388 | *Pgap1* | GPI inositol-deacylase |
| ENSRNOG00000015898 | *Fert2* | Tyrosine-protein kinase Fer |
| ENSRNOG00000015898 | *Fert2* | Tyrosine-protein kinase Fer |
| ENSRNOG00000015898 | *Fert2* | Tyrosine-protein kinase Fer |
| ENSRNOG00000015898 | *Fert2* | Tyrosine-protein kinase Fer |
| ENSRNOG00000015898 | *Fert2* | Tyrosine-protein kinase Fer |
| ENSRNOG00000015898 | *Fert2* | Tyrosine-protein kinase Fer |
| ENSRNOG00000019623 | *D3ZQW0_RAT* | Uncharacterized protein |
| ENSRNOG00000019623 | *D3ZQW0_RAT* | Uncharacterized protein |
| ENSRNOG00000019623 | *D3ZQW0_RAT* | Uncharacterized protein |
| ENSRNOG00000019623 | *D3ZQW0_RAT* | Uncharacterized protein |
| ENSRNOG00000008786 | *Ap1b1* | AP-1 complex subunit beta-1 |
| ENSRNOG00000008786 | *Ap1b1* | AP-1 complex subunit beta-1 |
| ENSRNOG00000008786 | *Ap1b1* | AP-1 complex subunit beta-1 |
| ENSRNOG00000013933 | *Med27* | mediator of RNA polymerase II tr |
| ENSRNOG00000018413 | *Per3* | Period circadian protein homolog |
| ENSRNOG00000018413 | *Per3* | Period circadian protein homolog |
| ENSRNOG00000018413 | *Per3* | Period circadian protein homolog |
| ENSRNOG00000018413 | *Per3* | Period circadian protein homolog |

| | | |
|---|---|---|
| ENSRNOG00000036585 | *F1LN76_RAT* | Uncharacterized protein |
| ENSRNOG00000014079 | *Stat4* | signal transducer and activator of |
| ENSRNOG00000014079 | *Stat4* | signal transducer and activator of |
| ENSRNOG00000014241 | *Ece1* | Endothelin-converting enzyme 1 |
| ENSRNOG00000014241 | *Ece1* | Endothelin-converting enzyme 1 |
| ENSRNOG00000015514 | *Bcat1* | Branched-chain-amino-acid amin |
| ENSRNOG00000015514 | *Bcat1* | Branched-chain-amino-acid amin |
| ENSRNOG00000015514 | *Bcat1* | Branched-chain-amino-acid amin |
| ENSRNOG00000015514 | *Bcat1* | Branched-chain-amino-acid amin |
| ENSRNOG00000007330 | *Capzb* | F-actin-capping protein subunit b |
| ENSRNOG00000007330 | *Capzb* | F-actin-capping protein subunit b |
| ENSRNOG00000007330 | *Capzb* | F-actin-capping protein subunit b |
| ENSRNOG00000008279 | *Mrps15* | 28S ribosomal protein S15 |
| ENSRNOG00000014504 | *Il1r1* | Interleukin-1 receptor type 1 |
| ENSRNOG00000014504 | *Il1r1* | Interleukin-1 receptor type 1 |
| ENSRNOG00000014504 | *Il1r1* | Interleukin-1 receptor type 1 |
| ENSRNOG00000001804 | *ITPR2_RAT* | Inositol 1 |
| ENSRNOG00000001804 | *ITPR2_RAT* | Inositol 1 |
| ENSRNOG00000001804 | *ITPR2_RAT* | Inositol 1 |
| ENSRNOG00000001804 | *ITPR2_RAT* | Inositol 1 |
| ENSRNOG00000003901 | *Ccdc104* | Coiled-coil domain-containing pro |
| ENSRNOG00000003901 | *Ccdc104* | Coiled-coil domain-containing pro |
| ENSRNOG00000003901 | *Ccdc104* | Coiled-coil domain-containing pro |
| ENSRNOG00000003901 | *Ccdc104* | Coiled-coil domain-containing pro |
| ENSRNOG00000003901 | *Ccdc104* | Coiled-coil domain-containing pro |
| ENSRNOG00000003901 | *Ccdc104* | Coiled-coil domain-containing pro |
| ENSRNOG00000000373 | *LOC100362857* | Uncharacterized protein |
| ENSRNOG00000001141 | *Srrm4* | Uncharacterized protein |
| ENSRNOG00000001174 | *Pde9a* | high affinity cGMP-specific 3' |
| ENSRNOG00000001174 | *Pde9a* | high affinity cGMP-specific 3' |
| ENSRNOG00000001298 | *F1LQV2_RAT* | Uncharacterized protein |
| ENSRNOG00000001618 | *Ripk4* | receptor-interacting serine/threo |
| ENSRNOG00000001658 | *IRK6_RAT* | G protein-activated inward rectifi |
| ENSRNOG00000001658 | *IRK6_RAT* | G protein-activated inward rectifi |
| ENSRNOG00000003741 | *Nptx1* | Neuronal pentraxin-1 |
| ENSRNOG00000004516 | *Itgbl1* | Integrin beta-like protein 1 |
| ENSRNOG00000004516 | *Itgbl1* | Integrin beta-like protein 1 |
| ENSRNOG00000004928 | *Sntg2* | gamma-2-syntrophin |

| | | |
|---|---|---|
| ENSRNOG00000004928 | *Sntg2* | gamma-2-syntrophin |
| ENSRNOG00000004966 | *Slc38a11* | Uncharacterized protein |
| ENSRNOG00000004966 | *Slc38a11* | Uncharacterized protein |
| ENSRNOG00000004966 | *Slc38a11* | Uncharacterized protein |
| ENSRNOG00000004966 | *Slc38a11* | Uncharacterized protein |
| ENSRNOG00000004966 | *Slc38a11* | Uncharacterized protein |
| ENSRNOG00000004966 | *Slc38a11* | Uncharacterized protein |
| ENSRNOG00000004994 | *Agr3* | anterior gradient protein 3 homo |
| ENSRNOG00000004994 | *Agr3* | anterior gradient protein 3 homo |
| ENSRNOG00000004994 | *Agr3* | anterior gradient protein 3 homo |
| ENSRNOG00000005451 | *E9PU24_RAT* | Uncharacterized protein |
| ENSRNOG00000006226 | *Cacng2* | voltage-dependent calcium chanr |
| ENSRNOG00000006890 | *LOC100365021* | Uncharacterized protein |
| ENSRNOG00000006890 | *LOC100365021* | Uncharacterized protein |
| ENSRNOG00000006891 | *Avpr1b* | Vasopressin V1b receptor |
| ENSRNOG00000006891 | *Avpr1b* | Vasopressin V1b receptor |
| ENSRNOG00000006957 | *Gria4* | Glutamate receptor 4 |
| ENSRNOG00000006957 | *Gria4* | Glutamate receptor 4 |
| ENSRNOG00000006957 | *Gria4* | Glutamate receptor 4 |
| ENSRNOG00000007108 | *Bmp7* | bone morphogenetic protein 7 |
| ENSRNOG00000007185 | *D3ZCP4_RAT* | leiomodin-1 |
| ENSRNOG00000007575 | *Lppr1* | Lipid phosphate phosphatase-rela |
| ENSRNOG00000007843 | *Wnt2* | Protein Wnt |
| ENSRNOG00000008065 | *Wwc1* | Uncharacterized protein |
| ENSRNOG00000008570 | *Cadps* | Calcium-dependent secretion acti |
| ENSRNOG00000008570 | *Cadps* | Calcium-dependent secretion acti |
| ENSRNOG00000008570 | *Cadps* | Calcium-dependent secretion acti |
| ENSRNOG00000008570 | *Cadps* | Calcium-dependent secretion acti |
| ENSRNOG00000008761 | *Freq* | Neuronal calcium sensor 1 |
| ENSRNOG00000008766 | *Grin2b* | Glutamate |
| ENSRNOG00000009771 | *Cdh10* | cadherin-10 |
| ENSRNOG00000011184 | *Slc13a4* | solute carrier family 13 member 4 |
| ENSRNOG00000011184 | *Slc13a4* | solute carrier family 13 member 4 |
| ENSRNOG00000011203 | *Farp1* | FERM |
| ENSRNOG00000011203 | *Farp1* | FERM |
| ENSRNOG00000011475 | *Snip* | SRC kinase signaling inhibitor 1 |
| ENSRNOG00000011475 | *Snip* | SRC kinase signaling inhibitor 1 |
| ENSRNOG00000011475 | *Snip* | SRC kinase signaling inhibitor 1 |

| | | |
|---|---|---|
| ENSRNOG00000011475 | *Snip* | SRC kinase signaling inhibitor 1 |
| ENSRNOG00000012189 | *Lect2* | leukocyte cell-derived chemotaxi |
| ENSRNOG00000012189 | *Lect2* | leukocyte cell-derived chemotaxi |
| ENSRNOG00000012342 | *Ttc29* | Tetratricopeptide repeat protein |
| ENSRNOG00000012817 | *Lrrc17* | leucine rich repeat containing 17 |
| ENSRNOG00000012817 | *Lrrc17* | leucine rich repeat containing 17 |
| ENSRNOG00000012906 | *Bcas1* | Breast carcinoma-amplified seque |
| ENSRNOG00000012906 | *Bcas1* | Breast carcinoma-amplified seque |
| ENSRNOG00000012906 | *Bcas1* | Breast carcinoma-amplified seque |
| ENSRNOG00000012906 | *Bcas1* | Breast carcinoma-amplified seque |
| ENSRNOG00000012906 | *Bcas1* | Breast carcinoma-amplified seque |
| ENSRNOG00000013391 | *Sorbs2* | Sorbin and SH3 domain-containin |
| ENSRNOG00000013391 | *Sorbs2* | Sorbin and SH3 domain-containin |
| ENSRNOG00000013391 | *Sorbs2* | Sorbin and SH3 domain-containin |
| ENSRNOG00000013391 | *Sorbs2* | Sorbin and SH3 domain-containin |
| ENSRNOG00000013391 | *Sorbs2* | Sorbin and SH3 domain-containin |
| ENSRNOG00000013391 | *Sorbs2* | Sorbin and SH3 domain-containin |
| ENSRNOG00000013391 | *Sorbs2* | Sorbin and SH3 domain-containin |
| ENSRNOG00000014775 | *Nol4* | nucleolar protein 4 |
| ENSRNOG00000015058 | *F1M245_RAT* | Uncharacterized protein |
| ENSRNOG00000015058 | *F1M245_RAT* | Uncharacterized protein |
| ENSRNOG00000015149 | *F1M0L6_RAT* | pro-neuregulin-4 |
| ENSRNOG00000016043 | *Aqp4* | Aquaporin-4 |
| ENSRNOG00000016374 | *Fgfr2* | fibroblast growth factor receptor |
| ENSRNOG00000016374 | *Fgfr2* | fibroblast growth factor receptor |
| ENSRNOG00000016374 | *Fgfr2* | fibroblast growth factor receptor |
| ENSRNOG00000016374 | *Fgfr2* | fibroblast growth factor receptor |
| ENSRNOG00000016374 | *Fgfr2* | fibroblast growth factor receptor |
| ENSRNOG00000016374 | *Fgfr2* | fibroblast growth factor receptor |
| ENSRNOG00000016472 | *42067* | Uncharacterized protein |
| ENSRNOG00000016483 | *Myo16* | Myosin-XVI |
| ENSRNOG00000016483 | *Myo16* | Myosin-XVI |
| ENSRNOG00000016483 | *Myo16* | Myosin-XVI |
| ENSRNOG00000016712 | *Lhcgr* | Lutropin-choriogonadotropic hor |
| ENSRNOG00000016712 | *Lhcgr* | Lutropin-choriogonadotropic hor |
| ENSRNOG00000016712 | *Lhcgr* | Lutropin-choriogonadotropic hor |
| ENSRNOG00000016712 | *Lhcgr* | Lutropin-choriogonadotropic hor |
| ENSRNOG00000016712 | *Lhcgr* | Lutropin-choriogonadotropic hor |

| | | |
|---|---|---|
| ENSRNOG00000016712 | *Lhcgr* | Lutropin-choriogonadotropic hor |
| ENSRNOG00000016712 | *Lhcgr* | Lutropin-choriogonadotropic hor |
| ENSRNOG00000016712 | *Lhcgr* | Lutropin-choriogonadotropic hor |
| ENSRNOG00000016712 | *Lhcgr* | Lutropin-choriogonadotropic hor |
| ENSRNOG00000016712 | *Lhcgr* | Lutropin-choriogonadotropic hor |
| ENSRNOG00000016783 | *Fshr* | Follicle-stimulating hormone rece |
| ENSRNOG00000016783 | *Fshr* | Follicle-stimulating hormone rece |
| ENSRNOG00000016887 | *Hrh4* | Histamine H4 receptor |
| ENSRNOG00000017712 | *Cartpt* | Cocaine- and amphetamine-regul |
| ENSRNOG00000017712 | *Cartpt* | Cocaine- and amphetamine-regul |
| ENSRNOG00000018602 | *F1M4M6_RAT* | calmodulin binding transcription |
| ENSRNOG00000018602 | *F1M4M6_RAT* | calmodulin binding transcription |
| ENSRNOG00000018602 | *F1M4M6_RAT* | calmodulin binding transcription |
| ENSRNOG00000018607 | *Slc12a3* | Solute carrier family 12 member |
| ENSRNOG00000018739 | *Pax7* | paired box protein Pax-7 |
| ENSRNOG00000019613 | *Syt9* | Synaptotagmin-9 |
| ENSRNOG00000020138 | *Slc4a3* | Anion exchange protein 3 |
| ENSRNOG00000020138 | *Slc4a3* | Anion exchange protein 3 |
| ENSRNOG00000020138 | *Slc4a3* | Anion exchange protein 3 |
| ENSRNOG00000020532 | *Kcnq1* | Potassium voltage-gated channel |
| ENSRNOG00000020532 | *Kcnq1* | Potassium voltage-gated channel |
| ENSRNOG00000021169 | *RGD1562196* | -- |
| ENSRNOG00000021566 | *F1M3H5_RAT* | Uncharacterized protein |
| ENSRNOG00000021984 | *Rgs7* | Regulator of G-protein signaling 7 |
| ENSRNOG00000021984 | *Rgs7* | Regulator of G-protein signaling 7 |
| ENSRNOG00000022297 | *Kcnmb4* | Calcium-activated potassium cha |
| ENSRNOG00000022420 | *F1M7E6_RAT* | Uncharacterized protein |
| ENSRNOG00000024127 | *Atp13a5* | probable cation-transporting ATP |
| ENSRNOG00000024657 | *Mfsd4* | major facilitator superfamily dom |
| ENSRNOG00000024657 | *Mfsd4* | major facilitator superfamily dom |
| ENSRNOG00000024657 | *Mfsd4* | major facilitator superfamily dom |
| ENSRNOG00000024657 | *Mfsd4* | major facilitator superfamily dom |
| ENSRNOG00000024657 | *Mfsd4* | major facilitator superfamily dom |
| ENSRNOG00000024657 | *Mfsd4* | major facilitator superfamily dom |
| ENSRNOG00000024657 | *Mfsd4* | major facilitator superfamily dom |
| ENSRNOG00000024880 | *D3ZKH1_RAT* | Uncharacterized protein |
| ENSRNOG00000025365 | *LOC684861* | Uncharacterized protein |
| ENSRNOG00000025365 | *LOC684861* | Uncharacterized protein |

| | | |
|---|---|---|
| ENSRNOG00000025480 | *LOC684368* | Uncharacterized protein |
| ENSRNOG00000025776 | *Ccdc148* | coiled-coil domain-containing pro |
| ENSRNOG00000025889 | *GNAS3_RAT* | Neuroendocrine secretory protei |
| ENSRNOG00000025889 | *GNAS3_RAT* | Neuroendocrine secretory protei |
| ENSRNOG00000025889 | *GNAS3_RAT* | Neuroendocrine secretory protei |
| ENSRNOG00000025889 | *GNAS3_RAT* | Neuroendocrine secretory protei |
| ENSRNOG00000027954 | *Cdh15* | cadherin-15 |
| ENSRNOG00000028225 | *Tnni3k* | Serine/threonine-protein kinase T |
| ENSRNOG00000028281 | -- | -- |
| ENSRNOG00000028630 | *F1LY04_RAT* | Uncharacterized protein |
| ENSRNOG00000029933 | *F1M8Z1_RAT* | Uncharacterized protein |
| ENSRNOG00000030060 | *F1LYB4_RAT* | Uncharacterized protein |
| ENSRNOG00000030060 | *F1LYB4_RAT* | Uncharacterized protein |
| ENSRNOG00000030098 | *D3ZVM4_RAT* | tripartite motif-containing protei |
| ENSRNOG00000030547 | *Dpp6* | Dipeptidyl aminopeptidase-like pr |
| ENSRNOG00000030547 | *Dpp6* | Dipeptidyl aminopeptidase-like pr |
| ENSRNOG00000031100 | *Klhl1* | kelch-like protein 1 |
| ENSRNOG00000031100 | *Klhl1* | kelch-like protein 1 |
| ENSRNOG00000031189 | *Ido1* | Indoleamine 2 |
| ENSRNOG00000031782 | *F1M6H4_RAT* | Uncharacterized protein |
| ENSRNOG00000031782 | *F1M6H4_RAT* | Uncharacterized protein |
| ENSRNOG00000031921 | *F1M2T6_RAT* | Uncharacterized protein |
| ENSRNOG00000031922 | *F1LVN6_RAT* | Uncharacterized protein |
| ENSRNOG00000031922 | *F1LVN6_RAT* | Uncharacterized protein |
| ENSRNOG00000031922 | *F1LVN6_RAT* | Uncharacterized protein |
| ENSRNOG00000031922 | *F1LVN6_RAT* | Uncharacterized protein |
| ENSRNOG00000031922 | *F1LVN6_RAT* | Uncharacterized protein |
| ENSRNOG00000033026 | *Dclk3* | serine/threonine-protein kinase D |
| ENSRNOG00000033162 | *Chia* | Acidic mammalian chitinase |
| ENSRNOG00000033162 | *Chia* | Acidic mammalian chitinase |
| ENSRNOG00000033162 | *Chia* | Acidic mammalian chitinase |
| ENSRNOG00000033162 | *Chia* | Acidic mammalian chitinase |
| ENSRNOG00000033162 | *Chia* | Acidic mammalian chitinase |
| ENSRNOG00000033274 | *RGD1565415* | Uncharacterized protein |
| ENSRNOG00000033892 | *F1LY54_RAT* | Uncharacterized protein |
| ENSRNOG00000033892 | *F1LY54_RAT* | Uncharacterized protein |
| ENSRNOG00000033892 | *F1LY54_RAT* | Uncharacterized protein |
| ENSRNOG00000033892 | *F1LY54_RAT* | Uncharacterized protein |

| | | |
|---|---|---|
| ENSRNOG00000033892 | *F1LY54_RAT* | Uncharacterized protein |
| ENSRNOG00000033892 | *F1LY54_RAT* | Uncharacterized protein |
| ENSRNOG00000034177 | *Efna5* | Ephrin-A5 |
| ENSRNOG00000034343 | *U6* | U6 spliceosomal RNA |
| ENSRNOG00000035313 | *U6* | U6 spliceosomal RNA |
| ENSRNOG00000035313 | *U6* | U6 spliceosomal RNA |
| ENSRNOG00000035313 | *U6* | U6 spliceosomal RNA |
| ENSRNOG00000036519 | -- | -- |
| ENSRNOG00000036958 | *RGD1311728* | Uncharacterized protein |
| ENSRNOG00000037164 | *LOC100361340* | Uncharacterized protein |
| ENSRNOG00000037202 | *F1LZP6_RAT* | Uncharacterized protein |
| ENSRNOG00000037315 | *RGD1559714* | TRAF domain and POZ/BTB conta |
| ENSRNOG00000037315 | *RGD1559714* | TRAF domain and POZ/BTB conta |
| ENSRNOG00000037315 | *RGD1559714* | TRAF domain and POZ/BTB conta |
| ENSRNOG00000037315 | *RGD1559714* | TRAF domain and POZ/BTB conta |
| ENSRNOG00000037367 | *F1LW06_RAT* | Uncharacterized protein |
| ENSRNOG00000037367 | *F1LW06_RAT* | Uncharacterized protein |
| ENSRNOG00000037367 | *F1LW06_RAT* | Uncharacterized protein |
| ENSRNOG00000038328 | *Gjc2* | gap junction protein |
| ENSRNOG00000038328 | *Gjc2* | gap junction protein |
| ENSRNOG00000038721 | *D3ZJN6_RAT* | Uncharacterized protein |
| ENSRNOG00000039152 | *Frem3* | FRAS1-related extracellular matri |
| ENSRNOG00000039526 | *F1LZ12_RAT* | Uncharacterized protein |
| ENSRNOG00000039629 | *F1M9D7_RAT* | Uncharacterized protein |
| ENSRNOG00000039855 | *F1M6C9_RAT* | Uncharacterized protein |
| ENSRNOG00000040188 | *Lcn3* | Uncharacterized protein |
| ENSRNOG00000040337 | *D3ZKK1_RAT* | Uncharacterized protein |
| ENSRNOG00000040915 | *SNORD24* | Small nucleolar RNA SNORD24 |
| ENSRNOG00000041471 | -- | -- |
| ENSRNOG00000041556 | *5_8S_rRNA* | 5.8S ribosomal RNA |
| ENSRNOG00000041610 | -- | -- |
| ENSRNOG00000042221 | *E9PSJ9_RAT* | RAF domain and POZ/BTB contair |
| ENSRNOG00000042221 | *E9PSJ9_RAT* | RAF domain and POZ/BTB contair |
| ENSRNOG00000042745 | *LOC682835* | Uncharacterized protein |
| ENSRNOG00000042745 | *LOC682835* | Uncharacterized protein |
| ENSRNOG00000042745 | *LOC682835* | Uncharacterized protein |
| ENSRNOG00000042884 | *D3ZCU5_RAT* | Uncharacterized protein |
| ENSRNOG00000043035 | *Fbxl13* | F-box and leucine-rich repeat pro |

| | | |
|---|---|---|
| ENSRNOG00000043035 | *Fbxl13* | F-box and leucine-rich repeat pro |
| ENSRNOG00000043173 | *F1LVU1_RAT* | Uncharacterized protein |
| ENSRNOG00000044911 | *7SK* | 7SK RNA |
| ENSRNOG00000045135 | *U6* | U6 spliceosomal RNA |

ne protein 3

ent protein 3

taining family H member 2
cid transporter 1
cid transporter 1

cotransporter 4
cotransporter 4

:haracterized protein
:haracterized protein

/l-CoA dehydrogenase

ppressor 2

\ member 1
\ member 1

ie methyltransferase
ie methyltransferase

ne protein 3
RGD1566169_predictedUncharacterized protein

ent protein 3

ne protein 3

:ed protein
atase-interacting protein 1

omains protein 3

type II-alpha regulatory subunit

ssed protein
ssed protein

ember 3

e 4

e 4

ı
ı
ı

ɔtein CASK
ɔtein CASK
ɔtein CASK
ɔtein CASK
ɔtein CASK
ɔtein CASK

:ein 1
:ein 1
:ein 1

ıit beta
og

ing protein 2

er 14

protein 2

ubunit A-like 1

'
; protein 2
se-2
se-2
se-2

ng protein 27
ng protein 27
51
protein 4
peptide 58
peptide 58
peptide 58
peptide 58
'
'

hosphatase O
hosphatase O
nase
nase
rotein 8E
TAO3

RGD1566226_predictedUncharacterized protein
e CWC27 homolog
ulatory subunit beta
ulatory subunit beta
ulatory subunit beta
x subunit 1B
itase 1
2

ubunit
dase 3
dase 3
dase 3
protein kinase kinase 2
protein kinase kinase 2
protein kinase kinase 2
protein kinase kinase 2
protein kinase kinase 2
omain-containing protein 3

e

1
1
1
1

б
б
б
13 (RGD1312026)

ated potassium channel protein 3
on factor ATF-6 alpha
2
2
atase 6 catalytic subunit

form 1
form 1
form 1
pendent dioxygenase ABH6
pendent dioxygenase ABH6

ase AER61

otein 8C
ein RTF1 homolog
ein RTF1 homolog
ein RTF1 homolog

enzyme 2 (Uba2)
enzyme 2 (Uba2)

.9 (Predicted)Uncharacterized protein

ıe-rich protein 2
ıe-rich protein 2

02 (RGD1306839)

mily member 6
mily member 6
mily member 6

mannosidase-like 2
C2
C2
C2
C2
C2
C2

otein 1

otein 1 homolog

se 1
se 1
se 1
homolog
homolog
homolog
homolog

-
-

in

ase RNF217
')
')
tor DBS
tor DBS
tor DBS
tor DBS
n-receptor type 12
n-receptor type 12

nscription factor
nscription factor

kinase
kinase
kinase
kinase
amily B member 1B allele A

racting protein 4
racting protein 4
racting protein 4
racting protein 4
racting protein 4
racting protein 4
racting protein 4

otor 146

rotein 2
rotein 2
rotein 2

e factor
e factor
omolog

RGD1562310_predictedUncharacterized protein
e

subunit RPB9
/11
/11
/11
/11
ma Fc region receptor III
repeats protein
repeats protein
repeats protein

1
1
cription factor
cription factor
cription factor
cription factor
cription factor

rotein

rotein
rotein
se 3

B
B
taining protein
taining protein

actor 2-alpha kinase 4
actor 2-alpha kinase 4

lehydrogenase
lehydrogenase

)
)
)
)
)
)
)
)
)
)

)
)
)
kinase kinase 1
kinase kinase 1
34

on stimulator
on stimulator

nosphatase mu
nosphatase mu

cium channel subunit alpha-1A
cium channel subunit alpha-1A
cium channel subunit alpha-1A
cium channel subunit alpha-1A

anger 2
anger 2

otein 146
or receptor 1
d protein kinase kinase 7
d protein kinase kinase 7

ein 16
ein 16
ein 16
ein 16

nase
nit 3

n stimulator-like 1
n stimulator-like 1
RIO3

subunit gamma-2
subunit gamma-2
3
3
3
1
1
1
1
tor
tor
tor
tor
tor
tor
tor
se 1
WNK1
WNK1
WNK1

anscription subunit 27
; 3
; 3
; 3
; 3

f transcription 4
f transcription 4

otransferase
otransferase
otransferase
otransferase
eta
eta
eta

otein 104
otein 104
otein 104
otein 104
otein 104
otein 104

onine-protein kinase 4
ier potassium channel 2
ier potassium channel 2

log
log
log

nel gamma-2 subunit

ated protein type 1

ivator 1
ivator 1
ivator 1
ivator 1

4
4

n 2
n 2
29

ence 1 homolog
ence 1 homolog
ence 1 homolog
ence 1 homolog
ence 1 homolog
ıg protein 2
ıg protein 2
ıg protein 2
ıg protein 2
ıg protein 2
ıg protein 2
ıg protein 2

2 isoform a
2 isoform a
2 isoform a
2 isoform a
2 isoform a
2 isoform a

mone receptor
mone receptor
mone receptor
mone receptor
mone receptor

mone receptor
mone receptor
mone receptor
mone receptor
mone receptor
eptor
eptor

lated transcript proteinCART(1-52)CART(55-102)CART(62-102)
lated transcript proteinCART(1-52)CART(55-102)CART(62-102)
activator 1 isoform 1
activator 1 isoform 1
activator 1 isoform 1
3

subfamily KQT member 1
subfamily KQT member 1

,
,
nnel subunit beta-4

'ase 13A5
nain-containing protein 4
nain-containing protein 4
nain-containing protein 4
nain-containing protein 4
nain-containing protein 4
nain-containing protein 4
nain-containing protein 4

otein 148
n 55LHAL tetrapeptideGPIPIRRH peptide
n 55LHAL tetrapeptideGPIPIRRH peptide
n 55LHAL tetrapeptideGPIPIRRH peptide
n 55LHAL tetrapeptideGPIPIRRH peptide

TNNI3K

n 71
rotein 6
rotein 6

DCLK3

ining protein T1
ining protein T1
ining protein T1
ining protein T1

x protein 3

ning protein T2
ning protein T2

tein 13

tein 13
