# Supplementary material for: A Bayesian Approach for Analysis of Whole-Genome Bisulfite Sequencing Data Identifies Disease-Associated Changes in DNA Methylation
Source: Genetics. 2017 Feb 16;205(4):1443–58. doi: 10.1534/genetics.116.195008 (PMC5378105; doi:10.1534/genetics.116.195008)
Supplement: Supplementary file 15 [file 1443TableS4.pdf]

Supplementary Table 4. List of DSS called deifferentailly methylated regions (DMRs; 500b

DSS Called Differentailly Methylated Regions

Chr.	Start Position (bp)	End Position (bp)	Number of CpG	Delta in Methylation (LEW-WKY)
18	74384212	74384517	9	0.55
12	45388863	45389260	8	-0.57
11	30968707	30968950	6	0.65
1	201199584	201200184	18	-0.53
17	3958378	3958755	13	0.51
12	9683275	9683841	14	-0.62
20	6208143	6208388	6	0.70
12	45388863	45389260	8	-0.57
12	9976121	9976446	10	0.53
11	45442813	45443476	6	-0.20
11	30968707	30968950	6	0.65
10	3768643	3769261	12	0.62
14	11926941	11927809	20	0.66
10	72117718	72117864	7	-0.57
13	43003365	43003604	7	0.81
7	24229425	24229683	10	0.50
14	77663267	77663426	6	-0.49
6	8144581	8144681	6	-0.50
5	48703345	48703503	6	-0.54
5	39763534	39763732	11	-0.50
10	72117718	72117864	7	-0.57
3	44701381	44702170	24	-0.68
8	31961263	31961315	9	0.57
8	54381850	54382069	11	0.76
8	63650013	63650447	9	-0.60
2	41515365	41515517	6	-0.47
8	47600316	47600708	7	0.66
16	79777986	79778329	11	0.62
3	59730733	59731057	8	0.63
9	14266318	14266855	18	0.56
19	48907678	48908091	16	0.69
16	70554885	70555098	6	-0.54
1	196783603	196784043	11	0.60
18	1054187	1054560	28	-0.46
18	1054187	1054560	28	-0.46
1	43174814	43175047	7	0.59
1	240595618	240595841	7	0.64
1	240595618	240595841	7	0.64
1	203418848	203418948	11	0.54
1	190990317	190990437	7	-0.58

16	58275643	58275834	6	-0.72
18	50390326	50390721	9	0.72
5	91565229	91565445	21	0.58
14	47278577	47278995	7	0.77
14	47282756	47283168	9	0.55
16	79777986	79778329	11	0.62
4	15764023	15764386	12	0.53
12	9683275	9683841	14	-0.62
13	23252336	23252549	8	0.55
20	12785392	12785843	20	-0.49
1	201199584	201200184	18	-0.53
12	1577590	1578072	19	0.75
17	3958378	3958755	13	0.51
1	201199584	201200184	18	-0.53

p window) that overlap with genes (n=45). The overlapping DMRs are those located wit

Length (bp)	Feature	Chr.	Start (bp)	End (bp)	Strand
306	downstream	18	74383471	74384471	+
397	exon	12	45388794	45388878	-
243	exon	11	30968926	30968988	-
600	exon	1	201199467	201199807	-
377	exon	17	3956809	3958985	+
566	exon	12	9683833	9683960	-
245	intron	20	6205766	6210798	+
397	intron	12	45387834	45391191	-
325	intron	12	9950380	10016402	+
663	intron	11	45440895	45472119	-
243	intron	11	30968210	30968926	-
618	intron	10	3765810	3787798	-
868	intron	14	11919526	11929689	+
146	intron	10	72115308	72117885	+
239	intron	13	42899039	43027499	+
258	intron	7	24225865	24230728	+
159	intron	14	77659217	77700245	+
100	intron	6	8142095	8144745	-
158	intron	5	48611899	48715885	+
198	intron	5	39747700	39789542	-
146	intron	10	72111149	72124567	-
789	intron	3	44693927	44721452	-
52	intron	8	31960461	31978095	+
219	intron	8	54381678	54382426	-
434	intron	8	63649705	63653719	+
152	intron	2	41478488	41571720	-
392	intron	8	47598339	47602062	+
343	intron	16	79744155	79910164	-
324	intron	3	59727962	59731721	-
537	intron	9	14222049	14362740	-
413	intron	19	48730700	48917387	+
213	intron	16	70554046	70556232	-
440	intron	1	196693498	196826546	+
373	intron	18	1009185	1152690	+
373	intron	18	1009185	1152690	+
233	intron	1	43167138	43274818	-
223	intron	1	240564622	240631888	+
223	intron	1	240564622	240631888	+
100	intron	1	203383721	203459580	+
120	intron	1	190987715	191006715	+

191	intron	16	58214917	58297046	+
395	intron	18	50365721	50406058	-
216	intron	5	91550057	91566087	+
418	intron	14	47266630	47293334	-
412	intron	14	47266630	47293334	-
343	intron	16	79742690	79841040	+
363	intron	4	15733080	15831387	-
566	intron	12	9674655	9686037	-
213	intron	13	23096577	23258406	+
451	intron	20	12784454	12786253	+
601	upstream	1	201199807	201200807	-
483	upstream	12	1577242	1578242	+
377	utr3	17	3957001	3958985	+
600	utr5	1	201199716	201199807	-

in ± 1kb from the gene boundaries.

Overlapping Genomic Feature		
Overlap with DMR (bp)	Overlapping Gene ID	Gene Symbol
259	ENSRNOG00000042957	*D3ZF23_RAT*
15	ENSRNOG00000000665	*Pitpnb*
24	ENSRNOG00000002051	*Synj1*
223	ENSRNOG00000015078	*Ifitm3*
377	ENSRNOG00000017613	*Pols*
8	ENSRNOG00000043178	*Znf655*
245	ENSRNOG00000000498	*Anks1a*
397	ENSRNOG00000000665	*Pitpnb*
325	ENSRNOG00000000999	*Smurf1*
663	ENSRNOG00000001616	*Senp7*
219	ENSRNOG00000002051	*Synj1*
618	ENSRNOG00000002294	*Snx29*
868	ENSRNOG00000002361	*Prkg2*
146	ENSRNOG00000002598	*F1LZC0_RAT*
239	ENSRNOG00000003878	*Thsd7b*
258	ENSRNOG00000004302	*Pah*
159	ENSRNOG00000005302	*Slc2a9*
100	ENSRNOG00000005877	*LPPRC_RAT*
158	ENSRNOG00000006170	*Bach2*
198	ENSRNOG00000007441	*Klhl32*
146	ENSRNOG00000007523	*Cct6b*
789	ENSRNOG00000007528	*Kcnh7*
52	ENSRNOG00000008709	*Rics*
219	ENSRNOG00000011228	*Layn*
434	ENSRNOG00000011619	*Myo9a*
152	ENSRNOG00000011623	*Rab3c*
392	ENSRNOG00000012453	*RGD1564560*
343	ENSRNOG00000012573	*Dlgap2*
324	ENSRNOG00000012580	*LOC311134*
537	ENSRNOG00000012708	*RGD1563020*
413	ENSRNOG00000014371	*Cdh13*
213	ENSRNOG00000014741	*D4A6Z8_RAT*
440	ENSRNOG00000016038	*Mgmt*
373	ENSRNOG00000016366	*Colec12*
373	ENSRNOG00000016366	*Colec12*
233	ENSRNOG00000018013	*PRKN2_RAT*
223	ENSRNOG00000018532	*E9PTD5_RAT*
223	ENSRNOG00000018532	*E9PTD5_RAT*
100	ENSRNOG00000020532	*Kcnq1*
120	ENSRNOG00000020624	*Acadsb*

191	ENSRNOG00000022374	*Sgcz*
395	ENSRNOG00000023197	*Znf608*
216	ENSRNOG00000024190	*LOC683719*
418	ENSRNOG00000026241	*D3ZY83_RAT*
412	ENSRNOG00000026241	*D3ZY83_RAT*
343	ENSRNOG00000038026	*F1M6B7_RAT*
363	ENSRNOG00000040029	*F1M3I5_RAT*
566	ENSRNOG00000043178	*Znf655*
213	ENSRNOG00000043185	*Cntnap5*
451	ENSRNOG00000043212	*Dip2a*
377	ENSRNOG00000015078	*Ifitm3*
482	ENSRNOG00000028892	*D3ZUH5_RAT*
377	ENSRNOG00000017613	*Pols*
91	ENSRNOG00000015078	*Ifitm3*

Gene Name
Uncharacterized protein
Phosphatidylinositol transfer protein beta isoform
Synaptojanin-1
Interferon-induced transmembrane protein 3
DNA polymerase sigma
zinc finger protein 655
ankyrin repeat and sterile alpha motif domain containing 1A
Phosphatidylinositol transfer protein beta isoform
similar to RIKEN cDNA 4930431E10 (RGD1309707), mRNA
Sentrin-specific protease 7
Synaptojanin-1
sorting nexin-29
cGMP-dependent protein kinase 2
Uncharacterized protein
thrombospondin type-1 domain-containing protein 7B
Phenylalanine-4-hydroxylase
solute carrier family 2, facilitated glucose transporter member 9
Leucine-rich PPR motif-containing protein, mitochondrial
transcription regulator protein BACH2
Uncharacterized protein
T-complex protein 1 subunit zeta-2
Potassium voltage-gated channel subfamily H member 7
Uncharacterized protein
layilin
Myosin-IXa
Ras-related protein Rab-3C
similar to RCK (RGD1564560), mRNA
Disks large-associated protein 2
Uncharacterized protein
Uncharacterized protein
cadherin-13
Uncharacterized protein
Methylated-DNA--protein-cysteine methyltransferase
Collectin-12
Collectin-12
E3 ubiquitin-protein ligase parkin
polycomb group RING finger protein 5
polycomb group RING finger protein 5
Potassium voltage-gated channel subfamily KQT member 1
Short/branched chain specific acyl-CoA dehydrogenase, mitochondrial

sarcoglycan zeta
zinc finger protein 608
Uncharacterized protein
Uncharacterized protein
Uncharacterized protein
Uncharacterized protein
Uncharacterized protein
zinc finger protein 655
Contactin-associated protein like 5-4
disco-interacting protein 2 homolog A
Interferon-induced transmembrane protein 3
Uncharacterized protein
DNA polymerase sigma
Interferon-induced transmembrane protein 3
