# Supplementary material for: A Bayesian Approach for Analysis of Whole-Genome Bisulfite Sequencing Data Identifies Disease-Associated Changes in DNA Methylation
Source: Genetics. 2017 Feb 16;205(4):1443–58. doi: 10.1534/genetics.116.195008 (PMC5378105; doi:10.1534/genetics.116.195008)
Supplement: Supplementary file 16 [file 1443TableS5.pdf]

**Gene Name**
Abca1
Acadsb
Amotl1
Btd
Cd300lg
Cped1
Cpne5
Ctse
Cyb561a3
D3ZGN7_RAT
Dntt
Ephb2
Fgf12
Folr1
Gcom1
Ifitm3
Kansl1l
Kif7
Lzts2
Maff
Man1c1
Mcoln2
Mcoln3
Mgmt
Mmp11
Myo1d
Myo5b
Pithd1
Plekhh2
RGD1309870
Rhbdl3
Rin2
Rnaset2
Rnpep
Scd1
Sept1
She
Slc38a1
Slc4a5
Smtn

Ston2
Svep1
Thrb
Tmem82
Treml1
U1
Ydjc

Gene Description
ATP-binding cassette, subfamily A (ABC1), member 1
acyl-CoA dehydrogenase, short/branched chain
angiomotin-like 1
biotinidase
Cd300 molecule-like family member G
cadherin-like and PC-esterase domain containing 1
copine V
cathepsin E
cytochrome b561 family, member A3
Uncharacterized protein
DNA nucleotidylexotransferase
Eph receptor B2
fibroblast growth factor 12
folate receptor 1 (adult)
GRINL1A complex locus protein 1
interferon induced transmembrane protein 3
KAT8 regulatory NSL complex subunit 1-like
kinesin family member 7
leucine zipper, putative tumor suppressor 2
v-maf avian musculoaponeurotic fibrosarcoma oncogene homolog F
mannosidase, alpha, class 1C, member 1
mucolipin 2
mucolipin 3
O-6-methylguanine-DNA methyltransferase
matrix metallopeptidase 11
myosin ID
myosin Vb
PITH (C-terminal proteasome-interacting domain of thioredoxin-like) domain containing 1
pleckstrin homology domain containing, family H (with MyTH4 domain) member 2
hypothetical LOC289778
rhomboid, veinlet-like 3 (Drosophila)
Ras and Rab interactor 2
ribonuclease T2
arginyl aminopeptidase (aminopeptidase B)
stearoyl-Coenzyme A desaturase 1
septin 1
Src homology 2 domain containing E
solute carrier family 38, member 1
solute carrier family 4, sodium bicarbonate cotransporter, member 5
smoothelin

stonin 2
sushi, von Willebrand factor type A, EGF and pentraxin domain containing 1
thyroid hormone receptor beta
transmembrane protein 82
triggering receptor expressed on myeloid cells-like 1
U1 spliceosomal RNA
YdjC homolog (bacterial)

/KY and LEW rat strains that are also di

Ensembl Gene ID	CHR
ENSRNOG00000018126	chr5
ENSRNOG00000020624	chr1
ENSRNOG00000008990	chr8
ENSRNOG00000019656	chr16
ENSRNOG00000020837	chr10
ENSRNOG00000021840	chr4
ENSRNOG00000000522	chr20
ENSRNOG00000006963	chr13
ENSRNOG00000020702	chr1
ENSRNOG00000027841	chr4
ENSRNOG00000013615	chr1
ENSRNOG00000012531	chr5
ENSRNOG00000001931	chr11
ENSRNOG00000019902	chr1
ENSRNOG00000017202	chr8
ENSRNOG00000015078	chr1
ENSRNOG00000028149	chr9
ENSRNOG00000026857	chr1
ENSRNOG00000014969	chr1
ENSRNOG00000012886	chr7
ENSRNOG00000017087	chr5
ENSRNOG00000015089	chr2
ENSRNOG00000015024	chr2
ENSRNOG00000016038	chr1
ENSRNOG00000028344	chr20
ENSRNOG00000003276	chr10
ENSRNOG00000014104	chr18
ENSRNOG00000010873	chr5
ENSRNOG00000005124	chr6
ENSRNOG00000037621	chr14
ENSRNOG00000005515	chr10
ENSRNOG00000010131	chr3
ENSRNOG00000013190	chr1
ENSRNOG00000006720	chr13
ENSRNOG00000013552	chr1
ENSRNOG00000017804	chr1
ENSRNOG00000020797	chr2
ENSRNOG00000005291	chr7
ENSRNOG00000010378	chr4
ENSRNOG00000019451	chr14

ENSRNOG00000004458	chr6
ENSRNOG00000033110	chr5
ENSRNOG00000006649	chr15
ENSRNOG00000011970	chr5
ENSRNOG00000040109	chr9
ENSRNOG00000035334	chr2
ENSRNOG00000001861	chr11

fferentially expressed between the two strains in the same tissue.

DMR Gene overlap
70555077-70555215
190990317-190990437
11391719-11392350
7121594-7121762
91104033-91104084/91104084-91104178/91103844-91104033/91104033-91104041
48334740-48334986
7471342-7471507/7471342-7471507
44601546-44601743
213078596-213079072/213078596-213079072
157369967-157369979
246217613-246217908
155446650-155447336/155447682-155447726/155578344-155578692
74423528-74423536
159315263-159315432/159315263-159315300
75999078-75999242
201199522-201199807/201199807-201200184/201199716-201199807
65578002-65578018/65578002-65578018
135484912-135485255
250085410-250085533
117335736-117336128
153336351-153336935/153345531-153346146
244382399-244382456/244382223-244382399
244285393-244285537/244286406-244286792
196783603-196784043
13130833-13131181
68789450-68789566/68789190-68789566
71444981-71445138
154761710-154762122
7949640-7949973
92374023-92374031
68549388-68549672
134342611-134342621/134416266-134416684
47244465-47244525
48283546-48283871
249471145-249471156/249470979-249471145
186478917-186479089
182060749-182061173
135296975-135297088
117447942-117448201
84172871-84173275/84173275-84173369/84173369-84173390

115280790-115281128
76087419-76087478/76088075-76088118/76087478-76088075/76203959-76203980
9324042-9324638
160519056-160519285
8052189-8052235/8052044-8052189
244285393-244285537
85848019-85848083/85847836-85848019/85847836-85848164/85848019-85848083

Average methylation difference	Gene feature	$\log_2$ fold change (diff. expression)
-0.55	intron	0.77
-0.65	intron	1.13
-0.40	intron	-2.56
-0.49	intron	0.81
-0.43/-0.43/-0.43/-0.43	exon/intron/upstream/5' UTR	3.42
0.38	intron	-2.27
-0.41/-0.41	downstream/intron	-1.95
-0.34	intron	-1.05
-0.37/-0.37	exon/3' UTR	-1.03
0.38	intron	-1.23
-0.42	intron	4.31
0.35/0.36/0.40	intron/intron/intron	-5.29
0.35	intron	3.21
-0.40/-0.40	exon/3' UTR	-1.58
0.48	intron	-2.78
-0.39/-0.39/-0.39	exon/upstream/5' UTR	3.62
0.38/0.38	end/intron	1.30
-0.35	exon	1.37
0.35	intron	-0.97
0.35	intron	-2.66
-0.43/-0.46	intron/intron	1.04
-0.49/-0.489	exon/intron	-1.22
-0.35/0.40	intron/intron	-2.63
0.38	intron	1.06
-0.34	intron	-1.81
0.39/0.39	downstream/intron	-2.94
-0.44	intron	-3.46
0.36	intron	0.82
0.43	intron	-5.27
0.37	intron	-4.04
-0.38	intron	6.22
0.43/0.40	intron/intron	0.96
0.40	exon	0.80
0.44	intron	-0.86
0.36/0.36	exon/intron	-1.68
-0.34	up	-2.01
-0.41	intron	2.18
-0.42	intron	1.75
0.43	intron	-1.37
0.49/0.49/0.49	exon/intron/exon	-2.02

-0.38	intron	-1.62
-0.45/-0.45/-0.45/0.34	exon/exon/intron/upstream	1.37
-0.39	intron	1.20
-0.40	up	-3.02
-0.37/-0.37	exon/intron	1.90
-0.35	up	-8.45
0.41/0.41/0.41/0.41	exon/intron/upstream/5' UTR	-1.91

adjusted p-value (diff. expression)
0.014
0.002
0.001
0.038
0.011
2.74E-04
0.049
0.005
0.011
0.029
1.25E-15
8.28E-05
0.032
0.002
0.007
1.71E-08
0.008
5.80E-06
0.007
5.90E-12
0.006
0.002
2.42E-19
0.034
0.040
6.45E-24
5.37E-05
0.030
6.75E-09
1.41E-05
1.08E-40
0.028
0.029
0.049
0.027
0.001
0.012
2.60E-08
1.86E-04
8.91E-06

0.012
0.015
0.032
1.87E-04
0.002
0.008
5.69E-09
